# Supplementary material for: New insights into the genetic etiology of Alzheimer’s disease and related dementias
Source: Nat Genet. 2022 Apr 4;54(4):412–36. doi: 10.1038/s41588-022-01024-z (PMC9005347; doi:10.1038/s41588-022-01024-z)

nature portfolio

https://doi.org/10.1038/s41588-022-01024-z

Supplementary information

New insights into the genetic etiology of Alzheimer's disease and related dementias

In the format provided by the authors and unedited

Uncorrected proofs

New insights into the genetic etiology of Alzheimer's disease and related dementias

Céline Bellenguez[1,*,#], Fahri Küçükali[2,3,4,*], Iris E. Jansen[5,6,*], Luca Kleineidam[7,8,9,*], Sonia Moreno-Grau[10,11,*], Najaf Amin[12,13,*], Adam C. Naj[14,15,*], Rafael Campos-Martin[8,*], Benjamin Grenier-Boley[1], Victor Andrade[7,8], Peter A. Holmans[16], Anne Boland[17], Vincent Damotte[1], Sven J. van der Lee[5,18], Marcos R. Costa[1,19], Teemu Kuulasmaa[20], Qiong Yang[21,22], Itziar de Rojas[10,11], Joshua C. Bis[23], Amber Yaqub[12], Ivana Prokic[12], Julien Chapuis[1], Shahzad Ahmad[12,24], Vilmantas Giedraitis[25], Dag Aarsland[26,27], Pablo Garcia-Gonzalez[10,11], Carla Abdelnour[10,11], Emilio Alarcón-Martín[10,28], Daniel Alcolea[11,29], Montserrat Alegret[10,11], Ignacio Alvarez[30,31], Victoria Álvarez[32,33], Nicola J. Armstrong[34], Anthoula Tsolaki[35,36], Carmen Antúnez[37], Ildebrando Appollonio[38,39], Marina Arcaro[40], Silvana Archetti[41], Alfonso Arias Pastor[42,43], Beatrice Arosio[44,45], Lavinia Athanasiu[46], Henri Bailly[47], Nerisa Banaj[48], Miquel Baquero[49], Sandra Barral[50,184,188], Alexa Beiser[20,51], Ana Belén Pastor[52], Jennifer E. Below[53], Penelope Benchek[54,55], Luisa Benussi[56], Claudine Berr[57], Céline Besse[17], Valentina Bessi[58,59], Giuliano Binetti[56,60], Alessandra Bizarro[61], Rafael Blesa[11,29], Mercè Boada[10,11], Eric Boerwinkle[62,63], Barbara Borroni[64], Silvia Boschi[65], Paola Bossù[66], Geir Bråthen[67,68], Jan Bressler[62,69], Catherine Bresner[16], Henry Brodaty[34,70], Keeley J. Brookes[71], Luis Ignacio Brusco[72,73,74], Dolores Buiza-Rueda[11,191], Katharina Bûrger[75,76], Vanessa Burholt[77,78], William S. Bush[79], Miguel Calero[10,52,80], Laura B. Cantwell[81], Geneviève Chene[82,83], Jaeyoon Chung[84], Michael L. Cuccaro[85], Ángel Carracedo[86,87], Roberta Cecchetti[88], Laura Cervera-Carles[11,29], Camille Charbonnier[89], Hung-Hsin Chen[90], Caterina Chillotti[91], Simona Ciccone[45], Jurgen A.H.R. Claassen[92], Christopher Clark[93], Elisa Conti[38], Anaïs Corma-Gómez[94], Emanuele Costantini[95], Carlo Custodero[96], Delphine Daian[17], Maria Carolina Dalmasso[8], Antonio Daniele[95], Efthimios Dardiotis[97], Jean-François Dartigues[98], Peter Paul de Deyn[99], Katia de Paiva Lopes[101,102,103,104], Lot D. de Witte[104], Stéphanie Debette[98,105], Jürgen Deckert[106], Teodoro del Ser[52], Nicola Denning[107], Anita DeStefano[20,21,108], Martin Dichgans[75,75,109], Janine Diehl-Schmid[110], Mónica Diez-Fairen[30,31], Paolo Dionigi Rossi[45], Srdjan Djurovic[46], Emmanuelle Duron[47], Emrah Düzel[111,112], Carole Dufouil[82,83], Gudny Eiriksdottir[113], Sebastiaan Engelborghs[114,115,116,117], Valentina Escott-Price[15,107], Ana Espinosa[10,11], Michael Ewers[75,76], Kelley M. Faber[118], Tagliavini Fabrizio[119], Sune Fallgaard Nielsen[120], David W. Fardo[121], Lucia Farotti[122], Chiara Fenoglio[123], Marta Fernández-Fuertes[94], Raffaele Ferrari[124,125], Catarina B Ferreira[126], Evelyn Ferri[45], Bertrand Fin[17], Peter Fischer[127], Tormod Fladby[128], Klaus Fließbach[8,9], Bernard Fongang[129], Myriam Fornage[68,69], Juan Fortea[11,29], Tatiana M. Foroud[118], Silvia Fostinelli[56], Nick C. Fox[130], Emlio Franco-Macías[131], María J. Bullido[11,132,133], Ana Frank-García[11,132,134], Lutz Froelich[135], Brian Fulton-Howard[136], Daniela Galimberti[40,123], Jose Maria García-Alberca[11,137], Pablo García-González[10], Sebastian Garcia-Madrona[138], Guillermo Garcia-Ribas[138], Roberta Ghidoni[56], Ina Giegling[139], Giaccone Giorgio[119], Alison M. Goate[136], Oliver Goldhardt[110], Duber Gomez-Fonseca[140], Antonio González-Pérez[141], Caroline Graff[142,143], Giulia Grande[144], Emma Green[145], Timo Grimmer[110], Edna Grünblatt[146,147,148], Michelle Grunin[55], Vilmundur Gudnason[149], Tamar Guetta-Baranes[150], Annakaisa Haapasalo[151], Georgios Hadjigeorgiou[152], Jonathan L. Haines[79], Kara L. Hamilton-Nelson[153], Harald Hampel[154], Olivier Hanon[47], John Hardy[125], Annette M. Hartmann[139], Lucrezia Hausner[135], Janet Harwood[16], Stefanie Heilmann-Heimbach[155], Seppo Helisalmi[156,157], Michael T. Heneka[7,9], Isabel Hernández[10,11], Martin J. Herrmann[106], Per Hoffmann[155], Clive Holmes[158], Henne Holstege[5,18], Raquel Huerto Vilas[42,43], Marc Hulsman[5,18], Jack Humphrey[100,101,102,103], Geert Jan Biessels[159], Xueqiu Jian[129], Charlotte Johansson[142], Gyungah R. Jun[84], Yuriko Kastumata[160], John Kauwe[161], Patrick G. Kehoe[162], Lena Kilander[21], Anne Kinhult Ståhlbom[142], Miia Kivipelto[163,164,165,166], Anne Koivisto[156,167,168], Johannes Kornhuber[169], Mary H. Kosmidis[170], Walter A. Kukull[171], Pavel P. Kuksa[15], Brian W. Kunkle[152], Amanda B. Kuzma[172], Carmen Lage[11,173], Erika J Laukka[144,174], Lenore Launer[175,176], Alessandra Lauria[61], Chien-Yueh Lee[15], Jenni Lehtisalo[156,200], Ondrej Lerch[177,178], Alberto Lleó[11,29], William Longstreth Jr[179], Oscar Lopez[22], Adolfo Lopez de Munain[11,180], Seth Love[162], Malin Löwemark[21], Lauren Luckcuck[16], Kathryn L. Lunetta[20], Yiyi Ma[18,182], Juan Macías[94], Catherine A. MacLeod[183], Wolfgang Maier[7,9], Francesca Mangialasche[163], Marco Spallazzi[184], Marta Marquié[10,11], Rachel Marshall[16], Eden R. Martin[153], Angel Martín Montes[11,132,134], Carmen Martínez Rodríguez[33], Carlo Masullo[187], Richard Mayeux[50,186], Simon Mead[187], Patrizia Mecocci[88], Miguel Medina[11,52], Alun Meggy[107], Shima Mehrabian[188], Silvia Mendoza[137], Manuel Menéndez-González[33], Pablo Mir[11,189],

Susanne Moebus[190], Merel Mol[191], Laura Molina-Porcel[192,193], Laura Montrreal[10], Laura Morelli[194], Fermin Moreno[11,180], Kevin Morgan[195], Thomas Mosley[196], Markus M Nöthen[155], Carolina Muchnik[72,197], Shubhabrata Mukherjee[198], Benedetta Nacmias[58,199], Tiia Ngandu[200], Gael Nicolas[89], Børge G. Nordestgaard[201,202], Robert Olaso[17], Adelina Orellana[10,11], Michela Orsini[95], Gemma Ortega[10,11], Alessandro Padovani[63], Caffarra Paolo[203], Goran Papenberg[144], Lucilla Parnetti[122], Florence Pasquier[204], Pau Pastor[30,31], Gina Peloso[20,51], Alba Pérez-Cordón[10], Jordi Pérez-Tur[11,205,206], Pierre Pericard[207], Oliver Peters[208,209], Yolande A.L. Pijnenburg[5], Juan A Pineda[94], Gerard Piñol-Ripoll[42,43], Claudia Pisanu[210], Thomas Polak[106], Julius Popp[211,212,213], Danielle Posthuma[6], Josef Priller[209,214], Raquel Puerta[10], Olivier Quenez[89], Inés Quintela[86], Jesper Qvist Thomassen[215], Alberto Rábano[11,52], Innocenzo Rainero[64], Farid Rajabli[153], Inez Ramakers[216], Luis M Real[94,217], Marcel J.T. Reinders[218], Christiane Reitz[181,186,218], Dolly Reyes-Dumeyer[181,182], Perry Ridge[219], Steffi Riedel-Heller[220], Peter Riederer[221], Natalia Roberto[10], Eloy Rodriguez-Rodriguez[11,173], Arvid Rongve[222,223], Irene Rosas Allende[32,33], Maitée Rosende-Roca[10,11], Jose Luis Royo[224], Elisa Rubino[225], Dan Rujescu[139], María Eugenia Sáez[141], Paraskevi Sakka[226], Ingvild Saltvedt[67,227], Ángela Sanabria[10,11], María Bernal Sánchez-Arjona[131], Florentino Sanchez-Garcia[228], Pascual Sánchez Juan[11,173], Raquel Sánchez-Valle[229], Sigrid B Sando[66,67], Chloé Sarnowski[62], Claudia L. Satizabal[21,108,129], Michela Scamosci[88], Nikolaos Scarmeas[50,230], Elio Scarpini[40,123], Philip Scheltens[5], Norbert Scherbaum[231], Martin Scherer[232], Matthias Schmid[9,233], Anja Schneider[7,9], Jonathan M. Schott[130], Geir Selbæk[128,234], Davide Seripa[235], Manuel Serrano[236], Jin Sha[14], Alexey A Shadrin[46], Olivia Skrobot[162], Susan Slifer[153], Gijsje J. L. Snijders[104], Hilkka Soininen[156], Vincenzo Solfrizzi[96], Alina Solomon[156,163], Yeunjoo Song[55], Sandro Sorbi[58,199], Oscar Sotolongo-Grau[10], Gianfranco Spalletta[48], Annika Spottke[9,237], Alessio Squassina[238], Eystein Stordal[239], Juan Pablo Tartan[10], Lluís Tárraga[10,11], Niccolo Tesí[5,18], Anbupalam Thalamuthu[34], Tegos Thomas[35,36], Giuseppe Tosto[50,182], Latchezar Traykov[188], Lucio Tremolizzo[38,39], Anne Tybjærg-Hansen[202,215], Andre Uitterlinden[240], Abbe Ullgren[142], Ingun Ulstein[234], Sergi Valero[10,11], Otto Valladares[15], Christine Van Broeckhoven[2,3,241], Jeffery Vance[85], Badri N. Vardarajan[50], Aad van der Lugt[242], Jasper Van Dongen[2,3,4], Jeroen van Rooij[191,242], John van Swieten[191], Rik Vandenberghe[243,244], Frans Verhey[216], Jean-Sébastien Vidal[47], Jonathan Vogelgsang[245,246], Martin Vyhnalek[177,178], Michael Wagner[7,9], David Wallon[247], Li-San Wang[15], Ruiqi Wang[20,21], Leonie Weinhold[233], Jens Wiltfang[245,248,249], Gill Windle[183], Bob Woods[183], Mary Yannakoulia[250], Habil Zare[129], Yi Zhao[15], Xiaoling Zhang[251,252], Congcong Zhu[251], Miren Zulaica[11,253], EADB[254], Gra@ce[254], Degesco[254], ADGC[254], Charge[254], EADI[254], GERAD[254], DemGen[254], FinnGen[254], Lindsay A. Farrer[20,84,108], Bruce M. Psaty[22,172,255], Mohsen Ghanbari[12], Towfique Raj[100,101,102,103], Perminder Sachdev[34], Karen Mather[34], Frank Jessen[7,9], M. Arfan Ikram[12], Alexandre de Mendonça[126], Jakub Hort[176,177], Tsolaki Magda[35,36], Margaret A. Pericak-Vance[151], Philippe Amouyel[1], Julie Williams[16,107], Ruth Frikke-Schmidt[202,215], Jordi Clarimon[11,29], Jean-François Deleuze[17], Giacomina Rossi[119], Sudha Seshadri[21,108,129], Ole A. Andreassen[46], Martin Ingelsson[25], Mikko Hiltunen[19,**], Kristel Sleegers[2,3,4,**], Gerard D. Schellenberg[15,**], Cornelia M. van Duijn[12,13,**], Rebecca Sims[16,**], Wiesje M. van der Flier[5,**], Agustín Ruiz[10,11,**], Alfredo Ramirez[7,8,9,129,256**,] Jean-Charles Lambert[1,**,#]

* These authors contributed equally
** These authors jointly supervised this work
# corresponding authors:

Céline Bellenguez: celine.bellenguez@pasteur-lille.fr
Jean-Charles Lambert: jean-charles.lambert@pasteur-lille.fr

1. Univ. Lille, Inserm, CHU Lille, Institut Pasteur Lille, U1167-RID-AGE - Facteurs de risque et déterminants moléculaires des maladies liées au vieillissement, F-59000 Lille, France
2. Complex Genetics of Alzheimer's Disease Group, VIB Center for Molecular Neurology, VIB, Antwerp, Belgium
3. Laboratory of Neurogenetics, Institute Born - Bunge, Antwerp, Belgium
4. Department of Biomedical Sciences, University of Antwerp, Antwerp, Belgium
5. Alzheimer Center Amsterdam, Department of Neurology, Amsterdam Neuroscience, Vrije Universiteit Amsterdam, Amsterdam UMC, Amsterdam, The Netherlands
6. Department of Complex Trait Genetics, Center for Neurogenomics and Cognitive Research, Amsterdam Neuroscience, Vrije University, Amsterdam, The Netherlands.
7. Department of Neurodegenerative Diseases and Geriatric Psychiatry, University Hospital Bonn, Bonn, Germany
8. Division of Neurogenetics and Molecular Psychiatry, Department of Psychiatry and Psychotherapy, University of Cologne, Medical Faculty, Cologne, Germany.
9. German Center for Neurodegenerative Diseases (DZNE Bonn), Bonn, Germany
10. Research Center and Memory clinic Fundació ACE, Institut Català de Neurociències Aplicades, Universitat Internacional de Catalunya, Barcelona, Spain
11. CIBERNED, Network Center for Biomedical Research in Neurodegenerative Diseases, National Institute of Health Carlos III, Madrid, Spain
12. Department of Epidemiology, ErasmusMC, Rotterdam, The Netherlands
13. Nuffield Department of Population Health Oxford University, Oxford, UK
14. Department of Biostatistics, Epidemiology, and Informatics; Penn Neurodegeneration Genomics Center, University of Pennsylvania Perelman School of Medicine, Philadelphia, Pennsylvania, USA
15. Department of Pathology and Laboratory Medicine, University of Pennsylvania Perelman School of Medicine, Philadelphia, Pennsylvania, USA
16. MRC Centre for Neuropsychiatric Genetics and Genomics, Division of Psychological Medicine and Clinical Neuroscience, School of Medicine, Cardiff University, Cardiff, UK
17. Université Paris-Saclay, CEA, Centre National de Recherche en Génomique Humaine, 91057, Evry, France
18. Section Genomics of Neurodegenerative Diseases and Aging, Department of Human Genetics Amsterdam UMC, Vrije Universiteit Amsterdam, Amsterdam UMC, Amsterdam, The Netherlands
19. Brain Institute, Federal University of Rio Grande do Norte, Av. Nascimento de Castro 2155 Natal, Brazil
20. Institute of Biomedicine, University of Eastern Finland, Kuopio, Finland
21. Department of Biostatistics, Boston University School of Public Health, Boston, MA, USA.
22. Framingham Heart Study, Framingham, MA, USA.
23. Cardiovascular Health Research Unit, Department of Medicine, University of Washington, Seattle, WA, USA.
24. LACDR, Leiden, The Netherlands
25. Dept.of Public Health and Carins Sciences / Geriatrics, Uppsala University
26. Centre of Age-Related Medicine, Stavanger University Hospital, Norway
27. Institute of Psychiatry, Psychology & Neuroscience, PO 70, 16 De Crespigny Park, London, UK
28. Department of Surgery, Biochemistry and Molecular Biology, School of Medicine, University of Málaga, Málaga, Spain.
29. Department of Neurology, II B Sant Pau, Hospital de la Santa Creu i Sant Pau, Universitat Autònoma de Barcelona, Barcelona, Spain.
30. Fundació Docència i Recerca MútuaTerrassa and Movement Disorders Unit, Department of Neurology, University Hospital MútuaTerrassa, Terrassa 08221, Barcelona, Spain
31. Memory Disorders Unit, Department of Neurology, Hospital Universitari Mutua de Terrassa, Terrassa, Barcelona, Spain
32. Laboratorio de Genética. Hospital Universitario Central de Asturias, Oviedo, Spain
33. Servicio de Neurología HOspital Universitario Central de Asturias- Oviedo and Instituto de Investigación Biosanitaria del Principado de Asturias, Oviedo, Spain
34. Centre for Healthy Brain Ageing, School of Psychiatry, Faculty of Medicine, University of New South Wales, Sydney, Australia
35. 1st Department of Neurology, Medical school, Aristotle University of Thessaloniki, Thessaloniki, Makedonia, Greece
36. Alzheimer Hellas, Thessaloniki, Makedonia, Greece
37. Unidad de Demencias, Hospital Clínico Universitario Virgen de la Arrixaca, Spain
38. School of Medicine and Surgery, University of Milano-Bicocca, Italy
39. Neurology Unit, "San Gerardo" hospital, Monza, Italy
40. Fondazione IRCCS Ca' Granda, Ospedale Policlinico, Milan, Italy
41. Department of Laboratory Diagnostics, III Laboratory of Analysis, Brescia Hospital, Brescia, Italy
42. Unitat Trastorns Cognitius, Hospital Universitari Santa Maria de Lleida, Lleida, Spain
43. Institut de Recerca Biomedica de Lleida (IRBLLeida), Lleida, Spain
44. Department of Clinical Sciences and Community Health, University of Milan, Italy
45. Geriatic Unit, Fondazione Cà Granda, IRCCS Ospedale Maggiore Policlinico, Milan, Italy
46. NORMENT Centre, University of Oslo, Oslo, Norway
47. Université de Paris, EA 4468, APHP, Hôpital Broca, Paris, France
48. Laboratory of Neuropsychiatry, Department of Clinical and Behavioral Neurology, IRCCS Santa Lucia Foundation, Rome, Italy
49. Servei de Neurologia, Hospital Universitari i Politècnic La Fe, Valencia, Spain
50. Taub Institute on Alzheimer's Disease and the Aging Brain, Department of Neurology, Columbia University, New York, New York, USA

51. Boston University and the NHLBI's Framingham Heart Study, Boston, MA, USA
52. CIEN Foundation/Queen Sofia Foundation Alzheimer Center, Madrid, Spain
53. Vanderbilt Brain Institute, Vanderbilt University, Nashville, Tennesee, USA
54. Cleveland Institute for Computational Biology Case Western Reserve University, Cleveland, Ohio, USA
55. Department of population and Quantitative Health Sciences Case Western Reserve University, Cleveland, Ohio, USA
56. Molecular Markers Laboratory, IRCCS Istituto Centro San Giovanni di Dio Fatebenefratelli, Brescia, Italy
57. Univ. Montpellier, Inserm U1061, Neuropsychiatry: epidemiological and clinical research, PSNREC, Montpellier, France
58. Department of Neuroscience, Psychology, Drug Research and Child Health University of Florence, Florence Italy
59. Azienda Ospedaliero-Universitaria Careggi, Florence, Italy
60. MAC - Memory Clinic, IRCCS Istituto Centro San Giovanni di Dio Fatebenefratelli, Brescia
61. Geriatrics Unit Fondazione Policlinico A. Gemelli IRCCS, Rome, Italy
62. Human Genetics Center, School of Public Health, University of Texas Health Science Center at Houston, Houston, TX, USA
63. Human Genome Sequencing Center, Baylor College of Medicine, Houston, TX, USA
64. Centre for Neurodegenerative Disorders, Department of Clinical and Experimental Sciences, University of Brescia, Brescia, Italy
65. Department of Neuroscience “Rita Levi Montalcini”, University of Torino, Torino, Italy
66. Experimental Neuro-psychobiology Laboratory, Department of Clinical and Behavioral Neurology, IRCCS Santa Lucia Foundation, Rome, Italy
67. Department of Neurology and Clinical Neurophysiology, University Hospital of Trondheim, Trondheim, Norway
68. Department of Neuromedicine and Movement Science, Norwegian University of Science and Technology, Trondheim, Norway
69. University of Texas Health Science Center at Houston School of Public Health, TX, USA
70. Dementia Centre for Research Collaboration, School of Psychiatry, University of New South Wales, Sydney, Australia
71. Biosciences, School of Science and Technology, Nottingham Trent University, Nottingham UK
72. Centro de Neuropsiquiatría y Neurología de la Conducta (CENECON), Facultad de Medicina, Universidad de Buenos Aires (UBA), C.A.B.A, Buenos Aires, Argentina.
73. Departamento Ciencias Fisiológicas UAII, Facultad de Medicina, UBA, C.A.B.A, Buenos Aires, Argentina
74. Hospital Interzonal General de Agudos Eva Perón, San Martín, Buenos Aires, Argentina
75. Institute for Stroke and Dementia Research, Klinikum der Universität München, Ludwig-Maximilians-Universität LMU, Munich, Germany
76. German Center for Neurodegenerative Diseases (DZNE, Munich), Munich, Germany.
77. Faculty of Medical & Health Sciences, University of Auckland, New Zealand
78. Wales Centre for Ageing & Dementia Research, Swansea University, Wales, New Zealand
79. Department of Population & Quantitative Health Sciences, Case Western Reserve University, Cleveland, Ohio, USA
80. UFIEC, Instituto de Salud Carlos III, Madrid, Spain
81. Department of Pathology and Laboratory Medicine University of Pennsylvania, Philadelphia, PA, USA
82. Inserm, Bordeaux Population Health Research Center, UMR 1219, Univ. Bordeaux, ISPED, CIC 1401-EC, Univ Bordeaux, Bordeaux, France
83. CHU de Bordeaux, Pole santé publique, Bordeaux, France
84. Medicine Biomedical Genetics Boston University School of Medicine, Boston, MA, US
85. Dr. John T. Macdonald Foundation Department of Human Genetics, University of Miami, Miami, Florida, USA
86. Grupo de Medicina Xenómica, Centro Nacional de Genotipado (CEGEN-PRB3-ISCIII). Universidade de Santiago de Compostela, Santiago de Compostela, Spain
87. Fundación Pública Galega de Medicina Xenómica- CIBERER-IDIS, University of Santiago de Compostela, Santiago de Compostela, Spain
88. Institute of Gerontology and Geriatrics, Department of Medicine and Surgery, University of Perugia, Perugia, Italy
89. Normandie Univ, UNIROUEN, Inserm U1245 and CHU Rouen, Department of Genetics and CNR-MAJ, Rouen, France
90. Division of Genetic Medicine, Vanderbilt University, Nashville, Tennesee, USA
91. Unit of Clinical Pharmacology, University Hospital of Cagliari, Cagliari, Italy
92. Radboudumc Alzheimer Center, Department of Geriatrics, Radboud University Medical Center, Nijmegen, the Netherlands
93. Institute for Regenerative Medicine, University of Zürich, Schlieren, Switzerland
94. Unidad Clínica de Enfermedades Infecciosas y Microbiología. Hospital Universitario de Valme, Sevilla, Spain
95. Department of Neuroscience, Catholic University of Sacred Heart, Fondazione Policlinico Universitario A. Gemelli IRCCS, Rome, Italy
96. University of Bari, “A. Moro”, Bary, Italy
97. School of Medicine, University of Thessaly, Larissa, Greece
98. University Bordeaux, Inserm, Bordeaux Population Health Research Center, France
99. Department of Neurology, University Medical Center Groningen, the Netherlands
100. Nash Family Department of Neuroscience & Friedman Brain Institute, Icahn School of Medicine at Mount Sinai, New York, USA
101. Ronald M. Loeb Center for Alzheimer’s disease, Icahn School of Medicine at Mount Sinai, New York, USA
102. Department of Genetics and Genomic Sciences & Icahn Institute for Data Science and Genomic Technology, Icahn School of Medicine at Mount Sinai, New York, USA
103. Estelle and Daniel Maggin Department of Neurology, Icahn School of Medicine at Mount Sinai, New York, USA

104. Department of Psychiatry, Icahn School of Medicine at Mount Sinai, New York, USA
105. University Bordeaux, Inserm, Bordeaux Population Health Research Center, France
106. Department of Psychiatry, Psychosomatics and Psychotherapy, Center of Mental Health, University Hospital, Wuerzburg, Germany
107. UKDRI@ Cardiff, School of Medicine, Cardiff University, Cardiff, UK
108. Department of Neurology, Boston University School of Medicine, Boston, MA, USA
109. Munich Cluster for Systems Neurology (SyNergy), Munich, Germany.
110. Technical University of Munich, School of Medicine, Klinikum rechts der Isar, Department of Psychiatry and Psychotherapy, Munich, Germany
111. Institute of Cognitive Neurology and Dementia Research (IKND), Otto-Von-Guericke University, Magdeburg, Germany
112. German Center for Neurodegenerative Diseases (DZNE), Magdeburg, Germany.
113. Icelandic Heart Association, Kopovagur, Iceland
114. Center for Neurosciences, Vrije Universiteit Brussel (VUB), Brussels, Belgium
115. Reference Center for Biological Markers of Dementia (BIODEM), Institute Born-Bunge, University of Antwerp, Antwerp, Belgium
116. Institute Born-Bunge, University of Antwerp, Antwerp, Belgium
117. Department of Neurology, UZ Brussel, Brussels, Belgium
118. Department of Medical and Molecular Genetics, Indiana University, Indianapolis, Indiana, USA
119. Fondazione IRCCS, Istituto Neurologico Carlo Besta, Milan Italy
120. Department of Clinical Biochemistry, Herlev and Gentofte Hospital, Herlev, Denmark
121. Sanders-Brown Center on Aging, Department of Biostatistics, University of Kentucky, Lexington, Kentucky, USA
122. Centre for Memory Disturbances, Lab of Clinical Neurochemistry, Section of Neurology, University of Perugia, Italy
123. University of Milan, Milan, Italy
124. Laboratory of Neurogenetics, Department of Internal Medicine, Texas Tech University Health Science Center, Lubbock, Texas, USA
125. Reta Lila Weston Research Laboratories, Department of Molecular Neuroscience, UCL Institute of Neurology, London, UK.
126. Faculty of Medicine, University of Lisbon, Portugal
127. Department of Psychiatry, Social Medicine Center East- Donauspital, Vienna, Austria
128. Institute of Clinical Medicine, University of Oslo, Oslo, Norway
129. Glenn Biggs Institute for Alzheimer’s & Neurodegenerative Diseases, University of Texas Health Sciences Center, San Antonio, TX, USA
130. Dementia Research Centre, UCL Queen Square Institute of Neurology, London, UK
131. Unidad de Demencias, Servicio de Neurología y Neurofisiología. Instituto de Biomedicina de Sevilla (IBiS), Hospital Universitario Virgen del Rocío/CSIC/Universidad de Sevilla, Seville, Spain
132. Instituto de Investigacion Sanitaria ‘Hospital la Paz’ (IdIPaz), Madrid, Spain
133. Centro de Biología Molecular Severo Ochoa (UAM-CSIC), Madrid, Spain
134. Hospital Universitario la Paz, Madrid, Spain
135. Department of geriatric Psychiatry, Central Institute for Mental Health, Mannheim, University of Heidelberg, Germany
136. Dept. of Genetics and Genomic Sciences, Ronald M. Loeb Center for Alzheimer's disease Icahn School of Medicine at Mount Sinai, New York, NY, USA
137. Alzheimer Research Center & Memory Clinic, Andalusian Institute for Neuroscience, Málaga, Spain.
138. Hospital Universitario Ramon y Cajal, IRYCIS, Madrid, Spain
139. Department of Psychiatry and Psychotherapy, Medical University of Vienna, Vienna, Austria
140. Department of Biostatistics, Epidemiology, and Informatics Perelman School of Medicine, University of Pennsylvania, Philadelphia, PA, USA
141. CAEBI, Centro Andaluz de Estudios Bioinformáticos, Sevilla, Spain.
142. Karolinska Institutet, Center for Alzheimer Research, Department NVS, Division of Neurogeriatrics, Stockholm, Sweden
143. Unit for Hereditary dementias, Karolinska University Hospital-Solna, Stockholm, Sweden
144. Aging Research Center, Department of Neurobiology, Care Sciences and Society, Karolinska Institutet and Stockholm University, Stockholm, Sweden
145. Institute of Public Health, University of Cambridge, UK
146. Department of Child and Adolescent Psychiatry and Psychotherapy, University Hospital of Psychiatry Zurich, University of Zurich, Zurich, Switzerland
147. Neuroscience Center Zurich, University of Zurich and ETH Zurich, Switzerland
148. Zurich Center for Integrative Human Physiology, University of Zurich, Switzerland
149. Icelandic Heart Association, University of Iceland, Faculty of Medicine, Reykjavik, Iceland
150. Human Genetics, School of Life Sciences, Life Sciences Building, University Park, University of Nottingham, Nottingham, UK
151. A.I Virtanen Institute for Molecular Sciences, University of Eastern Finland, Kuopio, Finland
152. Department of Neurology, Medical School, University of Cyprus, Cyprus
153. The John P. Hussman Institute for Human Genomics, University of Miami, Miami, Florida,
154. Sorbonne University, GRC n° 21, Alzheimer Precision Medicine Initiative (APMI), AP-HP, Pitié-Salpêtrière Hospital, Paris, France
155. Institute of Human Genetics, University of Bonn, School of Medicine & University Hospital Bonn, Bonn, Germany
156. Institute of Clinical Medicine - Neurology, University of Eastern, Kuopio, Finland

157. Institute of Clinical Medicine – Internal Medicine, University of Eastern Finland, Kuopio, Finland
158. Clinical and Experimental Science, Faculty of Medicine, University of Southampton, Southampton, UK
159. Department of Neurology, UMC Utrecht Brain Center, Utrecht, the Netherlands
160. Biostatistics, University of Kentucky College of Public Health, Lexington, Kentucky, USA
161. Department of Biology, Brigham Young University, Provo, Utah,
162. Translational Health Sciences, Bristol Medical School, University of Bristol, Bristol, UK
163. Division of Clinical Geriatrics, Center for Alzheimer Research, Care Sciences and Society (NVS), Karolinska Institutet, Stockholm, Sweden
164. Institute of Public Health and Clinical Nutrition, University of Eastern Finland, Kuopio, Finland
165. Neuroepidemiology and Ageing Research Unit, School of Public Health, Imperial College London, London, United Kingdom
166. Stockholms Sjukhem, Research & Development Unit, Stockholm, Sweden
167. Department of Neurology, Kuopio University Hospital, Kuopio, Finland
168. Department of Neurosciences, University of Helsinki and Department of Geriatrics, Helsinki University Hospital, Helsinki, Finland
169. Department of Psychiatry and Psychotherapy, Universitätsklinikum Erlangen, and Friedrich-Alexander Universität Erlangen-Nürnberg, Erlangen, Germany.
170. Laboratory of Cognitive Neuroscience, School of Psychology, Aristotle University of Thessaloniki, Thessaloniki, Greece
171. Department of Epidemiology, University of Washington, Seattle, Washington, USA
172. Department of Pathology and Laboratory Medicine University of Pennsylvania, Philadelphia, PA, USA
173. Neurology Service, Marqués de Valdecilla University Hospital (University of Cantabria and IDIVAL), Santander, Spain.
174. Stockholm Gerontology Research Center, Stockholm, Sweden
175. Laboratory of Epidemiology, Demography, and Biometry, National Institute of Aging, The National Institutes of Health, Bethesda, MD, USA
176. Intramural Research Program/National Institute on Aging/National Institutes of Health, Bethesda, MD, USA
177. Memory Clinic, Department of Neurology, Charles University, 2nd Faculty of Medicine and Motol University Hospital, Czech Republic
178. International Clinical Research Center, St. Anne's University Hospital Brno, Brno, Czech Republic
179. Departments of Neurology and Epidemiology, University of Washington, Seattle, Washington, USA
180. Department of Neurology. Hospital Universitario Donostia. OSAKIDETZA-Servicio Vasco de Salud, San Sebastian, Spain
181. Taub Institute Columbia University, New York, New York, USA
182. Department of Neurology, Columbia University, New York, New York, USA
183. School of Health Sciences, Bangor University, UK
184. Unit of Neurology, University of Parma and AOU, Parma, Italy
185. Institute of Neurology, Catholic University of the Sacred Heart, Rome, Itlay
186. Gertrude H. Sergievsky Center, Columbia University, New York, New York, USA
187. MRC Prion Unit at UCL, UCL Institute of Prion Diseases, London, UK
188. Clinic of Neurology, UH "Alexandrovska", Medical University - Sofia, Sofia, Bulgaria
189. Unidad de Trastornos del Movimiento, Servicio de Neurología y Neurofisiología. Instituto de Biomedicina de Sevilla (IBiS), Hospital Universitario Virgen del Rocío/CSIC/Universidad de Sevilla, Seville, Spain
190. Institute for Urban Public Health, University Hospital of University Duisburg-Essen, Essen, Germany
191. Department of Neurology, ErasmusMC, Rotterdam, The Netherlands
192. Neurological Tissue Bank of the Biobanc-Hospital Clinic-IDIBAPS, Institut d'Investigacions Biomèdiques August Pi i Sunyer, Barcelona, Spain.
193. Alzheimer's disease and other cognitive disorders Unit. Neurology Department, Hospital Clinic, Barcelona, Spain
194. Laboratory of Brain Aging and Neurodegeneration- FIL-CONICET, Buenos Aires, Argentina
195. Human Genetics, School of Life Sciences, University of Nottingham, UK
196. Memory Impairment and Neurodegenerative Dementia (MIND) Center, University of Mississippi Medical Center, Jackson, MS, USA
197. Laboratorio de Bioquímica Molecular, Facultad de Medicina, Instituto de Investigaciones Médicas A. Lanari, UBA, C.A.B.A, Buenos Aires, Argentina.
198. Department of Medicine, University of Washington, Seattle, Washington, USA
199. IRCCS Fondazione Don Carlo Gnocchi, Florence, Italy
200. Public Health Promotion Unit, Finnish Institute for Health and Welfare, Helsinki, Finland
201. Department of Clinical Biochemistry, Herlev and Gentofte Hospital, Herlev, Denmark
202. Department of Clinical Medicine, University of Copenhagen, Copenhagen, Denmark
203. DIMEC, University of Parma, Parma, Italy
204. Univ. Lille, Inserm, CHU Lille, UMR1172, Resources and Research Memory Center (MRRC) of Distalz, Licend, Lille France
205. Institut de Biomedicina de València-CSIC CIBERNED, valència, Spain
206. Unitat Mixta de de Neurología y Genética, Institut d'Investigació Sanitària La Fe, valència, Spain
207. Univ. Lille, CNRS, Inserm, CHU Lille, Institut Pasteur de Lille, US 41-UMS 2014-PLBS, bilille, Lille, France
208. Institute of Psychiatry and Psychotherapy, Charité-Universitätsmedizin Berlin, Corporate Member of Freie Universität Berlin, Humboldt-Universität Zu Berlin, and Berlin Institute of Health, Berlin, Germany
209. German Center for Neurodegenerative Diseases (DZNE), Berlin, Germany.
210. Department of Biomedical Sciences, University of Cagliari, Italy

211. CHUV, Old Age Psychiatry, Department of Psychiatry, Lausanne, Switzerland
212. Old Age Psychiatry, Department of Psychiatry, Lausanne University Hospital, Lausanne, Switzerland
213. Department of Geriatric Psychiatry, University Hospital of Psychiatry Zürich, Zürich, Switzerland
214. Department of Neuropsychiatry and Laboratory of Molecular Psychiatry, Charité, Charitéplatz 1, 10117 Berlin, Germany
215. Department of Clinical Biochemistry, Rigshospitalet, Copenhagen, Denmark
216. Maastricht University, Department of Psychiatry & Neuropsychologie, Alzheimer Center Limburg, Maastricht, the Netherlands
217. Depatamento de Especialidades Quirúrgicas, Bioquímica e Inmunología. Facultad de Medicina. Universidad de Málaga. Málaga, Spain
218. Delft Bioinformatics Lab, Delft University of Technology, Delft, The Netherlands
219. Bioinformatics, College of Life Sciences, Brigham Young University, Provo, Utah, USA
220. Institute of Social Medicine, Occupational Health and Public Health, University of Leipzig, 04103 Leipzig, Germany
221. Center of Mental Health, Clinic and Policlinic of Psychiatry, Psychosomatics and Psychotherapy, University Hospital of Würzburg, Wuerzburg, Germany
222. Department of Research and Innovation, Helse Fonna, Haugesund Hospital, Haugesund, Norway.
223. The University of Bergen, Institute of Clinical Medicine (K1), Bergen Norway
224. Departamento de Especialidades Quirúrgicas, Bioquímicas e Inmunología, School of Medicine, University of Málaga, Málaga, Spain
225. Department of Neuroscience and Mental Health, AOU Città della Salute e della Scienza di Torino, Torino, Italy
226. Athens Association of Alzheimer's disease and Related Disorders, Athens, Greece
227. Department of Geriatrics, St. Olav's Hospital, Trondheim University Hospital, Norway
228. Department of Immunology, Hospital Universitario Doctor Negrín, Las Palmas de Gran Canaria, Spain
229. Neurology department-Hospital Clínic, IDIBAPS, Universitat de Barcelona, Barcelona, Spain.
230. 1st Department of Neurology, Aiginition Hospital, National and Kapodistrian University of Athens, Medical School, Greece
231. LVR-Hospital Essen, Department of Psychiatry and Psychotherapy, Medical Faculty, University of Duisburg-Essen, Virchowstr. 174, 45147 Essen, Germany
232. Department of Primary Medical Care, University Medical Centre Hamburg-Eppendorf, Hamburg, Germany
233. Institute of Medical Biometry, Informatics and Epidemiology, University Hospital of Bonn, Bonn, Germany
234. Department of Geriatric Medicine, Oslo University Hospital, Oslo, Norway
235. Laboratory for Advanced Hematological Diagnostics, Department of Hematology and Stem Cell Transplant, Lecce, Italy
236. Centro de Investigación Biomédica en Red de Diabetes y Enfermedades Metabólicas Asociadas, CIBERDEM, Spain, Hospital Clínico San Carlos, Madrid, Spain
237. Department of Neurology, University of Bonn, Bonn, Germany
238. Department of Biomedical Sciences, Section of Neuroscience and Clinical Pharmacology, University of Cagliari, Italy
239. Department of Psychiatry, Namsos Hospital, Namsos, Norway
240. Department of Internal medicine and Biostatistics, ErasmusMC, Rotterdam, The Netherlands
241. Neurodegenerative Brain Diseases Group, VIB Center for Molecular Neurology, VIB, Antwerp, Belgium
242. Department of Neurology, ErasmusMC, Rotterdam, The Netherlands
243. Laboratory for Cognitive Neurology, Department of Neurosciences, University of Leuven, Belgium
244. Neurology Department, University Hospitals Leuven, Leuven, Belgium
245. Department of Psychiatry and Psychotherapy, University Medical Center Goettingen, Goettingen, Germany
246. Department of Psychiatry, Harvard Medical School, McLean Hospital, Belmont, MA, USA
247. Normandie Univ, UNIROUEN, Inserm U1245, CHU Rouen, Department of Neurology and CNR-MAJ, F 76000, Normandy Center for Genomic and Personalized Medicine, Rouen, France
248. German Center for Neurodegenerative Diseases (DZNE), Goettingen, Germany
249. Medical Science Department, iBiMED, Aveiro, Portugal
250. Department of Nutrition and Diatetics, Harokopio University, Athens, Greece
251. Department of Medicine (Biomedical Genetics) Boston University School of Medicine, Boston, MA, USA
252. Department of Biostatistics Boston University School of Public Health, Boston, MA, USA
253. Neurosciences Area. Instituto Biodonostia. San Sebastian, Spain
254. See supplementary list of authors
255. Department of Health Service, University of Washington, Seattle, USA
256. Excellence Cluster on Cellular Stress Responses in Aging-Associated Diseases (CECAD), University of Cologne, Cologne, Germany.

TABLE OF CONTENTS

1. Sample description 9
1.1. Stage I samples 9
1.2. Stage II samples 18
1.3. Longitudinal studies 26

2. Quality control 27
2.1. EADB 27
2.2. Other datasets 30

3. Imputations 32

4. GRCh37/GRCh38 conversion 33

5. Stage II analyses 33

6. Conditional analyses 35

7. HLA analyses 36

8. PheWAS 36

9. GWAS signal colocalization analyses 37

10. Pathway analyses 38
10.1. Gene sets 38
10.2. Expression enrichment analysis in a mouse single-cell dataset 38

11. Functional interpretation of GWAS results 39
11.1. Gene prioritization methods 39
11.2. Gene prioritization results 45
11.3. Short description of some functions of the prioritized genes and their potential implication in AD 55

12. STRING protein interaction analysis 59

13. Genetic risk score analyses 60
13.1. Description of indices of predictive accuracy 60
13.2. Fixed effect and random effects meta-analysis 61

14. List of URLs 61

15. Supplementary References 62

16. Acknowledgments 75

17. Supplementary Figures 85

1. Sample description

1.1. Stage I samples

<u>The European Alzheimer & Dementia Biobank dataset (EADB)</u>

This consortium groups together 20,464 Alzheimer's disease (AD) cases and 22,244 controls after quality controls from 15 European countries (Belgium, Bulgaria, Czech Republic, Denmark, Finland, France, Germany, Greece, Italy, Portugal, Spain, Sweden, Switzerland, The Netherlands and the UK). These samples were genotyped in three independent centers (France, Germany and the Netherlands) leading to define three nodes: EADB-France, EADB-Germany and EADB-Netherlands. In addition, EADB also included Australian partners.

EADB-France
In the France node, samples were collected from nine countries (39 centers/studies), and after quality controls (QCs), we obtained 13,867 AD cases and 15,310 controls. All these samples were genotyped at the Centre National de Recherche en Génomique Humaine (CNRGH, Evry, France).

Belgium: The participants were part of a large prospective cohort[1] of Belgian AD patients and healthy elderly control individuals. The patients were ascertained at the memory clinic of Middelheim and Hoge Beuken (Hospital Network Antwerp, Belgium) and at the memory clinic of the University Hospitals of Leuven, Belgium. The control individuals were the partners of the patients or volunteers from the Belgian community. The study protocols were approved by the ethics committees of the Antwerp University Hospital and the participating neurological centers at the different hospitals of the BELNEU consortium and by the University of Antwerp.

Czech Republic: The Czech Brain Aging Study (CBAS)[2] is a longitudinal memory-clinic–based study recruiting subjects at risk of dementia (subjects referred for cognitive complaints-SCD, MCI). The CBAS+ study is a cross-sectional study of patients in the early stages of dementia. All subjects signed informed consent and both studies were approved by the local ethics committee.

Denmark: The Copenhagen General Population Study (CGPS) is a prospective study of the Danish general population initiated in 2003 and still recruiting. Individuals were selected randomly based on the national Danish Civil Registration System to reflect the adult Danish population aged 20-100. Data were obtained from a self-administered questionnaire reviewed together with an investigator at the day of attendance, a physical examination, and from blood samples including DNA extraction.

Finland: *The ADGEN cohort*[3]: a clinic-based collection of AD patients from Eastern and Northern Finland examined in the Department of Neurology in Kuopio University Hospital and the Department of Neurology in Oulu University Hospital. All the patients were diagnosed with probable AD according to the criteria of the National Institute of Neurological and Communicative Disorders and Stroke and the Alzheimer's disease and Related Disorders Association (NINCDS–ADRDA). The study was approved by the ethics committee of Kuopio University Hospital, Finland (420/2016). *The FINGER study*[4]: a Finnish multi-domain lifestyle RCT enrolling 1,260 older adults with an increased risk of dementia from the general population. The intensive lifestyle intervention lasted for two years, and follow-up extends currently up to seven years. The FINGER study was approved by the coordinating ethics committee of the Hospital District of Helsinki and Uusimaa (94/13/03/00/2009 and HUS/1204/2017), and all the participants gave written informed consent.

France: *The BALTAZAR multicenter (23 memory centers) prospective study*[5]: 1,040 participants from September 2010 to April 2015. They were classified as AD cases (n = 501) according to DSM IV-TR and NINCDS–ADRDA criteria as well as amnestic mild cognitive impairment (MCI) cases (a MCI, n = 417) and non-amnestic MCI cases (na MCI, n = 122)

according to Petersen's criteria. A comprehensive battery of cognitive tests was performed, including MMSE, verbal fluency, and FCSRT. All the participants or their legal guardians gave written informed consent. The study was approved by the Paris ethics committee (CPP Ile de France IV Saint Louis Hospital). *MEMENTO*: a clinic-based study[6] aimed at better understanding the natural history of AD, dementia, and related diseases. Between 2011 and 2014, 2,323 individuals presenting either recently diagnosed MCI or isolated cognitive complaints were enrolled in 26 memory centers in France. This study was performed in accordance with the guidelines of the Declaration of Helsinki. The MEMENTO study protocol has been approved by the local ethics committee (Comité de Protection des Personnes Sud-Ouest et Outre Mer III; approval number 2010-A01394-35). All the participants provided written informed consent. *The CNRMAJ-Rouen study*[7]: early onset AD patients (n = 870). The patients or their legal guardians provided written informed consent. This study was approved by the ethics committee of CPP Ile de France II.

Italy: The AD cases and controls were collated through Italy in different centers: Brescia, Cagliari, Florence, Milan, Rome, Pertugia, San Giovani Rotondo and Torino. AD cases were diagnosed according to DSM III-R,IV and NINCDS–ADRDA criteria. Controls were defined a minima as subjects without DMS-III-R dementia criteria and with integrity of their cognitive functions (MMS>25).

Spain: The Dementia Genetic Spanish Consortium (DEGESCO) is a national consortium comprising 23 research centers and hospitals across the country, that holds the institutional coverage of The Network Center for Biomedical Research in Neurodegenerative Diseases (CIBERNED). Created in 2013, DEGESCO's objective is the promotion and conduction of genetic studies aimed at understanding the genetic architecture of neurodegenerative dementias in the Spanish population and participates in coordinated actions in national and international frameworks. All DNA samples are in compliance with the Law of Biomedical Research (Law 14/2007) and the Royal Decree on Biobanks (RD 1716/2011). Patients included in the present study met clinical criteria for probable or possible disease established by the National Institute of Neurological and Communication Disorders and Stroke and the Alzheimer Disease and Related Disorders Association (NINCDS-ADRDA). Cognitively healthy controls were unrelated individuals who had a documented MMSE in the normal range. Contributing centers in the France node genotyping were Centro de Biología Molecular Severo Ochoa (CSIC-UAM (Madrid), the Institute Biodonostia, University of Basque Contry (EHU-UPV, San Sebastián), Institut de Biomedicina de Valencia CSIC (València), and Sant Pau Biomedical Research Institute (Barcelona).

Sweden: *Upsala.* The Swedish AD patients were ascertained at the Memory Disorder Unit at Uppsala University Hospital. For all patients, the diagnosis was established according to the National Institute on Neurological Disorders and Stroke, and the Alzheimer's Disease and Related Disorders Association (NINDS-ADRDA) guidelines[8]. Healthy control subjects were recruited from the same geographic region following advertisements in local newspapers and displayed no signs of dementia upon Mini Mental State Examination (MMSE). *Swedish National Study on Aging and Care in Kungsholmen (SNAC-K)* data was collected. The original SNAC-K population consisted of 4590 living and eligible persons who lived on the island of Kungsholmen in Central Stockholm, belonged to pre-specified age strata, and were randomly selected to take part in the study. Between 2001 and 2004, 3363 persons participated in the baseline assessment. They belonged to the age cohorts 60, 66, 72, 78, 81, 84, 87, 90, 93, and 96 years and 99 years and older. The examination consists of three parts: a nurse interview, a medical examination, and a neuropsychological testing session. Altogether, the examination takes about six hours. The participants are reexamined each time they reach the next age cohort. All parts of the SNAC-K project have been approved by the ethical committee at Karolinska Institutet or the regional ethical review board. Informed consent was collected from all the participants or, if the person was severely cognitively impaired, from their next of kin.

The UK: *MRC*. The sample set comprises individuals with AD and healthy controls recruited across the MRC Centre for Neuropsychiatric Genetics and Genomics, Cardiff

University, Cardiff, UK; Institute of Psychiatry, London, UK; University of Cambridge, Cambridge, UK. The collection of the samples was through multiple channels, including specialist NHS services and clinics, research registers and Join Dementia Research (JDR) platform. The participants were assessed at home or in research clinics along with an informant, usually a spouse, family member or close friend, who provided information about and on behalf of the individual with dementia. Established measures were used to ascertain the disease severity: Bristol activities of daily living (BADL), Clinical Dementia Rating scale (CDR), Neuropsychiatric Inventory (NPI) and Global Deterioration Scale (GIDS). Individuals with dementia completed the Addenbrooke's Cognitive Examination (ACE-r), Geriatric Depression Scale (GeDS) and National Adult Reading Test (NART) too. Control participants were recruited from GP surgeries and by means of self-referral (including existing studies and Joint Dementia Research platform). For all other recruitment, all AD cases met criteria for either probable (NINCDS-ADRDA, DSM-IV) or definite (CERAD) AD. All elderly controls were screened for dementia using the Mini Mental State Examination (MMSE) or ADAS-cog, were determined to be free from dementia at neuropathological examination or had a Braak score of 2.5 or lower. Control samples were chosen to match case samples for age, gender, ethnicity and country of origin. Informed consent was obtained for all study participants, and the relevant independent ethical committees approved study protocols. *SOTON, University of Southampton, Southampton, UK*. All AD cases met criteria for either probable (NINCDS-ADRDA, DSM-IV) or definite (CERAD) AD. All elderly controls were screened for dementia using the MMSE or ADAS-cog, were determined to be free from dementia at neuropathological examination or had a Braak score of 2.5 or lower. *Nottingham and Manchester*, *University of Nottingham, Nottingham, UK and Manchester Brain Bank*. All AD cases met criteria for either probable (NINCDS-ADRDA, DSM-IV) or definite (CERAD) AD. All elderly controls were screened for dementia using the MMSE or ADAS-cog, were determined to be free from dementia at neuropathological examination or had a Braak score of 2.5 or lower. *KCL, London Neurodegenerative Diseases Brain Bank*. All AD cases met criteria for either probable (NINCDS-ADRDA, DSM-IV) or definite (CERAD) AD. All elderly controls were screened for dementia using the MMSE or ADAS-cog, were determined to be free from dementia at neuropathological examination or had a Braak score of 2.5 or lower. *PRION,* All AD cases met criteria for either probable (NINCDS-ADRDA, DSM-IV) or definite (CERAD) AD. All elderly controls were screened for dementia using the MMSE or ADAS-cog, were determined to be free from dementia at neuropathological examination or had a Braak score of 2.5 or lower. *CFAS Wales*, The Cognitive Function and Ageing Study Wales (CFAS-Wales) is a longitudinal population-based study of people aged 65 years and over in rural and urban areas of Wales that aims to investigate physical and cognitive health in older age and examine the interactions between health, social networks, activity, and participation. Individuals aged 65 years and over were randomly sampled from general medical practice lists between 2011 and 2013, stratified by age to ensure equal numbers in two age groups, 65-74 years and 75 and over. The baseline sample included 3593 older people and included those living in care homes as well as those living at home. Those who provided written consent to join the study were interviewed in their own homes by trained interviewers and could choose to have the interview conducted through the medium of either English or Welsh. Participants were followed up 2 years later. All AD cases met criteria for either probable (NINCDS-ADRDA, DSM-IV) or definite (CERAD) AD. All elderly controls were screened for dementia using the MMSE or CAMCOG, and were determined to be free from dementia. *UCL-DRC*. the UCL Alzheimer's disease cohort of the Dementia Research Centre (UCL - EOAD DRC) included patients seen at the Cognitive Disorders Clinics at The National Hospital for Neurology and Neurosurgery (Queen Square), or affiliated hospitals. Individuals were assessed clinically and diagnosed as having probable Alzheimer's disease based on contemporary clinical criteria in use at the time, including imaging and neuropsychological testing where appropriate.

EADB-Germany

In the German node, samples were collected from seven countries (11 centers/studies) and after QCs, we obtained 4,159 AD cases and 4,545 controls. All these samples were genotyped at Life&brain (Bonn, Germany).

Germany: DELCODE (the multicenter DZNE-Longitudinal Cognitive Impairment and Dementia Study). This is an observational longitudinal memory clinic-based multicenter study in Germany comprising 400 subjects with Subjective cognitive decline (SCD), 200 mild cognitive impairment (MCI) patients, 100 AD dementia patients, 200 control subjects without subjective or objective cognitive decline, and 100 first-degree relatives of patients with a documented diagnosis of AD dementia. All patient groups (SCD, MCI, AD) are referrals, including self-referrals, to the participating memory centers. The control group and the relatives of AD dementia patients are recruited by standardized public advertisement. Ten university-based memory centers are participating, all being collaborators of local DZNE sites. All patient groups (SCD, MCI, AD) were assessed clinically at the respective memory centers before entering DELCODE. The assessments include medical history, psychiatric and neurological examination, neuropsychological testing, blood laboratory work-up, cerebrospinal fluid (CSF) biomarkers, and routine MRI, all according to the local standards. The Consortium to Establish a Registry for Alzheimer's Disease (CERAD) neuropsychological test battery was applied at all memory centers to measure cognitive function. German age, sex, and education-adjusted norms of the CERAD neuropsychological battery are available online (www.memoryclinic.ch). Detail description is of recruitment protocol is reported elsewhere. *The VOGEL study*: The VOGEL study is a prospective, observational, long-term follow-up study with three time points of investigation within 6–8 years. This cohort includes dementia and healthy subjects. Residents of the city of Würzburg born between 1936 and 1941 were recruited. Every participant underwent physical, psychiatric, and laboratory examinations and performed intense neuropsychological testing as well as VSEP and NIRS according to the published procedures. A total of 604 subjects were included. *The Heidelberg/Mannheim memory clinic sample:* This cohort includes 61 subjects from whom 40 MCI patients were recruited and assessed between 2012 and 2016. Some of those patients converted to dementia by AD or other dementias. *The PAGES study:* This study includes 301 subjects. AD patients were recruited at the memory clinic of the Department of Psychiatry, University of Munich, Germany. Participants in whom dementia associated with AD was diagnosed fulfilled the criteria for probable AD according to the NINCDS–ADRDA. The control group included participants who were randomly selected from the general population of Munich. Controls who had central nervous system diseases or psychotic disorders or who had first-degree relatives with psychotic disorders were excluded. *The Technische Universität München study:* This cohort includes 359 healthy, AD, and other dementias patients recruited from the Centre for Cognitive Disorders. All the participants provided written informed consent. A biobank was submitted to the ethics committee of the Technical University of Munich, School of Medicine (Munich, Germany), which raised no objections and approved the biobank (reference number 347-14). *The Göttingen Universität study:* This study includes 111 in- and outpatients with a healthy or AD dementia status from the Department of Psychiatry of the University of Göttingen. The study's ethical statement was provided locally at the Göttingen University Medical Centre. *The German Dementia Competence Network (DCN) cohort:* Individuals from the DCN cohort were recruited from 14 university hospital memory clinics across Germany between 2003 and 2005[9]. The study was approved by the respective ethics committees, and written informed consent was obtained from all the participants prior to inclusion. *The German Study on Aging, Cognition, and Dementia (AgeCoDe):* The AgeCoDe study is a general practice (GP) registry-based longitudinal study in elderly individuals that recruited patients aged 75 years and above in six German cities from 2003 to 2004[10]. The study was approved by the respective ethics committees, and written informed consent was obtained from all the participants prior to inclusion.

Greece: *the HELIAD study*, comprising 49 AD cases and 1,150 controls. HELIAD is a population-based, multidisciplinary, collaborative study designed to estimate, in the Greek

population over the age of 64 years, the prevalence and incidence of MCI, AD, other forms of dementia, and other neuropsychiatric conditions of aging and to investigate associations between nutrition and cognitive dysfunction or age-related neuropsychiatric diseases. The participants were selected through random sampling from the records of two Greek municipalities, Larissa and Marousi. All the participants signed informed consent in Greek.

Portugal: *the Lisbon study* from Portugal, totaling 78 AD cases and 74 controls. This cohort was recruited in 2008–2009 to investigate the connections between oxidative stress and lipid dyshomeostasis in AD. The project includes 190 subjects and was approved by the local ethics committee, and all the participants provided written informed consent. This study includes healthy and dementia-by-AD subjects.

Spain: Those samples are part of DEGESCO. DEGESCO Centers from whom DNA samples were genotyped in the German node (1,778 cases and 470 controls) were the Alzheimer Research Center and Memory Clinic, Fundació ACE, Institut Català de Neurociències Aplicades (Barcelona), the Neurology Service at University Hospital Marqués de Valdecilla (Santander), the Alzheimer's disease and other cognitive disorders, Neurology Department, at Hospital Clínic, IDIBAPS (Barcelona), the Molecular Genetics Laboratory, at the Hospital Universitario Central de Asturias (Oviedo), and Fundació Docència i Recerca Mútua de Terrassa and Movement Disorders Unit, Department of Neurology, University Hospital Mútua de Terrassa (Barcelona).

Switzerland: Two datasets from Switzerland and Austria were combined, totaling 182 AD cases and 388 controls. *The Lausanne study*: This study includes 137 community-dwelling participants aged 55+ years with cognitive impairment (memory clinic patients with MCI, dementia) or normal cognition (recruited by advertisement, word of mouth). The study's ethical statement was provided locally at the Department of Psychiatry, Geneva University Centre, Switzerland. *The VITA study:* This is a longitudinal study of 606 individuals (Vienna, Austria) who were 75 years old in 2000, followed up every 30–90 months. This cohort includes dementia and healthy subjects. All the participants gave written informed consent. The study conformed to the latest version of the Declaration of Helsinki and was approved by the ethics committee of the City of Vienna, Austria

EADB-Netherlands

In the Dutch node, samples were collected from six organizations in the Netherlands and after QCs, we obtained 2,438 AD cases and 2,389 controls. All these samples were genotyped at the Erasmus Medical University (Rotterdam, The Netherlands). The Medical Ethics Committee (METC) of the local institutes approved the studies. All the participants and/or their legal guardians gave written informed consent for participation in the clinical and genetic studies. Samples from the following institutes were included. 1) *Erasmus Medical Center:* most individuals were selected from population studies from the epidemiology department and accounted for most of the controls, while a smaller subset of samples originated from the neurology department, where AD was diagnosed according to the National Institute of Neurological and Communicative Disorders and Stroke-Alzheimer's Disease and Related Disorders Association (NINCDS-ADRDA) criteria for AD[11]. 2) *The Amsterdam Dementia Cohort (ADC)*[12]: This cohort comprises patients who visit the memory clinic of the VU University Medical Centre, the Netherlands. The diagnosis of probable AD is based on the clinical criteria formulated by the NINCDS–ADRDA and based on the NIA–AA. Diagnosis of MCI was made according to Petersen and NIA-AA. Controls presented with subjective cognitive decline at the memory clinic, but performed within normal limits on all clinical investigations. 3) *The 100-Plus study:* This study includes Dutch-speaking individuals who (i) can provide official evidence for being aged 100 years or older, (ii) self-report to be cognitively healthy, which is confirmed by a proxy, (iii) consent to the donation of a blood sample, (iv) consent to (at least) two home visits from a researcher, and (v) consent to undergo an interview and neuropsychological test battery[13]. 4) *Parelsnoer Institute:* a collaboration between 8 Dutch University Medical Centers in which clinical data and biomaterials from patients suffering from chronic diseases (so called "Pearls") are collected according to harmonized protocols. The Pearl Neurodegenerative Diseases[14] includes

individuals diagnosed with dementia, mild cognitive impairment, and controls with subjective memory complaints. 5) *The Netherlands Brain Bank:* a non-profit organization that collects human brain tissue of donors with a variety of neurological and psychiatric disorders, but also of non-diseased donors. A clinical diagnosis of AD is based on the clinical criteria of probable AD[8,15]. The selected AD patients for this study all received a definitive diagnosis which was based on autopsy. 6) *Maastricht University Medical Center*: a subset of individuals that were referred to the memory clinic for cognitive complaints were included if they participated in the BioBank-Alzheimer Centrum Limburg (BB-ACL)[16]. Diagnosis of MCI was made according to the criteria of Petersen, and diagnosis of AD-type dementia was made according to the criteria of the DSM-4[17], and the NINCDS-ADRDA[8].

EADB-Australia

The Sydney MAS study: a longitudinal study investigating MCI, related syndromes, and age-related cognitive change. Older adults (70–90 years old) were randomly recruited from the community in Sydney, Australia (n = 1,037). An extensive interview was undertaken and questionnaire data collected, including demographics, cognitive performance, and medical history. The majority of participants provided blood samples for genetic analysis. Neuroimaging was performed on a subset of participants. Ethics approval for the study was provided by the ethics committee of the University of New South Wales and the Illawarra Area Health Service Human Research Ethics Committee. All the participants provided written informed consent to join the study. More information is provided in Sachdev et al[18]. In our study, there were 43 AD cases and 215 controls. Due to the low sample size, the study was not considered in the meta-analysis. However, samples from Sydney MAS study were included in the evaluation of the association of the polygenic risk score with conversion to all-dementia and AD dementia.

GR@ACE

The GR@ACE study[19] recruited Alzheimer's disease (AD) patients from Fundació ACE, Institut Català de Neurociències Aplicades (Catalonia, Spain), and control individuals from three centers: Fundació ACE (Barcelona, Spain), Valme University Hospital (Seville, Spain), and the Spanish National DNA Bank–Carlos III (University of Salamanca, Spain) (http://www.bancoadn.org). Additional cases and controls were obtained from dementia cohorts included in the Dementia Genetics Spanish Consortium (DEGESCO)[20]. At all sites, AD diagnosis was established by a multidisciplinary working group—including neurologists, neuropsychologists, and social workers—according to the DSM-IV criteria for dementia and the National Institute on Aging and Alzheimer's Association's (NIA–AA) 2011 guidelines for diagnosing AD. In our study, we considered as AD cases any individuals with dementia diagnosed with probable or possible AD at any point in their clinical course.
Genotyping was conducted using the Axiom 815K Spanish biobank array (Thermo Fisher) at the Spanish National Centre for Genotyping (CeGEN, Santiago de Compostela, Spain). The genotyping array not only is an adaptation of the Axiom biobank genotyping array but also contains rare population-specific variations observed in the Spanish population.

The Rotterdam Study

The Rotterdam Study is a prospective population-based middle-aged and elderly cohort that started in 1990 in the district of Ommoord, in Rotterdam, The Netherlands. The study includes 14,926 participants and has three subcohorts[21]. At start of the study, all inhabitants of the district of Ommoord who were aged 55 years and older were invited to participate. At baseline, in 1990-1993, of the 10,215 invited inhabitants, 7,983 agreed to participate in the baseline examination (response rate 78%). In 2000, the cohort was extended with 3,011 participants (67% of invitees). This extension consisted of all persons living in the study district who had become 55 years and older or had moved into the study district. A second extension was initiated in 2006, in which 3,932 participants (65% of invitees) who were 45 years and older were included. Study rounds consist of a home interview and visits with extensive investigations at the dedicated research centre. Rounds are repeated every 4-6

years. Participants are continuously monitored for diseases and mortality through linkage of the medical records from the general practitioners and municipality records. The Rotterdam Study has been approved by the Medical Ethics Committee of the Erasmus MC and by the Ministry of Health, Welfare and Sport of The Netherlands. All participants provided written informed consent to participate in the study and to obtain information from their treating physicians.

A total of 11,496 participants from the three subcohorts who were genotyped passed genotyping quality control (92% of all subjects with genotyping)[22]. Exclusion criteria were a call rate <98%, Hardy–Weinberg p-value <10−6, minor allele frequency <0.01%, excess autosomal heterozygosity >0.336, sex mismatch, and outlying identity-by-state clustering estimates. Imputations were performed using the Haplotype Reference Consortium (HRC) panel[23].

Dementia ascertainment involved cognitive screening at the study research centre. We further assessed individuals with a Mini-mental state examination (MMSE) score <26 or Geriatric Mental State Schedule organic level >0[24], by administering the Cambridge Mental Disorders of the Elderly Examination by a research physician. We also interviewed spouses or informants. A consensus panel headed by a consultant neurologist established the final diagnosis according to standard criteria. We studied the outcomes of all-cause dementia (DSM-III-R), and Alzheimer's disease (NINCDS–ADRDA). For the assessment of dementia, and type of dementia, the latest follow-up information with available data was used to determine the disease state. Follow-up for dementia was near complete until 1st January 2016. Within this period, participants were censored at date of dementia diagnosis, death, or loss to follow-up. For this study, we included participants from the first, second and third subcohort (N=11,070).

European Alzheimer's Disease Initiative (EADI) Consortium

EADI is composed of several case-control studies and one population-based cohort, the 3C study[25,26]. Case-control studies are comprised of AD cases and cognitively normal controls across France. The population-based cohort, the 3C study, is a prospective study of the relationship between vascular factors and dementia carried out in the three French cities Bordeaux, Montpellier and Dijon. The AD status was defined based on 12 years follow-up for Dijon participants, 14-15 years follow-up for Montpellier participants and 17-18 years follow-up for Bordeaux participants. All other non demented subjects of 3C were included as controls. All AD cases, both in the case-control studies and the 3C study, were ascertained by neurologists and the clinical diagnosis of probable AD was established according to the DSM-III-R and NINCDS-ADRDA criteria. Samples that passed DNA quality control were genotyped with Illumina Human 610-Quad BeadChips.

Genetic and Environmental Risk in AD (GERAD) Consortium/Defining Genetic, Polygenic, and Environmental Risk for Alzheimer's Disease (PERADES) Consortium

The GERAD/PERADES sample comprises 3,177 Alzheimer's disease cases and 7,277 controls with available age and gender data[27]. Cases and elderly screened controls were recruited by the Medical Research Council (MRC) Genetic Resource for Alzheimer's disease (Cardiff University; Institute of Psychiatry, London; Cambridge University; Trinity College Dublin), the Alzheimer's Research Trust (ART) Collaboration (University of Nottingham; University of Manchester; University of Southampton; University of Bristol; Queen's University Belfast; the Oxford Project to Investigate Memory and Ageing (OPTIMA), Oxford University); Washington University, St Louis, United States; MRC PRION Unit, University College London; London and the South East Region Alzheimer's disease project (LASER-AD), University College London; Competence Network of Dementia (CND) and Department of Psychiatry, University of Bonn, Germany; the National Institute of Mental Health (NIMH) Alzheimer's disease Genetics Initiative. 6129 population controls were drawn from large existing cohorts with available GWAS data, including the 1958 British Birth Cohort (1958BC) (http://www.b58cgene.sgul.ac.uk), the KORA F4 Study and the Heinz Nixdorf Recall Study. All Alzheimer's disease cases met criteria for either probable (NINCDS-ADRDA, DSM-IV) or

definite (CERAD) Alzheimer's disease. All elderly controls were screened for dementia using the MMSE or ADAS-cog, were determined to be free from dementia at neuropathological examination or had a Braak score of 2.5 or lower. Genotypes from all cases and 4617 controls were previously included in the AD GWAS by Harold and colleagues[27]. Genotypes for the remaining 2660 population controls were obtained from WTCCC2.

The Norwegian DemGene Network
This is a Norwegian network of clinical sites collecting cases from memory clinics based on a standardized examination of cognitive, functional, and behavioral measures and data on the progression of most patients. The Norwegian DemGene Network includes 2,224 cases and 3,089 healthy controls from different studies described elsewhere[28]. The cases were diagnosed according to recommendations from the NIA–AA, the NINCDS–ADRDA criteria, or the ICD-10 research criteria. The controls were screened with a standardized interview and cognitive tests. Additional controls from blood donors of the Oslo University Hospital, Ulleval Hospital, were included (*n*=4992, age between 18-65 years, 48% female). They were thoroughly screened for diseases and medication, and provided blood for DNA analysis, in line with approval from the Regional Committee for Medical and Health Research Ethics. Individuals from the DemGene study and blood donors were genotyped using either the Human Omni Express-24 v1.1 chip (Illumina Inc., San Diego, CA) or the DeCodeGenetics_V1_20012591_A1 chip at deCODE Genetics (Reykjavik, Iceland).

The Neocodex–Murcia study (NxC)
This study includes 324 sporadic AD patients and 754 controls of unknown cognitive status from the Spanish general population collected by Neocodex[29,30]. AD patients were diagnosed as having possible or probable AD in accordance with the NINCDS–ADRDA criteria.

The Copenhagen City Heart Study (CCHS)
CCHS is a prospective study of the Danish general population initiated in 1976-78 with follow-up examinations in 1981-83, 1991-94, 2001-03, and 2011-13. Individuals were selected randomly based on the national Danish Civil Registration System to reflect the adult Danish population aged 20-100. Data were obtained from a self-administered questionnaire reviewed together with an investigator at the day of attendance, a physical examination, and from blood samples including DNA extraction. Genotypes were available on 8,118 individuals from the 1991-94 and 2001-03 examinations following genotyping on the Illumina Metabochip and/or the Illumina HumanExome.

Bonn studies
DietBB: The DietBB sample included in this GWAS is a subsample extracted from the AgeCoDe cohort in the context of an ongoing genome-wide methylation analysis for dementia. In addition to methylation, the DietBB samples has genome-wide genotype data which was included in this study. The German study on aging, cognition and dementia (AgeCoDe)[10,31] study is a general practice (GP) registry-based longitudinal study in elderly individuals on the identification of predictors of dementia. Participants were recruited in six German cities (Bonn, Dusseldorf, Hamburg, Leipzig, Mannheim, and Munich) with a total of 138 GPs connected to the study sites. The inclusion criteria for this study were an age of 75 years and older, absence of dementia according to GP judgment, and at least one contact with the GP within the past 12 months. Exclusion criteria were GP consultations by home visits only, living in a nursing home, severe illness with an anticipated fatal outcome within 3 months, language barrier, deafness or blindness, and lack of ability to provide informed consent. Baseline recruitment was performed in 2002 and 2003. The study was approved by the local ethical committees of the Universities of Bonn, Hamburg, Dusseldorf, Heidelberg/Mannheim, and Leipzig, and the Technical University of Munich. A total of 3327 subjects provided informed consent for participation after being provided with a complete description of the study protocol. The study assessments were performed by trained

interviewers at the subjects' home. Seventy individuals were excluded after baseline interview because of the presence of dementia according to standard assessment, and 40 subjects were excluded for age less than 75 years. In AgeCoDe, dementia was diagnosed according to the criteria set of DSM-IV in a consensus conference with the interviewer and an experienced geriatrician or geriatric psychiatrist. The etiological diagnosis of dementia in AD was established according to the National Institute of Neurological and Communicative Diseases and Stroke/Alzheimer's Disease and Related Disorders Association (NINCDS-ADRDA) criteria for probable AD[8]. Mixed dementia was diagnosed in cases of cerebrovascular events without temporal relationship to cognitive decline. Mixed dementia and dementia in AD were combined. Dementia diagnosis in subjects who were not interviewed personally was based on the Global Deterioration Scale[32] (score ≥4 points). In these cases, an etiological diagnosis was established only if the information provided was sufficient to judge etiology according to the criteria just described. For DietBB, cohort participants where included if they were dementia-free at baseline and available biomaterial for DNA analysis is available. This criterion led to the selection of 320 participants. In 120 of these participants, dementia of the AD-type occurred at any follow up. The additional 200 remain free of dementia until last follow up of AgeCoDe.

Bonn OMNI cohort: the Bonn OMNI cohort consists of AD patients and controls derived from a larger German GWAS cohort which was recruited from the following three sources: (i) the German Dementia Competence Network; (ii) the German study on Aging, Cognition, and Dementia in primary care patients (AgeCoDe); and (iii) the interdisciplinary Memory Clinic at the University Hospital of Bonn. The control sample comprised of individuals from the population-based study Heinz Nixdorf Recall (HNR) study cohort. This sample was previously used for replication in Lambert et al.[33]. *The German study on aging, cognition and dementia (AgeCoDe)*: see description above. *The German competence network cohort (DCN)*: The DCN cohort includes 1,095 patients with mild cognitive impairment (MCI) and 648 cases with mild Alzheimer's disease (AD) clinical dementia syndrome that were recruited from 14 university hospital memory clinics across Germany between 2003 and 2005[9]. Exclusion criteria were substance abuse or dependence, insufficient German language skills, multi-morbidity, comorbid condition with excess mortality, circumstances that would have made regular attendance at follow-up visits questionable and lack of an informant. The diagnosis of mild dementia according to ICD-10 criteria required a decline of cognitive ability (at least 1 SD) from a previous level in at least 2 domains as evidenced by age-corrected standardized tests, impairment in activities of daily living (i.e. B-ADL > 6), changes in personality, drive, social behavior or control of emotion but no clouding of consciousness. These changes must have persisted for at least 3 months. The etiological diagnosis of AD was assigned according to NINCDS-ADRDA criteria[8]. *Memory clinic Bonn*: The interdisciplinary Memory Clinic of the Department of Psychiatry and Department of Neurology at the University Hospital in Bonn provided further patients. Diagnoses were assigned according the NINCDS/ADRDA criteria[8] and on the basis of clinical history, physical examination, neuropsychological testing (using the CERAD neuropsychological battery, including the MMSE), laboratory assessments, and brain imaging. *Control sample:* In the Heinz Nixdorf Recall (Risk Factors, Evaluation of Coronary Calcification, and Lifestyle) study, participants were randomly sampled in three cities in Germany. The study design has previously been described[34,35]. Briefly, 4814 participants aged 45 to 75 years were enrolled between 2000 and 2003 (t0, baseline). Cognitive performance of participants was evaluated at follow up scheduled 5 years after baseline (t1, n = 4157, 2005–2008) and then again at follow up 5 years after t1 (t2, n = 3087, 2010–2015). Controls sample was selected if participant did not present cognitive impairment as reported at the last available evaluation. Cognitive evaluation has been described extensively previously[36,37]. Herein, cognitive impairment at t1 was defined as a performance of one standard deviation (SD) below the age- and education-adjusted mean except for the clock-drawing test, where a performance ≥3 was rated as impaired (for a detailed description, see the study by Winkler et al.[36]). The study was approved by the University of

Duisburg-Essen Institutional Review Board and followed established guidelines of good epidemiological practice.

UK Biobank
AD/Dementia cases were extracted from UK Biobank (data release Feb 2020) self-report, ICD10 diagnoses and ICD10 cause of death. Proxy AD/Dementia cases included all participants who reported at least one biological relative (parents and siblings) affected with dementia either at baseline or follow up. Participants who answered “Do not know” or “Prefer not to answer” were excluded from analyses. Individual who did not report dementia or any family history of dementia were used as controls. Our analysis included 2,447 diagnosed cases, 46,828 proxy cases of dementia and 338,440 controls.

1.2. Stage II samples

Alzheimer’s Disease Genetics Consortium (ADGC)
The ADGC dataset comprises subjects from 35 datasets including two waves of the Adult Changes in Thought (ACT) cohort study [ACT1/ACT2]; ten waves of cases and cognitively normal controls from the National Institute on Aging (NIA) Alzheimer Disease Centers (ADCs); the Alzheimer Disease Neuroimaging Initiative (ADNI); the Biomarkers of Cognitive Decline Among Normal Individuals (BIOCARD) Cohort; two waves of the Religious Orders Study/Memory and Aging Project (ROSMAP1-2) and the Chicago Health and Aging Project (CHAP) cohort studies at Rush University; the Einstein Aging Study (EAS); the Multi-Site Collaborative Study for Genotype-Phenotype Associations in Alzheimer’s Disease (GenADA) Study by GlaxoSmithKline; Mayo Clinic Jacksonville (MAYO) and Rochester (RMAYO) case-control datasets; the Multi-Institutional Research in Alzheimer's Genetic Epidemiology (MIRAGE) study; the NIA Late-Onset Alzheimer’s Disease (LOAD) Family Study (NIA-LOAD); the Netherlands Brain Bank (NBB) case-control dataset; the Oregon Health and Science University (OHSU) case-control dataset; the Pfizer case-control dataset; the Texas Alzheimer’s Research and Care Consortium (TARCC) dataset; the Translational Genomics Research Institute series 2 (TGEN2) dataset; the University of Miami (UM)/ Case Western Reserve University (CWRU)/ Mt. Sinai School of Medicine (MSSM) and UM/CWRU/TARCC wave 2 datasets [UM/CWRU/MSSM and UM/CWRU/TARCC2]; the Universitatsklinikum Saarlandes (UKS) case-control dataset; the University of Pittsburgh (UPITT) case-control dataset; Washington University (WASHU) wave 1 and 2 case-control datasets [WASHU1/WASHU2]; and the Washington Heights-Inwood Community Aging Project (WHICAP) study datasets.
Descriptions of the ACT1, ADC waves 1-7, ADNI, BIOCARD, CHAP, EAS, GenADA, MAYO, MIRAGE, NBB, NIA-LOAD, OHSU, PFIZER, RMAYO, ROSMAP1, ROSMAP2, TARCC, TGEN2, UKS, UM/CWRU/MSSM, UM/CWRU/TARCC2, UPITT, WASHU1, WASHU2, and WHICAP cohorts have been provided in previous ADGC and IGAP studies[33,38–42]. Here we update descriptions of these studies, where applicable, and provide descriptions for ACT2, ADC wave 8-10, as well as the Combined Small Datasets Collection (CSDC). The CSDC is a harmonized collection of merged small datasets (comprising the existing datasets of ACT2, BIOCARD, CHAP2, EAS, NBB, RMAYO, ROSMAP2, and WASHU2) used in common variant analyses to deal with small case and control counts within the individual studies. All analyses were restricted to individuals of European ancestry. All subjects were recruited under protocols approved by the appropriate Institutional Review Boards (IRBs).

<u>ACT1/ACT2:</u> The ACT cohort is an urban and suburban elderly population from a stable HMO that includes 2,581 cognitively intact subjects age ≥ 65 who were enrolled between 1994 and 1998[43,44]. An additional 811 subjects were enrolled in 2000-2002 using the same methods except oversampling clinics with more minorities. More recently, a Continuous Enrollment strategy was initiated in which new subjects are contacted, screened, and enrolled to keep 2,000 active at-risk person-years accruing in each calendar year. This resulted in an enrollment of 4,146 participants as of May 2009. All clinical data are reviewed at a consensus conference. Dementia onset is assigned half-way between the prior biennial

and the exam that diagnosed dementia. A waiver of consent was obtained from the IRB to enroll deceased ACT participants. In total, ACT contributed data on 553 individuals with probable or possible Alzheimer's disease (70 with autopsy-confirmation) and on 1,579 cognitively normal elders (CNEs, 155 with autopsy-confirmation) who were included in the analyses, with 2,103 cases/1,571 CNEs in the first wave (ACT1) and 29 cases/8 CNEs in the second wave (ACT2).

NIA ADC Samples (ADC1-10): The NIA ADC cohort included subjects ascertained and evaluated by the clinical and neuropathology cores of the 32 NIA-funded ADCs. Data collection is coordinated by the National Alzheimer's Coordinating Center (NACC). NACC coordinates collection of phenotype data from the 32 ADCs, cleans all data, coordinates implementation of definitions of Alzheimer's disease cases and controls, and coordinates collection of samples. The complete ADC cohort consists of 3,311 autopsy-confirmed and 2,889 clinically-confirmed Alzheimer's disease cases, and 247 cognitively normal elders (CNEs) with complete neuropathology data who were older than 60 years at age of death, and 3,687 living CNEs evaluated using the Uniform dataset (UDS) protocol[45,46] who were documented to not have mild cognitive impairment (MCI) and were between 60 and 100 years of age at assessment. Based on the data collected by NACC, the ADGC Neuropathology Core Leaders Subcommittee derived inclusion and exclusion criteria for Alzheimer's disease and control samples. All autopsied subjects were age ≥ 60 years at death. Based on the data collected by NACC, the ADGC Neuropathology Core Leaders Subcommittee derived inclusion and exclusion criteria for Alzheimer's disease and control samples. All autopsied subjects were age ≥ 60 years at death. Alzheimer's disease cases were demented according to NINCDS-ADRDA/DSMIV-V criteria or Clinical Dementia Rating (CDR) ≥ 137[8,11]. Neuropathologic stratification of cases followed NIA/Reagan criteria explicitly or used a similar approach when NIA/Reagan criteria were coded as not done, missing, or unknown. Cases were intermediate or high likelihood by NIA/Reagan criteria with moderate to frequent amyloid plaques[47] and neurofibrillary tangle (NFT) Braak stage of III-VI[48,49]. Persons with Down's syndrome, non-Alzheimer's disease tauopathies and synucleinopathies were excluded. All autopsied controls had a clinical evaluation within two years of death. Controls did not meet NINCDS-ADRDA/DSMIV-V criteria for dementia, did not have a diagnosis of mild cognitive impairment (MCI), and had a CDR of 0, if performed. Controls did not meet or were low-likelihood Alzheimer's disease by NIA/Reagan criteria, had sparse or no amyloid plaques, and a Braak NFT stage of 0 – II. ADCs sent frozen tissue from autopsied subjects and DNA samples from some autopsied subjects and from living subjects to the ADCs to the National Cell Repository for Alzheimer's Disease (NCRAD). DNA was prepared by NCRAD for genotyping and sent to the genotyping site at Children's Hospital of Philadelphia. ADC samples were genotyped and analyzed in separate batches (waves 1-10). The ADC data used in the analyses (ADC1-10) consist of 6,292 cases and 4,980 CNEs in total.

ADNI: ADNI is a longitudinal, multi-site observational study including Alzheimer's disease, mild cognitive impairment (MCI), and elderly individuals with normal cognition assessing clinical and cognitive measures, MRI and PET scans (FDG and 11C PIB) and blood and CNS biomarkers. For this study, ADNI contributed data on 268 Alzheimer's disease cases with MRI confirmation of Alzheimer's disease diagnosis and 173 healthy controls with Alzheimer's disease-free status confirmed as of most recent follow-up. Alzheimer's disease subjects were between the ages of 55–90, had an MMSE score of 20–26 inclusive, met NINCDS-ADRDA criteria for probable Alzheimer's disease[8,11], and had an MRI consistent with the diagnosis of Alzheimer's disease. Control subjects had MMSE scores between 28 and 30 and a Clinical Dementia Rating of 0 without symptoms of depression, MCI or other dementia and no current use of psychoactive medications. According to the ADNI protocol, subjects were ascertained at regular intervals over 3 years, but for the purpose of our analysis we only used the final ascertainment status to classify case-control status. Additional details of the study design are available elsewhere[41,50,51].

BIOCARD: The BIOCARD study is supported by a grant jointly funded by the National Institute on Aging (NIA) and the National Institute of Mental Health (NIMH). The

overarching goal of the BIOCARD Study is to identify biomarkers associated with progression from normal cognitive status to cognitive impairment or dementia, with a particular focus on Alzheimer's Disease. Please see Albert et al.[52] for a detailed description of the study. A total of 354 individuals were initially enrolled in the study. Recruitment was conducted by the staff of the Geriatric Psychiatry Branch (GPB) of the intramural program of the NIMH, beginning in 1995 and ending in 2005. The domains of information collected as part of the study include: cognitive testing, magnetic resonance imaging (MRI), cerebrospinal fluid (CSF), amyloid imaging (using PET-PiB), and blood specimens. Investigators at the Johns Hopkins University School of Medicine began evaluating participants in 2009, and subjects are seen annually. At each visit there are assessments of medical and cognitive status, as well as acquisition of MRI, CSF, PET-PiB, and blood. Each subject in the analyses received a consensus diagnosis by a team of neurologists, neuropsychologists, research nurses and research assistants of the BIOCARD Clinical Core at Johns Hopkins with diagnoses based on evidence of clinical or cognitive dysfunction (i.e., individuals with a CDR score > 0 and/or evidence of decline on cognitive testing). To the extent possible, this diagnosis did not use the cognitive test scores. In brief, (1) clinical data relating to the medical, neurologic and psychiatric status of the subject were examined, (2) reports of changes in cognition by the subject and other sources were examined, and (3) decline in cognitive performance was established. Cognitive test scores were used to: (1) determine whether the subject had become cognitively impaired, and (2) determine the likely etiology of such impairment. These diagnostic procedures are comparable to those implemented in the Alzheimer's Disease Centers (ADC) program, supported by the NIA. For this study, BIOCARD contributed data on 6 Alzheimer’s disease cases and 112 healthy controls with Alzheimer’s disease-free status confirmed as of most recent follow-up.

CHAP: CHAP is an on-going community based study of individuals from a geographically defined community of 3 neighborhoods in Chicago, Illinois (Morgan Park, Washington Heights, and Beverly), with 6,158 participants in the first phase of the study (78.7% overall; 80.5% of the blacks, 74.6% of the whites)[53]. Data were collected in cycles of approximately 3 years; each consisting of an in-home interview of all participants and clinical evaluation of a random, stratified sample. The baseline cycle measured disease prevalence and provided risk factor data prior to incident disease onset. A cohort of 3,838 persons free of Alzheimer’s disease was identified; 729 persons were sampled for baseline clinical evaluation. Persons in the disease-free cohort had either good cognitive function at baseline, or if cognitive function was intermediate or poor, were free from Alzheimer’s disease at the baseline clinical evaluation. This disease-free cohort was evaluated for incident disease after an average of 4.1 years. Sampling for incident clinical evaluation was based on age, sex, race, and change in cognitive function (i.e., stable or improved, small decline, or large decline). The sample set available in the ADGC for genetic analyses included 27 Alzheimer’s disease cases and 144 persons free of Alzheimer’s disease at time of last assessment. All subjects were age 65 years or older at last assessment.

EAS: Based at the Albert Einstein College of Medicine, the EAS is an ongoing community based cohort study of cognitive aging and Alzheimer’s disease in the elderly which began over four decades ago. Please see Barzilai et al.[54] and Katz et al.[55] for details. The EAS cohort has employed systematic recruiting methods to reduce the selection biases that arise from clinic-based samples and to capture the racial diversity within the Bronx community. Since 1993, a total of 1,944 participants have been enrolled. Between 1993 and 2004, Health Care Financing Administration/Centers for Medicaid and Medicare Services (HCFA/CMS) rosters of Medicare eligible persons aged 70 and above were used to develop sampling frames of community residing participants in Bronx County. Since 2004, New York City Board of Elections registered voter lists for the Bronx have been used due to changes in policies for release of HCFA/CMS rosters. Individuals were mailed introductory letters regarding the study and were then telephoned to complete a brief screening interview. Eligible participants were at least 70 years of age, Bronx residents, non-institutionalized, and English speaking. Exclusion criteria included visual or auditory impairments that preclude neuropsychological testing, active psychiatric symptomatology that interfered with the ability

to complete assessments, and non-ambulatory status. Written informed consent was obtained at the initial clinic visit. In-person evaluations were completed at baseline and at subsequent 12-month intervals. Functional status was assessed by the self-administered CERAD C1-ALT, a cognitive/functional impairment instrument, and the Instrumental Activities of Daily Living scale (IADL), a subscale on the Lawton Brody Activities of Daily Living Scale. The score on the IADL was based on 5 domains of function that were common to both elderly men and women. Scores for each domain were dichotomized as impaired vs. not impaired and then the domain scores were summed. If the participant agreed, an informant completed the CERAD C2-ALT, a cognitive/functional impairment instrument, and the Informant Questionnaire on Cognitive Decline in the Elderly (IQ-CODE) 14 forms. The standard neurological physical examination was adapted from the Unified Parkinson's Disease Rating Scale. The evaluation assessed the participant's memory for significant recent events in the news and personal events. The coherence and focus of responses, repetitiveness, and language were determined. When possible, informants were interviewed to ascertain whether they noted any cognitive changes in the participant, and to assess accuracy of the participant's responses. The neurologist also assessed each participant for abnormal behaviors, fluctuation in cognition, and history of sleep disturbance and visual/auditory hallucinations. The neurologist assigned an Hachinski Ischemic Score (HIS), the Clinical Dementia Rating (CDR), and provided a clinical impression of presence or absence of dementia. A diagnosis of dementia was based on standardized clinical criteria from the Diagnostic and Statistical Manual, Fourth Edition (DSM-IV) and required impairment in memory plus at least one additional cognitive domain, accompanied by evidence of functional decline. Diagnoses were assigned at consensus case conferences, which included comprehensive review of cognitive test results, relevant neurological signs and symptoms, and functional status. Memory impairment was defined as scores in the impaired range on any of the memory tests in the neuropsychological battery. (FCSRT ≤ 2430 or 1.5 standard deviations (SD) below the age-adjusted mean on Logical Memory) Functional decline was determined at case conference based on information from self or informant report, impairment score on the IADL Lawton Brody Scale, clinical evaluation, and informant questionnaires. Alzheimer's disease was diagnosed in participants with dementia meeting clinical criteria for probable or possible disease established by the NINCDS-ADRDA[8,11]. Incident dementia and Alzheimer's disease were diagnosed in persons free of dementia at baseline who met criteria at follow-up. A subset of individuals who participated in the clinical studies of the EAS came to autopsy, providing an important quality control for diagnostic accuracy. A clinical diagnosis of dementia had a positive predictive value (PPV) of 96% for significant pathology upon autopsy. A clinical diagnosis of possible or probable Alzheimer's disease had a PPV of 79% for the presence of NIA-Reagan intermediate or high likelihood Alzheimer type pathology based on an autopsy sample of 175. For this study, EAS contributed data on 9 Alzheimer's disease cases and 141 healthy controls with Alzheimer's disease-free status confirmed as of most recent follow-up.

GenADA: GenADA study data analyzed included 666 Alzheimer's disease cases and 712 CNEs ascertained from nine memory referral clinics in Canada between 2002 and 2005. Patients and CNEs were of non-Hispanic White (NHW) ancestry from Northern Europe. All patients with Alzheimer's disease satisfied NINCDS-ADRDA and DSM-IV criteria for probable Alzheimer's disease with Global Deterioration Scale scores of 3-7[8,11]. CNEs had MMSE test scores higher than 25 (mean 29.2 ± 1.1), a Mattis Dementia Rating Scale score of ≥ 136, a Clock Test without error, and no impairments on seven instrumental activities of daily living questions from the Duke Older American Resources and Services Procedures test. Data were collected under an academic-industrial grant from Glaxo-Smith-Kline, Canada by Principal Investigator P. St George-Hyslop. Detailed characteristics of this cohort have been described previously[56].

MAYO/RMAYO: All 671 cases and 1,279 controls consisted of NHW subjects from the United States ascertained at the Mayo Clinic. All subjects were diagnosed by a neurologist at the Mayo Clinic in Jacksonville, Florida or Rochester, Minnesota. The neurologist confirmed a Clinical Dementia Rating score of 0 for all controls; cases had

diagnoses of possible or probable Alzheimer's disease made according to NINCDS-ADRDA criteria[8,11]. Autopsy-confirmed samples (221 cases, 216 CNEs) came from the brain bank at the Mayo Clinic in Jacksonville, FL and were evaluated by a single neuropathologist. In clinically-identified cases, the diagnosis of definite Alzheimer's disease was made according to NINCDS-ADRDA criteria. All Alzheimer's disease brains analyzed in the study had a Braak score of 4.0 or greater. Brains employed as controls had a Braak score of 2.5 or lower but often had brain pathology unrelated to Alzheimer's disease and pathological diagnoses that included vascular dementia, frontotemporal dementia, dementia with Lewy bodies, multi-system atrophy, amyotrophic lateral sclerosis, and progressive supranuclear palsy.

MIRAGE: The MIRAGE study is a family-based genetic epidemiology study of Alzheimer's disease that enrolled Alzheimer's disease cases and unaffected sibling controls at 17 clinical centers in the United States, Canada, Germany, and Greece (details elsewhere[57]), and contributed 1,229 subjects (491 Alzheimer's disease cases and 738 CNEs), a subset of the cases and controls that were incorporated into our prior studies[38,41] which met more stringent QC criteria for this study. Briefly, families were ascertained through a proband meeting the NINCDS-ADRDA criteria for definite or probable Alzheimer's disease[8,11]. Unaffected sibling controls were verified as cognitively healthy based on a Modified Telephone Interview of Cognitive Status score ≥ 86[58].

UM/CWRU/TARCC2: The UM/CWRU/TARCC2 sample included 256 cases and 189 controls from the University of Miami, Case Western Reserve University, and the Texas Alzheimer's Research Care Consortium (wave 2). All Alzheimer's disease cases had onset of disease symptoms after age 65 years and met NINCDS-ADRDA criteria for probable or possible Alzheimer's disease[8,11]. Controls were adjudicated to have MMSE scores greater than 28 and no clinically identified signs of cognitive impairment. Additional details of subject recruitment at these sites are described in the UM/CWRU/MSSM (formerly UM/VU/MSSM) and TARCC cohort descriptions in this supplement and elsewhere[38,40,59].

NIA-LOAD: The NIA LOAD Family Study[60] recruited families with two or more affected siblings with LOAD and unrelated, CNEs similar in age and ethnic background. A total of 1,819 cases and 1,969 CNEs from 1,802 families were recruited through the NIA LOAD study, NCRAD, and the University of Kentucky, with 1,798 cases and 1,568 CNEs included for analysis. One case per family was selected after determining the individual with the strictest diagnosis (definite > probable > possible LOAD). If there were multiple individuals with the strictest diagnosis, then the individual with the earliest age of onset was selected. The controls included only those samples that were neurologically evaluated to be normal and were not related to a study participant.

NBB: The Netherlands Brain Bank, which has been previously described elsewhere[59], is a department of the Netherlands Institute for Neuroscience, an institute of the Royal Netherlands Academy of Arts and Sciences. The NBB is a non-profit organization that collects human brain tissue from donors with a variety of neurological and psychiatric disorders and brain tissue from non-diseased donors, as well as anonymized summaries of donors' medical records to be made available for neuroscience research[61]. The sample set available in the ADGC for genetic analyses included 80 pathologically-confirmed Alzheimer's disease cases and 48 subjects free of Alzheimer's pathology at autopsy. All cases were age 65 years or older at time of diagnosis, and all controls were age 65 years or older at time of death.

OHSU: The OHSU dataset includes 132 autopsy-confirmed Alzheimer's disease cases and 153 deceased controls that were evaluated for dementia within 12 months prior to death (age at death > 65 years), which are a subset of the 193 cases and 451 controls examined in our previous study[41] meeting more stringent QC criteria in this study. Subjects were recruited from aging research cohorts at 10 NIA-funded ADC and did not overlap other samples assembled by the ADGC. A more extensive description of control samples can be found elsewhere[62].

Pfizer: The Pfizer sample collection comprises Alzheimer's disease cases taken from the Lipitor's Effect in Alzheimer's Disease (LEADe) trial, including subjects who converted to Alzheimer's disease after ascertainment as MCI, as well as 216 probable Alzheimer's

disease subjects enrolled by PrecisionMed for a case-control study and 149 subjects from a Phase II trial (#A3041005) of CP-457920 (a selective α5 GABAA receptor inverse agonist) in Alzheimer's disease. Samples were collected from multiple clinical sites, and with appropriate IRB/ethics committee approvals at each individual site, with written and informed consent given by subjects for use in follow-up studies. All subjects were diagnosed with probable or possible Alzheimer's disease if they met NINCDS-ADRDA and/or DSM-IV criteria, and had Mini-Mental Status Exam (MMSE) scores < 25 at baseline[8,11]. The control group included subjects from two studies: 1) the PrecisionMed case-control study (#A9010012), which recruited elderly subjects free of neurological or psychiatric conditions, and 2) 999-GEN-0583-001, which obtained a reference population of cognitively, neurologically, and psychiatrically normal subjects. Controls have no neuropsychiatric conditions or diseases and had MMSE>27 at the time of enrollment. For Alzheimer's disease analysis, all cases with age-at-onset (AAO) less than 65 years were removed to exclude early-onset Alzheimer's disease subjects. All controls were re-matched with remaining cases according to gender, age (all controls are older than cases), and ethnicity (only individuals with NHW background were analyzed). The final Pfizer Alzheimer's disease case-control GWAS dataset included 696 cases and 762 controls. Cases from the PrecisionMed/ A3041005 and LEADe studies and age-matched controls were genotyped using the Illumina HumanHap550 array. *APOE* genotypes were determined from genotypes for rs429358 and rs7412 obtained using Taqman assays.

ROSMAP: ROSMAP are two community-based cohort studies. The ROS has been ongoing since 1993, with a rolling admission. Through July of 2010, 1,139 older nuns, priests, and brothers from across the United States initially free of dementia who agreed to annual clinical evaluation and brain donation at the time of death completed their baseline evaluation. The MAP has been on-going since 1997, also with a rolling admission. Through July of 2010, 1,356 older persons from across northeastern Illinois initially free of dementia who agreed to annual clinical evaluation and organ donation at the time of death completed their baseline evaluation. Details of the clinical and neuropathologic evaluations have been previously reported[63–65]. A total of 1,064 persons passed genotyping QC. Of these, 295 met clinical criteria for Alzheimer's disease at the time of their last clinical evaluation or time of death and met neuropathologic criteria for Alzheimer's disease for those on whom neuropathologic data were available, and 769 were without dementia or MCI at the time of their last clinical evaluation or time of death and did not meet neuropathologic criteria for Alzheimer's disease for those on whom neuropathologic data were available. A second wave of ROSMAP (referred to as ROSMAP2 in this study) included 59 persons who met clinical criteria for Alzheimer's disease at the time of their last clinical evaluation or time of death and met neuropathologic criteria for Alzheimer's disease for those on whom neuropathologic data were available, and 217 persons who were without dementia or MCI at the time of their last clinical evaluation or time of death and did not meet neuropathologic criteria for Alzheimer's disease for those on whom neuropathologic data were available.

TARCC: The TARCC is a collaborative Alzheimer's research effort directed and funded by the Texas Council on Alzheimer's Disease and Related Disorders (the Council), as part of the Darrell K Royal Texas Alzheimer's Initiative. Composed of Baylor College of Medicine (BCM), Texas Tech University Health Sciences Center (TTUHSC), University of North Texas Health Science Center (UNTHSC), the UT Southwestern Medical Center at Dallas (UTSW), University of Texas Health Science Center at San Antonio (UTHSCSA), Texas A&M Health Science Center (TAMHSC), and the University of Texas at Austin (UTA), this consortium was created to establish a comprehensive research cohort of well characterized subjects to address better diagnosis, treatment, and ultimately prevention of Alzheimer's disease[66]. The resulting prospective cohort, the Texas Harris Alzheimer's Research Study, contains clinical, neuropsychiatric, genetic, and blood biomarker data on more than 3,000 participants diagnosed with Alzheimer's disease, mild cognitive impairment (MCI), and cognitively normal individuals. Longitudinal data/sample collection and follow-up on participants occurs on an annual basis. Two waves of case-control data from TARCC were examined as part of genetic analyses in the ADGC. Data from the TARCC included

323 cases and 181 controls in the first wave (included in the TARCC1 cohort), with 84 cases and 115 controls in the second wave (included in the UM/CWRU/TARCC2 cohort). All TARCC subjects were greater than 65 years of age at disease onset (cases) or at last disease-free exam (non-cases).

TGEN2: Among the TGEN2 data analyzed were 668 clinically- and neuropathologically-characterized brain donors, and 365 CNEs without dementia or significant Alzheimer's disease pathology. Of these cases and CNEs, 667 were genotyped as a part of the TGEN1 series[67]. Samples were obtained from twenty-one different National Institute on Aging-supported Alzheimer's disease Center brain banks and from the Miami Brain Bank as previously described[67–70]. Additional individual samples from other brain banks in the United States, United Kingdom, and the Netherlands were also obtained in the same manner. The criteria for inclusion were as follows: self-defined ethnicity of European descent, neuropathologically confirmed Alzheimer's disease or neuropathology present at levels consistent with status as a control, and age of death greater than 65. Autopsy diagnosis was performed by board-certified neuropathologists and was based on the presence or absence of the characterization of probable or possible Alzheimer's disease. Where possible, Braak staging and/or CERAD classification were employed. Samples derived from subjects with a clinical history of stroke, cerebrovascular disease, comorbidity with any other known neurological disease, or with the neuropathological finding of Lewy bodies were excluded.

UKS: The UKS cohort is a thoroughly diagnosed case-control cohort from Universitätsklinikum des Saarlandes, consisting of individuals clinically diagnosed with sporadic Alzheimer's disease (N = 596; mean age onset, 72.2 ± 6.6 years) and cognitively healthy, age-, gender-, and ethnicity-matched population-based controls (N = 170; 64.1 ± 3.0 years).

UM/CWRU/MSSM: The UM/CWRU/MSSM dataset (formerly UM/VU/MSSM[71–74]) contains 1,177 cases and 1,126 CNEs ascertained at the University of Miami, Case Western Reserve University and Mt. Sinai School of Medicine, including 409 autopsy-confirmed cases and 136 controls, primarily from the Mt. Sinai School of Medicine[75]. An additional 16 cases were included and 34 controls excluded from the data analyzed in the Jun et al. 2010 study[41]. Each affected individual met NINCDS-ADRDA criteria for probably or definite Alzheimer's disease[8,11] with age at onset greater than 60 years as determined from specific probe questions within the clinical history provided by a reliable family informant or from documentation of significant cognitive impairment in the medical record. Cognitively healthy controls were unrelated individuals from the same catchment areas and frequency matched by age and gender, and had a documented MMSE or 3MS score in the normal range. Cases and controls had similar demographics: both had similar ages-at-onset/ages-at-exam of 71.1 (±17.4 SD) for cases and 73.5 (±10.6 SD) for controls, and cases and controls were 64.5% and 61.3% female, respectively.

UPITT: The University of Pittsburgh dataset contains 1,255 NHW Alzheimer's disease cases (of which 277 were autopsy-confirmed) recruited by the University of Pittsburgh Alzheimer's Disease Research Center, and 829 NHW, CNEs ages 60 and older (2 were autopsy-confirmed). All Alzheimer's disease cases met NINCDS-ADRDA criteria for probable or definite Alzheimer's disease[8,11]. Additional details of the cohort used for GWAS have been previously published[76].

WASHU: An NHW LOAD case-control dataset consisting of 377 cases and 281 healthy elderly controls was used in analyses for this study. This dataset was split between two analysis datasets (WASHU1 and WASHU2). Participants were recruited as part of a longitudinal study of healthy aging and dementia. Diagnosis of dementia etiology was made in accordance with standard criteria and methods[46]. Severity of dementia was assessed using the Clinical Dementia Rating scale[77].

WHICAP: WHICAP is a community-based longitudinal study of aging and dementia among elderly, urban-dwelling residents[78,79]. Beginning enrolment in 1989, WHICAP has followed more than 5,900 residents over 65 years of age, including white, African American, and Hispanic participants. Detailed clinical assessments were performed at approximately

24-month intervals over the 7 years of the initial study. All interviews were conducted in either English or Spanish. The choice of language was decided by the subject to ensure the best performance, and the majority of assessments were performed in the subject's home, which included medical, neurological, and neuropsychological evaluations. Results of the neurological, psychiatric, and neuropsychological assessments were reviewed in a consensus conference comprised of neurologists, psychiatrists, and neuropsychologists. Based on this review all participants were assigned to one of three categories: dementia, cognitive impairment, or normal cognitive function. The sample set available in the ADGC for genetic analyses included 73 Alzheimer's disease cases and 560 subjects with normal cognitive function.

CSDC: The Combined Small Datasets Collection is a harmonized dataset including all data from eight separately ascertained datasets or waves of datasets already described above (ACT2, BIOCARD, CHAP2, EAS, NBB, RMAYO, ROSMAP2, and WASHU2). None of these datasets were separately incorporated into the analyses, and were only analyzed in the CSDC. Datasets or waves were incorporated into the CSDC if they included fewer than 100 cases and/or 100 CNEs. As all datasets were genotyped separately but imputed to the same dataset, genotyped single nucleotide polymorphisms (SNPs) overlapping all of the high-density genotyping platforms used were extracted and a set of ~20,000 variants were used to estimate both population substructure and explore potential heterogeneity between datasets using EIGENSTRAT/EIGENSOFT[80,81]. Data-set level association analyses similar to those described for all other cohorts and datasets were performed, though covariate adjustment additionally included indicator variables for study to adjust for residual batch effects not captured in PCs. Association results among common variants (minor allele frequency [MAF]>0.01) in the CSDC were similar to other datasets in the ADGC with very modest deflation (λ=0.9828) while rare variant associations demonstrated extreme patterns of association with a high degree of genomic inflation (λ=1.227). For this reason, only common variant association results from the CSDC were incorporated into common variant meta-analysis, while rare variant association results were excluded from rare variant meta-analysis (none of the eight individual datasets composing the CSDC were used in rare variant meta-analysis).

Cohorts for Heart and Aging Research in Genomic Epidemiology (CHARGE)

CHS: The Cardiovascular Health Study (CHS) is a population-based cohort study of risk factors for coronary heart disease and stroke in adults ≥65 years conducted across four field centers[82]. The original predominantly European ancestry cohort of 5,201 persons was recruited in 1989-1990 from random samples of the Medicare eligibility lists; subsequently, an additional predominantly African-American cohort of 687 persons was enrolled for a total sample of 5,888. Genotyping was performed using the Illumina 370CNV BeadChip system (for European ancestry participants, in 2007) or the Illumina HumanOmni1-Quad_v1 BeadChip system (for African-American participants, in 2010). CHS was approved by institutional review committees at each field center and individuals in the present analysis had available DNA and gave informed consent including consent to use of genetic information for the study of cardiovascular disease.

FHS: Framingham heart study (FHS) samples consist of 4350 well genotyped individuals from Original and Offspring cohorts[83,84]. The details of recruitment and surveillance of AD, and genotyping in FHS have been detailed previously[85]. Briefly, the Original cohort of the FHS has been evaluated biennially since 1948, was screened for prevalent dementia and AD since 1974-76. The Offspring cohort (offspring of original cohorts and spouse of offspring), recruited in 1971 and examined once every 4 years, have been screened for prevalent dementia with a neuropsychological battery and brain MRI. The AD status used in this study was taken from surveillance up to 2017. FHS participants had DNA extracted and provided consent for genotyping in the 1990s. Genotyping using the Affymetrix GeneChip® Human Mapping 500K Array Set and 50K Human Gene Focused Panel.® was attempted in 5293 Original and Offspring cohort participants.

FinnGen

FinnGen is a public-private partnership project that aggregates genotype data from Finnish biobanks (https://www.finngen.fi/en). The latest FinnGen release (Data Freeze 6) consists of 260,405 samples after quality control with population outliers excluded via principal component analysis based on genetic data. The samples have been linked with harmonized data from several national healthcare related registries. AD (Alzheimer's disease, wide definition, N=7,329) cases were identified from hospital discharge and cause of death registries having G30 (International Classification of Diseases (ICD)-10) or 29010 (ICD-8) codes, from Finnish-specific Social Insurance Institute (KELA) reimbursement registry having 307 or G30 (ICD-10) codes, and from medicine purchase registry having N06D (Anatomical Therapeutic Chemical, ATC) code. The same criteria were used as exclusion criteria for controls (N=252,879). The overlap between Finnish EADB and FinnGen controls is 0.3%.

Patients and control subjects in FinnGen provided informed consent for biobank research, based on the Finnish Biobank Act. Alternatively, older research cohorts, collected prior the start of FinnGen (in August 2017), were collected based on study-specific consents and later transferred to the Finnish biobanks after approval by Fimea, the National Supervisory Authority for Welfare and Health. Recruitment protocols followed the biobank protocols approved by Fimea. The Coordinating Ethics Committee of the Hospital District of Helsinki and Uusimaa (HUS) approved the FinnGen study protocol Nr HUS/990/2017.

The FinnGen study is approved by Finnish Institute for Health and Welfare (permit numbers: THL/2031/6.02.00/2017, THL/1101/5.05.00/2017, THL/341/6.02.00/2018, THL/2222/6.02.00/2018, THL/283/6.02.00/2019, THL/1721/5.05.00/2019, THL/1524/5.05.00/2020, and THL/2364/14.02/2020), Digital and population data service agency (permit numbers: VRK43431/2017-3, VRK/6909/2018-3, VRK/4415/2019-3), the Social Insurance Institution (permit numbers: KELA 58/522/2017, KELA 131/522/2018, KELA 70/522/2019, KELA 98/522/2019, KELA 138/522/2019, KELA 2/522/2020, KELA 16/522/2020 and Statistics Finland (permit numbers: TK-53-1041-17 and TK-53-90-20).

The Biobank Access Decisions for FinnGen samples and data utilized in FinnGen Data Freeze 6 include: THL Biobank BB2017_55, BB2017_111, BB2018_19, BB_2018_34, BB_2018_67, BB2018_71, BB2019_7, BB2019_8, BB2019_26, BB2020_1, Finnish Red Cross Blood Service Biobank 7.12.2017, Helsinki Biobank HUS/359/2017, Auria Biobank AB17-5154, Biobank Borealis of Northern Finland_2017_1013, Biobank of Eastern Finland 1186/2018, Finnish Clinical Biobank Tampere MH0004, Central Finland Biobank 1-2017, and Terveystalo Biobank STB 2018001.

1.3. Longitudinal studies

14 longitudinal cohorts were included in the genetic risk score (GRS) analysis (Supplementary Table 33). We used MCI patients from: the Dutch Amsterdam dementia cohort (ADC)[86], the German dementia competence network cohort (DCN)[9], two cohorts from the Spanish Fundacio ACE memory clinic cohort (FACE, AMC)[87], the French Balthazar cohort (HAN)[88], the Belgian memory clinic cohort from the Hospital Network Antwerp (UAN)[89], the German memory clinic of Halle (UHA), and the German memory clinic of Mannheim (ZIM)[90]. From the population based studies we used patients from: the German study on aging, cognition and dementia (AgeCoDe)[91], the Austrian VITA study[92], the Australian Sydney Memory and Ageing Study[18,93], the French Three City study (3C)[25], the Framingham Heart Study[83,84] and the Dutch Rotterdam study[24,94]. All selection criteria for the MCI patients and criteria used to define conversion to dementia are provided in the respective references.

2. Quality control

2.1. EADB

Genotyping

EADB genomic DNA samples were transferred to 3 genotyping centers: the Centre National de Recherche en Génomique Humaine, Evry, France (CNRGH), the Erasmus Medical Center, Rotterdam, the Netherlands (Erasmus MC) and the LIFE & BRAIN Center, Bonn, Germany (LIFE & BRAIN Gmbh). Samples that passed the DNA quality control (QC) were genotyped with the Illumina Infinium Global Screening Array (GSA, GSAsharedCUSTOM_24+v1.0). Raw probe intensities were shared with the CNRGH, which performed the genotype calling on all samples using the same custom cluster file obtained with the GenTrain 3.0 clustering algorithm (https://www.illumina.com/content/dam/illumina-marketing/documents/products/technotes/gentrain3-technical-note-370-2016-015.pdf). Of note, insertion and deletion polymorphisms were excluded from this process and only single nucleotide polymorphisms were called. During the genotyping QC process, three genotyping batches were considered: (1) 49 genotyping chips were identified as possibly problematic and thus were considered as a separate batch (denoted possibly problematic chips batch or PPC batch in the next sections), (2) a batch of samples was genotyped and processed after all other samples (denoted last genotyped batch or LGB batch in the next sections) and (3) the main batch including all other samples.

Chip assessment

Prior to the initial QC, positions and alleles of variants were assessed. First, using the Illumina support files (https://support.illumina.com/array/array_kits/infinium-global-screening-array/downloads.html), variants for which the position was erroneous in the first version of the manifest had their positions corrected and variants which are part of the removed markers list provided by Illumina (i.e., exclusion because of multi-mapping, poor clustering, non-validated correlation against 1000 Genomes data, multinucleotide variants or discrepant rsID) were excluded. Then, variants' probes were aligned against both Human reference genome assemblies GRCh37 (GRCh37.p13) and GRCh38 (GRCh38.p12) using the bwa software v0.7.17 with the BWA-MEM algorithm[95]. Only variants for which the full-length probe(s) aligned uniquely on the genome without any mismatch were retained. Next, the GRCh37 coordinates of variants were remapped to the GRCh38 assembly (GCF_000001405.26) using the NCBI Remapping Service (https://www.ncbi.nlm.nih.gov/genome/tools/remap). Variants which were unmapped, not mapped on a primary contig of the Primary Assembly (chromosomes 1-22, X, Y, MT), or with a discordant position according to the previous alignment step on GRCh38, were excluded. Last, a normalization process of the alleles was performed on both GRCh37 and GRCh38 assemblies using the bcftools v1.9 software (http://www.htslib.org/doc/bcftools.html) in order to obtain alleles expressed on the plus strand. Variants showing incompatible alleles against the reference assembly were removed. Finally, only variants passing in all steps, for both GRCh37 and GRCh38 assemblies, were included and their coordinates and alleles were set according to the GRCh38 assembly for the rest of the pipeline.

Variant Intensity Quality Control

A QC on the intensity metrics extracted from the Illumina GenomeStudio software v2.0.3 (https://www.illumina.com/techniques/microarrays/array-data-analysis-experimental-design/genomestudio.html) was then performed on all autosomes and chromosome X variants. Only the metrics from the main batch were used in this QC. The involved intensity metrics and their thresholds were adapted from the CHARGE Consortium HumanExome BeadChip quality control paper[96]. Variants were removed based on the exclusion criteria described in the Supplementary Table 46.

Sample Quality Control

To be consistent with the main batch and PPC batches, the sample QC for the LGB batch, performed afterwards, used the same thresholds (including the steps which require the computation of a metric mean or median).

Pre-quality control. First, a pre-quality control was performed on both autosomes and the chromosome X variants. Variants having a *P* value <1e-15 for the Hardy-Weinberg equilibrium test in controls (in females only for chromosome X variants) in at least one genotyping center or globally, or showing a missingness >0.05 in at least one genotyping center or >0.025 globally, were excluded prior to all the following sample quality control steps.

Heterozygosity and missingness. Sample missingness was computed using all autosomal variants while the sample heterozygosity was computed at the pruned set of autosomal variants (maximum r^2 of linkage disequilibrium (LD) set to 0.2 with a window of 500kb) using PLINK v1.9 (https://www.cog-genomics.org/plink2/). Samples showing a missingness >0.05 or showing a heterozygosity metric (Method-of-moments F coefficient estimate) outside the interval mean ±6 standard deviation (sd) were removed.

Population outliers. In order to identify population outliers, a principal component analysis (PCA) was performed. The 1000 Genomes Phase3 data (1000GP3) called on the GRCh38 assembly was used as the reference (http://ftp.1000genomes.ebi.ac.uk/vol1/ftp/data_collections/1000_genomes_project/release/20190312_biallelic_SNV_and_INDEL/). First, a subset of variants was selected to be included in the PCA: variants in common between 1000GP3 and the GSA variants passing the variant QC, having a minor allele frequency (MAF) >0.01 in both 1000GP3 and EADB, not ambiguous (i.e., A/T and C/G variants) and not located in high LD regions as described here[97] as well as LCT (2q21), HLA and 2 inversion regions (8p23 and 17q21.31) following the TOPMed analysis pipeline described here (https://github.com/UW-GAC/analysis_pipeline). Variants were then pruned using PLINK v1.9 (maximum r^2 of LD set to 0.2 using a window of 500kb). Principal components (PCs) were computed on the 1000GP3 samples and the EADB samples were projected onto these PCs using the FlashPCA v2.0 software[98]. Samples falling outside the interval median ±12 median absolute deviation (mad), computed using only the EADB samples, on PC1 or on PC2 were flagged as population outliers.

Sex-check. A sex-check of samples was performed to identify discordances between the genetic and the clinical sex information. All chromosome X variants of the non pseudoautosomal region, passing the variant pre-quality control, were pruned (maximum r^2 of LD set to 0.2 with a window of 500kb) and then used in the sex-check function of PLINK v1.9. Using default parameters of PLINK v1.9 for exclusion on the resulting inbreeding coefficient F (i.e., F <0.8 for males, F >0.2 for females), samples with a discordant genetic and clinical sex were removed. Samples who had no clinical sex available were not excluded and their imputed genetic sex was used as a replacement in latter analyses. Also, plates showing more than 30% of failed sex-check samples were excluded.

Relatedness. An analysis was performed to infer the relatedness between samples using the GENESIS v2.14.4 package[99]. This package provides methods to infer relatedness by taking into account the population structure. The pipeline described on the package documentation (https://bioconductor.org/packages/release/bioc/vignettes/GENESIS/inst/doc/pcair.html) was followed. All samples already excluded by a previous sample QC step or flagged as population outlier were excluded of this analysis. The included variants were the same as the ones included in the population outlier analysis minus the ones failing the variant QC described below (missingness, differential missingness test, Hardy-Weinberg test or frequency test). The default parameters of the pipeline were used at the exception of the kinship and divergence thresholds, used to assign relatives and samples of divergent ancestry in the PCAir step, which were set to 2^(-9/2) and -2^(-9/2) respectively, following the TOPMed analysis pipeline. Also, 21 PCs were used in the PC-Relate step of the pipeline which computes the final kinship coefficients. All pairs with a kinship >0.09375 (representing

the mean between the 2nd and 3rd degrees) were then selected and processed in 3 different categories: duplicate samples (samples with a pairwise kinship >0.45), multi-related samples (samples related to more than one sample) and samples related to only one other sample. First, the duplicate samples were processed, excluding all involved samples if there was a status or sex mismatch (not considering a missing variable as discordant). If the clinical variables matched, samples were excluded in the following order: (1) the sample from the PPC batch, (2) the sample from cohorts not being imputed with TOPMed, (3) the sample with a missing status or (4) the sample with the highest missingness. Concerning the multi-related samples, samples were sorted by considering first the one with the most related samples (sorting also alphanumerically by the sample identifier in case of multiple samples with the same number of related samples for reproducibility) and this sample was excluded. All pairs involving this excluded sample were then discarded and the process was repeated until no more multi-related samples remain. Finally, for the remaining pairs, samples were excluded in the following order: (1) the sample from the PPC batch, (2) the sample with a pathogenic mutation, (3) the sample from cohorts not being imputed with TOPMed, (4) the control over the case or the sample with a missing status over the control or (5) the sample with the highest missingness.

Possibly problematic chips batch. After all the sample QC steps, we assessed the impact of the samples present in the PPC batch by performing a Genome-Wide Association study (GWAs) on all the EADB GSA samples and noticed many false positives signals coming from these samples so that this batch was entirely excluded.

Variant Quality control

For the variant QC, the initial set of autosomal variants passing the variant intensity quality control was used (re-integrating the variants failing the pre-quality control of the sample QC). All samples failing the sample QC were removed prior to the variant QC.

Missingness and Hardy-Weinberg equilibrium. The variants missingness, the p-value of the differential missingness test between cases and controls and the Hardy-Weinberg equilibrium test *P* value were computed using PLINK v1.9. Variants showing a missingness >0.05 in at least one genotyping center or having a *P* value of the Fisher's exact test on cases/controls missing calls <1e-10, were excluded. The Hardy-Weinberg equilibrium tests were performed only in controls and for each genotyping center/country pair separately. A variant was excluded if at least one center/country test showed a *P* value <5e-8.

Frequency checks. Two frequency tests of variants were performed to compare the frequency in the EADB GSA samples, excluding population outliers, against two reference panels: (1) the Haplotype Reference Consortium[100] (HRC) and (2) the Genome Aggregation Database[101] (gnomAD). For HRC, the release r1.1 was used and the frequency extracted was the one excluding 1000 Genome samples. For gnomAD, the release v3 was used as this version contains more than 70,000 whole genomes and thus has a better estimation for non exonic variants than the v2.1.1 which mostly contains whole exomes. Finnish and non-Finnish allele counts and frequencies were extracted from gnomAD sites having a PASS filter with more than 50% of the genomes called. To perform the frequency test, a Pearson chi-square test was performed on the allelic counts. After graphical review of the chi-squared test statistics (χ^2) distribution, variants having (1) χ^2 >3,000 in both HRC and gnomAD, (2) χ^2 >3,000 in HRC and not present in gnomAD or (3) χ^2 >3,000 in gnomAD and not present in HRC were excluded because of large difference of frequency. When the gnomAD and HRC tests disagreed (i.e., <3,000 in one and > 3,000 in the other), the variant was kept due to the uncertainty. Finally, when no frequency information was available from both HRC and gnomAD, the variant was excluded if it showed a different minor allele compared to the TOPMed reference panel freeze5[102] with a MAF difference higher than 0.2. Finally, to assess the frequency difference between genotyping centers, genome wide association studies (GWAS) were performed between controls across genotyping centers after excluding population outliers and related samples. First, PCs for all controls were computed with flashPCA2 using the same variants included in the relatedness estimation. Then we performed the analyses to compare controls from one genotyping center to controls from

another genotyping center (3 analyses in total). The center was thus converted into a binary variable and used as the analysis phenotype. Adjustments included PCs associated significantly with the center and the analyses were performed with the SNPTEST software[103], v2.5.4-beta3 using an additive model with the new model-fitting functionality (newml method). All variants having a Likelihood Ratio Test *P* value <1e-5 were excluded.

Ambiguous variants. All ambiguous variants (e.i: A/T and C/G) showing a MAF >0.4 were excluded.

Duplicated variants. Concerning the duplicated variants of the chip, only the copy with the minimum missingness was kept.

Last genotyped batch supplementary steps. Since this batch was available after the variant QC of the main batch, only the variants passing all QC steps in the main batch were used as the initial set of variants prior to the batch variant QC. All steps described in this section were then performed for that batch separately. Also, since this batch contains only German samples, one supplementary step was performed: a GWAS between controls from this batch and German controls from the main batch following the same pipeline described in the Frequency checks section.

Analysis principal components computation

The principal components used as adjustment in the analysis were computed using AD cases and controls only, on the same variants which were retained for the relatedness estimation. These PCs were computed using the FlashPCA v2.0 software.

Clinical data QC

After QC of the genotyping data, we additionally excluded controls with age below 30 and individuals with known pathogenic mutations.

The EADB study finally included 20,464 AD cases and 22,244 controls for 606,881 autosomal variants (Supplementary Fig. 49 and 50). Among those, 20,301 AD cases and 21,839 controls were imputed with the TOPMed reference panel (EADB-TOPMed) while 163 AD cases and 405 controls were imputed with the HRC reference panel (EADB-HRC) as described in the Imputations section.

2.2. Other datasets

European Alzheimer's Disease Initiative (EADI) Consortium

The EADI chip assessment only included the alignment, remapping and normalization step. The sample QC was already detailed here[85] and only the relatedness step was redone using the methodology used for the EADB GSA samples. The variant QC followed the same pipeline and steps than the EADB GSA chip with the same metrics and thresholds at the exception of the Frequency tests where (1) only allele counts from non-Finnish samples were extracted from the gnomAD reference panel and (2) the χ^2 threshold used was set to 1,500 because of the EADI sample size. In addition, variants having a MAF <0.01 were excluded. Also, no controls GWAS across controls/centers step was performed because not applicable for this study. After QC and exclusion of individuals with known pathogenic mutations, the EADI study included 2,400 AD cases and 6,338 controls for 523,431 autosomal variants.

Genetic and Environmental Risk in AD (GERAD) Consortium

We removed individuals with missing genotype rates > 0.01. We also applied a filter based on mean autosomal heterozygosity, inconsistencies between reported gender and genotype-determined gender. All individuals passing these QC filters were examined for potential genetic relatedness by calculating identity-by-descent (IBD) estimates for all possible pairs of individuals in PLINK, and removing one of each pair with an IBD estimate ⩾0.125. We assessed population structure within the data using principal components analysis as

implemented in EIGENSTRAT[80] to infer continuous axes of genetic variation. Eigenvectors were calculated based on LD-pruned SNPs common to all arrays. The EIGENSTRAT program also identifies genetic outliers, which are defined as individuals whose ancestry is at least 6 s.d. from the mean on one of the top ten axes of variation. Individuals either were genotyped on the Illumina 610-quad chip or on the Illumina HumanHap550 array. We assessed the effects of different missing data rates and Hardy-Weinberg filters, aiming to remove poorly performing SNPs without excluding markers that may show genuine association with Alzheimer's disease. Markers were excluded if they had a minor allele frequency (MAF) <0.01 or a Hardy-Weinberg $P \geqslant 1 \times 10^{-5}$ in either cases or controls. SNPs with a MAF $\geqslant$0.05 were excluded if they had a genotype missing rate of >0.03 in either cases or controls; for SNPs with a MAF between 0.01 and 0.05, a more stringent genotype missing rate threshold of 0.01 was used. To minimize inter-chip and inter-cohort differences minor allele frequencies were compared between controls in the different groups using logistic regression analysis, incorporating the top four PCs as covariates as previously described. Comparisons were performed only between individuals from the same geographical region (that is, British Isles, Germany or USA). For each of the four categories of SNPs, a quantile-quantile (Q-Q) plot was produced for each cohort control comparison, and the significance threshold used to exclude SNPs was based on where the observed χ^2 statistics departed from the null expectation. Finally, we applied a variant QC following the same pipeline and steps than the EADB GSA chip with the same metrics and thresholds at the exception of the Frequency tests where (1) only allele counts from non-Finnish samples were extracted from the gnomAD reference panel and (2) the χ^2 threshold used was set to 1,500 because of the GERAD sample size. After QC, the GERAD study included 3,030 AD cases and 7,153 controls for 418,258 autosomal variants.

Bonn studies

DietBB: The dietBB chip assessment only included the alignment, remapping and normalization step. The sample and variant QCs followed the same pipeline and steps than the EADB GSA chip with the same metrics and thresholds at the exception of the Frequency tests where (1) only allele counts from non-Finnish samples were extracted from the gnomAD reference panel and (2) the χ^2 threshold used was set to 250 because of the dietBB sample size. Also, no control GWAs across controls/centers step was performed because not applicable for this study. After QC, the dietBB study included 139 AD cases and 177 controls for 630,058 autosomal variants.

Bonn OMNI cohort: The Bonn OMNI chip assessment only included the alignment, remapping and normalization. The sample and variant QCs followed the same pipeline and steps than the EADB GSA chip with the same metrics and thresholds at the exception of the Frequency tests where (1) only allele counts from non-Finnish samples were extracted from the gnomAD reference panel and (2) the χ^2 threshold used was set to 500 because of the Bonn OMNI sample size. Also, no controls GWAs across controls/centers step was performed because not applicable for this study. After QC, the Bonn OMNI study included 496 AD cases and 1,033 controls for 789,359 autosomal variants.

DemGene (DG) Consortium

deCODE chip batch: The deCODE chip assessment only included the alignment, remapping and normalization step. The sample and variant QCs followed the same pipeline and steps than the EADB GSA chip with the same metrics and thresholds at the exception of Hardy-Weinberg tests were all samples were included in the test, instead of only controls, because of the low number of controls. The Frequency test was also modified so that (1) only allele counts from non-Finnish samples were extracted from the gnomAD reference panel and (2) the χ^2 threshold used was set to 250 because of its sample size. Also, no controls GWAs across controls/centers step was performed because not applicable for this study. After QC, the deCODE chip batch included 300 AD cases and 11 controls for 638,952 autosomal variants.

Omni chip batch: The Omni chip assessment only included the alignment, remapping and normalization step. The sample and variant QCs followed the same pipeline and steps than the EADB GSA chip with the same metrics and thresholds at the exception of the Frequency test where (1) only allele counts from non-Finnish samples were extracted from the gnomAD reference panel and (2) the χ^2 threshold used was set to 1,500 because of its sample size. Moreover, there were multiple batches for this Omni chip which were taken into account for the differential missingness and the GWAs across controls. For the differential missingness, the test was performed globally as well as for the 4 batches including both cases and controls with enough sample sizes and, following the EADB GSA pipeline, a variant was excluded if it failed in at least one test. For the GWAs across controls, we selected the 4 batches with more than 400 controls and performed the GWAs following the same pipeline and thresholds than the EADB GSA chip. After QC, the Omni chip batch included 1,393 AD cases and 5,915 controls for 654,313 autosomal variants.

The Copenhagen City Heart Study (CCHS, Denmark)

The CCHS chips assessments were performed in parallel for both chips and only included the alignment, remapping and normalization step. Since common samples were genotyped on both chips, the sample and variant QCs were performed in parallel for both chips and followed the same pipeline and steps than the EADB GSA chip with the same metrics and thresholds at the exception of the population outlier QC where samples falling outside the interval median ±4 mad, computed using only CCHS samples, on PC1 or on PC2 were flagged as population outliers and the Frequency test where (1) only allele counts from non-Finnish samples were extracted from the gnomAD reference panel and (2) the χ^2 threshold used was set to 1,500 because of the sample size. After the quality control steps performed for both chips, we merged the samples and variants passing individual chip QCs using PLINK v1.9 where discordant genotypes between the 2 chips were set to missing. After the merging process, all the sample and variants QCs steps were performed again using the same pipeline except for the relatedness estimation for which the KING software[104] was used because of the absence of population structure. Finally, after performing the PCA analysis on all CCHS samples, some outliers were identified on the first PC and were excluded (PC1 <-0.05). After QC, the CCHS study included 365 AD cases and 6,106 controls for 467,446 autosomal variants.

GR@ACE

The sample and variant QCs followed similar pipeline and steps than the EADB QC. Duplicated samples between GR@ACE and EADB were excluded.

Relatedness across studies

Relatedness was inferred across each pair of the following studies: Bonn, CCHS, EADB, EADI, DemGene and GERAD. The process followed the same methods described in section 2, restricting the initial set of variants to variants in common between the two studies. Using the same thresholds, one sample of each related pair was excluded in one study, while the other sample was retained in the other study. The number of related and duplicate samples between processed studies can be found in Supplementary Table 47.

3. Imputations

TOPMed imputations

All samples and variants passing the QC were used as the input of the imputation process. The imputation was performed by the Michigan Imputation Server[105] where the TOPMed Freeze5 reference panel was granted to the EADB consortium. The server version used was the 1.2.4 with Eagle v2.4[106] as the phasing software and Minimac4 v4-1.0.2 as the imputation software. Due to the limitation in terms of maximum number of samples per job (20,000), the GSA samples were split into 5 batches (4 for the main batch and one for the LGB batch). After the imputation process of all batches, in order to have a global imputation

quality, a merged imputation quality was recomputed including all samples using the bcftools impute_info plugin.

HRC imputations

Samples which were imputed with the HRC reference panel were sent to the Sanger Imputation server (https://imputation.sanger.ac.uk/) where the HRC reference panel r1.1 was used. The data were phased using the Eagle v2.0.5 software and the imputation process was performed by PBWT v3.1.

For studies imputed with the HRC panel, we excluded from the meta-analysis variants with a very large difference of frequency between the HRC and TOPMed panels (χ^2 >15,000). For the UK Biobank study, we further excluded variants with a very large difference of frequency between the UK10K+1000G or UK10K or 1000G panels and the TOPMed panel (χ^2 >5,000).

4. GRCh37/GRCh38 conversion

Prior to the meta-analysis, a conversion for variants from GRCh37 assemblies (i.e., studies not imputed with the TOPMed reference panel) was performed. First, variant positions were lifted from the GRCh37 assembly to the GRCh38 assembly using the UCSC liftover software (https://genome.ucsc.edu/cgi-bin/hgLiftOver). Variants failing the lift process were removed. Then, a normalization followed by a left-alignment process of these GRCh38 positions was performed using the bcftools v1.9 software to obtain the reference alleles in the GRCh38 assembly. Comparing the reference alleles between GRCh37 and GRCh38 allowed to define if the GRCh37 alleles needed a flip, a swap or both in order to be represented in the GRCh38 assembly. For ambiguous variants, we also compared the flanking sequences (10 bases) of the variant in order to decide if a flip or a swap of alleles was needed. When results of the comparison was too complex to interpret or when the flanking sequences show more than 1 mismatch between the 2 assemblies, the variant was discarded.

5. Stage II analyses

Alzheimer disease genomic consortium (ADGC)

Variant- and sample-level quality control (QC). Standard QC was performed on individual datasets using PLINK v1.9[107–109] and including filtering and re-estimating all quality metrics after excluding variants with a missingness rate of >10% of genotype calls. QC filters included exclusions on SNPs with call rates below 98% for Illumina and 95% for Affymetrix panels; SNPs with departure from Hardy-Weinberg Equilibrium (HWE) of $P<10^{-6}$ among cognitively-normal elders (CNEs, either non-cases or controls) for variants of MAF>0.01; and SNPs with informative missingness by case-CNE status of $P<10^{-6}$. Samples were dropped if the individual call rate was <95%; if X chromosome heterozygosity indicated inconsistency between predicted and reported sex; or if population substructure analyses (described below) indicated the sample did not cluster with 1000 Genomes Phase 3 populations of European ancestry.

Relatedness Check. Relatedness was assessed using the "--genome" function of PLINK v1.9. Using ~20,000 LD-pruned SNPs sampled from among genotyped variants, pi-hat (the proportion of alleles shared IBD) was estimated across all pairs of subjects across all ADGC datasets. Among pairs of subjects with no known familial relationships, one sample was excluded among pairs with pi-hat>0.95 if phenotype and covariate data matched, otherwise both samples were excluded; among all pairs with pi-hat>0.4 but less than 0.95, one sample was kept giving preference to cases over CNEs, age (earlier age-at-onset among case pairs, later age-at-exam among CNE pairs). Pairs of relatives were dropped from family datasets if pi-hat differed substantially from expectation based on their reported relationships.

Populations substructure. To identify samples of non-European ancestry, we performed a principal components (PCs) analysis using 'smartpca' in EIGENSOFT v7.2.1

[80,81] on the subset of ~20,000 LD-pruned SNPs used for relatedness checks on genotypes from all samples within each individual dataset and from the 1000 Genomes Phase III reference panels. Subjects not clustering with European ancestry groups were excluded from analysis. To account for the effects of population substructure in our analysis, a second PC analysis was performed using only the remaining subjects in each dataset. PCs 1-10 were examined for association with AD case-control status and eigenvector loading, and only PCs showing nominal association with AD ($P<0.05$) and eigenvector loadings >3 were used in covariate adjustment for populations substructure (average number of PCs used is 3; range: 2-4).

Imputation. For each dataset, SNPs not directly genotyped were imputed on the Michigan Imputation Server (MIS)[105] using samples of all ancestries available on the Haplotype Reference Consortium (HRC) v1.1 reference panel[100], which includes 39,235,157 SNPs observed on 64,976 haplotypes (from 32,488 subjects), all with an estimated minor allele count (MAC)≥5 and observed in samples from at least two separately-ascertained data sources. Phasing on the MIS was done with Eagle v2.4.1[106] while imputation was performed using Minimac3[105]. Quality of imputation for all variants was assessed using R^2 for imputation quality, although all variants were retained and not filtered prior to analysis. For rare variants, a global average of R^2 across all datasets weighted by sample size was considered.

Single-variant Association Analysis and Meta-analysis for Common Variants (MAF>0.01). Single variant-based association analysis on datasets of unrelated cases and CNEs were performed in SNPTEST v2.5.6[103] using score-based logistic regression under an additive model, with adjustment for PCs only. Family-based datasets were analyzed using the GWAF v2.2 package in R[110] which implemented a generalized estimating equation (GEE) approach to account for correlation between subjects. For each study, we filtered (i) variants with missing effect size, standard error or *P* value, (ii) variants with absolute value of effect size above 5, (iii) variants with imputation quality below 0.3. Within-study association results
were meta-analyzed using a fixed-effects approach with inverse variance-weighting using METAL v2011-03-25[111].

Single-variant Association Analysis and Meta-analysis for Rare Variants (MAF≤0.01). Rare variant association and meta-analysis was performed for individual variants using the SeqMeta package in R[112,113]. SeqMeta v.1.6.7 performs a score-based logistic regression, estimating scores in individuals using 'prepScores()' and performing meta-analysis using 'singleSNPMeta()'. Family-based datasets were analyzed by selecting a maximally-informative subset of unrelated individuals for analysis, and no datasets with fewer than 100 cases and/or CNEs were analyzed (including the CSDC which demonstrated extreme association patterns and genomic inflation suggesting potential bias). As in common variant analyses, models evaluated included covariate adjustment for PCs. After meta-analysis, we filtered (i) variants with missing effect size, standard error or *P* value, (ii) variants with absolute value of effect size above 5, (iii) variants with average imputation quality of $R^2< 0.3$.

GRCh38/GRCh37 conversion. We converted variant positions and alleles from the GRCh37 assembly to the GRCh38 assembly (see above), and excluded variants for which conversion was not possible or problematic.

Cohorts for Heart and Aging Research in Genomic Epidemiology (CHARGE)

CHS: In CHS, the following exclusions were applied to identify a final set of 306,655 autosomal SNPs: call rate < 97%, HWE *P* < 10-5, > 2 duplicate errors or Mendelian inconsistencies (for reference CEPH trios), heterozygote frequency = 0, SNP not found in imputation reference panel. Imputation to the TOPMed Freeze5 panel was performed on the Michigan imputation server. SNPs were excluded for variance on the allele dosage ≤0.01. These analyses were limited to the 2152 European ancestry participants from the CHS Memory Study[114] with successful genotyping.

FHS: 4425 persons met QC criteria (call rate>97%, no extreme heterozygosity or high Mendelian error rate). Imputation to the TOPMed Freeze5 panel was performed on the

Michigan imputation server. GWAS was carried out using a logistic regression model fitted via generalized estimating equations, with each family as a cluster, minimally adjusting for cohort status and the first and ninth PCs that were associated with the outcome.

CHARGE meta-analysis: For each study, we filtered (i) variants with missing effect size, standard error or *P* value, (ii) variants with absolute value of effect size above 5, (iii) variants with imputation quality below 0.3. FHS and CHS results were then combined with a fixed-effect meta-analysis (inverse variance weighted approach) as implemented in METAL v2011-03-25 to obtain the CHARGE results.

FinnGen

Detailed description of the FinnGen analysis pipeline can be found on the FinnGen website (https://finngen.gitbook.io/documentation/methods/phewas). Briefly, genome statistics were analyzed using Scalable and Accurate Implementation of Generalized mixed model (SAIGE v0.36.3.2), which uses saddle point approximation (SPA) to calibrate unbalanced case-control ratios[115]. The first ten genetic PCs, sex, age, and genotyping batch were used as covariates.

Stage II meta-analysis

A fixed-effect meta-analysis was performed with METAL v2011-03-25 (inverse variance weighted approach) to combine ADGC, CHARGE and FinnGen results.

6. Conditional analyses

In some regions, several variants were identified associated at the genome-wide significance level. In each of those regions, we performed an approximate conditional analysis of each associated variant conditionally on each other variant. The analyses were run with the GCTA-COJO v1.93.2beta approach[116,117], using the same EADB-TOPMed LD reference panel as in the PLINK v1.9 clumping procedure. We repeated those analyses by performing exact conditional analyses as implemented in SNPTEST v2.5.4-beta3 on the EADB-TOPMed dataset.

Those conditional analyses were also run between 1) the *OARD1* and *WWOX* variants detected by Kunkle et al[85], and our top variants in the *TREM2* and *MAF* loci respectively; 2) the *TRIP4* variant detected by Ruiz et al[118] and our top variant in the *SNX1* locus; 3) the *ABCA7* variant with *P* value < $5x10^{-8}$ in the Stage I + II analysis but with negative follow-up results in Stage II (see Supplementary Table 6) and our top variant in the *ABCA7* locus.

The results are provided in Supplementary Tables 3 and 4. According to those conditional analyses, the following pairs of loci can be considered as independent: i) *UMAD1* and *ICA1*; ii) *CLU* and *PTK2B*; iii) *APH1B* and *SNX1*; iv) *SNX1* and *TRIP4*; v) *DOC2A* and *KAT8*; vi) *WWOX* and *MAF*; vii) *ABCA7* and *KLF16*; viii) *APP* and *ADAMTS1*. Besides, we identified several independent signals in the *MME*, *TREM2*, *SORL1*, *IGH* gene cluster, and *PLCG2* loci. In the *ABCA7* loci, the two tested signals are also independent. However, the clumping procedure identified several signals in the *CELF1*/*SPI1* and *MAPT* loci respectively, which are not independent according to those conditional analyses.

After validation by conditional analyses (Supplementary Tables 3 and 4), this approach led us to define 39 signals in 33 loci already known to be associated with the risk of developing AD and related dementia (ADD) and to propose 42 new loci (Table 1, Supplementary Table 5 and Supplementary Fig. 2-29). Six of these loci (*APP*, *ANK3*/*CCDC6, NCK2, PRKD3, TSPAN14* and *SHARPIN*) have already been reported in two preprints that examined some of the GWAS data included in our study[119,120].

7. HLA analyses

These analyses were restricted to diagnosed cases in the following datasets: EADB-TOPMed, GR@ACE/DEGESCO, GERAD, EADI, DemGene, Bonn, CCHS and EADB-HRC.
Two-field resolution alleles of *HLA-A*, *HLA-B*, *HLA-C* class I genes and *HLA-DPB1*, *HLA-DQA1*, *HLA-DQB1*, and *HLA-DRB1* class II genes were imputed using the R package HIBAG v1.4[121]. When available, array-specific European training sets were used. Alleles with an imputation posterior probability below 0.5 were considered to be undetermined, as recommended by HIBAG's developers.
In addition to the individuals excluded from the single variant analysis, individuals with more than 20% of missing genotypes were excluded from the HLA allele analysis. We analyzed a total of 34,067 cases and 54,361 controls. HLA-imputed genotypes were converted into PLINK binary format (by considering each HLA allele as a SNP) and then analyzed with SNPTEST v2.5.3. The same covariates as in the single variant analysis were used. HLA alleles with an effect size greater than 5 were excluded. The results were then fed into a fixed-effect meta-analysis with an inverse variance weighted approach, as implemented in METAL (v2011-03-25) software. Only alleles with a frequency above 1% (n=111) were considered. Adjusted *P* values were computed using the false discovery rate method and the p.adjust function in R (stats package v3.6.2), and then applied to the meta-analysis *P* values. The false discovery rate threshold was set to 0.05.
Three-locus HLA class I or class II haplotypes were determined using the haplo.em function in the R haplo.stats v1.8.6 package. Only individuals with non-missing genotypes (28,253 cases and 46,005 controls) were included in this analysis, and only haplotypes with a frequency above 1% were considered (representing 36 three-locus haplotypes). The haplotypes were analyzed in the same way as the alleles.

The analysis of the imputed HLA alleles revealed an association of three HLA class II risk alleles (DQA1*01:01, DQB1*05:01, and DRB1*01:01), three class II protective alleles (DQA1*03:01, DQB1*03:02, and DRB1*04:04), and two HLA class I risk alleles (A*02:01 and B*57:01) (Supplementary Table 8 and Supplementary Fig. 31). The associated HLA class II alleles form two distinct three-locus haplotypes that also showed an association with AD (the risk haplotype DQA1*01:01~DQB1*05:01~DRB1*01:01, odds ratio (OR) [95% confidence interval (CI)]=1.10 [1.06-1.14] and the protective haplotype DQA1*03:01~DQB1*03:02~DRB1*04:04, OR=0.87 [0.82-0.93]). A class I haplotype containing the risk allele B*57:01 also showed an association with AD (A*01:01~B*57:01~C*06:02, OR=1.15 [1.05-1.27]) (Supplementary Table 9 and Supplementary Fig. 32).

8. PheWAS

We searched for the effects of the 83 ADD genome-wide significant variants (Table 1) in the GWAS of neurodegenerative and AD-related diseases. The effects and significance of the following diseases were provided by the corresponding authors of the following studies: Creutzfeldt-Jakob disease (CJD)[122], Dementia with Lewy-bodies (DLB)[123], amyotrophic lateral sclerosis (ALS)[124], Frontotemporal dementia (FTD)[125], Parkinson's disease (PD)[126], ischemic brain infarcts (MRI defined)[127] and ischemic stroke (clinical)[128], white matter hyperintensities (WMH)[129]. Reported effects and significance were transformed into Z-scores. The Z-scores for the risk allele for ADD from the current manuscript are reported (Supplementary Table 10). We were able to look-up 74 out of 83 ADD associated variants present in more than half of the explored traits. For these variants 91% (539/592) of the variant-trait associations were present in the GWAS. In Supplementary Fig. 33, we show these associations for the allele that increases the risk of ADD and clustered the variants. There were more variant-disease associations (*P* value<0.05) that increased ADD risk and

associated with an increased disease risk (N=42), compared to associations opposite to the effect in ADD. Inspecting the clusters, there was a cluster of ADD associated variants that was also associated with increased risk of Parkinson's disease (*HLA*, *CLU*, *NME8*, *SPPL2A*, *KAT8*). A second cluster associated with increased risk of FTD, ALS, and PD (*MAPT*, *MAF*, *CTSB*, *GRN*). A third cluster of loci associated with increased risk of almost all associated traits (*TNIP1*, *PICALM*, *HS3ST5*, *PLCG2*, *PLEKHA1*, *PRKD3*, *SHARPIN*, and *DOC2A*).
Next we conducted a PheWAS using the 'phewas' function of the R-package 'ieugwasr' v0.1.5 (20201127)[130,131]. This function searches traits that associate with a list of variants, with a *P* value lower than a given value in all GWAS harmonized summary statistics in the MRC IEU OpenGWAS data infrastructure[131]. We chose to search for the 83 ADD-associated variants (Table 1) only for association with $P<1x10^{-5}$. This resulted in 1980 significant associations. We included GWAS with priority (priority=0), excluded eQTLs, excluded results from Japan biobank GWAS, excluded all GWAS with '_raw' in them (as they are the duplicate of those with 'irnt'), removed duplicates and we removed all GWAS of AD or history of AD. After this cleaning, 660 associations remain. We report these in Supplementary Tables 11 and 12. In total 27 variants had no other trait than ADD associated with them (or variants were not present in the public GWAS), 9 had 1 single trait, 17 had between 2 and 5, 24 had between 6 and 20 and 6 were very pleiotropic with over 21 traits associated. Interesting traits that appear more than once are IGF-1 (8 loci), systolic and diastolic blood pressure (8 loci), Aspartate aminotransferase (8 loci), Apoliprotein A (5 loci), Albumin (7 loci), Alkaline phosphatase (6 loci) and Cystatin C (6 loci).

9. GWAS signal colocalization analyses

For loci known to be associated with other neurodegenerative disorders, colocalization analyses were performed. For each locus, we performed the analysis in two steps: (1) a fine mapping analysis in Stage I to see whether the signal was due to only one causal variant and (2) a colocalization analysis to see if this signal was shared with the other neurodegenerative disorder.

Fine mapping
To assess whether the locus contained multiple independent signals, all the imputed variants having a MAF ≥0.005 and which were analyzed in the meta-analysis in ≥95% of the total number of samples were considered. Those variants were extracted from the EADB-TOPMed imputations, restricted to the samples included in the analysis, and converted into a hard-called genotypes PLINK v1.9 format using a probability cutoff of 0.8. A joint analysis using the Stage I results, along with the EADB-TOPMed extracted genotypes for the linkage disequilibrium estimation, was performed using the GCTA-cojo method[132] of the GCTA software v1.93.2beta. We used the stepwise model selection procedure of GCTA-cojo with a *P* value threshold of 10^{-5}.

Colocalization
The colocalization analysis between Alzheimer's disease and the other neurodegenerative trait was performed for each locus independently. For each region, only the common variants between the Stage I results and the summary statistics of the other trait were considered. When the summary statistics of the other trait was expressed on another build than GRCh38, the variant alleles and positions were converted according to the method previously described. Then, only variants having a MAF ≥0.005 in Stage I and analyzed in ≥95% of the total number of samples in both traits were extracted. The linkage disequilibrium correlation matrix was then computed on the genotype probabilities using LDstore v2.0[133]. The colocalization analysis was performed with the coloc R package v4.0-4 using the enumeration of configurations under a single causal variant assumption method[134]. The analysis was performed on regression coefficients and their variance using defaults priors

but also using a p12 prior (prior probability of any random variant in the region is associated with both traits) of $5x10^{-6}$ for sensitivity analysis.

Loci assessed.
Colocalization analyses were performed for 5 loci:

- *IDUA*: the summary statistics from a Parkinson's disease (PD) GWAS[126] was used and the region tested was restricted to chr4:643555-1243555 (GRCh38).
- *GRN*: we used the summary statistics from a Frontotemporal Dementia (FTD) GWAS[135] (all type of FTDs) and a Frontotemporal lobar degeneration with TAR DNA binding protein (TDP-43) inclusions (FTLD-TDP) GWAS[136]. The region tested was restricted to chr17:44102876-44602876 (GRCh38).
- *TMEM106B*: We used the same summary statistics as for the GRN locus. The region tested was restricted to chr7:11961934-12461934 (GRCh38).
- *CTSB*: the summary statistics from a PD GWAS[126] was used and the region tested was restricted to chr8:11594613-12094613 (GRCh38).
- *TNIP1*: the summary statistics from an Amyotrophic Lateral Sclerosis (ALS)[124] GWAS was used and the region tested was restricted to chr5:150802827-151302827 (GRCh38).

10. Pathway analyses

10.1. Gene sets

The assignments of Gene Ontology (GO) terms to human genes were obtained from the "gene2go" file (downloaded from the NCBI on March 11th, 2020). "Parent" GO terms were assigned to genes using the ontology file downloaded on the same date. GO terms were assigned to genes on the basis of experimental or curated evidence of a specific type, and so we excluded evidence codes IEA (electronic annotation), NAS (non-traceable author statement), and RCA (inferred from reviewed computational analysis). Pathways were downloaded from the Reactome website on April 26th, 2020. Biocarta, KEGG and Pathway Interaction Database (PID) pathways were downloaded from the Molecular Signatures Database v7.1 (March 2020). Our analysis was restricted to GO terms containing between 10 and 2000 genes. No size restrictions were placed on the other gene sets, since there were fewer of them. This resulted in a total of 10,271 gene sets for analysis.

10.2. Expression enrichment analysis in a mouse single-cell dataset

Mouse microglia expression showed the most significant correlation with GWAS signal (P=$2.61x10^{-4}$), however at a lesser extent than that observed with human microglia expression (Supplementary Table 17).
Of the 58 gene sets showing significant enrichment ($q \leq 0.05$) for a GWAS signal that could be tested for interaction with human microglial expression, 49 could also be tested for interaction with mouse microglial expression (Supplementary Table 18). None of these gene sets were significant ($q \leq 0.05$) after correction for all the gene sets tested. However, 39 of the 49 gene sets had the same direction of interaction as Av. Exp. for microglial expression in human brains. This number is significantly higher than would be expected by chance (P=$3.85x10^{-5}$ in a two-sided binomial test) - indicating overall similarities in the interaction effects of human and mouse microglia expression with genes in biological pathways of relevance to the AD genetic risk.

11. Functional interpretation of GWAS results

11.1. Gene prioritization methods

Description of genetic and transcriptomic datasets and cohorts

Two RNA-sequencing (RNA-seq) data sources were used in this study. First, through the Accelerating Medicines Partnership AD (AMP-AD) Knowledge Portal, we used uniformly processed AD-relevant brain RNA-seq datasets from the Mayo RNAseq Study (MayoRNAseq)[137], The Religious Orders Study and Memory and Aging Project (ROSMAP)[138,139], and The Mount Sinai Brain Bank study (MSBB)[140] available under consortium study "AMP-AD Cross-Study RNAseq Harmonization" (accessed in December 2019). Briefly, for MayoRNAseq and ROSMAP RNA-seq datasets, PolyA-selected libraries were sequenced on an Illumina HiSeq2000 platform (101 bp paired-end); for the MSBB RNA-seq dataset, rRNA-depleted libraries were sequenced on an Illumina HiSeq250 (100 bp single-end). Of note, among these three RNA-seq datasets, only ROSMAP dataset was stranded. Inclusion criteria were: (i) RIN value ≥ 5, (ii) availability of whole-genome sequencing (WGS) genotypes, and (iii) passing RNA-seq QC checks performed by the respective studies such as expression principle component analysis (samples should be positioned within the mean PC1 and/or PC2 ± 3xSD range), gene body coverage (a ratio of <3 between read number values at the 80th and 20th percentile), no sample swaps, concordance with genetic information and clinical metadata available. Additionally, in case multiple QCed RNA-seq samples were available on the same brain region of an individual, the RNA-seq sample selection was prioritized first based on the higher number of mapped reads and then based on the lower rRNA ratio.

Furthermore, through the AMP-AD Knowledge Portal, we accessed cohort-specific multi-sample WGS VCFs of MayoRNAseq, ROSMAP and MSBB studies that were generated by running GATK[141] (v3.4) HaplotypeCaller on 150 bp paired-end reads (sequenced on an Illumina HiSeq X) aligned to GRCh37 human reference genome. For each cohort-specific WGS VCF, we only selected autosomal variants that are passing Variant Quality Score Recalibration (VQSR) filters. Multiallelic variants were split and indels were left-aligned using BCFtools (v1.9) norm function. Moreover, we applied genotype level QC by assigning individual genotypes as missing if genotype quality (GQ) < 20 (using BCFtools) or if allele depth ratios of heterozygous genotypes exceeding 1:3 ratio, or if allele depth ratios of homozygous genotypes are within 1:9 ratio (using vcffilterjdk v9750c96[142] with a custom filtering java code that is available upon request). We then removed the variants missing in more than 85% of the cohort and/or the variants deviating from Hardy-Weinberg Equilibrium (HWE $P < 10^{-6}$). For sample QC, we excluded the samples with call rate < 95%. For each cohort, PLINK v1.90b4 was first used to select non-missing (variant missingness ≤ 0.02), common (MAF ≥ 1%), and LD-pruned (PLINK parameters: "--indep-pairwise 500kb 1 0.2") variants that are out of following long-range LD loci that are likely to confound genomic scans: LCT (2q21), HLA (including MHC), 8p23 & 17q21.31 inversions, and 24 other long-range LD regions[97]. Using these variants, we then estimated identity-by-descent (IBD) and heterozygosity ratios across the samples and excluded them based on relatedness (for pairs with PI-HAT > 0.2, we kept the sample with higher call rate or higher Genomic Quality Number [GQN]) and on excess heterozygosity (out of mean ± 3xSD range). Furthermore, using these LD-pruned high-quality common variants, for each cohort we calculated genetic principal components for subsequent downstream analyses including molecular quantitative trait locus (QTL) mapping.

Finally, as a result of the above genetic and transcriptomic QC and selection criteria, in our study we included a total of 1067 QCed unique WGS samples with 1552 QCed unique RNA-seq samples derived from six different brain regions in AD-relevant frontal and temporal lobes. These regions and their respective studies are as below: *n*=259 temporal cortex (TCX) RNA-seq samples from MayoRNAseq (31% AD, 31% progressive supranuclear palsy, 11% pathological aging diagnoses, 27% healthy controls; 51% female, mean age at death at

>80 years) study, *n*=560 dorsolateral prefrontal cortex (DLPFC) samples from ROSMAP (spectrum of clinical diagnosis at death: 32% no cognitive impairment [CI], 34% AD with no other CI, 34% other MCI and dementia types; 64% female, mean age at death at >87 years) study, and *n*=248 individuals in MSBB (spectrum of clinical dementia rating scores: 14% no dementia, 13% MCI, rest 73% are dementia at different stages) 66% female, mean age at death at >84 years) study with RNA-seq data available from frontal pole (*n*=207; Brodmann area [BA] 10), superior temporal gyrus (*n*=186; BA22), parahippocampal gyrus (*n*=162; BA36), and inferior frontal gyrus (*n*=178; BA44). We downloaded RNA-seq BAM files (aligned to genome indexes generated from human genome GRCh38 and GENCODE 24 with STAR RNA-seq aligner v2.5.1b[143]) for these selected QCed samples for further processing and data analyses.
Second, we used a cohort of 70 EADB Flanders-Belgian samples with lymphoblastoid cell line (LCL) RNA sequencing (RNA-seq) and TOPMed-imputed genetic information from EADB project available (referred to as "EADB Belgian LCL" cohort). EADB Belgian LCL cohort individuals consisted of 51 AD patients (49% female, mean age at blood sampling 75.5 ± 4.4 years), 17 healthy controls (47% women, mean age at blood sampling 74.9 ± 6.7 years), and 2 individuals with mild cognitive impairment (50% female, mean age at blood sampling 74 ± 1.4 years).
Lymphoblast cells were cultured at 37 °C with 5% CO2 in RPMI1640 medium supplemented with 15% fetal bovine serum, 1% Glutamax, 1% Sodium pyruvate and 1% Penicillin/Streptomycin. Total RNA was isolated from Epstein - Barr virus (EBV) immortalized lymphoblasts derived from whole blood lymphocytes for all included samples. RNA isolation was performed using 10^7 lymphoblast cells for each sample with the RNeasy mini kit (Qiagen Inc., Valencia, CA) according to manufacturer's protocol. Depletion of genomic DNA from the RNA sample was performed by turbo DNase treatment (Life Technologies, Carlsbad, CA, USA). RNA concentration was measured by DropSense 16 (Trinean, Gentbrugge, Belgium). RNA integrity number (RIN) values were obtained using the Agilent Technologies 2100 Bioanalyzer (Agilent Technologies, Santa Clara, CA, USA). RIN values were between 6.6 and 9.7 with an average value of 8.4 ± 0.8 Sequence libraries were constructed using the TruSeq Stranded mRNA Library Prep Kit (Illumina, San Diego, CA) using 1µg total RNA for each sample. Library preparation included RNA poly-A selection, RNA fragmentation, and random-hexamer-primed reverse transcription cDNA synthesis. Sequencing of prepared libraries was performed using an Illumina HiSeq2000 sequencer at the Macrogen NGS sequencing core, Seoul, Rep. of Korea, generating an average of $72 \times 10^6 \pm 6 \times 10^6$ 101 base-pair (bp) paired-end sequence reads. RNA-seq reads were mapped to genome indexes generated from human reference genome GRCh38 and GENCODE 32 using STAR RNA-seq aligner (v2.7.3a). These 70 LCL RNA-seq samples were paired with their corresponding TOPMed-imputed genetic data (genotyped on Illumina Global Screening Array platform within the framework of EADB project). The genetic principal components for the genetic data of these 70 individuals were computed using PLINK (v1.9)[108] on the same subset of high-quality LD-pruned variants used to calculate principal components in EADB GWAS.

Expression & splicing quantification and cis-e/sQTL mapping
To quantify gene expression for mapping expression QTLs (eQTLs) we followed the GTEx pipeline[144] with adaptations. First, we downloaded GENCODE GTF files (v24 for AMP-AD datasets and v32 for EADB Belgian LCL dataset), patched chromosome prefixes and created collapsed gene models with the Python script "collapse_annotation.py" that merged known transcripts into a single transcript model for each gene. Then RNASeQC (v2.3.5; "--legacy" parameter was used to enable compatibility with RNASeQC v1.1.9) was used to quantify expression on seven different RNA-seq datasets[145]. We added the "--unpaired" parameter for allowing quantification of single-end reads of MSBB RNA-seq datasets and "--stranded rf" parameter was added for stranded RNA-seq datasets (EADB Belgian LCL & ROSMAP). After combining transcript per million (TPM) counts and gene counts per sample in each RNA-seq dataset, we created normalized gene expression matrices using

"eqtl_prepare_expression.py" Python script that (i) first filtered the genes with <0.1 TPM & <6 reads in at least 20% of samples in each dataset, (ii) normalized expression values for each sample using trimmed mean of M values (TMM) to account for library size, and (iii) normalized gene expression across samples for each gene with inverse normal transformation[146]. We also calculated Probabilistic Estimation of Expression Residuals (PEER) factors for each dataset to account for potential technical confounders for gene expression[147]. The resulting BED files of normalized gene expression matrices were used for eQTL mapping.

For annotation-free splicing quantification in each RNA-seq dataset, we used Leafcutter (v0.2.9) and RegTools (v0.5.1)[148,149]. First, RegTools "junction extract" command was used to extract exon-exon junctions with minimum anchor length of 8 bp, minimum intron size of 50 bp and maximum intron size of $5x10^5$ bp; and "-s 1" parameter was added for stranded RNA-seq datasets (ROSMAP and EADB Belgian LCL). The resulting splice junction quantification files per sample in each dataset were clustered using "leafcutter_cluster_regtools.py" Python script, where we filtered out splicing clusters with less than 50 split reads. We then used "prepare_phenotype_table.py" Python script that (i) performed quantile normalization of the distribution of splice junction usage ratios per sample to a normal distribution, (ii) standardized these ratios across samples, and (iii) calculated splicing principal components (sPCs) for each dataset. The resulting BED files of normalized splice junction usage ratios were used for splicing QTL (sQTL) mapping.

For eQTL and sQTL mapping, an enhanced version of FastQTL (v2.184_gtex) was utilized[150]. We used only the common (MAF ≥1%) QCed WGS variants (for AMP-AD cohorts) or common imputed (imputation quality score $R^2 \geq 0.3$ in the EADB cohort that was used in GWAS) variants (for EADB Belgian LCL cohort). Prior to QTL mapping, we lifted-over QCed AMP-AD WGS variants from GRCh37 to GRCh38 genome build by using Picard (v2.22.6) LiftOver tool. All genetic variants were annotated with dbSNPv151 (GRCh38) using BCFtools annotate function. We considered the genetic variants within 1 million bases window from the transcription start sites (TSS) and splice sites, respectively for eQTL and sQTL mapping. Sex and first 3 genetic principal component covariates were included in the linear regression models both for eQTL and sQTL mapping. In addition to these, following the recommendations of GTEx pipeline based on the sample size, for eQTL mapping we also included first 15 PEER factors as covariates for EADB Belgian LCL, 30 PEER factors for MSBB, 45 PEER factors for MayoRNAseq, and 60 PEER factors for ROSMAP datasets; and for sQTL mapping we added first 15 sPCs for all datasets in the linear regression models. We performed linear regression with FastQTL by first (i) generating nominal *P* values for each tested variant-gene or tested variant-splice junction pair, then (ii) using Beta distribution-adjusted empirical *P* values (generated by adaptive permutations with "--permute 1000 10000") of the most significant variant-molecular phenotype pair to calculate *q*-values[151] for estimating false discovery rate (FDR), (iii) applying FDR ≤0.05 filter to identify genes or splice junctions with at least one significant e/sQTL ("eGene" and "sJunction"), and finally (iv) defining all eQTL variant and eGene and sQTL variant and sJunction pairs as significant if their nominal *P* passes the significance threshold defined for each eGene and sJunction by the permutation and FDR procedure.

Molecular QTL Catalogues

In this study, in addition to 7 eQTL catalogues and 7 sQTL catalogues that we prepared (as explained above); we also used various publically available molecular QTL catalogues to assess the potential downstream regulatory effects of GWAS variants on these molecular phenotypes, including expression, splicing, and methylation. Our main source for these was GTEx v8 where we utilized cis-e/sQTL catalogues for selected AD-relevant GTEx brain regions (hippocampus [*n*=165], frontal cortex [*n*=175], cortex [*n*=205] and anterior cingulate cortex [*n*=147; BA24]), LCL (*n*=147), and whole blood (*n*=670). As our molecular phenotype quantification and QTL mapping methodology highly overlap with the methodology used to construct these GTEx catalogues, we primarily used these GTEx v8 catalogues to replicate the effects of significant cis-e/sQTL variants in a general population context (compared to

our cis-e/sQTL catalogues that are derived from the AD cohorts). Furthermore, we incorporated the results from Microglia Genomics Atlas (MiGA) that is a recently established microglial expression and splicing regulation dataset[152]. We retained significant eQTLs and sQTLs (after a similar permutation and FDR [≤0.05] procedure described above for the other e/sQTL catalogues) in four different brain regions: medial frontal gyrus (MFG [BA9], *n*=63), superior temporal gyrus (STG [BA22], *n*=55), subventricular zone (SVZ, *n*=53) and thalamus (THA, *n*=45). Because the sample size is rather low compared to other bulk RNA-seq based e/sQTL catalogues, as also recommended by the authors, we additionally used the meta-analysis results based on RE2 random effects model as implemented in METASOFT[153] that combined four brain regions assessed (*n*=216). To define the significance of meta-analysis microglial e/sQTL associations, the stringent Bonferroni-corrected *P* value thresholds based on the number of independent tests were used ($\leq 6.58 \times 10^{-10}$ for MiGA Meta eQTLs and $\leq 1.79 \times 10^{-10}$ for MiGA Meta sQTLs).

Moreover, we used the blood cis-eQTL catalogue from eQTLGen project[154] (December 2019 release) that is the largest eQTL catalogue as it analyzed the regulatory effects of variants in over 30K individuals. Furthermore, macrophage[155,156] and monocyte[157–160] (CD14+ or CD16+) cis-eQTLs uniformly prepared by eQTL Catalogue database[161] (release 3 - October 2020) were also used in our analyses, where we used a nominal *P* value threshold of $\leq 10^{-5}$ to define significance for the associations. We mainly prioritized the eQTL effects in the naïve state macrophages and monocytes over the effects in stimulated macrophages and monocytes that were stimulated with various stimulants such as Influenza, Listeria, Salmonella, IFNg (Interferon gamma), LPS (Lipopolysaccharides), Pam3CSK4 (Pam3CysSerLys4), and R848 (Resiquimod) with different lengths of time. Finally, in addition to these e/sQTL catalogues, we also utilized brain methylation QTL (mQTL; *n*=468) and histone acetylation (haQTL; *n*=433) catalogues available at Brain xQTL serve[162] and that were mapped by integrating imputed genotypes with H3K9Ac ChIP-seq data and DNA methylation array data of DLPFC samples from ROSMAP project[163,164].

e/sQTL colocalization and e/sTWAS

The genetic colocalization between EADB GWAS and e/sQTL signals were investigated using coloc (v4.0.4)[134] in 12 eQTL and 12 sQTL catalogues for which we have full summary statistics available (AMP-AD, EADB Belgian LCL, and MiGA). We uniformly annotated and matched the variants in the EADB GWAS summary statistics and e/sQTL summary statistics files with the rsIDs from dbSNPv151 (GRCh38), and if no rsID available, with the "CHR_POS_REF_ALT" format using BCFtools annotate function. We selected a list of eGenes and sJunctions whose significant e/sQTLs (in at least one e/sQTL catalogue assessed) are associated with ADD risk in EADB Stage I GWAS minimally at a suggestive significant level (*P* value $\leq 10^{-5}$). We then ran "coloc.abf" (Bayesian colocalization analysis using default priors) on each selected eGene and sJunction considering all tested variants within 1 Mb of the TSS or splice site (except for MiGA sQTLs that used a 100kb window). The results showed the calculated posterior probabilities (PP) for 5 different hypotheses between two signals compared: H0 (no causal variant for both traits), H1 (causal variant only for EADB GWAS), H2 (causal variant only for e/sQTL), H3 (two distinct causal variants) and H4 (common causal variant shared between EADB GWAS and e/sQTL catalogue). We considered a signal as colocalized in EADB GWAS and e/sQTL catalogue if coloc PP4 is at least 70%.

We investigated the association between predicted expression and splicing and ADD risk by performing a transcriptome-wide association study (TWAS) using EADB summary statistics and expression and splicing reference panels. First, we used FUSION pipeline[165] to create custom expression and splicing reference panels based on AMP-AD and EADB Belgian LCL e/sQTL catalogues generated. To this end, we supplied e/sQTL mapping input files to "FUSION.compute_weights.R" R script for expression and splice weight calculation per study. These prediction weights were computed using BLUP, LASSO, top SNP, and Elastic Net models. We used "--hsq_p 0.05" parameter for heritability *P* to calculate functional

weights for genes and splice junctions only if they are significantly heritable features. We generated a custom LD reference data (annotated with dbSNPv151 and excluding variants with HWE $P < 10^{-6}$) by analyzing the phased biallelic SNV and INDEL genetic variants called *de novo* on GRCh38 (for selected *n*=404 unrelated Non-Finnish European (NFE) samples) from the 1000 Genomes (1KG) project[166,167]. We restricted TWAS functional weight modelling by only using the variants that are both found in this LD reference data and the QCed genetic data of the cohort. We then ran TWAS by integrating EADB Stage I GWAS summary statistics, custom LD reference data, and custom expression (*n*=7) and splicing (*n*=7) reference panels using "FUSION.assoc_test.R" R script. The TWAS significance thresholds were defined per study based on Bonferroni correction on transcriptome-wide number of tested features (Supplementary Table 21).

Second, we ran additional expression and splicing TWAS of ADD using precalculated MASHR-based expression and splicing prediction models for GTEx v8 datasets[168] using S-PrediXcan[169,170] implemented in MetaXcan v0.6.12 tools[169]. We ran S-PrediXcan (with non-default parameters "--keep_non_rsid --model_db_snp_key varID --additional_output –throw) using EADB summary statistics and MASHR model and covariance files for the same GTEx brain regions, cells and tissues chosen for e/sQTL studies: hippocampus, frontal cortex, cortex, BA24, LCL and whole blood. The significance thresholds of S-PrediXcan results were defined per study based on Bonferroni correction on transcriptome-wide number of tested features (Supplementary Table 21).

Finally, we used FOCUS (v0.7) for fine-mapping of expression TWAS results[171]. We imported both FUSION and PrediXcan based expression weights per dataset and ran fine-mapping of TWAS associations in regions of interest (based on 1 Mb extended region of lead variants in each novel EADB GWAS loci) to calculate posterior inclusion probabilities (PIPs) for each association which were later used to define the 90% credible sets of genes (which we accepted as fine-mapped TWAS associations).

Long-read cDNA Sequencing

For validating two AD-associated splice junction clusters that contained complex novel cryptic splicing events within *TSPAN14*, we designed an amplicon-based long-read single-molecule nanopore sequencing (Oxford Nanopore Technologies) experiment[172] on cDNA derived from hippocampus, frontal cortex BA10 and LCL of AD patients and cognitively healthy individuals of our Flanders-Belgian cohort (Fig. 4). We first designed the following two amplicons with Primer3[8] (v4.1)[173]: long range cDNA amplicon 1 (forward primer: 5`-CTCTAACGCCAAGGTCAGCT-3`, reverse primer: 5`-CTCCCTCAACTCTGCTCCTC-3`) and long range cDNA amplicon 2 (the same forward primer, reverse primer: 5`-CTGACATGGCCAAGGAGTG-3`). All primers contained tag sequences for nanopore sequencing. All PCR amplifications were performed with 35 cycles using Platinum Taq DNA polymerase (Thermo Fisher). After the reactions, excess primers and nucleotides were cleaned with ExoSAP-IT (Thermo Fisher). Amplicon 1 was generated on *n*=60 LCL (44 AD patients, 15 healthy individuals, 1 MCI patient, 48% female, mean age at blood sampling 75.3 ± 5.3 years), *n*=18 frontal cortex BA10 (50% AD patient, 44% female, mean age at death 79 ± 7.4 years), and *n*=16 hippocampal (50% AD patient, 44% female, mean age at death 79.5 ± 8.3 years) cDNA samples; and barcoded with the PCR Barcoding Expansion 1-96 kit (Oxford Nanopore Technologies) using 20 amplification cycles on a 1/200 diluted template. Amplicon 2 was only run on a pooled unbarcoded sample of 14 LCL cDNA samples (8 AD patients, 5 healthy individuals, 1 MCI patient, 57% female, mean age at blood sampling 74.8 ± 4.2 years) as we only identified these splicing events in LCL. After purification with Agencourt AMPure XP beads (Beckman Coulter, Brea, CA, USA) and concentration measurement with Qubit (Thermo Fisher), amplicons were pooled equimolarly. The sequencing library was prepared as previously described[174]. SQK-LSK109 chemistry and FLO-FLG001 Flongle flow cell adapted into MinION platform were used for sequencing at the VIB-UAntwerp Center for Molecular Neurology, Antwerp, Belgium.

Base calling of the raw reads was performed with ONT basecaller Guppy (v3.2.4) on the Promethion compute device in Antwerp. After demultiplexing of basecalled FASTQ reads

with qcat (v1.0.1) alignment of demultiplexed reads to GRCh38 reference genome was performed with minimap2 (v2.17) with parameters "-L -ax splice"[175]. NanoStat (v1.1.2) was used to calculate sequencing statistics which indicated an output of 311 million sequenced bases and 387 thousand reads with a median read length of 903 bp[176]. The resulting number of successfully sequenced cDNA samples were: 59 LCL cDNA, 18 frontal cortex BA10 cDNA, and 16 hippocampal cDNA samples for Amplicon 1; and a pooled LCL cDNA sample for Amplicon 2. We removed secondary alignments and supplementary alignments from the aligned reads using Samtools (v1.9). The aligned reads whose lengths of clipped bases were over 20% of their actual length were excluded using SamJdk v9750c96[142]. For extracting Amplicon 2 specific aligned reads from the unbarcoded sample, we extracted the aligned reads containing unique reverse primer for this amplicon in the 3' end of the aligned read. The AD-associated cryptic splicing events and cryptic exons in *TSPAN14* were also confirmed by visualization of aligned reads on Integrative Genomics Viewer (IGV; v2.4.17)[177]. We merged the aligned reads of Amplicon 1 and Amplicon 2 based on LCL, frontal cortex and hippocampus categories and then ran mosdepth (v0.2.9) to generate the cumulative coverage tracks of each amplicon per cDNA type for further data visualization[178].

Genetically driven DNA methylation scan (MetaMeth)

We tested for association between ADD and genetically driven DNA methylation (DNAm) using the procedures proposed previously by Freytag et al.[179] and Barbeira et al.[169]. The approximate association statistics between the methylation of 5′-C-phosphate- G-3′ (CpG) sites and ADD were computed with the function *MetaMethScan* from the *EstiMeth* (v1.1) software. The approach was applied to the EADB Stage I summary statistics, using the default DNAm estimation models and variant covariance structure, which is inferred from the 1000G European population. We performed a systematic search of CpG association signals within a region of 1 Mb around the lead variants. We considered as significant CpG sites with *P* value < 0.05, after Bonferroni correction for multiple testing for 77,881 features. Each CpG was paired with its annotated gene(s) and respective positional annotation of CpGs. Significant CpG sites were annotated by their percentile and direction of blood-brain methylation correlation estimates across three brain regions that were obtained from BECon[180].

PWAS and pQTL colocalization

In order to gain insight into how ADD risk variants might regulate or impact protein expression and to add an additional proteome layer to our QTL-GWAS integration based gene prioritization pipeline, we used the results of a proteome-wide association study (PWAS), protein expression QTL (pQTL) mapping, and pQTL colocalization study recently performed by Wingo et al., 2021[181]. Wingo and colleagues have mapped pQTLs in dorsolateral prefrontal cortex samples from ROSMAP (*n*=376), investigated genetic colocalization between these pQTL signals and Jansen et al. AD GWAS[28], and run a PWAS using the same AD GWAS summary statistics[28]. In addition to this discovery PWAS, a confirmation PWAS was performed using dorsolateral prefrontal cortex samples from Banner dataset (*n*=152)[181].

We queried the lead variants in the newly identified loci in ROSMAP DLPFC pQTL catalogue and found no overlap between these lead variants and the significant pQTLs (at a Bonferroni-corrected $P \leq 5.48\times10^{-8}$ level). However, when querying the PWAS and pQTL coloc results from Wingo et al.[181], we identified FDR-significant PWAS associations for four candidate genes within newly identified loci in this study. We further used this information on four genes (i.e. *ICA1L*, *PLEKHA1*, *CTSH*, and *DOC2A*) for gene prioritization in the respective loci.

Variant annotation

For gene prioritization in each new locus, we used annotation information for the lead variants. These include (i) rare (MAF < 1%) association signal for the gene, (ii) protein-altering effect of the lead variant for the gene, and (iii) the nearest protein-coding gene to the

lead variant. We annotated the CADD (v1.6) PHRED scores[182] for the protein-altering lead variants.

<u>High-content siRNA screening for APP metabolism</u>

The SiGENOME SMARTpool siRNA library (targeting the 18,107 genes of the whole human genome) was screened in HEK293 cells stably over-expressing a mCherry-APP^{695wt}-YFP[183]. Briefly, 72 hours after transfection, cells were fixed for image acquisition with InCell Analyser 6000 high-resolution automated confocal microscope. Customized image analysis software (Columbus 2.7, PerkinElmer) was used for the image analysis and the quantification. The mean fluorescence intensity of each mCherry and YFP signals in the cytoplasm were quantified. The mean fluorescence intensity of each signals were then normalized to the fold change based on the non-targeting siRNA in the same plate. Because YFP fluorescence is detected weakly, due to its rapid turnover at the membrane[184], we decided to focus on the mCherry signal as the main read-out. An APP metabolism signal was defined when the log2 fold-change of the mCherry signal was above 1 in absolute value.

11.2. Gene prioritization results

The gene prioritization strategy is described in the Methods. The results for the prioritized genes (as Tier 1 and Tier 2) are summarized in Fig. 2A. A results summary for all candidate genes (i.e. the genes with a gene prioritization score >0) in the new loci is provided in Supplementary Table 20 and Supplementary Figure 34, with a full description of the results in Supplementary Tables 21-30 and Supplementary Fig. 35-45.

<u>Note on prioritisation of rare variant loci:</u> As the molecular QTL studies typically test common (MAF > 1%) variants for molecular phenotype associations, for analysis of rare association signals they have limited utility for gene prioritization. For prioritization of such rare variant loci, the variation annotation domain is the decisive domain. There are multiple studies to give extra weight for the closest gene when the lead variant is a rare variant. First, the frequently used gene-based rare variant association testing model, sequence kernel association test (SKAT[185]) and its extension SKAT-O[186], recommend upweighting of the effect of rare variants (typically at MAF < 1%, and with beta weights of 1 for common and 25 for rare variants) based on simulation and real-data analyses. Second, another study showed that the candidate large-effect non-coding rare variants are enriched near transcription start site (TSS) of their cis-eQTL genes; and importantly, their effect sizes decrease as a function of distance to TSS[187], that was also later demonstrated by other following studies conducted using GTEx data[188–190]. Moreover, when considering the high confidence lead variant - causal gene relationships in the GWAS Gold Standards repository (https://github.com/opentargets/genetics-gold-standards, curated by the Open Targets Platform), we found that the rare noncoding lead variants are more accurate in predicting the causal gene as the nearest annotated gene (72.7%), compared to common noncoding lead variants (62.6%). Of note, rare protein-altering variants were fully accurate to identify the high confidence causal genes, and this was true for 97.4% of the cases for the common protein-altering variant, an observation that also supported our considerably higher weight for protein-altering common and rare variants.

As a result of our gene prioritization methodology, in *n*=41 new loci (excluding IGH gene cluster locus [L27]) we prioritized 31 genes as Tier 1 genes and 24 genes as Tier 2 genes. A majority (25/31) of Tier 1 genes were single prioritized genes in their respective loci, and six of them were found in loci with other Tier 2 gene(s). These six loci contained seven Tier 2 genes, and in addition, we identified five loci with a single prioritized Tier 2 gene and five loci with multiple prioritized Tier 2 genes. Of note, of 31 Tier 1 genes, 24 genes (77%) were the nearest protein-coding gene.

Below we categorized each locus based on the gene prioritization results and further discuss those. For each novel locus, the prioritized gene information is provided, and the locus index number and gene prioritization score for the respective prioritized genes were indicated in parentheses.

<u>Loci with a single Tier 1 prioritized gene</u>

***SORT1* locus (L1). Tier 1: *SORT1* (Score = 20).** The lead variant is a rare missense variant (p.Lys302Glu, MAF = 0.5%, CADD = 22.8) located in the β-propeller domain of SORT1 involved in ligand binding. Of interest, it is in the predicted ligand binding site for GRN[191] (L36). This same rare variant was previously found to be enriched in European patients with FTD[192]. Of note, *SORT1* was the only candidate gene in the locus.

***NCK2 locus* (L4). Tier 1: *NCK2* (Score = 5).** In this locus the only candidate gene was *NCK2*, which collected prioritization points from the variant annotation domain as the intronic (within *NCK2*) lead variant is a rare variant (MAF = 0.5%).

***WDR12* locus (L5). Tier 1: *ICA1L* (Score = 46)**. In this locus, the lead variant is found in 3' UTR of *WDR12* however it is also ~7kb upstream from the transcription start site of *ICA1L*. eQTL mapping, eQTL coloc, and eTWAS results output multiple genes in the locus whose predicted upregulation are seemingly co-regulated by the ADD-associated LD block in the locus, meanwhile fine-mapping of eTWAS results prioritizes *ICA1L*, *WDR12*, *CARF* and *NBEAL1* in the different brain regions assessed (Supplementary Tables 22, 24, 26 and Supplementary Fig. 36a, 39, 41). Two of these genes, *ICA1L* and *CARF*, were additionally prioritized because of the multiple AD-related splicing modulations in sTWAS, meanwhile we also observed the lead variant as a significant sQTL for chr2:202819899-202828848 splice junction in *ICA1L* in TCX and DLPFC, whose sQTL signal also colocalized with ADD association signal (Supplementary Tables 23, 25, 27 and Supplementary Fig. 37, 40, 42). This splice junction is specific to known protein-coding short transcript of *ICA1L* (ENST00000418208.5) that is about 7 times smaller than the canonical isoform, and our data shows an increased ADD risk correlated with the increased predicted preference of this junction (Supplementary Table 27 and Supplementary Fig. 42). Furthermore, we identified that *ICA1L* modulated APP metabolism (Supplementary Fig. 45), and interestingly it is a paralog of another gene in our study, *ICA1* (in L15), that was also prioritized due to its effect on APP metabolism. Finally, in the recent PWAS of ADD, *ICA1L* was one of the significant hits[181], where predicted decreased *ICA1L* protein expression in DLPFC correlated with higher ADD risk (Supplementary Table 30 and Supplementary Fig. 43). Of note, this PWAS observation in brain is in line with the effect observed in the functional assay (i.e. silencing of *ICA1L* is associated with an increase in APP metabolism) and with the above mentioned sTWAS effect direction, however, it contradicts the expected effect direction from fine-mapped eTWAS hit in the same reference dataset (ROSMAP DLPFC). Nevertheless, it is important to mention that the same eTWAS association has an opposite effect compared to brain in GTEx LCL, where predicted decreased expression of *ICA1L* increases ADD risk. Taken together, in locus 5, our gene prioritization strategy categorized *ICA1L* as a Tier 1 gene.

***MME* locus (L6). Tier 1: *MME* (Score = 20)**. We observed two independent lead variants in L6, and one of them (rs61762319) was a predicted deleterious missense variant (p.Met8Val, CADD = 23.1). Interestingly, we identified that the other independent lead variant in *MME*, rs16824536, is a significant mQTL in DLPFC where the risk allele is significantly associated with higher methylated levels of cg25511593 CpG site within *MME* (Supplementary Table 29 and Supplementary Fig. 38a). This might suggest a complementary mechanism of action for both lead variants in a way that both predicted deleterious missense allele of rs61762319 and higher methylation levels (therefore expected potentially lower expression of *MME*) controlled by the risk allele of rs16824536 work

potentially in the same direction for the disease risk. Of note, *MME* was the only candidate gene in the locus.

***RHOH* locus (L8). Tier 1: *RHOH* (Score = 7).** The nearest gene *RHOH* was prioritized as Tier 1 in this locus as the risk allele of the lead variant is significantly associated with decreased *RHOH* expression in TCX and as we observed colocalization of *RHOH* eQTL signals in TCX with ADD genetic association signal (PP4 = 98%) (Supplementary Tables 22, 24 and Supplementary Fig. 36, 39). Of note, *RHOH* expression in brain is very strongly enriched (93%) in microglia (Fig. 2A and Supplementary Table 20).

***ANKH* locus (L9). Tier 1: *OTULIN* (Score = 15).** Our methodology could prioritize *OTULIN* in this locus with a high confidence (Tier 1) even though it is not the nearest protein-coding gene, because all the results of our assessments in this locus pointed out at *OTULIN* exclusively, including significant eQTL - lead variant overlap, eQTL coloc, and fine-mapped eTWAS results in brain (Supplementary Tables 22, 24, 26 and Supplementary Fig. 36a, 39, 41); all hinting at a correlation between increased ADD risk and predicted higher expression of *OTULIN*.

***TNIP1* locus (L11). Tier 1: *TNIP1* (4).** We identified that the risk allele of the lead variant (that is intronic in *TNIP1*) was significantly associated with increased *TNIP1* expression in DLPFC. (Supplementary Table 22 and Supplementary Fig. 36a). There were no other candidate genes in the locus, and *TNIP1* also collected weighted scores for being the nearest protein-coding gene to the signal. Therefore, in this locus our strategy prioritized *TNIP1* as a Tier 1 gene.

***ICA1* locus (L15). Tier 1: *ICA1* (Score = 22).** The evidence that supported the prioritization of this gene as a Tier 1 gene in L15 came from its association with APP metabolism modulation (Supplementary Fig. 45), along with being the nearest gene to the association signal. Interestingly, this gene is a paralog of another Tier 1 prioritized gene, *ICA1L* (in L5; discussed in the above section); and silencing of these two paralog genes caused opposite effect directions on APP metabolism functional screen (Supplementary Fig. 45).

***TMEM106B* locus (L16). Tier 1: *TMEM106B* (Score = 13).** The risk allele of the lead variant in L16 was significantly associated with decreased *TMEM106B* expression in cortex (GTEx) and in naïve and stimulated monocytes (Fairfax dataset) (Supplementary Table 22 and Supplementary Fig. 36). Furthermore, the risk allele was also associated with increased methylation levels of cg09613507 intragenic CpG site in brain (Supplementary Table 29 and Supplementary Fig. 38a); however, when the effect of all variants were considered, MetaMeth implicated this CpG site as potentially protective for ADD when predicted methylation levels are increased in blood (Supplementary Table 28 and Supplementary Fig. 38b). Moreover, sTWAS implicated the increased predicted preference for chr7:12224385-12229679 splice junction (specifically present in longer *TMEM106B* transcripts including the canonical transcript) in cortex as protective for ADD risk (Supplementary Table 27 and Supplementary Fig. 42). All these associations contributed into Tier 1 prioritization of *TMEM106B* in this locus. Of note, *TMEM106B* was the only candidate gene in the locus.

***JAZF1* locus (L17). Tier 1: *JAZF1* (Score = 7.5).** The prioritization of this gene as a Tier 1 gene was mainly driven by the colocalization of its eQTL signals in microglia with ADD genetic association signal. We observed eQTL coloc hits in MFG (PP4 = 77%), STG (PP4 = 89%), and THA (PP4 = 71%) (Supplementary Table 24 and Supplementary Fig. 39). Single-cell gene expression data supported microglia-specific eQTL colocalization results for this gene, as *JAZF1* is primarily expressed in microglia (45%, Fig. 2A and Supplementary Table 20). Finally, MetaMeth results suggested that increased methylation in two intragenic CpGs can be protective for ADD (Supplementary Table 28 and Supplementary Fig. 38b).

***SEC61G* locus (L18). Tier 1: *EGFR* (Score = 12).** In the intergenic signal of L18, *EGFR* is the Tier 1 prioritized gene because an intergenic, distant, and low-frequency cis-eQTL signal for *EGFR* which colocalizes with the ADD association signal (near ~1 coloc PP4s), and its fine-mapped eTWAS hits (with FOCUS PIP values of ~1) associate predicted decreased *EGFR* expression with decreased ADD risk both in TCX and DLPFC (see Fig 4; Supplementary Tables 22, 24, 26 and Supplementary Fig. 36a, 39, 41).

***CTSB* locus (L19). Tier 1: *CTSB* (Score = 4).** In this locus *CTSB* (the nearest gene) was prioritized as Tier 1 gene. The lead variant is a low-frequency 3' UTR variant in *CTSB*, and the risk allele of the lead variant was found to be associated with decreased CTSB expression in DLPFC (Supplementary Table 22 and Supplementary Fig. 36a). Of note, *CTSB* is primarily expressed in microglia (52%, see Fig. 2A and Supplementary Table 20).

***SHARPIN* locus (L20). Tier 1: *SHARPIN* (Score = 25).** In this locus the lead variant (rs34173062) is a missense variant (*SHARPIN* p.Ser17Phe) with a CADD score of 19. We found additional evidence that lower *SHARPIN* expression associated with higher ADD risk (fine-mapped eTWAS hit in GTEx brain BA24 region; Supplementary Table 26 and Supplementary Fig. 41) and that ADD risk is associated with the regulation of splicing of the first two exons of *SHARPIN* (sTWAS hits in AD-relevant GTEx brain regions; Supplementary Table 27 and Supplementary Fig. 42) that also contained rs34173062 missense lead variant.

***ABCA1* locus (L21). Tier 1: *ABCA1* (Score = 5).** The nearest gene *ABCA1* was prioritized as Tier 1 gene in this locus, because the risk allele of the lead variant in the locus was found to be associated with decreased methylation levels in brain for the CpG site cg14313833 that is 77 bp upstream of the TSS of *ABCA1* (Supplementary Table 29 and Supplementary Fig. 38a). The bibliographical data also strongly support implication of *ABCA1* in AD as ABCA1 overexpression reduces amyloid deposition in an AD-like mouse model[193] and the burden of rare variants in this gene was associated with AD risk[194]. Of note, *ABCA1* is primarily expressed in astrocytes (46%, see Fig. 2A and Supplementary Table 20).

***TSPAN14* locus (L23). Tier 1: *TSPAN14* (Score = 33).** *TSPAN14* was identified as the Tier 1 gene as it exhibited numerous AD-related expression, methylation, and splicing modulations. The protective and minor allele of the lead variant is associated with decreased *TSPAN14* expression and increased preference for cryptic splice junctions (within ADAM10-interacting domain) that we identified and experimentally confirmed by long-read single-molecule sequencing in brain and LCL samples (see Fig. 4; Supplementary Tables 22-28 and Supplementary Fig. 36-42, 44c). TSPAN14 was reported to regulate the trafficking and the function of the metalloprotease ADAM10[195].

***BLNK* locus (L24). Tier 1: *BLNK* (Score = 16).** In this locus *BLNK* was prioritized as the Tier 1 gene, mainly because of the presence of significant and fine-mapped eTWAS results in brain (FOCUS PIP values of 0.96 to 0.98) with considerable eQTL colocalization in DLPFC (PP4 = 97%) and importantly in microglia (MiGA Meta PP4 = 98%), predicting a protective effect of decreased *BLNK* expression (Supplementary Tables 22, 24, 26 and Supplementary Fig. 36, 39, 41). Of note, *BLNK* is primarily and almost exclusively expressed in microglia (98%, Fig. 2A and Supplementary Table 20).

***PLEKHA1* locus (L25). Tier 1: *PLEKHA1* (Score = 18).** The Tier 1 gene *PLEKHA1* was supported by eQTL overlap with the lead variant in DLPFC and microglia (the protective allele is associated with decreased expression), sQTL overlap (the protective allele is associated with increased preference) and sQTL coloc hit in DLPFC (PP4 = 82%) for its splice junction of chr10:122428316-122429624 that is related to alternative splicing of the last coding exon, and most importantly substantial eQTL coloc hits in microglia (PP4s of 88%, 92%, and 97% respectively in MFG, SVZ, and meta-analysis) (Supplementary Tables 22-25 and Supplementary Fig. 36, 37, 39, 10). Finally, in the PWAS of ADD, *PLEKHA1* was

one of the significant hits[181], where predicted decreased *PLEKHA1* protein expression in DLPFC correlated with decreased ADD risk (Supplementary Table 30 and Supplementary Fig. 43), which agrees with the effect direction of transcriptional effects of ADD-associated genetic variation in the locus we explained above.

***CTSH* locus (L29). Tier 1: *CTSH* (Score = 36.5).** In this locus the nearest (and the only candidate) gene *CTSH* was prioritized as Tier 1 gene as it was implicated by the numerous AD-driven modulations we observed, including i) the overlap of the lead variant with brain, macrophage, and monocyte eQTLs with considerably large effect sizes (where the protective allele was associated with decreased *CTSH* expression) in most of the eQTL catalogues investigated, ii) the overlap of the lead variant with sQTLs in brain controlling *CTSH* splicing, and consequently high coloc PP4 values in these tissues (and in microglia additionally) both for eQTL and sQTL coloc, iii) the fine-mapped eTWAS hits (agreeing on the effect direction of the protective allele) and iv) the significant sTWAS hits for splice junctions in *CTSH* (Supplementary Tables 22-27 and Supplementary Fig. 36, 37, 39-42). In addition, in the recent PWAS of ADD, *CTSH* was one of the significant hits together with a pQTL colocalization[181] where predicted decreased *CTSH* protein expression in DLPFC also correlated with decreased ADD risk (Supplementary Table 30 and Supplementary Fig. 43).

***DOC2A* locus (L30). Tier 1: *DOC2A* (Score = 65.5).** *DOC2A* locus was one of the most complex new locus we encountered as (i) it is among the most gene-dense loci, (ii) our methodology listed numerous genes in the locus with differing association patterns in different tissues and regions investigated, and additionally (iii) it is partially overlapping with BCKDK locus (that is ~1 Mb downstream) when 1 Mb extended coordinates are considered for analyses. While statistical fine-mapping of eTWAS and sTWAS prioritized numerous genes in different tissues (Supplementary Tables 26-27 and Supplementary Fig. 41, 42), genetic driven methylation signals pointed mostly to *DOC2A* as 5 CpGs (3 found in promoter region: cg27151362, cg03890691, cg07041748) have positive blood-brain methylation correlations at least in >50% percentile and point at a protective effect of increased methylation for ADD risk (Supplementary Table 28 and Supplementary Fig. 38b). Altogether, increased DOC2A expression, decreased methylation (therefore potentially increased DOC2A expression), and increased preference for canonical splicing were all predicted to be associated with increased ADD risk. Moreover, the lead variant was a missense protective variant in *DOC2A* (p.Gly48Ser, CADD = 10). *DOC2A* was also found to modulate APP metabolism (Supplementary Fig. 45). However, in the PWAS of ADD, *DOC2A* was one of the significant hits together with a pQTL colocalization[181], but predicted decreased *DOC2A* protein expression in DLPFC correlated with increased ADD risk (Supplementary Table 30 and Supplementary Fig. 43). With these information, our methodology prioritized *DOC2A* as a Tier 1 gene in this locus. However, one should be careful with interpretation of this complex locus 30 given that several other genes had high posterior probabilities of explaining the eTWAS association signal in the fine-mapping analysis of certain brain regions (Supplementary Fig. 41,44d), suggesting that multiple risk genes can be potential candidates in this locus, including *PPP4C*, *YPEL3*, and *INO80E* (prioritization scores of 30, 24.5, and 22 respectively) (Supplementary Tables 26-27 and Supplementary Fig. 41,42).

***MAF* locus (L31). Tier 1: *MAF* (Score = 5).** In this single candidate locus, the risk allele of the intergenic lead variant (nearest to *MAF*) was associated with decreased histone acetylation (H3K9Ac) levels (indicative for active chromatin) at an intergenic site (~14kb downstream of *MAF*) in brain (Supplementary Table 29 and Supplementary Fig. 38a). We found that this associated H3K9Ac peak was in line with the H3K27ac signature (measured at single-cell level in brain) in microglia and overlapped with a microglia-specific enhancer[196]. Furthermore, the intergenic lead variant rs450674 itself also overlapped with a microglia-specific enhancer. Of note, we observed that *MAF* was primarily expressed in microglia (56%, Fig. 2A and Supplementary Table 20).

***GRN* locus (L36). Tier 1: *GRN* (Score = 14).** *GRN* was the Tier 1 prioritized gene in L36. We observed a close to 100% probability for *GRN* eQTL signal colocalization with the genetic association signal in 5 out of 6 brain regions investigated, and fine-mapping of eTWAS strongly pointed towards *GRN* as the gene explaining the GWAS signal at this locus (FOCUS PIP=1 in 5 frontal and 1 temporal lobe prediction panels tested), suggesting a correlation between predicted lower *GRN* expression and increased ADD risk (Supplementary Tables 22, 24, 26 and Supplementary Fig. 36a, 39, 41, 44a). Bibliographical data were also clearly in favor of *GRN*: beyond its implication in frontotemporal dementia, *GRN* deficiency significantly reduces diffuse Aβ plaque growth in an AD-like mouse model and it has been proposed that this protective effect is due, in part, to enhanced microglial Aβ phagocytosis[197]. Moreover, rare variants in *GRN* have previously been associated with AD[198,199].

***SIGLEC11* locus (L38) Tier 1: *SIGLEC11* (Score = 15.5).** In locus 38, *SIGLEC11* was the Tier 1 prioritized gene. *SIGLEC11* has very high colocalization probabilities (coloc PP4 93% to 98%) between the GWAS signal in the locus and its brain eQTL signal in six different catalogues investigated and also in microglia (PP4 = 98%), hinting at a possible association between ADD risk and higher SIGLEC11 expression (Supplementary Table 24 and Supplementary Fig. 39). Even though eTWAS signals failed to pass stringent Bonferroni-corrected significance thresholds, statistical fine-mapping mapped *SIGLEC11* to the 90% credible set of plausible candidate genes (with FOCUS PIP values 0.39 to 0.64) in this genomic locus. We also observed the lead variant as a significant eQTL in many brain regions, microglia, macrophages, and monocytes (Supplementary Table 22 and Supplementary Fig. 36). Of note, *SIGLEC11* is near exclusively expressed in microglia (99%, Fig. 2A and Supplementary Table 20).

***RBCK1* locus (L40). Tier 1: *RBCK1* (Score = 12).** In this locus with a single and nearest candidate gene *RBCK1*, that was prioritized as a Tier 1 gene, we identified that the lead variant overlapped with significant eQTLs in temporal and frontal lobe, showing a *RBCK1* expression decreasing effect for the risk allele. Consequently, we observed a fine-mapped significant eTWAS hit (supported also by eQTL coloc PP4 of 99%) in DLPFC suggesting a potential protective effect of increased predicted *RBCK1* expression for ADD (Supplementary Table 22, 24, 26 and Supplementary Fig. 36a, 39, 41).

***SLC2A4RG* locus (L41). Tier 1: *LIME1* (Score = 22).** In novel locus 41, we did not obtain any significant results from e/sQTL coloc, e/sTWAS, and MetaMeth analyses. We thus considered the significant effects of the lead variants in molecular QTL catalogues; and we identified, uniquely in DLPFC, that the risk allele of the lead variant rs6742, a 3' UTR variant for *SLC2A4RG*, is associated with decreased *LIME1* and *RTEL1* levels and associated with increased preference for the chr20:63689132-63689750 splice junction (a canonical exon skipping event as in the ENST00000425905.5 short transcript) in *RTEL1* (Supplementary Tables 22-23 and Supplementary Fig. 36, 37). Moreover, we identified *LIME1* as a modulator of APP metabolism (Supplementary Fig. 45), where its silencing increased APP metabolism in the functional screen, consistent with expression decreasing effect of risk allele on *LIME1* expression. Consequently, our gene prioritization methodology prioritized *LIME1* in this locus as a Tier 1 gene.

***APP* locus (L42). Tier 1: *APP* (Score = 23.5).** *APP*, an obvious candidate for AD, was the Tier 1 prioritized gene in novel locus 42. We could detect the significant *APP* expression decreasing effect of the risk allele of the intronic lead variant rs2154481 in blood and monocytes (Supplementary Table 22 and Supplementary Fig. 36a, 36b), but not in AD-relevant bulk brain regions or in microglia. Of note, we also identified *CYYR1-AS1,* a non-coding gene in between *APP* and *ADAMTS1*, as having brain eQTL coloc & fine-mapped eTWAS signals, where its predicted increased expression is associated with increased ADD risk (Supplementary Tables 24, 26 and Supplementary Fig. 39, 41). Two other non-coding

genes, *AP000230.1* (proximal to *APP*) and *AP0001596.6* (proximal to *CYYR-AS1*), also had significant eTWAS associations in brain (Supplementary Tables 24, 26 and Supplementary Fig. 39, 41).

Loci with Tier 1 and Tier 2 prioritized genes

***ADAM17* locus (L2). Tier 1: *ADAM17* (Score = 13). Tier 2: *ITGB1BP1* (Score = 7).** In novel locus 2, the lead variant is positioned near the promoter region of *ADAM17* which was prioritized as a Tier 1 gene. Its eQTL signal colocalized with ADD genetic association signal (coloc PP4 values of 73% both in BA22 and in BA10) (Supplementary Table 24 and Supplementary Fig. 39) meanwhile eTWAS showed a potential protective effect of increased *ADAM17* expression for ADD (though, it could not pass stringent Bonferroni-correction). In addition, the ADD signal colocalized with the sQTLs that are controlling the splicing of the proximal first exons of *ADAM17* in both temporal and frontal lobe, which was also supported by sTWAS results (Supplementary Tables 25, 27 and Supplementary Fig. 40, 42). Another possible candidate was *ITGB1BP1*, prioritized at a lower confidence than *ADAM17* as a Tier 2 gene, where in AD-relevant brain regions the regulation of numerous *ITGB1BP1* splice junctions was colocalized with the GWAS signal, and these splice events were predicted to be associated with ADD (Supplementary Tables 25, 27 and Supplementary Fig. 40, 42).

***IDUA* locus (L7). Tier 1: *DGKQ* (Score = 38.5). Tier 2: *IDUA* (Score = 21).** For *IDUA* locus, among seven candidate genes, the three most proximal genes to the lead variant obtained similarly high number of hits in QTL-GWAS integration domains. The lead variant rs3822030 is positioned in a regulatory active promoter region of *SLC26A1* and intron of *IDUA*, and the risk allele was found to be significantly associated with increased methylation levels of a CpG site in proximity (cg21616051) as mQTL (Supplementary Table 29 and Supplementary Fig. 38a). In brain, eQTL coloc & fine-mapped eTWAS results mainly supported *SLC26A1* over the other two genes, suggesting a *SLC26A1* expression decreasing effect on the ADD risk in the locus through a plausible mechanism affecting its promoter sequence (Supplementary Tables 22, 24, 26 and Supplementary Fig. 36a, 39, 41). Moreover, *DGKQ* was also of interest as its eQTLs colocalized with ADD genetic association signal uniquely among *SLC26A1*, *DGKQ* and *IDUA* in frontal lobe (near 100% coloc PP4 in DLPFC) and as we also observed significant eTWAS association in EADB Belgian LCL dataset (however in risk-increasing direction contrary to its predicted brain expression effect) (Supplementary Tables 22, 24, 26 and Supplementary Fig. 36a, 39, 41). Importantly, DGKQ underexpression was associated with the modulation of the APP metabolism (Supplementary Fig. 45). Furthermore, sTWAS associated 5 splice junctions in *IDUA* (sQTL signal for 3 of these also colocalized with ADD signal) and 2 splice junctions in *DGKQ* with genetic ADD risk in the locus (Supplementary Tables 25, 27 and Supplementary Fig. 40, 42). Consequently, in this complex locus our gene prioritization method prioritized *DGKQ* with a higher confidence (Tier 1) than *IDUA* (Tier 2). Of note, no gene prioritization tier was assigned to *SLC26A1*, even if the weighted score for *SLC26A1* was also considerable (15). Taken together, *DGKQ* is a strong candidate in this locus, but further investigation is required to assess the probability of multiple genes responsible for the GWAS signal in this complex locus.

***ANK3* locus (L22). Tier 1: *CCDC6* (Score = 5). Tier 2: *ANK3* (Score = 4).** Between two candidate genes (*ANK3* and *CCDC6*), *ANK3* is the nearest gene to the lead variant in the locus. The lead variant is a significant eQTL for both genes in DLPFC where the minor and protective allele is associated with decreased expression for both genes (Supplementary Table 22 and Supplementary Fig. 36a). However, even though not passing stringent Bonferroni-corrected thresholds, predicted lower expression of *CCDC6* suggestively correlated with lower ADD risk in the locus, and *CCDC6* was also placed among the 90% credible gene set in the fine-mapping of eTWAS results in TCX and DLPFC brain regions. Moreover, importantly, we identified a colocalization between *CCDC6* eQTL and ADD

genetic association signal in microglia (MiGA Meta coloc PP4 = 81%) (Supplementary Table 24 and Supplementary Fig. 39). This is in line with the observation in a recent AD GWAS where monocyte eQTL signal of *CCDC6* colocalized with the AD signal[119]. Taken together, based on the current evidence, our methodology prioritized *CCDC6* over *ANK3* (despite the fact that it was the nearest gene) in this locus with a higher probability of being the causal risk gene, however we do not fully rule out the possibility for *ANK3* being the risk gene in the locus.

***TPCN1* locus (L26). Tier 1: *RITA1* (Score = 13.5). Tier 2: *IQCD* (Score = 9).** In novel locus 26, even though the minor and the risk allele of the lead variant is not frequent (MAF = 7%), we were able to identify significant and consistent eQTL association of it with decreased *RITA1* expression across all investigated brain regions except for BA10 and across several monocyte eQTL catalogues; and with increased *IQCD* and decreased *TPCN1* expression only in DLPFC. Moreover, eQTL coloc and eTWAS results mainly supported *RITA1* and *IQCD* (where increased ADD risk was correlated with decreased predicted *RITA1* expression and increased predicted *IQCD* expression), however implicated *RITA1* in more brain regions and with stronger probabilities. Therefore, in this locus our methodology prioritized *RITA1* as a Tier 1 gene based on the current evidence, but also accounted for possibility of *IQCD* being the risk gene in the locus at a lower confidence (Tier 2) (Supplementary Tables 22, 24, 26 and Supplementary Fig. 36a, 39, 41). Of note, the nearest gene in the locus, *TPCN1*, was not prioritized with a weighted score of 6.

***WDR81* locus (L34). Tier 1: *WDR81* (Score = 44). Tier 2: *SERPINF2* (Score = 26).** In this locus, the lead variant is a deleterious inframe deletion (p.Glu1033del, CADD = 16.37) within *WDR81.* The additional evidence was obtained from its significant APP modulation effect, colocalization of its eQTLs in temporal cortex with ADD genetic association signal (PP4 = 78%), and colocalization of its sQTLs for a cryptic splicing event (chr17:1732831-1733303) in DLPFC with ADD genetic association signal (PP4 = 79%) (Supplementary Tables 24-25 and Supplementary Fig. 39, 40, 45). However, we identified that *SERPINF2* (downstream to *WDR81*) also modulates APP, but with a rather lower log2 fold-change in APP mCherry signal compared to *WDR81* (Supplementary Fig. 45), and the sQTLs for canonical skipping (chr17:1745395-1747019) of exon 5 colocalized with ADD genetic association signal in DLPFC (coloc PP4 = 95%) (Supplementary Table 25 and Supplementary Fig. 40). Of note, we also observed cryptic splicing events in a repetitive non-coding region between *WDR81* and *SERPINF2* whose sQTLs greatly colocalized with ADD genetic association signal (Supplementary Table 25 and Supplementary Fig. 40). Consequently, our gene prioritization strategy here prioritizes *WDR81* as a Tier 1 and *SERPINF2* as a Tier 2 gene, despite the fact that both genes scored similarly in all domains except for variant annotation domain where *WDR81* collected more points for lead variant being a deleterious inframe deletion for this gene. However, it is important to note that we also cannot rule out the possibility of two genes being causal risk genes together in this locus.

***MYO15A* locus (L35). Tier 1: *MYO15A* (Score = 9). Tier 2: *LLGL1* (Score = 6), *TOP3A* (Score = 6).** In the novel locus 35, *MYO15A*, *LLGL1*, and *TOP3A* were of interest based on the number of significant associations in eQTL-GWAS and sQTL-GWAS integration domains. The nearest gene *MYO15A* had consistent eQTL coloc hits across all brain regions assessed (with between 72% - 85% eQTL coloc PP4), meanwhile *LLGL1* had an eQTL coloc PP4 of 96% in DLPFC and *TOP3A* had an eQTL coloc PP4 of 88% in BA36 (Supplementary Table 24 and Supplementary Fig. 39). On the other hand, the protective minor allele of the lead variant was significantly associated with decreased *TOP3A* expression in multiple brain regions and with increased *LLGL1* expression in DLPFC and naïve state monocytes (Supplementary Table 22 and Supplementary Fig. 37). The same protective allele also increased the preference for chr17:18163838-18167590 exon skipping event within FERM domain of *MYO15A* in DLPFC, and this sQTL signal colocalized with the

GWAS signal (sQTL coloc PP4 of 82%) (Supplementary Tables 23, 25 and Supplementary Fig. 37, 40). Consequently, with gene prioritization weighted score of 9, *MYO15A* was prioritized as Tier 1 gene; but *LLGL1* and *TOP3A* were also prioritized as Tier 2 genes in this locus with a score of 6.

<u>Loci with multiple Tier 2 prioritized genes</u>

***PRKD3* locus (L3). Tier 2: *PRKD3* (Score = 2), *CEBPZOS* (Score = 2), *EIF2AK2* (Score = 2).** In this locus, we only observed significant eQTL - lead variant overlap for *CEBPZOS* and *EIF2AK2*, and significant sQTL - lead variant overlap for two splice junctions in *NDUFAF7*; meanwhile *PRKD3* was the nearest gene. However, due to the lack of adequate scores from other type of evidence categories, our methodology assigned *PRKD3*, *CEBPZOS*, and *EIF2AK2* as Tier 2 genes in this locus, and none of these genes could be further prioritized as a Tier 1 gene.

***SNX1* locus (L28). Tier 2: *SNX1* (Score = 10), *FAM96A* (Score = 9).** Among the nine candidate genes presenting AD-related modulations in 1 Mb around the lead variant, *APH1B* (~820kb away from the lead variant) has very significant eQTL coloc, eTWAS and sTWAS hits in our analyses (Supplementary Tables 24, 26, 27 and Supplementary Fig. 39, 41, 42). However, we previously determined that *SNX1* and *APH1B* GWAS signals in this locus were independent (Supplementary Tables 3 and 4). We then considered four candidate genes that are closer to the independent SNX1 lead variant: *FAM96A* (alias being *CIAO2A*), *SNX1*, *SNX22*, and *CSNK1G1*.The risk allele of the lead variant rs3848143 is also associated with decreased *SNX1* expression in BA44, corroborated by eTWAS fine mapping for this brain region (Supplementary Tables 22, 26 and Supplementary Fig. 36a, 41). We additionally identified multiple sTWAS hits across different AD-relevant brain regions in proximity of *SNX1* lead variant, including in *FAM96A* and *CSNK1G1* which were both related to canonical exon- skipping splice events that were predicted to be protective for ADD (Supplementary Table 27 and Supplementary Fig. 42). Additionally for *FAM96A* we also observed that the lead variant was also a significant sQTL for regulation of the above mentioned splice junctions in *FAM96A*, additional lead variant eQTL effects for *FAM96A* were found in monocytes and in LCL, and *FAM96A* had a significant hit in GTEx LCL panel of eTWAS (suggesting a risk-increasing effect of decreased *FAM96A* expression) (Supplementary Tables 22, 23, 26 and Supplementary Fig. 36, 37, 41). Consequently, our prioritization method classified *SNX1* and *FAM96A* genes as Tier 2 genes.

***PRDM7* locus (L33). Tier 2: *PRDM7* (Score = 3.5), *CDK10* (Score = 3).** In the PRDM7 locus found close to the telomeric end of chromosome 16, MetaMeth showed significant association between higher ADD risk and lower predicted methylation of cg06295223 (Supplementary Table 28 and Supplementary Fig. 38b, that is a CpG site whose methylation is positively correlated (50- 75% percentile group) between blood and brain and located in the promoter region of *PRDM7*. eTWAS also identified a distant significant association for predicted expression of distal *CDK10* gene in GTEx frontal cortex (Supplementary Table 26 and Supplementary Fig. 41), meanwhile the minor risk-increasing allele of the lead variant was associated with increased *DBNDD1* expression in DLPFC. Of note, the *TUBB8P7* pseudogene also presented considerable significant AD-related modulations in the eQTL-GWAS integration domain(Supplementary Tables 22, 24, 26, 30 and Supplementary Fig. 36, 38, 40), and collected a total weighted score of 12, making it the only non-coding gene that would have been prioritized if we would have also considered non-coding genes in our methodology. In conclusion, considering the current evidence, our gene prioritization methodology classified both *PRMD7* and *CDK10* as Tier 2 genes in this locus, we were not able to prioritize a single gene with a higher confidence.

***KLF16* locus (L37). Tier 2: *KLF16* (Score = 9), *REXO1* (Score = 9), *ATP8B3* (Score = 8.5).** In novel locus 37, the association signal overlapped with the independent *ABCA7* signal upstream when 1 Mb extended coordinates were used to choose the candidate genes; therefore we considered *TCF3*, *KLF16*, *REXO1*, and *ATP8B3* that are proximal to the independent *KLF16* lead variant (Supplementary Tables 3 and 4). The risk allele of the lead variant rs149080927 (that is a 1 bp deletion) was significantly associated with increased *KLF16* expression (in multiple brain eQTL catalogues), increased *ATP8B3* expression, and decreased *REXO1* in AD-related brain regions (and additionally in macrophages with decreased *REXO1* expression) (Supplementary Table 22 and Supplementary Fig. 36a). MetaMeth also implicated a CpG within *ATP8B3* whose decreased predicted methylation is associated with increased ADD risk (Supplementary Table 28 and Supplementary Fig. 38b). In *REXO1*, the regulation of two splice junctions was also affected by the lead variant in brain (Supplementary Table 23 and Supplementary Fig. 37). Finally, we observed fine-mapped *KLF16* eTWAS associations (Supplementary Table 26 and Supplementary Fig. 41) in TCX and DLPFC that are independent of *POLR2E* eTWAS associations (Supplementary Fig. 44b) that are likely driven by another ADD risk locus that is positioned upstream, *ABCA7* locus. However; similarly, we also observed fine-mapped eTWAS hits in other panels investigated, such as *ATP8B3* in GTEx hippocampus, cortex, and BA24 (where increased predicted *ATP8B3* expression was correlated with increased ADD risk), and *REXO1* in GTEx frontal cortex (where decreased predicted *REXO1* expression was correlated with increased ADD risk). Taken together, the current evidence does not allow us to further prioritize one of these three genes over another, thus all three genes were classified as Tier 2 genes by our gene prioritization methodology.

***LILRB2* locus (L39). Tier 2: *MYADM* (Score = 3), *LILRB2* (Score = 2).** For *LILRB2* locus, in brain we could only find a considerable eQTL coloc for distal *MYADM* gene (~400kb from the lead variant) in BA44 region (coloc PP4 = 93%) (Supplementary Table 24 and Supplementary Fig. 39). Moreover, the risk allele of the lead variant rs587709 was associated with decreased expression of the nearest gene *LILRB2* in blood in both GTEx and eQTLGen (Supplementary Fig. 36a). With these evidences, our methodology prioritized both genes as Tier 2 risk genes in this locus; however, bibliographical data strongly supported *LILRB2*, as LilRb2 is an Aβ receptor and its murine homolog PirB is required for the deleterious effect of Aβ oligomers on hippocampal long-term potentiation in an AD-like mouse model[200]. In addition, molecules that inhibit Aβ-LilrB2 interactions in vitro and on the cell surface reduce Aβ cytotoxicity[201]. Of note, *LILRB2* was exclusively expressed in microglia (99.7%, Fig. 2A and Supplementary Table 20).

<u>Loci with a single Tier 2 prioritized gene</u>

For the below five loci, except for the weighted scores collected from being the nearest gene, we were unable to find another supporting evidence in other categories. Therefore, the nearest genes were prioritized as Tier 2 genes by our gene prioritization methodology.

***COX7C* (L10). Tier 2: *COX7C* (Score = 2).**

***RASGEF1C* (L12). Tier 2: *RASGEF1C* (Score = 2).**

***HS3HT5* (L13). Tier 2: *HS3HT5* (Score = 2).**

***UMAD1* (L14). Tier 2: *UMAD1* (Score = 2).**

***FOXF1* (L32). Tier 2: *FOXF1* (Score = 2).**

Loci without prioritized genes

***IGH* gene cluster locus (L27).** In this locus, even though the lead variant is only associated with expression of numerous *IGH* cluster genes in blood, we had multiple significant hits, especially in sTWAS analyses. However, we observed that the majority of splice junctions identified in sTWAS are very long, complex, non-canonical splice events; thus, likely a consequence of known fusion events[202] in this complex telomeric region of chromosome 14. Therefore, we did not prioritize any genes in this complex IGH cluster locus (Supplementary Tables 22-23, 25-27, Supplementary Fig. 36, 37, 40-42).

11.3. Short description of some functions of the prioritized genes and their potential implication in AD

SORT1 (Sortilin 1) encodes a member of the VPS10-related sortilin family of proteins which also include SORL1. This protein is involved in the traffic of protein from the Golgi to the endosomes, secretory vesicles, and the cell surface[203]. GWAS revealed an association between *SORT1* and reduced plasma LDL-cholesterol (LDL-C) as well as reduced coronary artery disease (CAD)[204]. In AD, animal model studies suggest that sortilin is a beneficial protein for the reduction of amyloid pathology in APP/PS1 mice by promoting APP degradation[205]. SORT1 was also shown to be a neuronal receptor for GRN, down-regulating GRN extracellular levels under stress conditions[206].

ITGB1BP1 (integrin subunit beta 1 binding protein 1) encodes a protein interacting with a NPXY sequence motif of beta1 integrin and consequently is important for integrin signaling pathway[207].

ADAM17 (a disintegrin and metalloprotease domain 17) encodes a protein which belongs to the same family than ADAM10. As for the latter, ADAM17 has been proposed to carry an α-secretase activity which leads to the increasing secretion of soluble APP-α fragment and reduction of Aβ generation[208]. In addition, it has been proposed that TREM2 is shed via Adam17 proteolytic activity[209]. Finally, ADAM17 is also known as TACE (TNFα converting enzyme) and is involved in inflammatory processes[210]. A rare loss-of function in *ADAM17* has been associated with familial forms of late-onset AD[211].

EIF2AK2 (translation initiation factor 2 alpha kinase 2) encodes a serine/threonine protein kinase which can phosphorylate translation initiation factor EIF2S1, which in turn inhibits protein synthesis. The encoded protein has been described to regulate NRLP3 inflammasome[212].

CEBPZOS (CCAAT enhancer binding protein zeta opposite strand) encodes a protein without known functions and no articles are available in PubMed.

PRKD3 (protein Kinase D3) encodes a serine/threonine protein kinase that has been mainly involved in cancer. This protein has been described as critical for CLU protein stability via inhibiting CLU's lysosomal distribution and degradation[213].

NCK2 (non-catalytic (region of) tyrosine kinase adaptor protein-2) encodes a protein involved in integrin signaling and as a consequence, signaling to regulate survival, proliferation and cell shape as well as polarity, adhesion, migration and differentiation[214]**.** In neurons, nck2 has been involved in change of neuron morphology[215] and synaptic transmission[216]. NCK2 is also interacting with PSEN2 and EGFR.

ICA1L (islet cell autoantigen 1 like) is a paralog of ICA1. This locus has been associated with small cerebral vessel disease[217]. It has been recently proposed that brain protein abundance of ICAL1 was genetically regulated in AD[181].

MME (membrane metalloendopeptidase) encodes neprilysin (NEP). In vivo and cell culture experiments have shown that a decreased NEP level results in an increased Aβ level and vice versa. NEP has been proposed as one of the most prominent degrading Ab enzyme[218].

DGKQ (diacylglycerol kinase theta) encodes a protein that, as other Diacylglycerol kinases, is an important regulator of lipid signaling and, consequently, important regulator of many diglyceride-dependent and phosphatidic acid-dependent proteins[219]. Ablation of the mammalian DGKQ orthologue, DGK-1 in *C. elegans*, prevents serotonin-mediated inhibition of neurotransmitter release at neuromuscular junctions suggesting that this protein is involved in synaptic transmission[220].

IDUA (iduronidase alpha-L) encodes an enzyme that hydrolyzes the terminal alpha-L-iduronic acid residues of two glycosaminoglycans, dermatan sulfate and heparan sulfate. In *Idua* -/- mouse, a modulation of the APP metabolism was reported likely through cathepsin B activation[221].

RHOH (ras homolog family member H) encodes a protein which acts as a negative regulator of cell growth and survival. The protein has been mainly involved in cancers[222].

OTULIN encodes a deubiquitinase which is an essential negative regulator of inflammation and autoimmunity[223]. OTULIN causes a potentially fatal autoinflammatory pathology termed OTULIN-related autoinflammatory syndrome (ORAS)[224]. Importantly, overexpression of OTULIN favours microglia activation and neuroinflammation through inhibition of the NF-κB signaling pathway in cerebral ischemia/reperfusion rats[225]. OTULIN is a specific regulator of the LUBAC complex which is a major actor of the TNF signaling[226]. Moreover, OTULIN antagonizes the cargo loading, retromer binding, endosome to plasma membrane trafficking functions of SNX27 (sorting nexin 27)[227] which is a protein known to regulate β-amyloid production potentially in interaction with Sorl1 or Presenilin 1[228,229].

COX7C (cytochrome c oxidase subunit 7C) encodes a protein part of the terminal component of the mitochondrial respiratory chain, and that catalyzes the electron transfer from reduced cytochrome c to oxygen.

TNIP1 (NFAIP3 interacting protein 1) encodes a protein which plays a role in autoimmunity and tissue homeostasis through the regulation of nuclear factor kappa-B activation. The TNIP1 locus has been associated with the risk of amyotrophic lateral sclerosis[230] and auto-immune diseases[231].

RASGEF1C (Ras-GEF domain-containing family member 1C) encodes a specific activator of Rap2 which regulates cell-cell adhesion[232]. Little is known about its functions in brain.

HS3ST5 (heparan sulfate-glucosamine 3-sulfotransferase 5) encodes a protein involved in post-translational modifications. Heparan sulfate proteaglycans have been involved in multiple pathways in AD from abeta production[233] or Tau seeding[234] to neuroinflammation[235].

UMAD1 (UBAP1-MVB12-associated (UMA) domain containing 1) encodes a protein for which we know almost nothing (only 3 publications on PubMed).

ICA1 (islet cell autoantigen 1) encodes a protein which has been initially described as an autoantigen associated with autoimmune type 1 diabetes (T1D)[236]. In neurons, ICA1 was shown to be involved in the recruitment of AMPA receptors at the synapses[237].

TMEM106B (transmembrane protein 106B) is well known to be associated with the risk of developing fronto-temporal dementia[136]. The corresponding protein has been involved in lysosomal dysfunction, myelin deficits[238], dendritic trafficking[239] or cell death[240].

JAZF1 (JAZF zinc finger 1) encodes a nuclear protein which functions as a transcriptional repressor. This gene has been associated with the risk of developing Type 2 diabetes and the protein regulates glucose and lipid homeostasis and inflammation[241].

EGFR (epidermal growth factor receptor) encodes a cell surface protein that binds to epidermal growth factor. Activation of the EGFR enhances neurite growth and regeneration through SORL1 functions[242] and Presenilin 1 has been shown to regulate EGFR turnover and signaling in the endosomal-lysosomal pathway[243]. EGFR was also proposed as target for treating amyloid-β-induced memory loss[244].

CTSB (cathepsin B) encodes a protein which is a lysosomal cysteine protease with both endopeptidase and exopeptidase activity. CTSB has been described to either participate to the production of pyroglutamate Aβ[245] or degrade amyloid-β in mice expressing APP[246]. In addition, oxidative stress has been proposed to activate NLRP3 through upregulating CTSB activity[247].

SHARPIN (SHANK associated RH domain interactor). The missense variant associated with AD risk has been described to attenuate an inflammatory/immune response that may promote late-onset AD development[248]. A common variant has also been associated with neuroanatomical variation in the limbic system[249]. The corresponding protein has also been described as a novel postsynaptic density protein[250] and interestingly, SHARPIN is an endogenous inhibitor of β1-integrin activation[251] and is part of the core enzymatic LUBAC complex[226].

ABCA1 (ATP binding cassette subfamily A member 1) encodes a member of the superfamily of ATP-binding cassette (ABC) transporters which transport various molecules across extra- and intracellular membranes. ABCA1 deficiency affects Basal Cognitive Deficits and Dendritic Density in Mice[252]. In addition, ABCA1 Deficiency was shown to exacerbate blood-brain barrier and white matter damage after stroke[253]. Its overexpression was described to reduce amyloid deposition in an AD-like mouse model[193].

CCDC6 (coiled-coil domain containing 6) encodes a protein ubiquitously expressed which may be a tumor suppressor. A chromosomal rearrangement resulting in the expression of a fusion gene containing a portion of this gene with different protooncogenes has been reported[254]. Little is known about its potential function in the brain but this protein has been proposed to be involved in actin cytoskeleton rearrangement[255].

ANK3 (Ankyrin 3) encodes a protein (AnkG) which is part of a family that is believed to link the integral membrane proteins to the underlying spectrin-actin cytoskeleton. Neuronal expression of AnkG is higher in AD brains when compared with healthy control subjects. AnkG is present in exosomal vesicles, and it accumulates in β-amyloid plaques[256].

TSPAN14 (tetraspanin 14) encodes a protein which regulates the trafficking and function of ADAM10[195]. The TSPAN14 locus has been also associated with periventricular white matter hyperintensities[129].

BLNK (B cell linker) encodes a protein which plays a critical role in B cell development and function[257], and plays an important role in PLCg2 activation, another genetic risk factor of AD[258]. BLNK is also significantly upregulated when exposed to Aβ[259].

PLEKHA1 (leckstrin homology domain containing A1) encodes a protein localized to the plasma membrane where it specifically binds phosphatidylinositol 3,4-bisphosphate. This protein may be involved in the formation of signaling complexes in the plasma membrane. The *PLEKHA1* locus has been associated with the risk of macular degeneration[260].

RITA1 (RBP-J interacting and tubulin associated) encodes a tubulin-binding protein that acts as a negative regulator of the Notch signaling pathway[261].

IQCD (IQ motif containing D) encodes a protein for which we know almost nothing.

FAM96A/CIAO2A (cytosolic iron-sulfur assembly component 2A) encodes a protein that has been described as a novel pro-apoptotic tumor suppressor[262].

SNX1 (sorting nexin 1) encodes a component of the retromer complex and is involved in several stages of intracellular trafficking. In particular, it has been described the participation of SNX1 in Sorl1 sorting[263], Sorl1 being a major genetic risk factor of AD and a major actor of the APP metabolism.

CTSH (cathepsin H) encodes a lysosomal cysteine proteinase important in the overall degradation of lysosomal proteins. Interestingly, the induction of neuronal death by up-

regulation of CTSH in microglia following LPS treatment has been reported[264] and CTSH has also been described to be over-expressed in microglia following Aβ exposure.

DOC2A (double C2 domain alpha) encodes a protein that is mainly expressed in the brain and potentially involved in Ca(2+)-dependent neurotransmitter release[265]. None is known about its potential implication in AD but a copy number variation of this gene has been associated with schizophrenia[266].

MAF (alias c-MAF) encodes for a transcriptional factor which appears to be mainly expressed in microglia. In this cell-type, adult microglia from p53-deficient mice have increased expression of this anti-inflammatory transcription factor[267].

FOXF1 **(Forkhead box F1)** belongs to the forkhead family of transcription factors. Its function in the brain is unknown.

CDK10 (cyclin dependent kinase 10) belongs to the CDK subfamily of the Ser/Thr protein kinase family and the corresponding protein has been involved in cell cycle progression[268]. Little is known about its potential function in the brain but CDK10 has been associated with modulation of neural progenitor survival[269].

PRDM7 (PR/SET domain 7) encodes a protein believed to have roles in transcription through histone methyltransferase activity[270]. This gene is only expressed in testis.

WDR81 encodes a multi-domain transmembrane protein which is predominantly expressed in the brain and is thought to play a role in endolysosomal trafficking[271]. WDR81 regulates adult hippocampal neurogenesis[272].

SERPINF2 (serpin family F member 2) encodes a member of the serpin family of serine protease inhibitors. SERPINF2 exhibits CpG associations with AD risk and altered expression in AD brains[164].

MYO15A (myosin XVA) encodes for an unconventional myosin. Mutations in these gene have been associated with hearing loss[273].

LLGL1 (LLGL scribble cell polarity complex component 1) encodes a protein that is part of a cytoskeletal network. LLGL1 directly binds to and promotes internalization of N-cadherin. Disruption of the N-cadherin-LLGL1 interaction during cortical development in vivo may lead to malformations of the cerebral cortex[274].

TOP3A (topoisomerase III alpha) encodes a DNA topoisomerase. Topoisomerases have been described to crosstalk with EGRF in cancer[275], and have been involved in autism and synaptic plasticity[276].

GRN (progranulin) is a gene known to be responsible for monogenic forms of fronto-temporal dementia. GRN is mainly expressed in microglia and as BLNK, its expression is significantly upregulated when exposed to Aβ in microglia[259]. GRN deficiency significantly reduces diffuse Aβ plaque growth in an AD-like mouse model and it has been proposed that this protective effect is due, in part, to enhanced microglial Aβ phagocytosis[277].

ATP8B3 (ATPase phospholipid transporting 8B3) encodes a protein mainly expressed in testis and little is known about its function in the brain.

REXO1 (RNA exonuclease 1 homolog). Little is known about its function in general.

KLF16 (Krüppel-Like Factor 16) encodes a transcription factor that binds GC and GT boxes and displaces Sp1 and Sp3 from these sequences[278]. This transcriptional factor might be involved in dopaminergic transmission in the brain[279].

SIGLEC11 (sialic acid binding Ig like lectin 11) encodes a protein belonging to the immunoglobulin superfamily. Siglec-11, which mediates immunosuppressive signals, is only expressed in microglia and is the only Siglec protein expressed in this cell type. Salminen et al. has proposed the following hypothesis: “aggregating amyloid plaques are masked in AD

by sialylated glycoproteins and gangliosides. Sialylation and glycosylation of plaques, mimicking the cell surface glycocalyx, can activate the immunosuppressive Siglec-11 receptors, as well as hiding the neuritic plaques, allowing them to evade the immune surveillance of microglial cells. This kind of immune evasion can prevent the microglial cleansing process of aggregating amyloid plaques in AD."[280].

MYADM (myeloid associated differentiation marker) encodes a protein which has been associated with blood pressure and hypertension[281]. Nothing is known about its potential implication in the brain.

LILRB2 (leukocyte immunoglobulin like receptor B2) encodes a protein thought to control inflammatory responses and cytotoxicity to help focus the immune response and limit autoreactivity. LilRb2 has been described to be an Ab receptor and the murine homolog PirB is required for deleterious effect of Aβ oligomers on hippocampal long-term potentiation in an AD-like mouse model[200]. In addition, molecules that inhibit Aβ-LilrB2 interactions in vitro and on the cell surface, reduce Aβ cytotoxicity[201].

RBCK1 (RANBP2-type and C3HC4-type zinc finger containing 1) encodes a protein (HOIL-1) that is part of the core of the LUBAC complex[226]. This complex is the only known E3 ubiquitin-ligating enzyme producing M1 ubiquitin linkages de novo. This complex also involving OTULIN and SHARPIN is a crucial modulator of innate and adaptive immune responses, and act by regulating inflammatory and cell death signaling[226].

RTEL1 (telomere elongation helicase 1) encodes a DNA helicase which functions in the stability, protection and elongation of telomeres. Only three publications are available on PubMed.

LIME1 (Lck interacting transmembrane adaptor 1) encodes a transmembrane adaptor protein involved in signaling pathways via its association with the Src family kinases Lck and Lyn. LIME1 has been proposed to interact with Grb2[282], a major actor of the APP metabolism (this observation potentially allows to explain its impact on the APP function/metabolism[283]). In addition, this protein has been described to potentially interact with PlCg2[282]. However, since LIME1 is not expressed in microglia, the latter observation precludes its potential implication in AD though this interaction in microglia.

12. STRING protein interaction analysis

The genes from the sets of interest (previous known genes and/or prioritized genes in Fig. 2, light and dark green) were tested for an excess of high-confidence protein-protein interactions as in[39]. First, a list of high-confidence (confidence score >0.7) human protein-protein interactions was downloaded from the latest version (v11.0)[284] of the STRING database (http://string-db.org). Then, a protein interaction network was generated for each of the genes as follows: (i) Choose a gene to start the network (the "seed" gene); (ii) For each remaining gene in the set of significant genes, add it to the network if its corresponding protein shows a high-confidence protein interaction with a protein corresponding to any gene already in the network; (iii) Repeat step 2 until no more gene can be added; (iv) Note the number of genes in the network; (v) Repeat, choosing each of the genes in turn as the seed gene; (vi) Note the size of the largest network.
To test whether the largest network was larger than expected by chance, given the total number of protein-protein interactions for each gene, 50,000 random sets of genes were generated, equal in number to the test set, with each gene chosen to have the same total number of protein-protein interactions as the corresponding gene in the actual data. Protein networks were generated for each gene as described above, and the size of the largest such network compared to that observed in the actual data. Excess interactions between two gene sets were tested by a similar approach.

13. Genetic risk score analyses

13.1. Description of indices of predictive accuracy

C-Index: The concordance index (C-index) can be used to index the discriminative capacity of a marker in survival data. It quantifies the proportions of pairs of patients for which the patient with a shorter time to conversion received a higher risk score. It has a range of 0-1 with 0.5 indicating a useless model (discrimination at chance level) and 1 indicating perfect discrimination. The C-index can also be formulated as the weighted average of the area of the time-point specific receiver operating characteristic curves (ROC-AUC) in survival data[285]. Therefore, the C-index is the same as the well-known area under the receiver operating characteristic curve (ROC-AUC) but usually reports on the average discriminative ability across time points.
The C-index was computed using the “cindex” function from the “pec” R-package (2020.11.17)[286], after accounting for censoring in the survival data via the inverse probability weighting derived from a Cox model[287]. To assess the added value of the GRS, we computed the difference in C-index between models excluding and including the GRS. A higher score therefore indicates improved discrimination between converters and non-converters due to the GRS. Since the c-index is based on ranks of participants, it has the advantage of being robust to model miscalibration but, as a downside, the interpretation of the change in the C-index depends on the accuracy of the baseline model (here, the model excluding the GRS). Small changes in the C-index are more likely to indicate small changes when performance of the baseline model is low, while the same change in the C-index from a well-performing baseline model can indicate a much stronger effect of the predictor of interest[288]. To account for this property of the index, we always present the C-index of the baseline model together with the change in C-index when adding the GRS to the model. Of note, C-indices for the baseline model are expected to be highly heterogeneous between cohorts as the overall performance of the baseline model can depend on specific study characteristics such as age range or *APOE* frequency. Changes in the C-index, however, are expected to be more homogeneous across cohorts as shown in previous research[289].

Continuous NRI: In contrast to the C-index, the interpretation of the continuous net reclassification index (NRI) does not depend on the strength of the baseline model[288]. The NRI has a range of -2 to 2 and is computed here as the sum of the proportions of individuals with and without conversion to dementia whose predicted risk score changed in the correct direction when adding the GRS to the model (i.e. increased risk score in the dementia-converters and a decreased risk score in the dementia-non-converters, respectively)[290]. Higher NRI therefore indicate a generally more appropriate assignment of individual predicted risks. An NRI of 0.16 corresponds to a small effect size and an NRI of 0.395 corresponds to a moderate effect size for continuous predictors[288]. The NRI therefore focuses more on the individual effect of a specific covariate (here the GRS) while for instance the C-index assesses overall model performance[288]. However, as a downside, the NRI can be sensitive to miscalibration of the models and should therefore not be used as a single marker of predictive performance[290]. The NRI was computed using the “IDI.INF” function from the “survIDINRI” (1.1.1) R-package[291], taking censoring into account (as described by Uno et al.[292]).

IPA: We additionally computed the index of prediction accuracy (IPA) which is a scaled version of the Brier score which constitutes a general, overall measure of predictive performance[293]. The latter assesses the mean squared difference between predicted probability of the event (here predicted dementia conversion) to the actual outcome (observed dementia conversions). To interpret the Briers score more easily (i.e. a range from -1 to 1, with a higher score indicating better predictive performance), we used a baseline model lacking predictors to derive the IPA[294]. The IPA was computed using the “IPA”

function from the "riskRegression" (2020.12.8) R package[295]. The IPA can take values between -1 and 1 with negative values indicating a harmful model (i.e. worse prediction than simply using the base rate of the outcome), zero indicating a useless model (prediction at chance level) and positive values indicating improvement in prediction. To assess the discriminative capacity of the GRS, we focused on the change in the index when adding the GRS to the model as compared to the IPA based on predictions without the GRS. As an advantage, the IPA combines discriminative capacity and model calibration of the model but, as a disadvantage, its scale depends on the probability of the outcome (here the dementia incidence rate) which can hamper the comparability of the score across different cohorts.

As recommended, we did not report p-values for the indices, since the statistical test of the association between the GRS and the AD risk is based on the Cox regression result[296]. Instead, the indices were used to further quantify the effect size of the association with regard to the GRS's predictive accuracy for AD conversion.

13.2. Fixed effect and random effects meta-analysis

To derive an average effect of the GRS across the examined cohorts, individual cohort results were meta-analyzed using both fixed effect and random effects meta-analysis for the following reasons: inverse-variance weighted meta-analysis under the fixed effect model derives an average effect across those cohorts that have been included in the analysis[297,298]. The average across this set of cohorts is valid even in the presence of heterogeneity between cohorts[297–299]. However, if heterogeneity is suspected to be substantial, use of random effects meta-analysis has been recommended[300] which can additionally improve the generalization of results to studies not included in the analysis[298]. However, random effects meta-analysis may have limited power with small numbers of studies as used in this analysis[300]. We therefore, additionally performed a random-effects meta-analysis using the DerSimonian-Laird estimator for heterogeneity which is more robust to violations of the normality assumption of the distribution of effects between cohorts with small numbers of studies[301]. Knapp-Hartung adjustment was also performed as it improves maintenance of correct type I errors and is robust to changes in the between-study variance estimation[302].

14. List of URLs

Bedtools: https://bedtools.readthedocs.io/en/latest/
BCFtools: http://samtools.github.io/bcftools/bcftools.html
Samtools: http:/www.htslib.org/doc/samtools.html
gene2go: ftp://ftp.ncbi.nlm.nih.gov/gene/DATA/
Gene Ontology: http://geneontology.org/docs/download-ontology/
Reactome: https://reactome.org/download-data
KEGG and Pathway Interaction Database (PID) pathways: https://www.gsea-msigdb.org/gsea/msigdb/index.jsp
AMP-AD rnaSeqReprocessing Study: https://www.synapse.org/#!Synapse:syn9702085
MayoRNAseq WGS VCFs: https://www.synapse.org/#!Synapse:syn11724002
ROSMAP WGS VCFs: https://www.synapse.org/#!Synapse:syn11724057
MSBB WGS VCFs: https://www.synapse.org/#!Synapse:syn11723899
GTEx pipeline: https://github.com/broadinstitute/gtex-pipeline
Leafcutter: https://github.com/davidaknowles/leafcutter
RegTools: https://github.com/griffithlab/regtools
Enhanced version of FastQTL: https://github.com/francois-a/fastqtl
Picard: https://broadinstitute.github.io/picard/
eQTLGen: https://www.eqtlgen.org/

eQTL Catalogue database: https://www.ebi.ac.uk/eqtl/Brain xQTL serve: http://mostafavilab.stat.ubc.ca/xqtl/
GTEx v8 eQTL and sQTL catalogues: https://www.gtexportal.org/
coloc: https://github.com/chr1swallace/coloc
FUSION: https://github.com/gusevlab/fusion_twas
GTEx v8 expression and splicing prediction models: http://predictdb.org/
MetaXcan: https://github.com/hakyimlab/MetaXcan
FOCUS: https://github.com/bogdanlab/focus
qcat: https://github.com/nanoporetech/qcat
minimap2: https://github.com/lh3/minimap2
NanoStat: https://github.com/wdecoster/nanostat
mosdepth: https://github.com/brentp/mosdepth
ggplot2: https://ggplot2.tidyverse.org/
LocusZoom: https://github.com/statgen/locuszoom-standalone
pyGenomeTracks: https://github.com/deeptools/pyGenomeTracks
BECon website: https://redgar598.shinyapps.io/BECon/
VCFs of phased biallelic SNV and INDEL variants of 1KG samples (de novo called on GRCh38):
ftp://ftp.1000genomes.ebi.ac.uk/vol1/ftp/data_collections/1000_genomes_project/release/20190312_biallelic_SNV_and_INDEL/
MiGA eQTLs: https://doi.org/10.5281/zenodo.4118605
MiGA sQTLs: https://doi.org/10.5281/zenodo.4118403
MiGA Meta-analysis: https://doi.org/10.5281/zenodo.4118676
Wingo et al. pQTL data: https://www.synapse.org/#!Synapse:syn23627957

15. Supplementary References

1. De Roeck, A. *et al.* An intronic VNTR affects splicing of ABCA7 and increases risk of Alzheimer's disease. *Acta Neuropathol.* **135**, 827–837 (2018).
2. Sheardova, K. *et al.* Czech Brain Aging Study (CBAS): Prospective multicentre cohort study on risk and protective factors for dementia in the Czech Republic. *BMJ Open* **9**, (2019).
3. Steinberg, S. *et al.* Loss-of-function variants in ABCA7 confer risk of Alzheimer's disease. *Nat. Genet.* **47**, 445–447 (2015).
4. Ngandu, T. *et al.* A 2 year multidomain intervention of diet, exercise, cognitive training, and vascular risk monitoring versus control to prevent cognitive decline in at-risk elderly people (FINGER): A randomised controlled trial. *Lancet* **385**, 2255–2263 (2015).
5. Hanon, O. *et al.* Plasma amyloid levels within the Alzheimer's process and correlations with central biomarkers. *Alzheimer's Dement.* **14**, 858–868 (2018).
6. Dufouil, C. *et al.* Cognitive and imaging markers in non-demented subjects attending a memory clinic: Study design and baseline findings of the MEMENTO cohort. *Alzheimer's Res. Ther.* **9**, (2017).
7. Nicolas, G. *et al.* Screening of dementia genes by whole-exome sequencing in early-onset Alzheimer disease: Input and lessons. *Eur. J. Hum. Genet.* **24**, 710–716 (2016).
8. McKhann, G. *et al.* Clinical diagnosis of Alzheimer's disease: report of the NINCDS-ADRDA Work Group under the auspices of Department of Health and Human Services Task Force on Alzheimer's Disease. *Neurology* **34**, 939–44 (1984).
9. Kornhuber, J. *et al.* Early and differential diagnosis of dementia and mild cognitive impairment: *Dement. Geriatr. Cogn. Disord.* **27**, 404–417 (2009).
10. Luck, T. *et al.* Mild cognitive impairment in general practice: Age-specific prevalence and correlate results from the German study on ageing, cognition and dementia in primary care patients (AgeCoDe). *Dement. Geriatr. Cogn. Disord.* **24**, 307–316

(2007).
11. McKhann, G. M. *et al.* The diagnosis of dementia due to Alzheimer's disease: Recommendations from the National Institute on Aging-Alzheimer's Association workgroups on diagnostic guidelines for Alzheimer's disease. *Alzheimer's Dement.* **7**, 263–269 (2011).
12. Van Der Flier, W. M. & Scheltens, P. Amsterdam dementia cohort: Performing research to optimize care. *Journal of Alzheimer's Disease* vol. 62 1091–1111 (2018).
13. Holstege, H. *et al.* The 100-plus Study of cognitively healthy centenarians: rationale, design and cohort description. *Eur. J. Epidemiol.* **33**, (2018).
14. Aalten, P. *et al.* The Dutch Parelsnoer Institute - Neurodegenerative diseases; methods, design and baseline results. *BMC Neurol.* **14**, (2014).
15. Dubois, B. *et al.* Research criteria for the diagnosis of Alzheimer's disease: revising the NINCDS-ADRDA criteria. *Lancet Neurology* vol. 6 734–746 (2007).
16. Ramakers, I. *et al.* Biobank Alzheimer Center Limburg cohort: design and cohort characteristics. *Prep.*
17. (APA), A. P. A. *Diagnostic and statistical manual of mental disorders.* (1994).
18. Sachdev, P. S. *et al.* The Sydney Memory and Ageing Study (MAS): Methodology and baseline medical and neuropsychiatric characteristics of an elderly epidemiological non-demented cohort of Australians aged 70-90 years. *Int. Psychogeriatrics* **22**, 1248–1264 (2010).
19. Moreno-Grau, S. *et al.* Genome-wide association analysis of dementia and its clinical endophenotypes reveal novel loci associated with Alzheimer's disease and three causality networks: The GR@ACE project. *Alzheimer's Dement.* **15**, 1333–1347 (2019).
20. Ruiz, A. *et al.* Assessing the role of the TREM2 p.R47H variant as a risk factor for Alzheimer's disease and frontotemporal dementia. *Neurobiol. Aging* **35**, 444.e1–4 (2014).
21. Ikram, M. A. *et al.* The Rotterdam Study: 2018 update on objectives, design and main results. *Eur. J. Epidemiol.* **32**, 807–850 (2017).
22. Niemeijer, M. N. *et al.* ABCB1 gene variants, digoxin and risk of sudden cardiac death in a general population. *Heart* **101**, 1973–1979 (2015).
23. Loh, P.-R. *et al.* Reference-based phasing using the Haplotype Reference Consortium panel. *Nat. Genet.* **48**, 1443–1448 (2016).
24. De Bruijn, R. F. A. G. *et al.* Determinants, MRI correlates, and prognosis of mild cognitive impairment: The rotterdam study. in *Journal of Alzheimer's Disease* vol. 42 S239–S249 (IOS Press, 2014).
25. 3C Study Group. Vascular factors and risk of dementia: design of the Three-City Study and baseline characteristics of the study population. *Neuroepidemiology* **22**, 316–25 (2003).
26. Lambert, J.-C. *et al.* Genome-wide association study identifies variants at CLU and CR1 associated with Alzheimer's disease. *Nat. Genet.* **41**, 1094–9 (2009).
27. Harold, D. *et al.* Genome-wide association study identifies variants at CLU and PICALM associated with Alzheimer's disease. *Nat. Genet.* **41**, 1088–93 (2009).
28. Jansen, I. E. *et al.* Genome-wide meta-analysis identifies new loci and functional pathways influencing Alzheimer's disease risk. *Nat. Genet.* **51**, 404–413 (2019).
29. Gayán, J. *et al.* Genetic Structure of the Spanish Population. *BMC Genomics* **11**, (2010).
30. Antúnez, C. *et al.* The membrane-spanning 4-domains, subfamily A (MS4A) gene cluster contains a common variant associated with Alzheimer's disease. *Genome Med.* **3**, (2011).
31. Jessen, F. *et al.* AD dementia risk in late MCI, in early MCI, and in subjective memory impairment. *Alzheimer's Dement.* **10**, 76–83 (2014).
32. Reisberg, B., Ferris, S. H., De Leon, M. J. & Crook, T. The global deterioration scale for assessment of primary degenerative dementia. *Am. J. Psychiatry* **139**, 1136–1139 (1982).

33. Lambert, J. C. *et al.* Meta-analysis of 74,046 individuals identifies 11 new susceptibility loci for Alzheimer's disease. *Nat. Genet.* **45**, 1452–8 (2013).
34. Schmermund, A. *et al.* Assessment of clinically silent atherosclerotic disease and established and novel risk factors for predicting myocardial infarction and cardiac death in healthy middle-aged subjects: Rationale and design of the Heinz Nixdorf RECALL study. *Am. Heart J.* **144**, 212–218 (2002).
35. Stang, A. *et al.* Baseline recruitment and analyses of nonresponse of the Heinz Nixdorf Recall Study: Identifiability of phone numbers as the major determinant of response. *Eur. J. Epidemiol.* **20**, 489–496 (2005).
36. Winkler, A. *et al.* Association of diabetes mellitus and mild cognitive impairment in middle-aged men and women. *J. Alzheimer's Dis.* **42**, 1269–1277 (2014).
37. Wege, N. *et al.* Population-based distribution and psychometric properties of a short cognitive performance measure in the population-based Heinz Nixdorf Recall study. *Neuroepidemiology* vol. 37 13–20 (2011).
38. Naj, A. C. *et al.* Common variants at MS4A4/MS4A6E, CD2AP, CD33 and EPHA1 are associated with late-onset Alzheimer's disease. *Nat. Genet.* **43**, 436–41 (2011).
39. Sims, R. *et al.* Rare coding variants in PLCG2, ABI3, and TREM2 implicate microglial-mediated innate immunity in Alzheimer's disease. *Nat. Genet.* (2017) doi:10.1038/ng.3916.
40. Jun, G. *et al.* A novel Alzheimer disease locus located near the gene encoding tau protein. *Mol. Psychiatry* **21**, 108–17 (2016).
41. Jun, G. *et al.* Meta-analysis confirms CR1, CLU, and PICALM as alzheimer disease risk loci and reveals interactions with APOE genotypes. *Arch. Neurol.* **67**, 1473–84 (2010).
42. Kunkle, B. W. *et al.* Genetic meta-analysis of diagnosed Alzheimer's disease identifies new risk loci and implicates A$β$, tau, immunity and lipid processing. *Nat. Genet.* **51**, 414–430 (2019).
43. Kukull, W. A. *et al.* Dementia and Alzheimer disease incidence: A prospective cohort study. *Arch. Neurol.* **59**, 1737–1746 (2002).
44. Larson, E. B. *et al.* Exercise is associated with reduced risk for incident dementia among persons 65 years of age and older. *Ann. Intern. Med.* **144**, 73–81 (2006).
45. Beekly, D. L. *et al.* The National Alzheimer's Coordinating Center (NACC) database: The uniform data set. *Alzheimer Disease and Associated Disorders* vol. 21 249–258 (2007).
46. Morris, J. C. *et al.* The Uniform Data Set (UDS): Clinical and cognitive variables and descriptive data from Alzheimer disease centers. *Alzheimer Dis. Assoc. Disord.* **20**, 210–216 (2006).
47. Mirra, S. S., Hart, M. N. & Terry, R. D. Making the diagnosis of Alzheimer's disease: A primer for practicing pathologists. *Arch. Pathol. Lab. Med.* **117**, 132–144 (1993).
48. Nagy, Z. *et al.* Assessment of the pathological stages of Alzheimer's disease in thin paraffin sections: A comparative study. *Dement. Geriatr. Cogn. Disord.* **9**, 140–144 (1998).
49. Braak, H. & Braak, E. Neuropathological stageing of Alzheimer-related changes. *Acta Neuropathologica* vol. 82 239–259 (1991).
50. Petersen, R. C. *et al.* Alzheimer's Disease Neuroimaging Initiative (ADNI): Clinical characterization. *Neurology* **74**, 201–209 (2010).
51. Saykin, A. J. *et al.* Alzheimer's Disease Neuroimaging Initiative biomarkers as quantitative phenotypes: Genetics core aims, progress, and plans. *Alzheimer's Dement.* **6**, 265–273 (2010).
52. Albert, M. *et al.* Cognitive Changes Preceding Clinical Symptom Onset of Mild Cognitive Impairment and Relationship to ApoE Genotype. *Curr. Alzheimer Res.* **11**, 773–784 (2014).
53. Bienias, J. L., Beckett, L. A., Bennett, D. A., Wilson, R. S. & Evans, D. A. Design of the Chicago Health and Aging Project (CHAP). in *Journal of Alzheimer's Disease* vol. 5 349–355 (IOS Press, 2003).

54. Barzilai, N., Rossetti, L. & Lipton, R. B. Einstein's institute for aging research: Collaborative and programmatic approaches in the search for successful aging. *Exp. Gerontol.* **39**, 151–157 (2004).
55. Katz, M. J. *et al.* Age-specific and sex-specific prevalence and incidence of mild cognitive impairment, dementia, and alzheimer dementia in blacks and whites: A report from the Einstein aging study. *Alzheimer Dis. Assoc. Disord.* **26**, 335–343 (2012).
56. Li, H. *et al.* Candidate single-nucleotide polymorphisms from a genomewide association study of Alzheimer disease. *Arch. Neurol.* **65**, 45–53 (2008).
57. Green, R. C. *et al.* Risk of dementia among white and African American relatives of patients with Alzheimer disease. *J. Am. Med. Assoc.* **287**, 329–336 (2002).
58. Roccaforte, W. H., Burke, W. J., Bayer, B. L. & Wengel, S. P. Validation of a Telephone Version of the Mini-Mental State Examination. *J. Am. Geriatr. Soc.* **40**, 697–702 (1992).
59. Sims, R. *et al.* Rare coding variants in PLCG2, ABI3, and TREM2 implicate microglial-mediated innate immunity in Alzheimer's disease. *Nat. Genet.* **49**, 1373–1384 (2017).
60. Lee, J. H. *et al.* Analyses of the National Institute on Aging Late-Onset Alzheimer's Disease Family Study: implication of additional loci. *Arch. Neurol.* **65**, 1518–26 (2008).
61. Ravid, R. & Swaab, D. F. The Netherlands brain bank--a clinico-pathological link in aging and dementia research. *J. Neural Transm. Suppl.* **39**, 143–53 (1993).
62. Kramer, P. L. *et al.* Alzheimer disease pathology in cognitively healthy elderly: A genome-wide study. *Neurobiol. Aging* **32**, 2113–2122 (2011).
63. Bennett, D. A. *et al.* The rush memory and aging project: Study design and baseline characteristics of the study cohort. *Neuroepidemiology* **25**, 163–175 (2005).
64. Bennett, D. A. *et al.* Natural history of mild cognitive impairment in older persons. *Neurology* **59**, 198–205 (2002).
65. Bennett, D. A., Schneider, J. A., Bienias, J. L., Evans, D. A. & Wilson, R. S. Mild cognitive impairment is related to Alzheimer disease pathology and cerebral infarctions. *Neurology* **64**, 834–841 (2005).
66. Hall, J. R. *et al.* Biomarkers of vascular risk, systemic inflammation, and microvascular pathology and neuropsychiatric symptoms in Alzheimer's disease. *J. Alzheimer's Dis.* **35**, 363–371 (2013).
67. Reiman, E. M. *et al.* GAB2 Alleles Modify Alzheimer's Risk in APOE ε4 Carriers. *Neuron* **54**, 713–720 (2007).
68. Caselli, R. J. *et al.* Cognitive domain decline in healthy apolipoprotein E ε4 homozygotes before the diagnosis of mild cognitive impairment. *Arch. Neurol.* **64**, 1306–1311 (2007).
69. Webster, J. A. *et al.* Genetic Control of Human Brain Transcript Expression in Alzheimer Disease. *Am. J. Hum. Genet.* **84**, 445–458 (2009).
70. Petyuk, V. A. *et al.* The human brainome: Network analysis identifies HSPA2 as a novel Alzheimer's disease target. *Brain* **141**, 2721–2739 (2018).
71. Scott, W. K. *et al.* Complete genomic screen in parkinson disease evidence for multiple genes. *J. Am. Med. Assoc.* **286**, 2239–2244 (2001).
72. Beecham, G. W. *et al.* Genome-wide association study implicates a chromosome 12 risk locus for late-onset Alzheimer disease. *Am. J. Hum. Genet.* **84**, 35–43 (2009).
73. Edwards, T. L. *et al.* Genome-Wide association study confirms SNPs in SNCA and the MAPT region as common risk factors for parkinson disease. *Ann. Hum. Genet.* **74**, 97–109 (2010).
74. Naj, A. C. *et al.* Dementia revealed: novel chromosome 6 locus for late-onset Alzheimer disease provides genetic evidence for folate-pathway abnormalities. *PLoS Genet.* **6**, e1001130 (2010).
75. Haroutunian, V. *et al.* Regional distribution of neuritic plaques in the nondemented elderly and subjects with very mild Alzheimer Disease. *Arch. Neurol.* **55**, 1185–1191 (1998).
76. Kamboh, M. I. *et al.* Association of CLU and PICALM variants with Alzheimer's

disease. *Neurobiol. Aging* **33**, 518–521 (2012).
77. Hughes, C. P., Berg, L., Danziger, W. L., Coben, L. A. & Martin, R. L. A new clinical scale for the staging of dementia. *Br. J. Psychiatry* **140**, 566–572 (1982).
78. Tang, M. X. *et al.* Incidence of AD in African-Americans, Caribbean Hispanics, and Caucasians in northern Manhattan. *Neurology* **56**, 49–56 (2001).
79. Mayeux, R., Small, S. A., Tang, M. X., Tycko, B. & Stern, Y. Memory performance in healthy elderly without Alzheimer's disease: Effects of time and apolipoprotein-E. *Neurobiol. Aging* **22**, 683–689 (2001).
80. Price, A. L. *et al.* Principal components analysis corrects for stratification in genome-wide association studies. *Nat. Genet.* **38**, 904–909 (2006).
81. Patterson, N., Price, A. L. & Reich, D. Population structure and eigenanalysis. *PLoS Genet.* **2**, 2074–2093 (2006).
82. Fried, L. P. *et al.* The cardiovascular health study: Design and rationale. *Ann. Epidemiol.* **1**, 263–276 (1991).
83. Dawber, T. R. & Kannel, W. B. The Framingham study. An epidemiological approach to coronary heart disease. *Circulation* **34**, 553–555 (1966).
84. Feinleib, M., Kannel, W. B., Garrison, R. J., McNamara, P. M. & Castelli, W. P. The framingham offspring study. Design and preliminary data. *Prev. Med. (Baltim).* **4**, 518–525 (1975).
85. Kunkle, B. W. *et al.* Genetic meta-analysis of diagnosed Alzheimer's disease identifies new risk loci and implicates Aβ, tau, immunity and lipid processing. *Nat. Genet.* **51**, 414–430 (2019).
86. Van Der Flier, W. M. *et al.* Optimizing patient care and research: The Amsterdam dementia cohort. *J. Alzheimer's Dis.* **41**, 313–327 (2014).
87. Espinosa, A. *et al.* A longitudinal follow-up of 550 mild cognitive impairment patients: Evidence for large conversion to dementia rates and detection of major risk factors involved. *J. Alzheimer's Dis.* **34**, 769–780 (2013).
88. Hanon, O. *et al.* Plasma amyloid levels within the Alzheimer's process and correlations with central biomarkers. *Alzheimer's Dement.* **14**, 858–868 (2018).
89. Van Der Mussele, S. *et al.* Behavioral symptoms in mild cognitive impairment as compared with Alzheimer's disease and healthy older adults. *Int. J. Geriatr. Psychiatry* **28**, 265–275 (2013).
90. Petersen, R. C. *et al.* Mild cognitive impairment: Clinical characterization and outcome. *Arch. Neurol.* **56**, 303–308 (1999).
91. Luck, T. *et al.* Mild cognitive impairment in general practice: Age-specific prevalence and correlate results from the German study on ageing, cognition and dementia in primary care patients (AgeCoDe). *Dement. Geriatr. Cogn. Disord.* **24**, 307–316 (2007).
92. Fischer, P. *et al.* Vienna transdanube aging 'VITA': Study design, recruitment strategies and level of participation. in *Journal of Neural Transmission, Supplement* 105–116 (Springer Wien, 2002). doi:10.1007/978-3-7091-6139-5_11.
93. Numbers, K. *et al.* Participant and informant memory-specific cognitive complaints predict future decline and incident dementia: Findings from the Sydney memory and ageing study. *PLoS One* **15**, (2020).
94. de Bruijn, R. F. A. G. *et al.* The potential for prevention of dementia across two decades: The prospective, population-based Rotterdam Study. *BMC Med.* **13**, (2015).
95. Li, H. & Durbin, R. Fast and accurate short read alignment with Burrows-Wheeler transform. *Bioinformatics* **25**, 1754–1760 (2009).
96. Grove, M. L. *et al.* Best Practices and Joint Calling of the HumanExome BeadChip: The CHARGE Consortium. *PLoS One* **8**, (2013).
97. Price, A. L. *et al.* Long-Range LD Can Confound Genome Scans in Admixed Populations. *Am. J. Hum. Genet.* **83**, 132–135 (2008).
98. Abraham, G., Qiu, Y. & Inouye, M. FlashPCA2: principal component analysis of Biobank-scale genotype datasets. *Bioinformatics* **33**, 2776–2778 (2017).
99. Gogarten, S. M. *et al.* Genetic association testing using the GENESIS R/Bioconductor

package. *Bioinformatics* **35**, 5346–5348 (2019).
100. McCarthy, S. *et al.* A reference panel of 64,976 haplotypes for genotype imputation. *Nat. Genet.* **48**, 1279–83 (2016).
101. Karczewski, K. J. *et al.* The mutational constraint spectrum quantified from variation in 141,456 humans. *Nature* **581**, 434–443 (2020).
102. Taliun, D. *et al.* Sequencing of 53,831 diverse genomes from the NHLBI TOPMed Program. *bioRxiv* **2**, 563866 (2019).
103. Marchini, J., Howie, B., Myers, S., McVean, G. & Donnelly, P. A new multipoint method for genome-wide association studies by imputation of genotypes. *Nat. Genet.* **39**, 906–13 (2007).
104. Manichaikul, A. *et al.* Robust relationship inference in genome-wide association studies. *Bioinformatics* **26**, 2867–2873 (2010).
105. Das, S. *et al.* Next-generation genotype imputation service and methods. *Nat. Genet.* **48**, 1284–1287 (2016).
106. Loh, P. R. *et al.* Reference-based phasing using the Haplotype Reference Consortium panel. *Nat. Genet.* **48**, 1443–1448 (2016).
107. Purcell, S. *et al.* PLINK: A tool set for whole-genome association and population-based linkage analyses. *Am. J. Hum. Genet.* **81**, 559–575 (2007).
108. Chang, C. C. *et al.* Second-generation PLINK: rising to the challenge of larger and richer datasets. *Gigascience* **4**, 7 (2015).
109. Chang, C. C. Data management and summary statistics with PLINK. in *Methods in Molecular Biology* vol. 2090 49–65 (Humana Press Inc., 2020).
110. Chen, M. H. & Yang, Q. GWAF: An R package for genome-wide association analyses with family data. *Bioinformatics* vol. 26 580–581 (2009).
111. Willer, C. J., Li, Y. & Abecasis, G. R. METAL: fast and efficient meta-analysis of genomewide association scans. *Bioinformatics* **26**, 2190–1 (2010).
112. Voorman, A., Brody, J., Chen, H., Lumley, T. & Davis, B. seqMeta: Meta-Analysis of Region-Based Tests of Rare DNA Variants. (2017).
113. R Core Team. R: A Language and Environment for Statistical Computing. (2020).
114. Lopez, O. L. *et al.* Evaluation of dementia in the Cardiovascular Health Cognition Study. *Neuroepidemiology* **22**, 1–12 (2003).
115. Zhou, W. *et al.* Efficiently controlling for case-control imbalance and sample relatedness in large-scale genetic association studies. *Nat. Genet.* **50**, 1335–1341 (2018).
116. Yang, J. *et al.* Conditional and joint multiple-SNP analysis of GWAS summary statistics identifies additional variants influencing complex traits. *Nat. Genet.* **44**, 369–75, S1-3 (2012).
117. Yang, J., Lee, S. H., Goddard, M. E. & Visscher, P. M. GCTA: a tool for genome-wide complex trait analysis. *Am. J. Hum. Genet.* **88**, 76–82 (2011).
118. Ruiz, A. *et al.* Follow-up of loci from the International Genomics of Alzheimer's Disease Project identifies TRIP4 as a novel susceptibility gene. *Transl. Psychiatry* **4**, e358 (2014).
119. Schwartzentruber, J. *et al.* Genome-wide meta-analysis, fine-mapping, and integrative prioritization identify new Alzheimer's disease risk genes. *medRxiv* 2020.01.22.20018424 (2020) doi:10.1101/2020.01.22.20018424.
120. de Rojas, I. *et al.* Common variants in Alzheimer's disease: Novel association of six genetic variants with AD and risk stratification by polygenic risk scores. *medRxiv* 19012021 (2019) doi:10.1101/19012021.
121. Zheng, X. *et al.* HIBAG - HLA genotype imputation with attribute bagging. *Pharmacogenomics J.* **14**, 192–200 (2014).
122. Jones, E. *et al.* Identification of novel risk loci and causal insights for sporadic Creutzfeldt-Jakob disease: a genome-wide association study. *Lancet Neurol.* **19**, 840–848 (2020).
123. Rongve, A. *et al.* GBA and APOE ε4 associate with sporadic dementia with Lewy bodies in European genome wide association study. *Sci. Rep.* **9**, (2019).

124. Nicolas, A. *et al.* Genome-wide Analyses Identify KIF5A as a Novel ALS Gene. *Neuron* **97**, 1268-1283.e6 (2018).
125. Ferrari, R. *et al.* Frontotemporal dementia and its subtypes: A genome-wide association study. *Lancet Neurol.* **13**, 686–699 (2014).
126. Nalls, M. A. *et al.* Identification of novel risk loci, causal insights, and heritable risk for Parkinson's disease: a meta-analysis of genome-wide association studies. *Lancet Neurol.* **18**, 1091–1102 (2019).
127. Chauhan, G. *et al.* Genetic and lifestyle risk factors for MRI-defined brain infarcts in a population-based setting. *Neurology* **92**, E486–E503 (2019).
128. Malik, R. *et al.* Multiancestry genome-wide association study of 520,000 subjects identifies 32 loci associated with stroke and stroke subtypes. *Nat. Genet.* **50**, 524–537 (2018).
129. Armstrong, N. J. *et al.* Common Genetic Variation Indicates Separate Causes for Periventricular and Deep White Matter Hyperintensities. *Stroke* **51**, 2111–2121 (2020).
130. Elsworth, B. *et al.* The MRC IEU OpenGWAS data infrastructure. *bioRxiv* 2020.08.10.244293 (2020) doi:10.1101/2020.08.10.244293.
131. Hemani, G. *et al.* The MR-base platform supports systematic causal inference across the human phenome. *Elife* **7**, (2018).
132. Yang, J. *et al.* Conditional and joint multiple-SNP analysis of GWAS summary statistics identifies additional variants influencing complex traits. *Nat. Genet.* **44**, 369–75, S1-3 (2012).
133. Benner, C. *et al.* Prospects of Fine-Mapping Trait-Associated Genomic Regions by Using Summary Statistics from Genome-wide Association Studies. *Am. J. Hum. Genet.* **101**, 539–551 (2017).
134. Giambartolomei, C. *et al.* Bayesian Test for Colocalisation between Pairs of Genetic Association Studies Using Summary Statistics. *PLoS Genet.* **10**, (2014).
135. Ferrari, R. *et al.* Frontotemporal dementia and its subtypes: A genome-wide association study. *Lancet Neurol.* **13**, 686–699 (2014).
136. Van Deerlin, V. M. *et al.* Common variants at 7p21 are associated with frontotemporal lobar degeneration with TDP-43 inclusions. *Nat. Genet.* **42**, 234–239 (2010).
137. Allen, M. *et al.* Human whole genome genotype and transcriptome data for Alzheimer's and other neurodegenerative diseases. *Sci. Data* **3**, (2016).
138. Mostafavi, S. *et al.* A molecular network of the aging human brain provides insights into the pathology and cognitive decline of Alzheimer's disease. *Nat. Neurosci.* **21**, 811–819 (2018).
139. Bennett, D. A. *et al.* Religious Orders Study and Rush Memory and Aging Project. *Journal of Alzheimer's Disease* vol. 64 S161–S189 (2018).
140. Wang, M. *et al.* The Mount Sinai cohort of large-scale genomic, transcriptomic and proteomic data in Alzheimer's disease. *Sci. Data* **5**, 180185 (2018).
141. McKenna, A. *et al.* The Genome Analysis Toolkit: a MapReduce framework for analyzing next-generation DNA sequencing data. *Genome Res.* **20**, 1297–303 (2010).
142. Lindenbaum, P. & Redon, R. Bioalcidae, samjs and vcffilterjs: Object-oriented formatters and filters for bioinformatics files. *Bioinformatics* **34**, 1224–1225 (2018).
143. Dobin, A. *et al.* STAR: Ultrafast universal RNA-seq aligner. *Bioinformatics* **29**, 15–21 (2013).
144. Consortium, T. Gte. *The GTEx Consortium atlas of genetic regulatory effects across human tissues The GTEx Consortium* Downloaded from. Science* vol. 369 http://science.sciencemag.org/ (2020).
145. Deluca, D. S. *et al.* RNA-SeQC: RNA-seq metrics for quality control and process optimization. *Bioinformatics* **28**, 1530–1532 (2012).
146. Robinson, M. D. & Oshlack, A. A scaling normalization method for differential expression analysis of RNA-seq data. *Genome Biol.* **11**, (2010).
147. Stegle, O., Parts, L., Durbin, R. & Winn, J. A bayesian framework to account for complex non-genetic factors in gene expression levels greatly increases power in

eQTL studies. *PLoS Comput. Biol.* **6**, 1–11 (2010).
148. Li, Y. I. *et al.* Annotation-free quantification of RNA splicing using LeafCutter. *Nat. Genet.* **50**, 151–158 (2018).
149. Feng, Y.-Y. *et al.* RegTools: Integrated analysis of genomic and transcriptomic data for discovery of splicing variants in cancer. *bioRxiv* 436634 (2018) doi:10.1101/436634.
150. Ongen, H., Buil, A., Brown, A. A., Dermitzakis, E. T. & Delaneau, O. Fast and efficient QTL mapper for thousands of molecular phenotypes. *Bioinformatics* **32**, 1479–1485 (2016).
151. Storey, J. D. & Tibshirani, R. Statistical significance for genomewide studies. *Proc. Natl. Acad. Sci. U. S. A.* **100**, 9440–9445 (2003).
152. De, K. *et al.* Atlas of genetic effects in human microglia transcriptome across brain regions, aging and disease pathologies. *bioRxiv* 2020.10.27.356113 (2020) doi:10.1101/2020.10.27.356113.
153. Han, B. & Eskin, E. Random-effects model aimed at discovering associations in meta-analysis of genome-wide association studies. *Am. J. Hum. Genet.* **88**, 586–598 (2011).
154. Võsa, U. *et al.* Unraveling the polygenic architecture of complex traits using blood eQTL meta-analysis. *bioRxiv* vol. 18 10 (2018).
155. Alasoo, K. *et al.* Shared genetic effects on chromatin and gene expression indicate a role for enhancer priming in immune response. *Nat. Genet.* **50**, 424–431 (2018).
156. Nédélec, Y. *et al.* Genetic Ancestry and Natural Selection Drive Population Differences in Immune Responses to Pathogens. *Cell* **167**, 657-669.e21 (2016).
157. Chen, L. *et al.* Genetic Drivers of Epigenetic and Transcriptional Variation in Human Immune Cells. *Cell* **167**, 1398-1414.e24 (2016).
158. Momozawa, Y. *et al.* IBD risk loci are enriched in multigenic regulatory modules encompassing putative causative genes. *Nat. Commun.* **9**, (2018).
159. Fairfax, B. P. *et al.* Innate immune activity conditions the effect of regulatory variants upon monocyte gene expression. *Science (80-.).* **343**, (2014).
160. Quach, H. *et al.* Genetic Adaptation and Neandertal Admixture Shaped the Immune System of Human Populations. *Cell* **167**, 643-656.e17 (2016).
161. Kerimov, N. *et al.* eQTL catalogue: A compendium of uniformly processed human gene expression and splicing QTLs. *bioRxiv* 2020.01.29.924266 (2020) doi:10.1101/2020.01.29.924266.
162. Ng, B. *et al.* An xQTL map integrates the genetic architecture of the human brain's transcriptome and epigenome. *Nat. Neurosci.* **20**, 1418–1426 (2017).
163. Lim, A. S. P. *et al.* Diurnal and seasonal molecular rhythms in human neocortex and their relation to Alzheimer's disease. *Nat. Commun.* **8**, (2017).
164. De Jager, P. L. *et al.* Alzheimer's disease: early alterations in brain DNA methylation at ANK1, BIN1, RHBDF2 and other loci. *Nat. Neurosci.* **17**, 1156–63 (2014).
165. Gusev, A. *et al.* Integrative approaches for large-scale transcriptome-wide association studies. *Nat. Genet.* **48**, 245–52 (2016).
166. Lowy-Gallego, E. *et al.* Variant calling on the GRCh38 assembly with the data from phase three of the 1000 Genomes Project. *Wellcome Open Res.* **4**, 50 (2019).
167. Auton, A. *et al.* A global reference for human genetic variation. *Nature* vol. 526 68–74 (2015).
168. Barbeira, A. N. *et al.* Exploiting the GTEx resources to decipher the mechanisms at GWAS loci. *bioRxiv* 814350 (2020) doi:10.1101/814350.
169. Barbeira, A. N. *et al.* Exploring the phenotypic consequences of tissue specific gene expression variation inferred from GWAS summary statistics. *Nat. Commun.* **9**, (2018).
170. Gamazon, E. R. *et al.* A gene-based association method for mapping traits using reference transcriptome data. *Nat. Genet.* **47**, 1091–1098 (2015).
171. Mancuso, N. *et al.* Probabilistic fine-mapping of transcriptome-wide association studies. *Nat. Genet.* **51**, 675–682 (2019).

172. Jain, M., Olsen, H. E., Paten, B. & Akeson, M. The Oxford Nanopore MinION: delivery of nanopore sequencing to the genomics community. *Genome Biol.* **17**, (2016).
173. Untergasser, A. *et al.* Primer3-new capabilities and interfaces. *Nucleic Acids Res.* **40**, (2012).
174. De Roeck, A. *et al.* Deleterious ABCA7 mutations and transcript rescue mechanisms in early onset Alzheimer's disease. *Acta Neuropathol.* **134**, 475–487 (2017).
175. Li, H. Minimap2: Pairwise alignment for nucleotide sequences. *Bioinformatics* **34**, 3094–3100 (2018).
176. De Coster, W., D'Hert, S., Schultz, D. T., Cruts, M. & Van Broeckhoven, C. NanoPack: Visualizing and processing long-read sequencing data. *Bioinformatics* **34**, 2666–2669 (2018).
177. Thorvaldsdóttir, H., Robinson, J. T. & Mesirov, J. P. Integrative Genomics Viewer (IGV): High-performance genomics data visualization and exploration. *Brief. Bioinform.* **14**, 178–192 (2013).
178. Pedersen, B. S. & Quinlan, A. R. Mosdepth: Quick coverage calculation for genomes and exomes. *Bioinformatics* **34**, 867–868 (2018).
179. Freytag, V. *et al.* Genetic estimators of DNA methylation provide insights into the molecular basis of polygenic traits. *Transl. Psychiatry* **8**, (2018).
180. Edgar, R. D., Jones, M. J., Meaney, M. J., Turecki, G. & Kobor, M. S. BECon: A tool for interpreting DNA methylation findings from blood in the context of brain. *Transl. Psychiatry* **7**, e1187–e1187 (2017).
181. Wingo, A. P. *et al.* Integrating human brain proteomes with genome-wide association data implicates new proteins in Alzheimer's disease pathogenesis. *Nat. Genet.* **53**, 143–146 (2021).
182. Rentzsch, P., Schubach, M., Shendure, J. & Kircher, M. CADD-Splice—improving genome-wide variant effect prediction using deep learning-derived splice scores. *Genome Med.* **13**, (2021).
183. Chapuis, J. *et al.* Genome-wide, high-content siRNA screening identifies the Alzheimer's genetic risk factor FERMT2 as a major modulator of APP metabolism. *Acta Neuropathol.* **133**, 955–966 (2017).
184. Sannerud, R. *et al.* ADP ribosylation factor 6 (ARF6) controls amyloid precursor protein (APP) processing by mediating the endosomal sorting of BACE1. *Proc. Natl. Acad. Sci. U. S. A.* **108**, (2011).
185. Wu, M. C. *et al.* Rare-variant association testing for sequencing data with the sequence kernel association test. *Am. J. Hum. Genet.* **89**, 82–93 (2011).
186. Lee, S. *et al.* Optimal unified approach for rare-variant association testing with application to small-sample case-control whole-exome sequencing studies. *Am. J. Hum. Genet.* **91**, 224–37 (2012).
187. Li, X. *et al.* Transcriptome sequencing of a large human family identifies the impact of rare noncoding variants. *Am. J. Hum. Genet.* **95**, 245–256 (2014).
188. Li, X. *et al.* The impact of rare variation on gene expression across tissues. *Nature* **550**, 239–243 (2017).
189. Aguet, F. *et al.* Transcriptomic signatures across human tissues identify functional rare genetic variation. *Science (80-.).* **369**, (2020).
190. Li, J., Kong, N., Han, B. & Sul, J. H. Rare variants regulate expression of nearby individual genes in multiple tissues. *PLoS Genet.* **17**, (2021).
191. Zheng, Y., Brady, O. A., Meng, P. S., Mao, Y. & Hu, F. C-terminus of progranulin interacts with the beta-propeller region of sortilin to regulate progranulin trafficking. *PLoS One* **6**, (2011).
192. Philtjens, S. *et al.* Rare nonsynonymous variants in SORT1 are associated with increased risk for frontotemporal dementia. *Neurobiol. Aging* **66**, 181.e3-181.e10 (2018).
193. Wahrle, S. E. *et al.* Overexpression of ABCA1 reduces amyloid deposition in the PDAPP mouse model of Alzheimer disease. *J. Clin. Invest.* **118**, 671–682 (2008).
194. Holstege, H. *et al.* Exome sequencing identifies novel AD-associated genes. *medRxiv*

18, 24 (2020).
195. Saint-Pol, J. *et al.* Regulation of the trafficking and the function of the metalloprotease ADAM10 by tetraspanins. *Biochemical Society Transactions* vol. 45 937–944 (2017).
196. Nott, A. *et al.* Brain cell type–specific enhancer–promoter interactome maps and disease-risk association. *Science (80-.).* **366**, 1134–1139 (2019).
197. Takahashi, H. *et al.* Opposing effects of progranulin deficiency on amyloid and tau pathologies via microglial TYROBP network. *Acta Neuropathol.* **133**, 785–807 (2017).
198. Brouwers, N. *et al.* Genetic variability in progranulin contributes to risk for clinically diagnosed Alzheimer disease. *Neurology* **71**, 656–664 (2008).
199. Brouwers, N. *et al.* Alzheimer and Parkinson diagnoses in progranulin null mutation carriers in an extended founder family. *Arch. Neurol.* **64**, 1436–1446 (2007).
200. Kim, T. *et al.* Human LilrB2 is a β-amyloid receptor and its murine homolog PirB regulates synaptic plasticity in an Alzheimer's model. *Science (80-.).* **341**, 1399–1404 (2013).
201. Cao, Q. *et al.* Inhibiting amyloid-ß cytotoxicity through its interaction with the cell surface receptor LilrB2 by structure-based design. *Nat. Chem.* **10**, (2018).
202. Cleynen, A. *et al.* Expressed fusion gene landscape and its impact in multiple myeloma. *Nat. Commun.* **8**, (2017).
203. Conlon, D. M. Role of sortilin in lipid metabolism. *Current Opinion in Lipidology* vol. 30 198–204 (2019).
204. Musunuru, K. *et al.* From noncoding variant to phenotype via SORT1 at the 1p13 cholesterol locus. *Nature* **466**, 714–9 (2010).
205. Ruan, C. S. *et al.* Sortilin inhibits amyloid pathology by regulating non-specific degradation of APP. *Exp. Neurol.* **299**, 75–85 (2018).
206. Hu, F. *et al.* Sortilin-mediated endocytosis determines levels of the frontotemporal dementia protein, progranulin. *Neuron* **68**, 654–667 (2010).
207. Kim, C., Ye, F. & Ginsberg, M. H. Regulation of integrin activation. *Annu. Rev. Cell Dev. Biol.* **27**, 321–345 (2011).
208. Qian, M., Shen, X. & Wang, H. The Distinct Role of ADAM17 in APP Proteolysis and Microglial Activation Related to Alzheimer's Disease. *Cellular and Molecular Neurobiology* vol. 36 471–482 (2016).
209. Feuerbach, D. *et al.* ADAM17 is the main sheddase for the generation of human triggering receptor expressed in myeloid cells (hTREM2) ectodomain and cleaves TREM2 after Histidine 157. *Neurosci. Lett.* **660**, 109–114 (2017).
210. Lisi, S., D'Amore, M. & Sisto, M. ADAM17 at the interface between inflammation and autoimmunity. *Immunology Letters* vol. 162 159–169 (2014).
211. Hartl, D. *et al.* A rare loss-of-function variant of ADAM17 is associated with late-onset familial Alzheimer disease. *Mol. Psychiatry* **25**, 629–639 (2020).
212. Xie, M. *et al.* PKM2-Dependent glycolysis promotes NLRP3 and AIM2 inflammasome activation. *Nat. Commun.* **7**, (2016).
213. Liu, Y., Zhou, Y., Ma, X. & Chen, L. Inhibition Lysosomal Degradation of Clusterin by Protein Kinase D3 Promotes Triple-Negative Breast Cancer Tumor Growth. *Adv. Sci.* **8**, (2021).
214. Lal, H. *et al.* Integrins and proximal signaling mechanisms in cardiovascular disease. *Front. Biosci.* **14**, 2307–2334 (2009).
215. Round, J. E. & Sun, H. The adaptor protein Nck2 mediates Slit1-induced changes in cortical neuron morphology. *Mol. Cell. Neurosci.* **47**, 265–273 (2011).
216. Thévenot, E. *et al.* p21-activated kinase 3 (PAK3) protein regulates synaptic transmission through its interaction with the Nck2/Grb4 protein adaptor. *J. Biol. Chem.* **286**, 40044–40059 (2011).
217. Chung, J. *et al.* Genome-wide association study of cerebral small vessel disease reveals established and novel loci. *Brain* **142**, 3176–3189 (2019).
218. Sikanyika, N. L., Parkington, H. C., Smith, A. I. & Kuruppu, S. Powering Amyloid Beta Degrading Enzymes: A Possible Therapy for Alzheimer's Disease. *Neurochem. Res.* **44**, 1289–1296 (2019).

219. Tu-Sekine, B. & Raben, D. M. Regulation of DGK-$θ$. *Journal of Cellular Physiology* vol. 220 548–552 (2009).
220. Nurrish, S., Ségalat, L. & Kaplan, J. M. Serotonin inhibition of synaptic transmission: g$α$(o) decreases the abundance of unc-13 at release sites. *Neuron* **24**, 231–242 (1999).
221. Viana, G. M. *et al.* Cathepsin b-associated activation of amyloidogenic pathway in murine mucopolysaccharidosis type i brain cortex. *Int. J. Mol. Sci.* **21**, (2020).
222. Troeger, A. & Williams, D. A. Hematopoietic-specific Rho GTPases Rac2 and RhoH and human blood disorders. *Experimental Cell Research* vol. 319 2375–2383 (2013).
223. Damgaard, R. B. *et al.* The Deubiquitinase OTULIN Is an Essential Negative Regulator of Inflammation and Autoimmunity. *Cell* **166**, 1215-1230.e20 (2016).
224. Fiil, B. K. & Gyrd-Hansen, M. OTULIN deficiency causes auto-inflammatory syndrome. *Cell Research* vol. 26 1176–1177 (2016).
225. Xu, H. *et al.* Lentivirus-mediated overexpression of OTULIN ameliorates microglia activation and neuroinflammation by depressing the activation of the NF-KB signaling pathway in cerebral ischemia/reperfusion rats. *J. Neuroinflammation* **15**, (2018).
226. Spit, M., Rieser, E. & Walczak, H. Linear ubiquitination at a glance. *J. Cell Sci.* **132**, (2019).
227. Stangl, A. *et al.* Regulation of the endosomal SNX27-retromer by OTULIN. *Nat. Commun.* **10**, (2019).
228. Huang, T. Y. *et al.* SNX27 and SORLA interact to reduce amyloidogenic subcellular distribution and processing of amyloid precursor protein. *J. Neurosci.* **36**, 7996–8011 (2016).
229. Wang, X. *et al.* Sorting nexin 27 regulates Aβ production through modulating γ-secretase activity. *Cell Rep.* **9**, 1023–1033 (2014).
230. Benyamin, B. *et al.* Cross-ethnic meta-analysis identifies association of the GPX3-TNIP1 locus with amyotrophic lateral sclerosis. *Nat. Commun.* **8**, (2017).
231. Chen, J., Yuan, F., Fan, X. & Wang, Y. Psoriatic arthritis: A systematic review of non-HLA genetic studies and important signaling pathways. *International Journal of Rheumatic Diseases* vol. 23 1288–1296 (2020).
232. Pannekoek, W. J., Linnemann, J. R., Brouwer, P. M., Bos, J. L. & Rehmann, H. Rap1 and Rap2 Antagonistically Control Endothelial Barrier Resistance. *PLoS One* **8**, (2013).
233. Patey, S. J. The role of heparan sulfate in the generation of Aβ. *Drug News Perspect.* **19**, 411–416 (2006).
234. Holmes, B. B. *et al.* Heparan sulfate proteoglycans mediate internalization and propagation of specific proteopathic seeds. *Proc. Natl. Acad. Sci. U. S. A.* **110**, (2013).
235. Zhang, X., Wang, B. & Li, J. P. Implications of heparan sulfate and heparanase in neuroinflammation. *Matrix Biology* vol. 35 174–181 (2014).
236. Pietropaolo, M. *et al.* Islet cell autoantigen 69 kD (ICA69): Molecular cloning and characterization of a novel diabetes-associated autoantigen. *J. Clin. Invest.* **92**, 359–371 (1993).
237. Cao, M. *et al.* PICK1-ICA69 heteromeric BAR domain complex regulates synaptic targeting and surface expression of AMPA receptors. *J. Neurosci.* **27**, 12945–12956 (2007).
238. Zhou, X. *et al.* Loss of TMEM106B leads to myelination deficits: implications for frontotemporal dementia treatment strategies. *Brain* **143**, 1905–1919 (2020).
239. Schwenk, B. M. *et al.* The FTLD risk factor TMEM106B and MAP6 control dendritic trafficking of lysosomes. *EMBO J.* **33**, 450–467 (2014).
240. Suzuki, H. & Matsuoka, M. The lysosomal trafficking transmembrane protein 106B is linked to cell death. *J. Biol. Chem.* **291**, 21448–21460 (2016).
241. Liao, Z. Z., Wang, Y. Di, Qi, X. Y. & Xiao, X. H. JAZF1, a relevant metabolic regulator in type 2 diabetes. *Diabetes/Metabolism Research and Reviews* vol. 35 (2019).
242. Stupack, J. *et al.* Soluble SORLA enhances neurite outgrowth and regeneration

through activation of the EGF Receptor/ERK signaling axis. *J. Neurosci.* **40**, JN-RM-0723-20 (2020).
243. Repetto, E., Yoon, I. S., Zheng, H. & Kang, D. E. Presenilin 1 regulates epidermal growth factor receptor turnover and signaling in the endosomal-lysosomal pathway. *J. Biol. Chem.* **282**, 31504–31516 (2007).
244. Wang, L. *et al.* Epidermal growth factor receptor is a preferred target for treating Amyloid-β-induced memory loss. *Proc. Natl. Acad. Sci. U. S. A.* **109**, 16743–16748 (2012).
245. Hook, G., Yu, J., Toneff, T., Kindy, M. & Hook, V. Brain pyroglutamate amyloid-β is produced by cathepsin b and is reduced by the cysteine protease inhibitor E64d, representing a potential alzheimer's disease therapeutic. *J. Alzheimer's Dis.* **41**, 129–149 (2014).
246. Wang, C., Sun, B., Zhou, Y., Grubb, A. & Gan, L. Cathepsin B degrades amyloid-β in mice expressing wild-type human amyloid precursor protein. *J. Biol. Chem.* **287**, 39834–39841 (2012).
247. Bai, H. *et al.* Cathepsin B links oxidative stress to the activation of NLRP3 inflammasome. *Exp. Cell Res.* **362**, 180–187 (2018).
248. Asanomi, Y. *et al.* A rare functional variant of SHARPIN attenuates the inflammatory response and associates with increased risk of late-onset Alzheimer's disease. *Mol. Med.* **25**, 20 (2019).
249. Soheili-Nezhad, S. *et al.* Imaging genomics discovery of a new risk variant for Alzheimer's disease in the postsynaptic SHARPIN gene. *Hum. Brain Mapp.* **41**, (2020).
250. Lim, S. *et al.* Sharpin, a novel postsynaptic density protein that directly interacts with the shank family of proteins. *Mol. Cell. Neurosci.* **17**, 385–397 (2001).
251. Rantala, J. K. *et al.* SHARPIN is an endogenous inhibitor of $β$1-integrin activation. *Nat. Cell Biol.* **13**, 1315–1324 (2011).
252. Fitz, N. F. *et al.* ABCA1 Deficiency Affects Basal Cognitive Deficits and Dendritic Density in Mice. *J. Alzheimer's Dis.* **56**, 1075–1085 (2017).
253. Cui, X. *et al.* Deficiency of brain ATP-binding cassette transporter A-1 exacerbates blood-brain barrier and white matter damage after stroke. *Stroke* **46**, 827–834 (2015).
254. Laxmi, A., Gupta, P. & Gupta, J. CCDC6, a gene product in fusion with different protoncogenes, as a potential chemotherapeutic target. *Cancer Biomarkers* vol. 24 383–393 (2019).
255. Beyer, T. *et al.* CRISPR/Cas9-mediated genomic editing of Cluap1/IFT38 reveals a new role in actin arrangement. *Mol. Cell. Proteomics* **17**, 1285–1294 (2018).
256. Santuccione, A. C. *et al.* Active vaccination with ankyrin G reduces β-amyloid pathology in APP transgenic mice. *Mol. Psychiatry* **18**, 358–368 (2013).
257. Tsukada, S., Baba, Y. & Watanabe, D. Btk and BLNK in B cell development. *Adv. Immunol.* **77**, 123–162 (2001).
258. Wang, J., Sohn, H., Sun, G., Milner, J. D. & Pierce, S. K. The autoinhibitory C-terminal SH2 domain of phospholipase C-γ2 stabilizes B cell receptor signalosome assembly. *Sci. Signal.* **7**, (2014).
259. Sierksma, A. *et al.* Novel Alzheimer risk genes determine the microglia response to amyloid-β but not to TAU pathology. *EMBO Mol. Med.* **12**, (2020).
260. Jakobsdottir, J. *et al.* Susceptibility genes for age-related maculopathy on chromosome 10q26. *Am. J. Hum. Genet.* **77**, 389–407 (2005).
261. Wacker, S. A. *et al.* RITA, a novel modulator of Notch signalling, acts via nuclear export of RBP-J. *EMBO J.* **30**, 43–56 (2011).
262. Schwamb, B. *et al.* FAM96A is a novel pro-apoptotic tumor suppressor in gastrointestinal stromal tumors. *Int. J. Cancer* **137**, 1318–1329 (2015).
263. Nielsen, M. S. *et al.* Sorting by the Cytoplasmic Domain of the Amyloid Precursor Protein Binding Receptor SorLA. *Mol. Cell. Biol.* **27**, 6842–6851 (2007).
264. Fan, K. *et al.* The induction of neuronal death by up-regulated microglial cathepsin H in LPS-induced neuroinflammation. *J. Neuroinflammation* **12**, (2015).

265. Groffen, A. J. A., Friedrich, R., Brian, E. C., Ashery, U. & Verhage, M. DOC2A and DOC2B are sensors for neuronal activity with unique calcium-dependent and kinetic properties. *J. Neurochem.* **97**, 818–833 (2006).
266. Glessner, J. T. *et al.* Strong synaptic transmission impact by copy number variations in schizophrenia. *Proc. Natl. Acad. Sci. U. S. A.* **107**, 10584–10589 (2010).
267. Su, W. *et al.* The p53 Transcription Factor Modulates Microglia Behavior through MicroRNA-Dependent Regulation of c-Maf. *J. Immunol.* **192**, 358–366 (2014).
268. Bagella, L., Giacinti, C., Simone, C. & Giordano, A. Identification of murine cdk10: Association with Ets2 transcription factor and effects on the cell cycle. *J. Cell. Biochem.* **99**, 978–985 (2006).
269. Yeh, C. W., Kao, S. H., Cheng, Y. C. & Hsu, L. S. Knockdown of cyclin-dependent kinase 10 (cdk10) gene impairs neural progenitor survival via modulation of raf1a gene expression. *J. Biol. Chem.* **288**, 27927–27939 (2013).
270. Blazer, L. L. *et al.* PR domain-containing protein 7 (PRDM7) is a histone 3 lysine 4 trimethyltransferase. *J. Biol. Chem.* **291**, 13509–13519 (2016).
271. Rapiteanu, R. *et al.* A Genetic Screen Identifies a Critical Role for the WDR81-WDR91 Complex in the Trafficking and Degradation of Tetherin. *Traffic* **17**, 940–958 (2016).
272. Wang, M. *et al.* WDR81 regulates adult hippocampal neurogenesis through endosomal SARA-TGFβ signaling. *Mol. Psychiatry* (2018) doi:10.1038/s41380-018-0307-y.
273. Farjami, M. *et al.* The worldwide frequency of MYO15A gene mutations in patients with non-syndromic hearing loss: A meta-analysis. *Iran. J. Basic Med. Sci.* **23**, 841–848 (2020).
274. Jossin, Y. *et al.* Llgl1 Connects Cell Polarity with Cell-Cell Adhesion in Embryonic Neural Stem Cells. *Dev. Cell* **41**, 481-495.e5 (2017).
275. Chauhan, M., Sharma, G., Joshi, G. & Kumar, R. Epidermal Growth Factor Receptor (EGFR) and its Cross-Talks with Topoisomerases: Challenges and Opportunities for Multi-Target Anticancer Drugs. *Curr. Pharm. Des.* **22**, 3226–3236 (2016).
276. King, I. F. *et al.* Topoisomerases facilitate transcription of long genes linked to autism. *Nature* **501**, 58–62 (2013).
277. Takahashi, H. *et al.* Opposing effects of progranulin deficiency on amyloid and tau pathologies via microglial TYROBP network. *Acta Neuropathol.* **133**, 785–807 (2017).
278. Kaczynski, J. A. *et al.* Functional analysis of basic transcription element (BTE)-binding protein (BTEB) 3 and BTEB4, a novel Sp1-like protein, reveals a subfamily of transcriptional repressors for the BTE site of the cytochrome P4501A1 gene promoter. *Biochem. J.* **366**, 873–882 (2002).
279. Suh, Y. *et al.* Dopamine D1 Receptor (D1R) Expression Is Controlled by a Transcriptional Repressor Complex Containing DISC1. *Mol. Neurobiol.* **56**, 6725–6735 (2019).
280. Salminen, A. & Kaarniranta, K. Siglec receptors and hiding plaques in Alzheimer's disease. *Journal of Molecular Medicine* vol. 87 697–701 (2009).
281. Huan, T. *et al.* A Meta-analysis of Gene Expression Signatures of Blood Pressure and Hypertension. *PLoS Genet.* **11**, (2015).
282. Ahn, E., Lee, H. & Yun, Y. LIME acts as a transmembrane adapter mediating BCR-dependent B-cell activation. *Blood* **107**, 1521–1527 (2006).
283. Russo, C. *et al.* The amyloid precursor protein and its network of interacting proteins: Physiological and pathological implications. in *Brain Research Reviews* vol. 48 257–264 (Elsevier, 2005).
284. Szklarczyk, D. *et al.* STRING v11: Protein-protein association networks with increased coverage, supporting functional discovery in genome-wide experimental datasets. *Nucleic Acids Res.* **47**, D607--D613 (2019).
285. Heagerty, P. J. & Zheng, Y. Survival model predictive accuracy and ROC curves. *Biometrics* **61**, 92–105 (2005).
286. Mogensen, U. B., Ishwaran, H. & Gerds, T. A. Evaluating Random Forests for

Survival Analysis Using Prediction Error Curves. *J. Stat. Softw.* **50**, (2012).
287. Gerds, T. A., Kattan, M. W., Schumacher, M. & Yu, C. Estimating a time-dependentconcordance index for survival prediction models with covariate dependent censoring. *Stat. Med.* **32**, 2173–2184 (2013).
288. Pencina, M. J., D'Agostino, R. B., Pencina, K. M., Janssens, A. C. J. W. & Greenland, P. Interpreting incremental value of markers added to risk prediction models. *Am. J. Epidemiol.* **176**, 473–481 (2012).
289. Pennells, L. *et al.* Assessing risk prediction models using individual participant data from multiple studies. *Am. J. Epidemiol.* **179**, 621–632 (2014).
290. Leening, M. J. G., Vedder, M. M., Witteman, J. C. M., Pencina, M. J. & Steyerberg, E. W. Net reclassification improvement: Computation, interpretation, and controversies: A literature review and clinician's guide. *Annals of Internal Medicine* vol. 160 122–131 (2014).
291. Uno, H. & Cai, T. survIDINRI: IDI and NRI for comparing competing risk prediction models with censored survival data. (2013).
292. Uno, H., Tian, L., Cai, T., Kohane, I. S. & Wei, L. J. A unified inference procedure for a class of measures to assess improvement in risk prediction systems with survival data. *Stat. Med.* **32**, 2430–2442 (2013).
293. Steyerberg, E. W. *et al.* Assessing the performance of prediction models: A framework for traditional and novel measures. *Epidemiology* vol. 21 128–138 (2010).
294. Kattan, M. W. & Gerds, T. A. The index of prediction accuracy: an intuitive measure useful for evaluating risk prediction models. *Diagnostic Progn. Res.* **2**, (2018).
295. Gerds, T. A. & Ozenne, B. riskRegression: Risk regression models and prediction scores for survival analysis with competing risks. (2019).
296. Pepe, M. S., Kerr, K. F., Longton, G. & Wang, Z. Testing for improvement in prediction model performance. *Stat. Med.* **32**, 1467–1482 (2013).
297. Rice, K., Higgins, J. P. T. & Lumley, T. A re-evaluation of fixed effect(s) meta-analysis. *J. R. Stat. Soc. Ser. A (Statistics Soc.* **181**, 205–227 (2018).
298. Hedges, L. V. & Vevea, J. L. Fixed- and Random-Effects Models in Meta-Analysis. *Psychol. Methods* **3**, 486–504 (1998).
299. Viechtbauer, W. Conducting meta-analyses in R with the metafor. *J. Stat. Softw.* **36**, 1–48 (2010).
300. Borenstein, M., Hedges, L. V., Higgins, J. P. T. & Rothstein, H. R. A basic introduction to fixed-effect and random-effects models for meta-analysis. *Res. Synth. Methods* **1**, 97–111 (2010).
301. Kontopantelis, E. & Reeves, D. Performance of statistical methods for meta-analysis when true study effects are non-normally distributed: A simulation study. *Stat. Methods Med. Res.* **21**, 409–426 (2012).
302. Langan, D. *et al.* A comparison of heterogeneity variance estimators in simulated random-effects meta-analyses. *Res. Synth. Methods* **10**, 83–98 (2019).

16. Acknowledgments

Additional support for EADB cohorts

The work for this manuscript was further supported by the CoSTREAM project (www.costream.eu) and funding from the European Union's Horizon 2020 research and innovation programme under grant agreement No 667375. This work is also funded by la fondation pour la recherché médicale (FRM) (EQU202003010147) Italian Ministry of Health (Ricerca Corrente); Ministero dell'Istruzione, del l'Università e della Ricerca–MIUR project "Dipartimenti di Eccellenza 2018–2022" to Department of Neuroscience "Rita Levi Montalcini", University of Torino (IR), and AIRAlzh Onlus-ANCC-COOP (SB); Partly supported by "Ministero della Salute", I.R.C.C.S. Research Program, Ricerca Corrente 2018-2020, Linea n. 2 "Meccanismi genetici, predizione e terapie innovative delle malattie complesse" and by the "5 x 1000" voluntary contribution to the Fondazione I.R.C.C.S.

Ospedale “Casa Sollievo della Sofferenza”; and RF-2018-12366665, Fondi per la ricerca 2019 (Sandro Sorbi). Copenhagen General Population Study (CGPS): We thank staff and participants of the CGPS for their important contributions. Karolinska Institutet AD cohort: Dr. C.G. and co-authors of the Karolinska Institutet AD cohort report grants from Swedish Research Council (VR) 2015-02926, 2018-02754, 2015-06799, Swedish Alzheimer Foundation, Stockholm County Council ALF and resarch school, Karolinska Institutet StratNeuro, Swedish Demensfonden, and Swedish brain foundation, during the conduct of the study. ADGEN: This work was supported by Academy of Finland (grant numbers 307866); Sigrid Jusélius Foundation; the Strategic Neuroscience Funding of the University of Eastern Finland; EADB project in the JPNDCO-FUND program (grant number 301220). CBAS: Supported by the project no. LQ1605 from the National Program of Sustainability II (MEYS CR), Supported by Ministry of Health of the Czech Republic, grant nr. NV19-04-00270 (All rights reserved), Grant Agency of Charles University Grants No. 693018 and 654217; the Ministry of Health, Czech Republic—conceptual development of research organization, University Hospital Motol, Prague, Czech Republic Grant No. 00064203; the Czech Ministry of Health Project AZV Grant No. 16—27611A; and Institutional Support of Excellence 2. LF UK Grant No. 699012. CNRMAJ-Rouen: This study received fundings from the Centre National de Référence Malades Alzheimer Jeunes (CNRMAJ). The Finnish Geriatric Intervention Study for the Prevention of Cognitive Impairment and Disability (FINGER) data collection was supported by grants from the Academy of Finland, La Carita Foundation, Juho Vainio Foundation, Novo Nordisk Foundation, Finnish Social Insurance Institution, Ministry of Education and Culture Research Grants, Yrjö Jahnsson Foundation, Finnish Cultural Foundation South Osthrobothnia Regional Fund, and EVO/State Research Funding grants of University Hospitals of Kuopio, Oulu and Turku, Seinäjoki Central Hospital and Oulu City Hospital, Alzheimer's Research & Prevention Foundation USA, AXA Research Fund, Knut and Alice Wallenberg Foundation Sweden, Center for Innovative Medicine (CIMED) at Karolinska Institutet Sweden, and Stiftelsen Stockholms sjukhem Sweden. FINGER cohort genotyping was funded by EADB project in the JPND CO-FUND (grant number 301220). Research at the Belgian EADB site is funded in part by the Alzheimer Research Foundation (SAO-FRA), The Research Foundation Flanders (FWO), and the University of Antwerp Research Fund. FK is supported by a BOF DOCPRO fellowship of the University of Antwerp Research Fund. SNAC-K is financially supported by the Swedish Ministry of Health and Social Affairs, the participating County Councils and Municipalities, and the Swedish Research Council. BDR Bristol: We would like to thank the South West Dementia Brain Bank (SWDBB) for providing brain tissue for this study. The SWDBB is part of the Brains for Dementia Research programme, jointly funded by Alzheimer’s Research UK and Alzheimer’s Society and is supported by BRACE (Bristol Research into Alzheimer’s and Care of the Elderly) and the Medical Research Council. BDR Manchester: We would like to thank the Manchester Brain Bankfor providing brain tissue for this study. The Manchester Brain Bank is part of the Brains for Dementia Research programme, jointly funded by Alzheimer’s Research UK and Alzheimer’s Society. BDR KCL: Human post-mortem tissue was provided by the London Neurodegenerative Diseases Brain Bank which receives funding from the UK Medical Research Council and as part of the Brains for Dementia Research programme, jointly funded by Alzheimer’s Research UK and the Alzheimer’s Society. The CFAS Wales study was funded by the ESRC (RES-060-25-0060) and HEFCW as ‘Maintaining function and well-being in later life: a longitudinal cohort study’. We are grateful to the NISCHR Clinical Research Centre for their assistance in tracing participants and in interviewing and in collecting blood samples, and to general practices in the study areas for their cooperation. MRC: We thank all individuals who participated in this study. Cardiff University was supported by the Alzheimer's Society (AS; grant RF014/164) and the Medical Research Council (MRC; grants G0801418/1, MR/K013041/1, MR/L023784/1) (R.S. is an AS Research Fellow). Cardiff University was also supported by the European Joint Programme for Neurodegenerative Disease (JPND; grant MR/L501517/1), Alzheimer's Research UK (ARUK; grant ARUK-PG2014-1), the Welsh Assembly Government (grant SGR544:CADR), Brain’s for dementia Research and a donation from the Moondance

Charitable Foundation. Cardiff University acknowledges the support of the UK Dementia Research Institute, of which J.W. is an associate director. Cambridge University acknowledges support from the MRC. Patient recruitment for the MRC Prion Unit/UCL Department of Neurodegenerative Disease collection was supported by the UCLH/UCL Biomedical Centre and NIHR Queen Square Dementia Biomedical Research Unit. The University of Southampton acknowledges support from the AS. King's College London was supported by the NIHR Biomedical Research Centre for Mental Health and the Biomedical Research Unit for Dementia at the South London and Maudsley NHS Foundation Trust and by King's College London and the MRC. ARUK and the Big Lottery Fund provided support to Nottingham University. A.Ram. : Part of the work was funded by the JPND EADB grant (German Federal Ministry of Education and Research (BMBF) grant: 01ED1619A). A. Ram. is also supported by the German Research Foundation (DFG) grants Nr: RA 1971/6-1, RA1971/7-1, and RA 1971/8-1. German Study on Ageing, Cognition and Dementia in Primary Care Patients (AgeCoDe): This study/publication is part of the German Research Network on Dementia (KND), the German Research Network on Degenerative Dementia (KNDD; German Study on Ageing, Cognition and Dementia in Primary Care Patients; AgeCoDe), and the Health Service Research Initiative (Study on Needs, health service use, costs and health-related quality of life in a large sample of oldestold primary care patients (85+; AgeQualiDe)) and was funded by the German Federal Ministry of Education and Research (grants KND: 01GI0102, 01GI0420, 01GI0422, 01GI0423, 01GI0429, 01GI0431, 01GI0433, 01GI0434; grants KNDD: 01GI0710, 01GI0711, 01GI0712, 01GI0713, 01GI0714, 01GI0715, 01GI0716; grants Health Service Research Initiative: 01GY1322A, 01GY1322B, 01GY1322C, 01GY1322D, 01GY1322E, 01GY1322F, 01GY1322G). VITA study: The support of the Ludwig Boltzmann Society and the AFI Germany have supported the VITA study. The former VITA study group should be acknowledged: W. Danielczyk, G. Gatterer, K Jellinger, S Jugwirth, KH Tragl, S Zehetmayer. Vogel Study: This work was financed by a research grant of the ‘‘Vogelstiftung Dr. Eckernkamp’’. HELIAD study: This study was supported by the grants: IIRG-09-133014 from the Alzheimer’s Association, 189 10276/8/9/2011 from the ESPA-EU program Excellence Grant (ARISTEIA) and the ΔΥ2β/οικ.51657/14.4.2009 of the Ministry for Health and Social Solidarity (Greece). Biobank Department of Psychiatry, UMG: Prof. Jens Wiltfang is supported by an Ilídio Pinho professorship and iBiMED (UID/BIM/04501/2013), and FCT project PTDC/DTP_PIC/5587/2014 at the University of Aveiro, Portugal. Lausanne study: This work was supported by grants from the Swiss National Research Foundation (SNF 320030_141179). PAGES study: Harald Hampel is an employee of Eisai Inc. During part of this work he was supported by the AXA Research Fund, the “Fondation partenariale Sorbonne Université” and the “Fondation pour la Recherche sur Alzheimer”, Paris, France. Mannheim, Germany Biobank: Department of geriatric Psychiatry, Central Institute for Mental Health, Mannheim, University of Heidelberg, Germany. Genotyping for the Swedish Twin Studies of Aging was supported by NIH/NIA grant R01 AG037985. Genotyping in TwinGene was supported by NIH/NIDDK U01 DK066134. WvdF is recipient of Joint Programming for Neurodegenerative Diseases (JPND) grants PERADES (ANR-13-JPRF-0001) and EADB (733051061). Gothenburg Birth Cohort (GBC) Studies: We would like to thank UCL Genomics for performing the genotyping analyses. The studies were supported by The Stena Foundation, The Swedish Research Council (2015-02830, 2013-8717), The Swedish Research Council for Health, Working Life and Wellfare (2013-1202, 2005-0762, 2008-1210, 2013-2300, 2013- 2496, 2013-0475), The Brain Foundation, Sahlgrenska University Hospital (ALF), The Alzheimer’s Association (IIRG-03-6168), The Alzheimer’s Association Zenith Award (ZEN-01-3151), Eivind och Elsa K:son Sylvans Stiftelse, The Swedish Alzheimer Foundation. Clinical AD, Sweden: We would like to thank UCL Genomics for performing the genotyping analyses. Barcelona Brain Biobank: Brain Donors of the Neurological Tissue Bank of the Biobanc-Hospital Clinic-IDIBAPS and their families for their generosity. Hospital Clínic de Barcelona Spanish Ministry of Economy and Competitiveness-Instituto de Salud Carlos III and Fondo Europeo de Desarrollo Regional (FEDER), Unión Europea, “Una manera de hacer Europa” grants (PI16/0235 to Dr. R. Sánchez-Valle and

PI17/00670 to Dr. A.Antonelli). AA is funded by Departament de Salut de la Generalitat de Catalunya, PERIS 2016-2020 (SLT002/16/00329). Work at JP-T laboratory was possible thanks to funding from Ciberned and generous gifts from Consuelo Cervera Yuste and Juan Manuel Moreno Cervera. Sydney Memory and Ageing Study (Sydney MAS): We gratefully acknowledge and thank the following for their contributions to Sydney MAS: participants, their supporters and the Sydney MAS Research Team (current and former staff and students). Funding was awarded from the Australian National Health and Medical Research Council (NHMRC) Program Grants (350833, 568969, 109308). This work was supported by InnoMed (Innovative Medicines in Europe), an integrated project funded by the European Union of the Sixth Framework program priority (FP6-2004- LIFESCIHEALTH-5). Oviedo: This work was partly supported by Grant from Fondo de Investigaciones Sanitarias-Fondos FEDER EuropeanUnion to V.A. PI15/00878. Project MinE: The ProjectMinE study was supported by the ALS Foundation Netherlands and the MND association (UK) (Project MinE, www.projectmine.com). The SPIN cohort: We are indebted to patients and their families for their participation in the “Sant Pau Initiative on Neurodegeneration cohort”, at the Sant Pau Hospital (Barcelona). This is a multimodal research cohort for biomarker discovery and validation that is partially funded by Generalitat de Catalunya (2017 SGR 547 to JC), as well as from the Institute of Health Carlos III-Subdirección General de Evaluación and the Fondo Europeo de Desarrollo Regional (FEDER- “Una manera de Hacer Europa”) (grants PI11/02526, PI14/01126, and PI17/01019 to JF; PI17/01895 to AL), and the Centro de Investigación Biomédica en Red Enfermedades Neurodegenerativas programme (Program 1, Alzheimer Disease to AL). We would also like to thank the Fundació Bancària Obra Social La Caixa (DABNI project) to JF and AL; and Fundación BBVA (to AL), for their support in funding this follow-up study. Adolfo López de Munain is supported by Fundación Salud 2000 (PI2013156), CIBERNED and Diputación Foral de Gipuzkoa (Exp.114/17). P.S.J. is supported by CIBERNED and Carlos III Institute of Health, Spain (PI08/0139, PI12/02288, and PI16/01652, PI20/01011), jointly funded by Fondo Europeo de Desarrollo Regional (FEDER), Unión Europea, “Una manera de hacer Europa”. We thank Biobanco Valdecilla for their support. Amsterdam dementia Cohort (ADC): Research of the Alzheimer center Amsterdam is part of the neurodegeneration research program of Amsterdam Neuroscience. The AlzheimerCenter Amsterdam is supported by Stichting Alzheimer Nederland and Stichting VUmc fonds. The clinical database structure was developed with funding from Stichting Dioraphte. Genotyping of the Dutch case-control samples was performed in the context of EADB (European Alzheimer&Dementia biobank) funded by the JPco-fuND FP-829-029 (ZonMW project number #733051061). This research is performed by using data from the Parelsnoer Institute an initiative of the Dutch Federation of University Medical Centres (www.parelsnoer.org). 100-Plus study: We are grateful for the collaborative efforts of all participating centenarians and their family members and/or relations. We thank the Netherlands Brain Bank for supplying DNA for genotyping. This work was supported by Stichting AlzheimerNederland (WE09.2014-03), Stichting Diorapthe, Horstingstuit foundation, Memorabel (ZonMW project number #733050814, #733050512) and Stichting VUmcFonds. Additional support for EADB cohorts: WF, SL, HH are recipients of ABOARD, a public-private partnership receiving funding from ZonMW (#73305095007) and Health~Holland, Topsector Life Sciences & Health (PPP-allowance; #LSHM20106). The DELCODE study was funded by the German Center for Neurodegenerative Diseases (Deutsches Zentrum für Neurodegenerative Erkrankungen (DZNE)), reference number BN012.

Gra@ce. The Genome Research @ Fundació ACE project (GR@ACE) is supported by Grifols SA, Fundación bancaria ‘La Caixa’, Fundació ACE, and CIBERNED (Centro de Investigación Biomédica en Red Enfermedades Neurodegenerativas (Program 1, Alzheimer Disease to MB and AR)). A.R. and M.B. receive support from the European Union/EFPIA Innovative Medicines Initiative Joint undertaking ADAPTED and MOPEAD projects (grant numbers 115975 and 115985, respectively). M.B. and A.R. are also supported by national grants PI13/02434, PI16/01861, PI17/01474 and PI19/01240. Acción Estratégica en Salud is

integrated into the Spanish National R + D + I Plan and funded by ISCIII (Instituto de Salud Carlos III)–Subdirección General de Evaluación and the Fondo Europeo de Desarrollo Regional (FEDER–'Una manera de hacer Europa'). Some control samples and data from patients included in this study were provided in part by the National DNA Bank Carlos III (www.bancoadn.org, University of Salamanca, Spain) and Hospital Universitario Virgen de Valme (Sevilla, Spain); they were processed following standard operating procedures with the appropriate approval of the Ethical and Scientific Committee. The present work has been performed as part of the doctoral program of I. de Rojas at the Universitat de Barcelona (Barcelona, Spain).

EADI. This work has been developed and supported by the LABEX (laboratory of excellence program investment for the future) DISTALZ grant (Development of Innovative Strategies for a Transdisciplinary approach to ALZheimer's disease) including funding from MEL (Metropole européenne de Lille), ERDF (European Regional Development Fund) and Conseil Régional Nord Pas de Calais. This work was supported by INSERM, the National Foundation for Alzheimer's disease and related disorders, the Institut Pasteur de Lille and the Centre National de Recherche en Génomique Humaine, CEA, the JPND PERADES, the Laboratory of Excellence GENMED (Medical Genomics) grant no. ANR-10-LABX-0013 managed by the National Research Agency (ANR) part of the Investment for the Future program, and the FP7 AgedBrainSysBio. The Three-City Study was performed as part of collaboration between the Institut National de la Santé et de la Recherche Médicale (Inserm), the Victor Segalen Bordeaux II University and Sanofi-Synthélabo. The Fondation pour la Recherche Médicale funded the preparation and initiation of the study. The 3C Study was also funded by the Caisse Nationale Maladie des Travailleurs Salariés, Direction Générale de la Santé, MGEN, Institut de la Longévité, Agence Française de Sécurité Sanitaire des Produits de Santé, the Aquitaine and Bourgogne Regional Councils, Agence Nationale de la Recherche, ANR supported the COGINUT and COVADIS projects. Fondation de France and the joint French Ministry of Research/INSERM "Cohortes et collections de données biologiques" programme. Lille Génopôle received an unconditional grant from Eisai. The Three-city biological bank was developed and maintained by the laboratory for genomic analysis LAG-BRC - Institut Pasteur de Lille.

GERAD/PERADES. We thank all individuals who participated in this study. Cardiff University was supported by the Wellcome Trust, Alzheimer's Society (AS; grant RF014/164), the Medical Research Council (MRC; grants G0801418/1, MR/K013041/1, MR/L023784/1), the European Joint Programme for Neurodegenerative Disease (JPND, grant MR/L501517/1), Alzheimer's Research UK (ARUK, grant ARUK-PG2014-1), Welsh Assembly Government (grant SGR544:CADR), a donation from the Moondance Charitable Foundation, UK Dementia's Platform (DPUK, reference MR/L023784/1), and the UK Dementia Research Institute at Cardiff. Cambridge University acknowledges support from the MRC. ARUK supported sample collections at the Kings College London, the South West Dementia Bank, Universities of Cambridge, Nottingham, Manchester and Belfast. King's College London was supported by the NIHR Biomedical Research Centre for Mental Health and Biomedical Research Unit for Dementia at the South London and Maudsley NHS Foundation Trust and Kings College London and the MRC. Alzheimer's Research UK (ARUK) and the Big Lottery Fund provided support to Nottingham University. Ulster Garden Villages, AS, ARUK, American Federation for Aging Research, NI R&D Office and the Royal College of Physicians/Dunhill Medical Trust provided support for Queen's University, Belfast. The University of Southampton acknowledges support from the AS. The MRC and Mercer's Institute for Research on Ageing supported the Trinity College group. DCR is a Wellcome Trust Principal Research fellow. The South West Dementia Brain Bank acknowledges support from Bristol Research into Alzheimer's and Care of the Elderly. The Charles Wolfson Charitable Trust supported the OPTIMA group. Washington University was funded by NIH grants, Barnes Jewish Foundation and the Charles and Joanne Knight Alzheimer's Research Initiative. Patient recruitment for the MRC Prion Unit/UCL Department of Neurodegenerative Disease collection was supported by

the UCLH/UCL Biomedical Research Centre and their work was supported by the NIHR Queen Square Dementia BRU, the Alzheimer's Research UK and the Alzheimer's Society. LASER-AD was funded by Lundbeck SA. The AgeCoDe study group was supported by the German Federal Ministry for Education and Research grants 01 GI 0710, 01 GI 0712, 01 GI 0713, 01 GI 0714, 01 GI 0715, 01 GI 0716, 01 GI 0717. Genotyping of the Bonn case-control sample was funded by the German centre for Neurodegenerative Diseases (DZNE), Germany. The GERAD Consortium also used samples ascertained by the NIMH AD Genetics Initiative. HH was supported by a grant of the Katharina-Hardt-Foundation, Bad Homburg vor der Höhe, Germany. The KORA F4 studies were financed by Helmholtz Zentrum München; German Research Center for Environmental Health; BMBF; German National Genome Research Network and the Munich Center of Health Sciences. The Heinz Nixdorf Recall cohort was funded by the Heinz Nixdorf Foundation and BMBF. We acknowledge use of genotype data from the 1958 Birth Cohort collection and National Blood Service, funded by the MRC and the Wellcome Trust which was genotyped by the Wellcome Trust Case Control Consortium and the Type-1 Diabetes Genetics Consortium, sponsored by the National Institute of Diabetes and Digestive and Kidney Diseases, National Institute of Allergy and Infectious Diseases, National Human Genome Research Institute, National Institute of Child Health and Human Development and Juvenile Diabetes Research Foundation International. The project is also supported through the following funding organisations under the aegis of JPND - www.jpnd.eu (United Kingdom, Medical Research Council (MR/L501529/1; MR/R024804/1) and Economic and Social Research Council (ES/L008238/1)) and through the Motor Neurone Disease Association. This study represents independent research part funded by the National Institute for Health Research (NIHR) Biomedical Research Centre at South London and Maudsley NHS Foundation Trust and King's College London. Prof Jens Wiltfang is supported by an Ilídio Pinho professorship and iBiMED (UID/BIM/04501/2013), at the University of Aveiro, Portugal.

Rotterdam study. Rotterdam (RS). This study was funded by the Netherlands Organisation for Health Research and Development (ZonMW) as part of the Joint Programming for Neurological Disease (JPND) as part of the PERADES Program (Defining Genetic Polygenic, and Environmental Risk for Alzheimer's disease using multiple powerful cohorts, focused Epigenetics and Stem cell metabolomics), Project number 733051021. This work was funded also by the European Union Innovative Medicine Initiative (IMI) programme under grant agreement No. 115975 as part of the Alzheimer's Disease Apolipoprotein Pathology for Treatment Elucidation and Development (ADAPTED, https://www.imi-adapted.eu) and the European Union's Horizon 2020 research and innovation programme as part of the Common mechanisms and pathways in Stroke and Alzheimer's disease CoSTREAM project (www.costream.eu, grant agreement No. 667375). The current study is supported by the Deltaplan Dementie and Memorabel supported by ZonMW (Project number 733050814) and Alzheimer Nederland. The Rotterdam Study is funded by Erasmus Medical Center and Erasmus University, Rotterdam, Netherlands Organization for the Health Research and Development (ZonMw), the Research Institute for Diseases in the Elderly (RIDE), the Ministry of Education, Culture and Science, the Ministry for Health, Welfare and Sports, the European Commission (DG XII), and the Municipality of Rotterdam. The authors are grateful to the study participants, the staff from the Rotterdam Study and the participating general practitioners and pharmacists. The generation and management of GWAS genotype data for the Rotterdam Study (RS-I, RS-II, RS-III) was executed by the Human Genotyping Facility of the Genetic Laboratory of the Department of Internal Medicine, Erasmus MC, Rotterdam, The Netherlands. The GWAS datasets are supported by the Netherlands Organization of Scientific Research NWO Investments (Project number 175.010.2005.011, 911-03-012), the Genetic Laboratory of the Department of Internal Medicine, Erasmus MC, the Research Institute for Diseases in the Elderly (014-93-015; RIDE2), the Netherlands Genomics Initiative (NGI)/Netherlands Organization for Scientific Research (NWO) Netherlands Consortium for Healthy Aging (NCHA), project number 050-060-810. We thank Pascal Arp, Mila Jhamai, Marijn Verkerk, Lizbeth Herrera and Marjolein Peters, MSc, and Carolina Medina-Gomez,

MSc, for their help in creating the GWAS database, and Karol Estrada, PhD, Yurii Aulchenko, PhD, and Carolina Medina- Gomez, MSc, for the creation and analysis of imputed data.

DemGene. The project has received funding from The Research Council of Norway (RCN) Grant Nos. 213837, 223273, 225989, 248778, and 251134 and EU JPND Program RCN Grant Nos. 237250, 311993, the South-East Norway Health Authority Grant No. 2013-123, the Norwegian Health Association, and KG Jebsen Foundation. The RCN FRIPRO Mobility grant scheme (FRICON) is co-funded by the European Union's Seventh Framework Programme for research, technological development and demonstration under Marie Curie grant agreement No 608695. European Community's grant PIAPP-GA-2011-286213 PsychDPC..

Bonn study. This group would like to thank Dr. Heike Koelsch for her scientific support. The Bonn group was funded by the German Federal Ministry of Education and Research (BMBF): Competence Network Dementia (CND) grant number 01GI0102, 01GI0711, 01GI042

ADGC. The National Institutes of Health, National Institute on Aging (NIH-NIA) supported this work through the following grants: ADGC, U01 AG032984, RC2 AG036528; Samples from the National Cell Repository for Alzheimer's Disease (NCRAD), which receives government support under a cooperative agreement grant (U24 AG21886) awarded by the National Institute on Aging (NIA), were used in this study. We thank contributors who collected samples used in this study, as well as patients and their families, whose help and participation made this work possible; Data for this study were prepared, archived, and distributed by the National Institute on Aging Alzheimer's Disease Data Storage Site (NIAGADS) at the University of Pennsylvania (U24-AG041689-01); NACC, U01 AG016976; NIA LOAD (Columbia University), U24 AG026395, U24 AG026390, R01AG041797; Banner Sun Health Research Institute P30 AG019610; Boston University, P30 AG013846, U01 AG10483, R01 CA129769, R01 MH080295, R01 AG017173, R01 AG025259, R01 AG048927, R01AG33193, R01 AG009029; Columbia University, P50 AG008702, R37 AG015473, R01 AG037212, R01 AG028786; Duke University, P30 AG028377, AG05128; Einstein Aging Study NIA grant at Albert Einstein College of Medicine, P01 AG03949. Emory University, AG025688; Group Health Research Institute, UO1 AG006781, UO1 HG004610, UO1 HG006375, U01 HG008657; Indiana University, P30 AG10133, R01 AG009956, RC2 AG036650; Johns Hopkins University, P50 AG005146, R01 AG020688; Massachusetts General Hospital, P50 AG005134; Mayo Clinic, P50 AG016574, R01 AG032990, KL2 RR024151; Mount Sinai School of Medicine, P50 AG005138, P01 AG002219; New York University, P30 AG08051, UL1 RR029893, 5R01AG012101, 5R01AG022374, 5R01AG013616, 1RC2AG036502, 1R01AG035137; North Carolina A&T University, P20 MD000546, R01 AG28786-01A1; Northwestern University, P30 AG013854; Oregon Health & Science University, P30 AG008017, R01 AG026916; Rush University, P30 AG010161, R01 AG019085, R01 AG15819, R01 AG17917, R01 AG030146, R01 AG01101, RC2 AG036650, R01 AG22018; TGen, R01 NS059873; University of Alabama at Birmingham, P50 AG016582; University of Arizona, R01 AG031581; University of California, Davis, P30 AG010129; University of California, Irvine, P50 AG016573; University of California, Los Angeles, P50 AG016570; University of California, San Diego, P50 AG005131; University of California, San Francisco, P50 AG023501, P01 AG019724; University of Kentucky, P30 AG028383, AG05144; University of Michigan, P30 AG053760 and AG063760; University of Pennsylvania, P30 AG010124; University of Pittsburgh, P50 AG005133, AG030653, AG041718, AG07562, AG02365; University of Southern California, P50 AG005142; University of Texas Southwestern, P30 AG012300; University of Miami, R01 AG027944, AG010491, AG027944, AG021547, AG019757; University of Washington, P50 AG005136, R01 AG042437; University of Wisconsin, P50 AG033514; Vanderbilt University, R01 AG019085; and Washington University, P50 AG005681, P01 AG03991, P01 AG026276. HP was supported by AG025711. ER was supported by CCNA. The Kathleen Price Bryan Brain Bank at Duke University Medical Center is funded by NINDS grant # NS39764, NIMH MH60451 and by Glaxo Smith Kline. Support was also from the Alzheimer's Association

(LAF, IIRG-08-89720; MP-V, IIRG-05- 14147), the US Department of Veterans Affairs Administration, Office of Research and Development, Biomedical Laboratory Research Program, and BrightFocus Foundation (MP-V, A2111048). P.S.G.-H. is supported by Wellcome Trust, Howard Hughes Medical Institute, and the Canadian Institute of Health Research. Genotyping of the TGEN2 cohort was supported by Kronos Science. The TGen series was also funded by NIA grant AG041232 to AJM and MJH, The Banner Alzheimer's Foundation, The Johnnie B. Byrd Sr. Alzheimer's Institute, the Medical Research Council, and the state of Arizona and also includes samples from the following sites: Newcastle Brain Tissue Resource (funding via the Medical Research Council, local NHS trusts and Newcastle University), MRC London Brain Bank for Neurodegenerative Diseases (funding via the Medical Research Council), South West Dementia Brain Bank (funding via numerous sources including the Higher Education Funding Council for England (HEFCE), Alzheimer's Research Trust (ART), BRACE as well as North Bristol NHS Trust Research and Innovation department and DeNDRoN), The Netherlands Brain Bank (funding via numerous sources including Stichting MS Research, Brain Net Europe, Hersenstichting Nederland Breinbrekend Werk, International Parkinson Fonds, Internationale Stiching Alzheimer Onderzoek), Institut de Neuropatologia, Servei Anatomia Patologica, Universitat de Barcelona. ADNI data collection and sharing was funded by the National Institutes of Health Grant U01 AG024904 and Department of Defense award number W81XWH-12-2-0012. ADNI is funded by the National Institute on Aging, the National Institute of Biomedical Imaging and Bioengineering, and through generous contributions from the following: AbbVie, Alzheimer's Association; Alzheimer's Drug Discovery Foundation; Araclon Biotech; BioClinica, Inc.; Biogen; Bristol-Myers Squibb Company; CereSpir, Inc.; Eisai Inc.; Elan Pharmaceuticals, Inc.; Eli Lilly and Company; EuroImmun; F. Hoffmann-La Roche Ltd and its affiliated company Genentech, Inc.; Fujirebio; GE Healthcare; IXICO Ltd.; Janssen Alzheimer Immunotherapy Research & Development, LLC.; Johnson & Johnson Pharmaceutical Research & Development LLC.; Lumosity; Lundbeck; Merck & Co., Inc.; Meso Scale Diagnostics, LLC.; NeuroRx Research; Neurotrack Technologies; Novartis Pharmaceuticals Corporation; Pfizer Inc.; Piramal Imaging; Servier; Takeda Pharmaceutical Company; and Transition Therapeutics. The Canadian Institutes of Health Research is providing funds to support ADNI clinical sites in Canada. Private sector contributions are facilitated by the Foundation for the National Institutes of Health (www.fnih.org). The grantee organization is the Northern California Institute for Research and Education, and the study is coordinated by the Alzheimer's Disease Cooperative Study at the University of California, San Diego. ADNI data are disseminated by the Laboratory for Neuro Imaging at the University of Southern California. We thank Drs. D. Stephen Snyder and Marilyn Miller from NIA who are *ex-officio* ADGC members. FTLD-TDP GWAS: National Institute on Aging (AG101024, AG066597 and AG017586)

CHARGE. Cardiovascular Health Study (CHS). This CHS research was supported by NHLBI contracts HHSN268201200036C, HHSN268200800007C, HHSN268201800001C, N01HC55222, N01HC85079, N01HC85080, N01HC85081, N01HC85082, N01HC85083, N01HC85086, 75N92021D00006; and NHLBI grants U01HL080295, U01HL130114, R01HL087652, R01HL105756, R01HL103612, R01HL120393 and 75N92021D00006 with additional contribution from the National Institute of Neurological Disorders and Stroke (NINDS). Additional support was provided through R01AG023629, R01AG033193, R01AG15928, R01AG20098, and U01AG049505 from the National Institute on Aging (NIA). A full list of principal CHS investigators and institutions can be found at CHS-NHLBI.org. The provision of genotyping data was supported in part by the National Center for Advancing Translational Sciences, CTSI grant UL1TR001881, and the National Institute of Diabetes and Digestive and Kidney Disease Diabetes Research Center (DRC) grant DK063491 to the Southern California Diabetes Endocrinology Research Center. Framingham Heart Study. This work was supported by the National Heart, Lung, and Blood Institute's Framingham Heart Study (contracts N01-HC-25195 and HHSN268201500001I). This study was also supported by grants from the National Institute on Aging: R01AG033193, U01AG049505,

U01AG52409, R01AG054076, RF1AG0059421 (S. Seshadri). S. Seshadri and A.L.D. were also supported by additional grants from the National Institute on Aging (R01AG049607, R01AG033040, RF1AG0061872, U01AG058589) and the National Institute of Neurological Disorders and Stroke (R01-NS017950, NS100605). The content is solely the responsibility of the authors and does not necessarily represent the official views of the US National Institutes of Health.

FinnGen. The FinnGen project is funded by two grants from Business Finland (HUS 4685/31/2016 and UH 4386/31/2016) and the following industry partners: AbbVie Inc., AstraZeneca UK Ltd, Biogen MA Inc., Celgene Corporation, Celgene International II Sàrl, Genentech Inc., Merck Sharp & Dohme Corp, Pfizer Inc., GlaxoSmithKline Intellectual Property Development Ltd., Sanofi US Services Inc., Maze Therapeutics Inc., Janssen Biotech Inc, and Novartis AG. Following biobanks are acknowledged for the project samples: Auria Biobank (www.auria.fi/biopankki), THL Biobank (www.thl.fi/biobank), Helsinki Biobank (www.helsinginbiopankki.fi), Biobank Borealis of Northern Finland (https://www.ppshp.fi/Tutkimus-ja-opetus/Biopankki/Pages/Biobank-Borealis-briefly-in-English.aspx), Finnish Clinical Biobank Tampere (www.tays.fi/en-US/Research_and_development/Finnish_Clinical_Biobank_Tampere), Biobank of Eastern Finland (www.ita-suomenbiopankki.fi/en), Central Finland Biobank (www.ksshp.fi/fi-FI/Potilaalle/Biopankki), Finnish Red Cross Blood Service Biobank (www.veripalvelu.fi/verenluovutus/biopankkitoiminta) and Terveystalo Biobank (www.terveystalo.com/fi/Yritystietoa/Terveystalo-Biopankki/Biopankki/). All Finnish Biobanks are members of BBMRI.fi infrastructure (www.bbmri.fi).

QTLs/TWAS analyses. The results published here are in whole or in part based on data obtained from the AD Knowledge Portal (https://adknowledgeportal.synapse.org/). For MayoRNAseq, the study data were provided by the following sources: The Mayo Clinic Alzheimers Disease Genetic Studies, led by Dr. Nilufer Ertekin-Taner and Dr. Steven G. Younkin, Mayo Clinic, Jacksonville, FL using samples from the Mayo Clinic Study of Aging, the Mayo Clinic Alzheimers Disease Research Center, and the Mayo Clinic Brain Bank. Data collection was supported through funding by NIA grants P50 AG016574, R01 AG032990, U01 AG046139, R01 AG018023, U01 AG006576, U01 AG006786, R01 AG025711, R01 AG017216, R01 AG003949, NINDS grant R01 NS080820, CurePSP Foundation, and support from Mayo Foundation. Study data includes samples collected through the Sun Health Research Institute Brain and Body Donation Program of Sun City, Arizona. The Brain and Body Donation Program is supported by the National Institute of Neurological Disorders and Stroke (U24 NS072026 National Brain and Tissue Resource for Parkinsons Disease and Related Disorders), the National Institute on Aging (P30 AG19610 Arizona Alzheimers Disease Core Center), the Arizona Department of Health Services (contract 211002, Arizona Alzheimers Research Center), the Arizona Biomedical Research Commission (contracts 4001, 0011, 05-901 and 1001 to the Arizona Parkinson's Disease Consortium) and the Michael J. Fox Foundation for Parkinsons Research. For ROSMAP, the study data were provided by the Rush Alzheimer’s Disease Center, Rush University Medical Center, Chicago. Data collection was supported through funding by NIA grants P30AG10161 (ROS), R01AG15819 (ROSMAP; genomics and RNAseq), R01AG17917 (MAP), R01AG30146, R01AG36042 (5hC methylation, ATACseq), RC2AG036547 (H3K9Ac), R01AG36836 (RNAseq), R01AG48015 (monocyte RNAseq) RF1AG57473 (single nucleus RNAseq), U01AG32984 (genomic and whole exome sequencing), U01AG46152 (ROSMAP AMP-AD, targeted proteomics), U01AG46161(TMT proteomics), U01AG61356 (whole genome sequencing, targeted proteomics, ROSMAP AMP-AD), the Illinois Department of Public Health (ROSMAP), and the Translational Genomics Research Institute (genomic). Additional phenotypic data can be requested at www.radc.rush.edu. For MSBB, the data were generated from postmortem brain tissue collected through the Mount Sinai VA Medical Center Brain Bank and were provided by Dr. Eric Schadt from Mount Sinai School of Medicine.

This work was supported by grants from the US National Institutes of Health (NIH NIA R21-AG063130, NIA R01-AG054005, NIA R56-AG055824, and NIA U01-AG068880).

18. Supplementary Figures

Supplementary Figure 1. Stage I QQ Plot. QQ Plot of Stage I meta-analysis results (excludes the APOE locus). Genomic inflation factors (λ) were slightly inflated (λ =1.08 overall and 1.17 when restricted to variants with minor allele frequency (MAF) above 1%. However, linkage disequilibrium score (LDSC) regression estimate indicated that the majority of this inflation was due to a polygenic signal, with the intercept being close to 1 (intercept=1.05, se=0.01 versus λ=1.2 on the variants considered in the LDSC analysis).

Supplementary Figure 2. LocusZoom and forest plots for (a) *SORT1*, (b) *CR1* and (c) *ADAM17* loci. The LocusZoom plot is based on the results from Stage I, and the variant in purple is the best associated variant in the Stage I + II meta-analysis. In the forest plots, data are presented as odds-ratio with 95% confidence interval. *P* values are two-sided raw *P* values derived from a fixed-effect meta-analysis. OR: odds ratio, CI: confidence interval, EA: effect allele, EAF: effect allele frequency range across all studies, HetP: heterogeneity *P* value, HetISq: heterogeneity statistic.

Supplementary Figure 3. LocusZoom and forest plots for (a) *PRKD3*, (b) *NCK2* and (c) *BIN1* loci. The LocusZoom plot is based on the results from Stage I, and the variant in purple is the best associated variant in the Stage I + II meta-analysis. In the forest plots, data are presented as odds-ratio with 95% confidence interval. *P* values are two-sided raw *P* values derived from a fixed-effect meta-analysis. OR: odds ratio, CI: confidence interval, EA: effect allele, EAF: effect allele frequency range across all studies, HetP: heterogeneity *P* value, HetISq: heterogeneity statistic.

Supplementary Figure 4. LocusZoom and forest plots for (a) *WDR12*, (b) *INPP5D* and (c) *MME* (1) loci. The LocusZoom plot is based on the results from Stage I, and the variant in purple is the best associated variant in the Stage I + II meta-analysis. In the forest plots, data are presented as odds-ratio with 95% confidence interval. *P* values are two-sided raw *P* values derived from a fixed-effect meta-analysis. OR: odds ratio, CI: confidence interval, EA: effect allele, EAF: effect allele frequency range across all studies, HetP: heterogeneity *P* value, HetISq: heterogeneity statistic.

Supplementary Figure 5. LocusZoom and forest plots for (a) *MME* (2), (b) *IDUA* and (c) *CLNK* loci. The LocusZoom plot is based on the results from Stage I, and the variant in purple is the best associated variant in the Stage I + II meta-analysis. In the forest plots, data are presented as odds-ratio with 95% confidence interval. *P* values are two-sided raw *P* values derived from a fixed-effect meta-analysis. OR: odds ratio, CI: confidence interval, EA: effect allele, EAF: effect allele frequency range across all studies, HetP: heterogeneity *P* value, HetISq: heterogeneity statistic.

Supplementary Figure 6. LocusZoom and forest plots for (a) *RHOH*, (b) *ANKH* and (c) *COX7C* loci. The LocusZoom plot is based on the results from Stage I, and the variant in purple is the best associated variant in the Stage I + II meta-analysis. In the forest plots, data are presented as odds-ratio with 95% confidence interval. *P* values are two-sided raw *P* values derived from a fixed-effect meta-analysis. OR: odds ratio, CI: confidence interval, EA: effect allele, EAF: effect allele frequency range across all studies, HetP: heterogeneity *P* value, HetISq: heterogeneity statistic.

Supplementary Figure 7. LocusZoom and forest plots for (a) *TNIP1*, (b) *RASGEF1C* and (c) *HLA-DQA1* loci. The LocusZoom plot is based on the results from Stage I, and the variant in purple is the best associated variant in the Stage I + II meta-analysis. In the forest plots, data are presented as odds-ratio with 95% confidence interval. *P* values are two-sided raw *P* values derived from a fixed-effect meta-analysis. OR: odds ratio, CI: confidence

interval, EA: effect allele, EAF: effect allele frequency range across all studies, HetP: heterogeneity *P* value, HetISq: heterogeneity statistic.

Supplementary Figure 8. LocusZoom and forest plots for (a) *UNC5CL*, (b) *TREM2* (R62H) and (c) *TREM2* (R47H) loci. The LocusZoom plot is based on the results from Stage I, and the variant in purple is the best associated variant in the Stage I + II meta-analysis. In the forest plots, data are presented as odds-ratio with 95% confidence interval. *P* values are two-sided raw *P* values derived from a fixed-effect meta-analysis. OR: odds ratio, CI: confidence interval, EA: effect allele, EAF: effect allele frequency range across all studies, HetP: heterogeneity *P* value, HetISq: heterogeneity statistic.

Supplementary Figure 9. LocusZoom and forest plots for (a) *TREML2*, (b) *CD2AP* and (c) *HS3ST5* loci. The LocusZoom plot is based on the results from Stage I, and the variant in purple is the best associated variant in the Stage I + II meta-analysis. In the forest plots, data are presented as odds-ratio with 95% confidence interval. *P* values are two-sided raw *P* values derived from a fixed-effect meta-analysis. OR: odds ratio, CI: confidence interval, EA: effect allele, EAF: effect allele frequency range across all studies, HetP: heterogeneity *P* value, HetISq: heterogeneity statistic.

Supplementary Figure 10. LocusZoom and forest plots for (a) *UMAD1*, (b) *ICA1* and (c) *TMEM106B* loci. The LocusZoom plot is based on the results from Stage I, and the variant in purple is the best associated variant in the Stage I + II meta-analysis. In the forest plots, data are presented as odds-ratio with 95% confidence interval. *P* values are two-sided raw *P* values derived from a fixed-effect meta-analysis. OR: odds ratio, CI: confidence interval, EA: effect allele, EAF: effect allele frequency range across all studies, HetP: heterogeneity *P* value, HetISq: heterogeneity statistic.

Supplementary Figure 11. LocusZoom and forest plots for (a) *JAZF1*, (b) *NME8* and (c) *SEC61G* loci. The LocusZoom plot is based on the results from Stage I, and the variant in purple is the best associated variant in the Stage I + II meta-analysis. In the forest plots, data are presented as odds-ratio with 95% confidence interval. *P* values are two-sided raw *P* values derived from a fixed-effect meta-analysis. OR: odds ratio, CI: confidence interval, EA: effect allele, EAF: effect allele frequency range across all studies, HetP: heterogeneity *P* value, HetISq: heterogeneity statistic.

Supplementary Figure 12. LocusZoom and forest plots for (a) *ZCWPW1/NYAP1*, (b) *EPHA1* and (c) *CTSB* loci. The LocusZoom plot is based on the results from Stage I, and the variant in purple is the best associated variant in the Stage I + II meta-analysis. In the forest plots, data are presented as odds-ratio with 95% confidence interval. *P* values are two-sided raw *P* values derived from a fixed-effect meta-analysis. OR: odds ratio, CI: confidence interval, EA: effect allele, EAF: effect allele frequency range across all studies, HetP: heterogeneity *P* value, HetISq: heterogeneity statistic.

Supplementary Figure 13. LocusZoom and forest plots for (a) *PTK2B*, (b) *CLU* and (c) *SHARPIN* loci. The LocusZoom plot is based on the results from Stage I, and the variant in purple is the best associated variant in the Stage I + II meta-analysis. In the forest plots, data are presented as odds-ratio with 95% confidence interval. *P* values are two-sided raw *P* values derived from a fixed-effect meta-analysis. OR: odds ratio, CI: confidence interval, EA: effect allele, EAF: effect allele frequency range across all studies, HetP: heterogeneity *P* value, HetISq: heterogeneity statistic.

Supplementary Figure 14. LocusZoom and forest plots for (a) *ABCA1*, (b) *USP6NL* and (c) *ANK3* loci. The LocusZoom plot is based on the results from Stage I, and the variant in purple is the best associated variant in the Stage I + II meta-analysis. In the forest plots, data are presented as odds-ratio with 95% confidence interval. *P* values are two-sided raw *P*

values derived from a fixed-effect meta-analysis. OR: odds ratio, CI: confidence interval, EA: effect allele, EAF: effect allele frequency range across all studies, HetP: heterogeneity *P* value, HetISq: heterogeneity statistic.

Supplementary Figure 15. LocusZoom and forest plots for (a) *TSPAN14*, (b) *BLNK* and (c) *PLEKHA1* loci. The LocusZoom plot is based on the results from Stage I, and the variant in purple is the best associated variant in the Stage I + II meta-analysis. In the forest plots, data are presented as odds-ratio with 95% confidence interval. *P* values are two-sided raw *P* values derived from a fixed-effect meta-analysis. OR: odds ratio, CI: confidence interval, EA: effect allele, EAF: effect allele frequency range across all studies, HetP: heterogeneity *P* value, HetISq: heterogeneity statistic.

Supplementary Figure 16. LocusZoom and forest plots for (a) *CELF1*/*SPI1*, (b) *MS4A* and (c) *PICALM* loci. The LocusZoom plot is based on the results from Stage I, and the variant in purple is the best associated variant in the Stage I + II meta-analysis. In the forest plots, data are presented as odds-ratio with 95% confidence interval. *P* values are two-sided raw *P* values derived from a fixed-effect meta-analysis. OR: odds ratio, CI: confidence interval, EA: effect allele, EAF: effect allele frequency range across all studies, HetP: heterogeneity *P* value, HetISq: heterogeneity statistic.

Supplementary Figure 17. LocusZoom and forest plots for (a) *SORL1* (1), (b) *SORL1* (2) and (c) *TPCN1* loci. The LocusZoom plot is based on the results from Stage I, and the variant in purple is the best associated variant in the Stage I + II meta-analysis. In the forest plots, data are presented as odds-ratio with 95% confidence interval. *P* values are two-sided raw *P* values derived from a fixed-effect meta-analysis. OR: odds ratio, CI: confidence interval, EA: effect allele, EAF: effect allele frequency range across all studies, HetP: heterogeneity *P* value, HetISq: heterogeneity statistic.

Supplementary Figure 18. LocusZoom and forest plots for (a) *FERMT2*, (b) *SLC24A4*/*RIN3* (1) and (c) *SLC24A4*/*RIN3* (2) loci. The LocusZoom plot is based on the results from Stage I, and the variant in purple is the best associated variant in the Stage I + II meta-analysis. In the forest plots, data are presented as odds-ratio with 95% confidence interval. *P* values are two-sided raw *P* values derived from a fixed-effect meta-analysis. OR: odds ratio, CI: confidence interval, EA: effect allele, EAF: effect allele frequency range across all studies, HetP: heterogeneity *P* value, HetISq: heterogeneity statistic.

Supplementary Figure 19. LocusZoom and forest plots for (a) *IGH* gene cluster (1), (b) *IGH* gene cluster (2) and (c) *SPPL2A* loci. The LocusZoom plot is based on the results from Stage I, and the variant in purple is the best associated variant in the Stage I + II meta-analysis. In the forest plots, data are presented as odds-ratio with 95% confidence interval. *P* values are two-sided raw *P* values derived from a fixed-effect meta-analysis. OR: odds ratio, CI: confidence interval, EA: effect allele, EAF: effect allele frequency range across all studies, HetP: heterogeneity *P* value, HetISq: heterogeneity statistic.

Supplementary Figure 20. LocusZoom and forest plots for (a) *ADAM10*, (b) *APH1B* and (c) *SNX1* loci. The LocusZoom plot is based on the results from Stage I, and the variant in purple is the best associated variant in the Stage I + II meta-analysis. In the forest plots, data are presented as odds-ratio with 95% confidence interval. *P* values are two-sided raw *P* values derived from a fixed-effect meta-analysis. OR: odds ratio, CI: confidence interval, EA: effect allele, EAF: effect allele frequency range across all studies, HetP: heterogeneity *P* value, HetISq: heterogeneity statistic.

Supplementary Figure 21. LocusZoom and forest plots for (a) *CTSH*, (b) *DOC2A* and (c) *KAT8* loci. The LocusZoom plot is based on the results from Stage I, and the variant in purple is the best associated variant in the Stage I + II meta-analysis. In the forest plots, data

are presented as odds-ratio with 95% confidence interval. *P* values are two-sided raw *P* values derived from a fixed-effect meta-analysis. OR: odds ratio, CI: confidence interval, EA: effect allele, EAF: effect allele frequency range across all studies, HetP: heterogeneity *P* value, HetISq: heterogeneity statistic.

Supplementary Figure 22. LocusZoom and forest plots for (a) *IL34*, (b) *MAF* and (c) *PLCg2* (1) loci. The LocusZoom plot is based on the results from Stage I, and the variant in purple is the best associated variant in the Stage I + II meta-analysis. In the forest plots, data are presented as odds-ratio with 95% confidence interval. *P* values are two-sided raw *P* values derived from a fixed-effect meta-analysis. OR: odds ratio, CI: confidence interval, EA: effect allele, EAF: effect allele frequency range across all studies, HetP: heterogeneity *P* value, HetISq: heterogeneity statistic.

Supplementary Figure 23. LocusZoom and forest plots for (a) *PLCg2* (2), (b) *FOXF1* and (c) *PRDM7* loci. The LocusZoom plot is based on the results from Stage I, and the variant in purple is the best associated variant in the Stage I + II meta-analysis. In the forest plots, data are presented as odds-ratio with 95% confidence interval. *P* values are two-sided raw *P* values derived from a fixed-effect meta-analysis. OR: odds ratio, CI: confidence interval, EA: effect allele, EAF: effect allele frequency range across all studies, HetP: heterogeneity *P* value, HetISq: heterogeneity statistic.

Supplementary Figure 24. LocusZoom and forest plots for (a) *WDR81*, (b) *SCIMP/RABEP1* and (c) *MYO15A* loci. The LocusZoom plot is based on the results from Stage I, and the variant in purple is the best associated variant in the Stage I + II meta-analysis. In the forest plots, data are presented as odds-ratio with 95% confidence interval. *P* values are two-sided raw *P* values derived from a fixed-effect meta-analysis. OR: odds ratio, CI: confidence interval, EA: effect allele, EAF: effect allele frequency range across all studies, HetP: heterogeneity *P* value, HetISq: heterogeneity statistic.

Supplementary Figure 25. LocusZoom and forest plots for (a) *GRN*, (b) *MAPT* and (c) *ABI3* loci. The LocusZoom plot is based on the results from Stage I, and the variant in purple is the best associated variant in the Stage I + II meta-analysis. In the forest plots, data are presented as odds-ratio with 95% confidence interval. *P* values are two-sided raw *P* values derived from a fixed-effect meta-analysis. OR: odds ratio, CI: confidence interval, EA: effect allele, EAF: effect allele frequency range across all studies, HetP: heterogeneity *P* value, HetISq: heterogeneity statistic.

Supplementary Figure 26. LocusZoom and forest plots for (a) *TSPOAP1*, (b) *ACE* and (c) *ABCA7* loci. The LocusZoom plot is based on the results from Stage I, and the variant in purple is the best associated variant in the Stage I + II meta-analysis. In the forest plots, data are presented as odds-ratio with 95% confidence interval. *P* values are two-sided raw *P* values derived from a fixed-effect meta-analysis. OR: odds ratio, CI: confidence interval, EA: effect allele, EAF: effect allele frequency range across all studies, HetP: heterogeneity *P* value, HetISq: heterogeneity statistic.

Supplementary Figure 27. LocusZoom and forest plots for (a) *KLF16*, (b) *SIGLEC11* and (c) *LILRB2* loci. The LocusZoom plot is based on the results from Stage I, and the variant in purple is the best associated variant in the Stage I + II meta-analysis. In the forest plots, data are presented as odds-ratio with 95% confidence interval. *P* values are two-sided raw *P* values derived from a fixed-effect meta-analysis. OR: odds ratio, CI: confidence interval, EA: effect allele, EAF: effect allele frequency range across all studies, HetP: heterogeneity *P* value, HetISq: heterogeneity statistic.

Supplementary Figure 28. LocusZoom and forest plots for (a) *RBCK1*, (b) *CASS4* and (c) *SLC2A4RG* loci. The LocusZoom plot is based on the results from Stage I, and the variant in

purple is the best associated variant in the Stage I + II meta-analysis. In the forest plots, data are presented as odds-ratio with 95% confidence interval. *P* values are two-sided raw *P* values derived from a fixed-effect meta-analysis. OR: odds ratio, CI: confidence interval, EA: effect allele, EAF: effect allele frequency range across all studies, HetP: heterogeneity *P* value, HetISq: heterogeneity statistic.

Supplementary Figure 29. LocusZoom and forest plots for (a) *APP* and (b) *ADAMTS1* loci. The LocusZoom plot is based on the results from Stage I, and the variant in purple is the best associated variant in the Stage I + II meta-analysis. In the forest plots, data are presented as odds-ratio with 95% confidence interval. *P* values are two-sided raw *P* values derived from a fixed-effect meta-analysis. OR: odds ratio, CI: confidence interval, EA: effect allele, EAF: effect allele frequency range across all studies, HetP: heterogeneity *P* value, HetISq: heterogeneity statistic.

Supplementary Figure 30. Comparison of ORs observed in the diagnosed cases only analysis and estimated in Stage I including ADD-proxy cases for the genome-wide significant loci. OR: odds-ratio

Supplementary Figure 31. Forest plots of the 8 *HLA* alleles associated with AD (FDR *P* below 0.05). Data are presented as odds-ratio with 95% confidence interval. Two-sided *P* values were derived from a fixed-effect meta-analysis and adjusted *P* values were computed using the false discovery rate method. AF: allele frequency, HetP: heterogeneity *P* value, HetISq: heterogeneity statistic.

Supplementary Figure 32. Forest plots of the 3 three-locus haplotypes associated with AD (FDR *P* below 0.05). Data are presented as odds-ratio with 95% confidence interval. Two-sided *P* values were derived from a fixed-effect meta-analysis and adjusted *P* values were computed using the false discovery rate method. AF: allele frequency, HetP: heterogeneity *P* value, HetISq: heterogeneity statistic.

Supplementary Figure 33. Z-scores for neurodegenerative and AD related diseases for the risk allele of the ADD associated variants. Colors represent the direction of association: the ADD-risk allele increases risk of the disease (red), decreases risk of the disease (blue) or shows no effect (white). Euclidian distances that were clustered according to unweighted pair group method with arithmetic mean (UPGMA) were used (columns and rows). *P* values (uncorrected) are denoted with \$ (P<5x10^{-8}), X (P<1x10^{-5}), # (P<1x10^{-3}), * (P<0.05). The *P* values are based on meta-analyses of GWAS summary statistics (details are described in the following references: Creutzfeldt-Jakob disease (CJD)[122], Dementia with Lewy-bodies (DLB)[123], amyotrophic lateral sclerosis (ALS)[124], Frontotemporal dementia (FTD)[125], Parkinson's disease (PD)[126], ischemic brain infarcts (MRI defined)[127] and ischemic stroke (clinical)[128], white matter hyperintensities (WMH)[129]). The following variants were not shown as they were not found (or a proxy) in more than half of the traits: rs141749679:SORT1, rs143332484:TREM2, rs75932628:TREM2, rs1160871:JAZF1, rs143080277:NCK2, rs35048651:WDR81, rs139643391:WDR12, rs616338:ABI3, rs149080927:KLF16. These are mainly the rarer variants or indels. CJD: Creutzfeldt-Jakob disease, DLB: Dementia with Lewy-bodies, ALS: amyotrophic lateral sclerosis, FTD: Frontotemporal dementia, PD: Parkinson's disease, WMH: white matter hyperintensities, MRI: magnetic resonance imaging.

Supplementary Figure 34. Overview of the gene prioritization strategy used in the study. Scoregene is the total weighted gene prioritization score for a gene, Scoretop is the top score observed in the investigated locus, RelDiffgene is the relative score difference for each gene in the investigated locus compared to the top-scoring gene, MinRelDifflocus is the minimum RelDiffgene observed in the investigated locus.

Supplementary Figure 35. Summary of weighted scores for each evidence category for all candidate genes in the 42 new genome-wide-significant loci, together with their cell-type specific expression patterns in human brain. Using our gene prioritization methodology, we considered the genes within 1 Mb of each novel lead variant and prioritized a total of 55 genes in 42 new loci at two different confidence levels (31 Tier 1 and 24 Tier 2 genes). The leftmost squares indicate the new locus index number. Here we show all candidate genes, i.e. scored for at least one hit for at least one category in our analyses, including non-coding genes that are not included in gene prioritization and shown with a "*" sign in the beginning of the gene. Different type of evidences is colored based on seven different domains they belong to. Weighted scores for each evidence category are rescaled to a 0-100 scale, and human brain cell-type specific expression proportion (based on average expression) for each gene is rescaled to a 0-100 scale, where darker colors represent higher scores or higher expression proportion. Gene prioritization tier category shows Tier 1 genes with a dark green color, and Tier 2 genes with a light green color. The genes that are not considered for gene prioritization methodology, either for being in *IGH* locus or in an independent locus, are shown in gray in the Tier column. MAFs and CADD (v1.6) PHRED scores for rare and/or protein-altering rare variants are labelled in white, and for the rest of the categories the rescaled values are labeled in black within the respective squares. Mon. Mac.: monocytes and macrophages.

Supplementary Figure 36. eQTL effects of lead variants within novel ADD risk loci in (a) AD-relevant brain regions, LCL, microglia, blood, and (b) in naïve state and stimulated macrophages and monocytes. Overlap of novel lead variants with significant eQTL variants affecting the expression of genes within 1 Mb in *n*=26 different eQTL catalogues. Absolute slope (beta) values of eQTL associations are indicated in increasing scale of point size. Gene expression increasing effect of the risk allele of the lead variant with eQTL association is colored red, and decreasing effect is colored blue. For stimulated macrophage and monocyte eQTL catalogues, "h" stands for hours of exposure to the stimulant, and the stimulants are Influenza, Listeria, Salmonella, IFNg (Interferon gamma), LPS (Lipopolysaccharides), Pam3CSK4 (Pam3CysSerLys4) and R848 (Resiquimod). Index number of novel loci are shown in parentheses.

Supplementary Figure 37. sQTL effects of lead variants within novel ADD risk loci in AD-relevant brain regions, LCL, microglia, and blood. Overlap of novel lead variants with significant sQTL variants affecting the alternative splicing of genes within 1 Mb in *n*=18 different sQTL catalogues. Absolute slope (beta) values of sQTL associations are indicated in increasing scale of point size. Increasing splice junction preference effect of the risk allele of the lead variant with sQTL association is colored red, and decreasing effect is colored blue. Index number of novel loci are shown in parentheses.

Supplementary Figure 38. (a) mQTL and haQTL effects of lead variants within novel ADD risk loci. Overlap of novel lead variants with significant mQTL and haQTL variants affecting respectively methylation & histone acetylation of the features within 1 Mb in ROSMAP DLPFC mQTL & haQTL catalogues (xQTL Serve). Shapes indicate the positional annotation of the feature for the related gene. Absolute Spearman's rho values of QTL associations are labelled below the shapes. Methylation or histone acetylation increasing effect of the risk allele of the lead variant is colored red, and decreasing effect is colored blue. Index number of novel loci are shown in parentheses. (b) ADD-associated predicted methylation results using MetaMeth. Results for ADD-associated significant (after Bonferroni correction) CpG features are shown, where Z-score of association is indicated in a heatmap scale from -7.5 to +7.5. Each CpG is paired with its annotated gene(s) and respective positional annotation of CpGs are shown in different shapes on the figure. MetaMeth hits are grouped by their percentile and direction of blood-brain methylation correlation estimates across 3 brain regions that were obtained from BECon website. Index number of novel loci are shown in parentheses.

Supplementary Figure 39. Colocalization between eQTL signals for genes and ADD association signals. Colocalization probability results for ADD signal with the eQTL signals of genes within 1 Mb of lead variants of novel ADD risk loci in *n*=12 different eQTL catalogues. Only the genes with at least one significant eQTL (in any included tissues or cell groups) overlapping with at least one suggestively significant (p =< 1E-5) ADD risk variant were tested. Colocalization probability PP4 estimate is indicated in increasing scale of point size and opacity for all tested colocalizations, and scores with at least eQTL coloc PP4 ≥ 70% is labelled on the figure as well. Only colocalized hits at an eQTL coloc PP4 ≥ 70% level in at least one catalogue are shown in this figure. Index number of novel loci are shown in parentheses.

Supplementary Figure 40. Colocalization between sQTL signals for splice junctions and ADD association signals. Colocalization probability results for ADD signal with the sQTL signals of splice junctions within 1 Mb of lead variants of novel ADD risk loci in *n*=12 different sQTL catalogues. Only the splice junctions with at least one significant sQTL (in any included tissues or cell groups) overlapping with at least one suggestively significant (*P* =< 1E-5) AD risk variant were tested. Colocalization probability PP4 estimate is indicated in increasing scale of point size and opacity for all tested colocalizations, and scores with at least sQTL coloc PP4 ≥ 70% is labelled on the figure as well. Only colocalized hits at a sQTL coloc PP4 ≥ 70% level in at least one catalogue are shown in this figure. Index number of novel loci are shown in parentheses.

Supplementary Figure 41. TWAS of ADD using Expression Reference Panels. Expression TWAS (eTWAS) results for genes within 1 Mb of lead variants in *n*=13 different expression reference panels used. For each expression reference panel, expression models that are not available in the respective panel are shown as dark gray. TWAS Z-score of association is indicated in a heatmap scale from -10 to +10. Significant eTWAS associations that are passing Bonferroni-corrected significance level threshold per reference panel are labelled with asterisk ("*"), fine-mapped eTWAS associations are labelled with a dagger ("†") along with PIP value, eQTL colocalizations with coloc PP4 ≥ 70% are labelled with "C". Associations are only illustrated if significant in at least one panel. Index number of novel loci are shown in parentheses.

Supplementary Figure 42. TWAS of ADD using Splicing Reference Panels. Splicing TWAS (sTWAS) results for splice junctions within 1 Mb of lead variants in *n*=13 different splicing reference panels used. For each splicing reference panel, splice junctions that are not available in the respective panel are shown as dark gray. TWAS Z-score of association is indicated in a heatmap scale from -10 to +10. Significant sTWAS associations that are passing Bonferroni-corrected significance level threshold per prediction panel is labelled on the figure with asterisk ("*") and sQTL colocalizations with coloc PP4 ≥ 70% are labelled with "C". Associations are only illustrated if significant in at least one panel. Index number of novel loci are shown in parentheses.

Supplementary Figure 43. PWAS of ADD using Protein Expression Reference Panels, as reported by Wingo et al., 2021. Protein-wide association study (PWAS) were conducted using Jansen et al., 2019 GWAS. PWAS results are shown for genes within 1 Mb of lead variants in *n*=2 different protein expression reference panels used. Protein expression models that are not available in the respective panel are shown as dark gray. PWAS Z-score of association is indicated in a heatmap scale from -10 to +10. Significant PWAS associations that are passing FDR-corrected significance level threshold per reference panel are labelled with asterisk ("*"), and pQTL colocalizations with coloc PP4 ≥ 70% are labelled with "C". Associations are only illustrated if significant in at least one panel. Index number of novel loci are shown in parentheses.

Supplementary Figure 44. Fine-mapping of expression TWAS results. Representative examples of fine-mapping of eTWAS results are shown below in the regional plots (generated by FOCUS) when multiple significant eTWAS hits are observed in the same expression reference panel. Posterior inclusion probability (PIP) values are shown in increasing point size, -log10 of marginal TWAS p-value is shown on y-axis, and pairwise predicted expression correlations between genes shown below the plots. (a) *GRN* locus in MayoRNASeq TCX, (b) *KLF16* locus in ROSMAP DLPFC, (c) *TSPAN14* locus in MSBB BA36, and (d) *DOC2A* locus in GTEx hippocampus.

Supplementary Figure 45. Mean fluorescence intensity variations (log2 fold-change) of the mCherry signal obtained after the silencing of genes associated with the ADD risk in HEK293 cells stably over-expressing a mCherry-APP^{695WT}-YFP in the 42 new loci. To evaluate the impact of each siRNA (SMARTPool), an average of 1,000 cells was analysed in triplicate. The mean fluorescence intensity was normalized to the fold change based on the non-targeting siRNA. Bars indicate the means ± S.D.

Supplementary Figure 46. STRING protein interaction analysis. The main networks are shown in a) previous genes, b) prioritized new genes and c) combined datasets. A significantly larger than expected network of interacting genes was observed in the set of previously identified genes ($P < 2x10^{-5}$), in the prioritized gene set in the new loci ($P = 2.8x10^{-3}$) and also in the combination of these two gene sets ($P < 2x10^{-5}$).

Supplementary Figure 47. Association between the GRS and the risk of progression to all-cause dementia in population-based cohorts (n=17,545 independent samples) (A) and MCI cohorts (n=4,114 independent samples) (B).
Data are presented as Hazard ratios (HR) together with 95% confidence intervals (95% CI) derived from Cox-regression analyses for each individual cohort. HRs indicate the effect of the GRS as the increment in the AD risk associated with each additional average risk allele in the GRS. Null Hypothesis testing is based on a meta-analysis of individual cohort effects using fixed effects (FE) and random effects (RE) models. Resulting HRs and 95%-CIs and the respective Z-test and associated two-sided *P* value are shown at the bottom of the figure. Heterogeneity between cohorts is indicated by the index I2 index together with the respective Cochran’s Q statistic (distributed as Chi² statistic), associated degrees of freedom, and *P* value. 3C: the 3 City study, AgeCoDe: German study on aging cognition and dementia, FHS: Framingham heart study, RS1: Rotterdam study first cohort, RS2: Rotterdam study second cohort, VITA: Vienna-Transdanube-Aging-study, MAS: Sydney Memory and Ageing Study, FACE: Fundacio ACE memory clinic cohort, AMC: Additional, independent memory clinic cohort from Fundacio ACE, UAN: memory clinic cohort from the Hospital Network Antwerp, DCN: German dementia competence network study, HAN: BALTAZAR multicenter prospective memory clinic study, UHA: University of Halle memory clinic cohort, ZIM: The Heidelberg/Mannheim memory clinic sample

Supplementary Figure 48. TNF complex and signaling. In red are indicated the proteins coded by ADD genetic risk factors. Binding of TNF induces trimerisation of TNFR1. This leads to recruitment and modulation of TNF core complex I components. OTULIN counteracts auto-ubiquitination of LUBAC. This allows to LUBAC recruitment to complex I. Presence of LUBAC stabilizes this complex and gene-activator signaling (NK-kB, AP-1). Absence of LUBAC favours apoptosis/necroptosis (from Spit et al, 2019). cIAP, cellular inhibitor of aptoptose. CYLD, CYLD lysine 63 deubiquitinase. HOIL-1 (*RBCK1*), heme-oxidized iron-responsive element-binding protein 2 ubiquitin ligase-1. HOIP (*RNF31*), HOIL-1-interacting protein. IκK, inhibitor of κB kinase. NEMO, NF-κb essential modulator. RIPK1, receptor-interacting protein kinase 1. SHARPIN, SH3 and multiple ankyrin repeat domains protein (SHANK)-associated RBCK1 homology (RH)-domain-interacting protein. SPATA2, spermatogenesis associated 2. TAB1/2, TAK binding protein 1/2. TAK1, TGF-β activated kinase 1. TBK1, TANK bindink kinase. TNF, tumor necrosis factor. TNFR, tumor necrosis

factor receptor. TNIP1, TNFAIP3 interacting protein 1. TRADD, TNFR1 assoiated death domain protein. TRAF2, TNFR-associated factor 2.

Supplementary Figure 49. EADB sample quality control.

Supplementary Figure 50. EADB variant quality control.

Supplementary Figure 1: Stage I QQ Plot. QQ Plot of Stage I meta-analysis results (excludes the *APOE* locus).

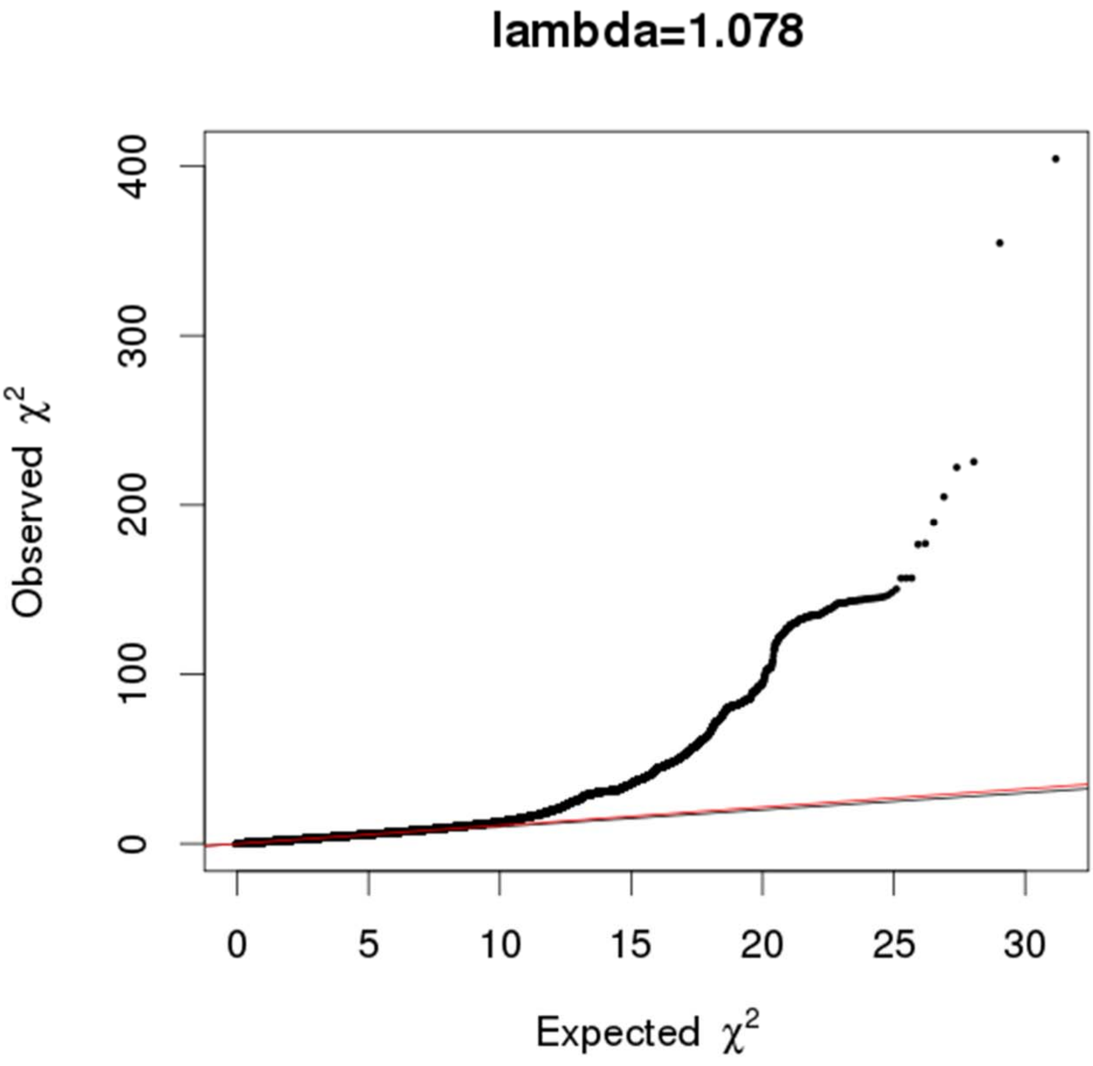

Supplementary Figure 2: LocusZoom and forest plots for (a) *SORT1*, (b) *CR1* and (c) *ADAM17* loci.

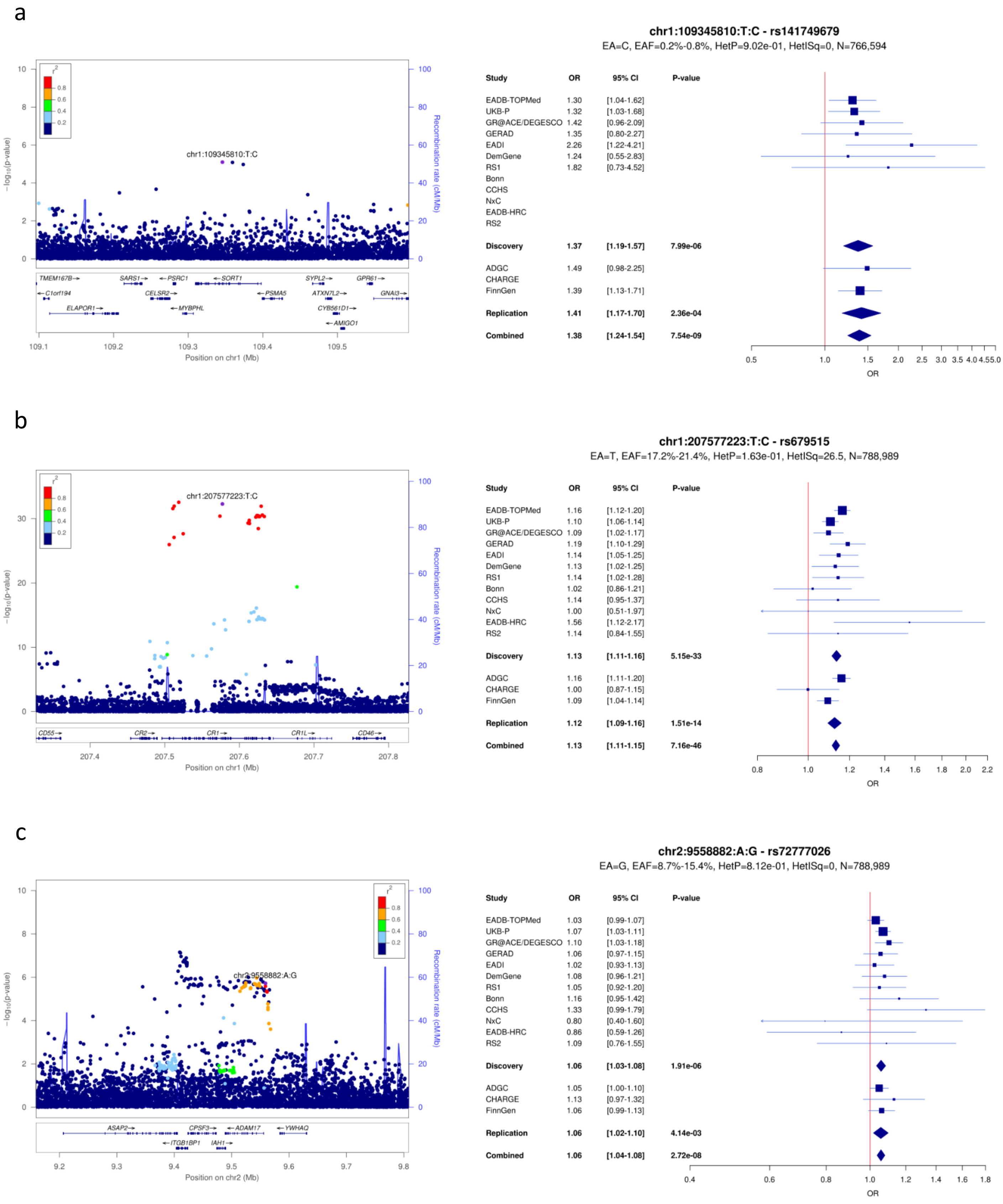

Supplementary Figure 3: LocusZoom and forest plots for (a) *PRKD3*, (b) *NCK2* and (c) *BIN1* loci.

a

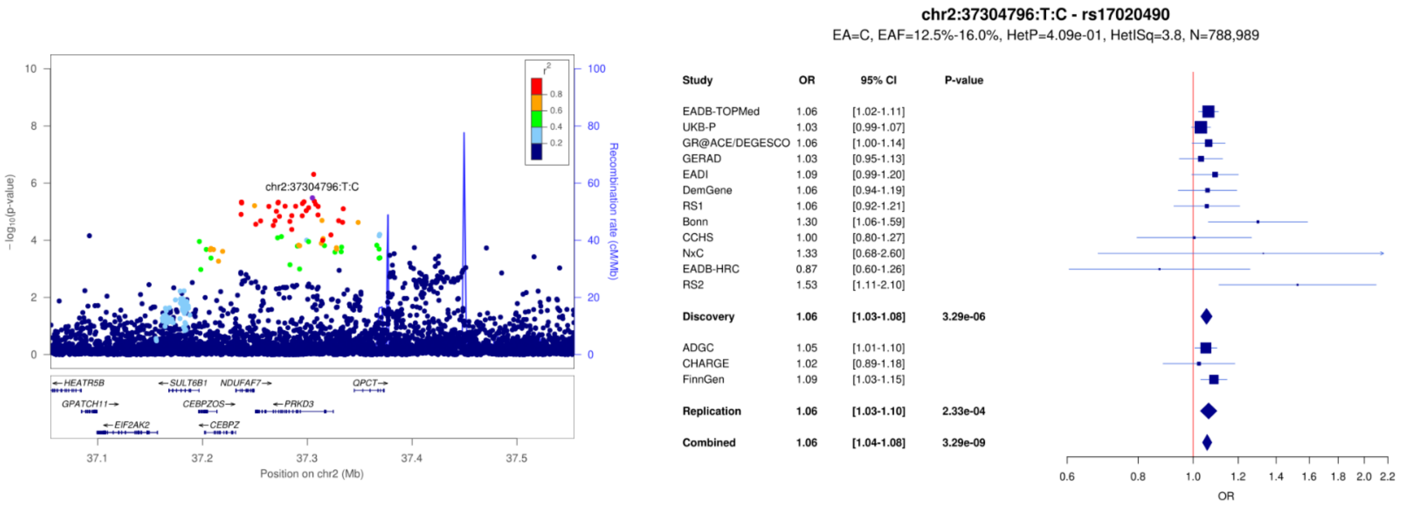

b

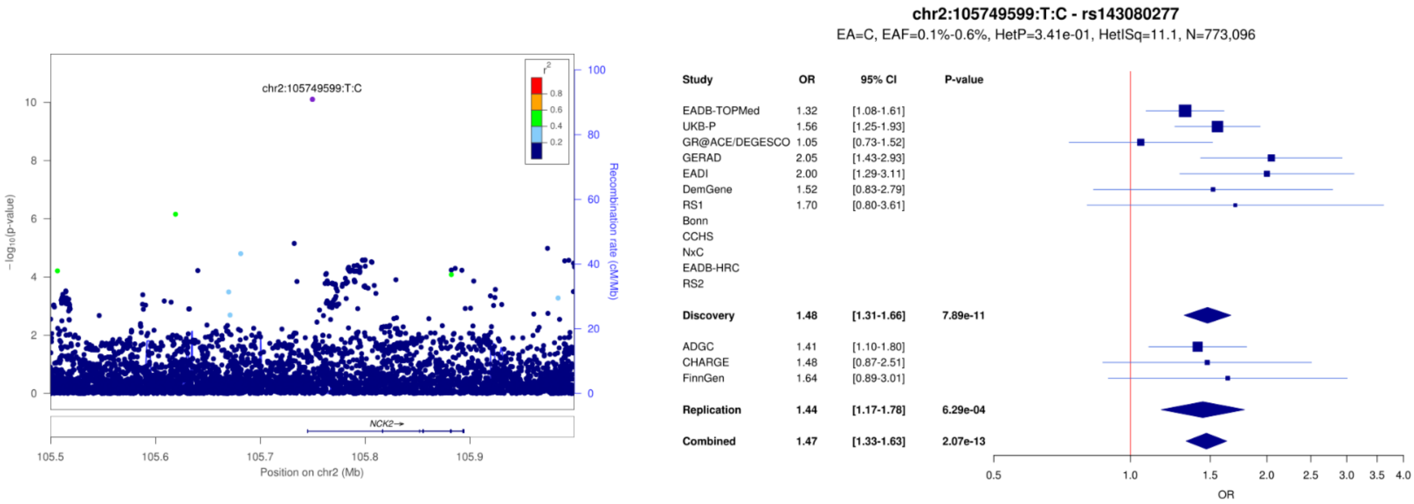

c

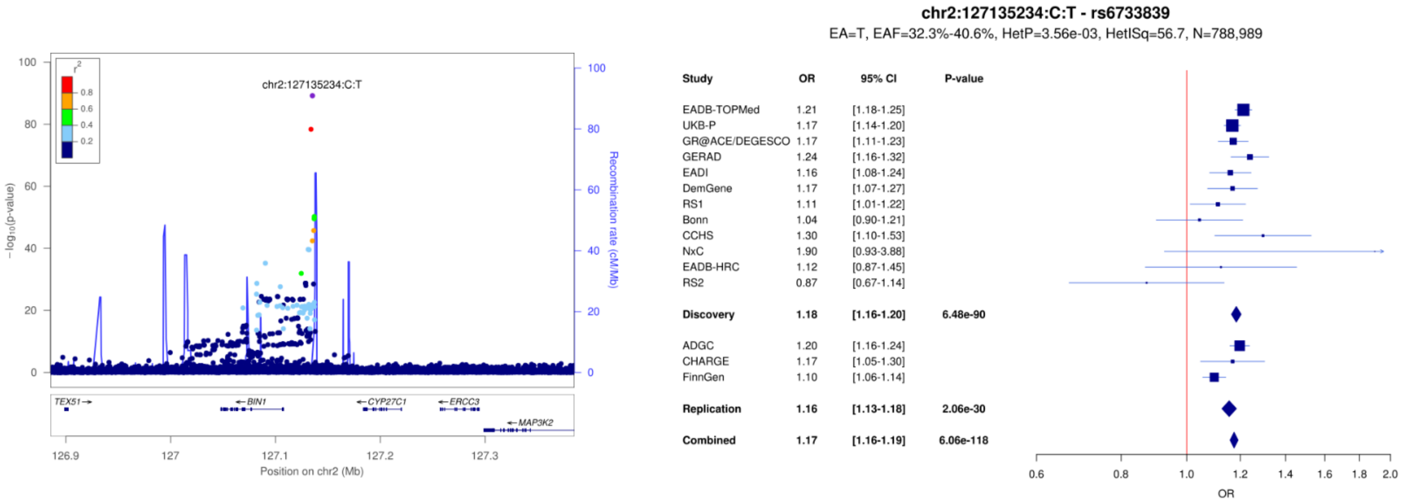

Supplementary Figure 4: LocusZoom and forest plots for (a) *WDR12*, (b) *INPP5D* and (c) *MME* (1) loci.

a

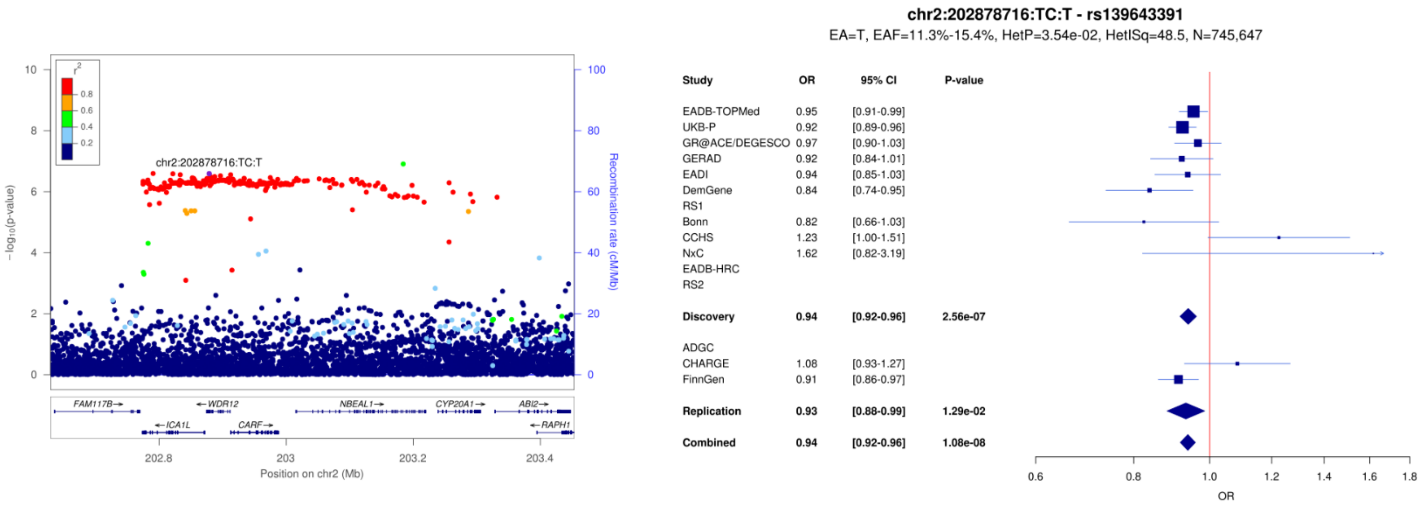

b

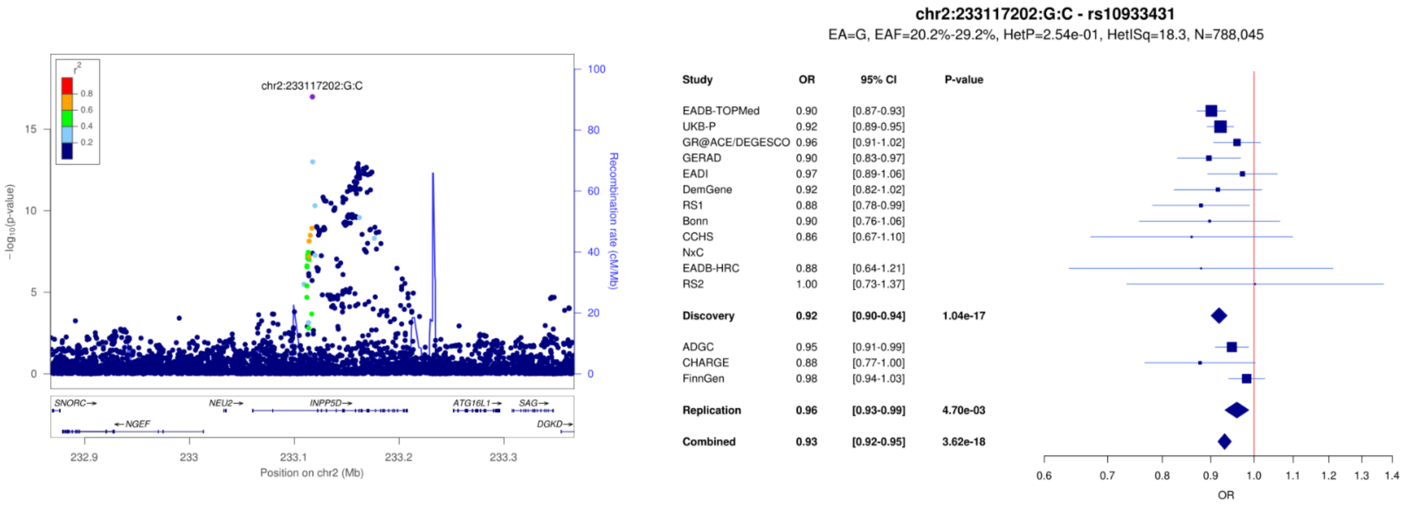

c

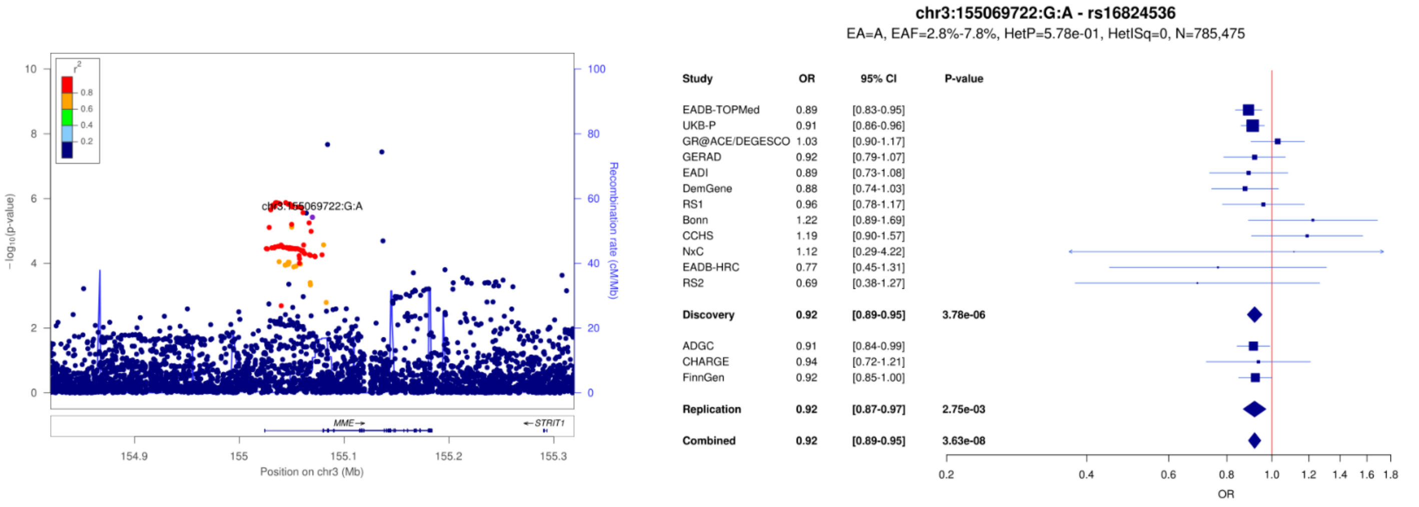

Supplementary Figure 5: LocusZoom and forest plots for (a) *MME* (2), (b) *IDUA* and (c) *CLNK* loci.

a

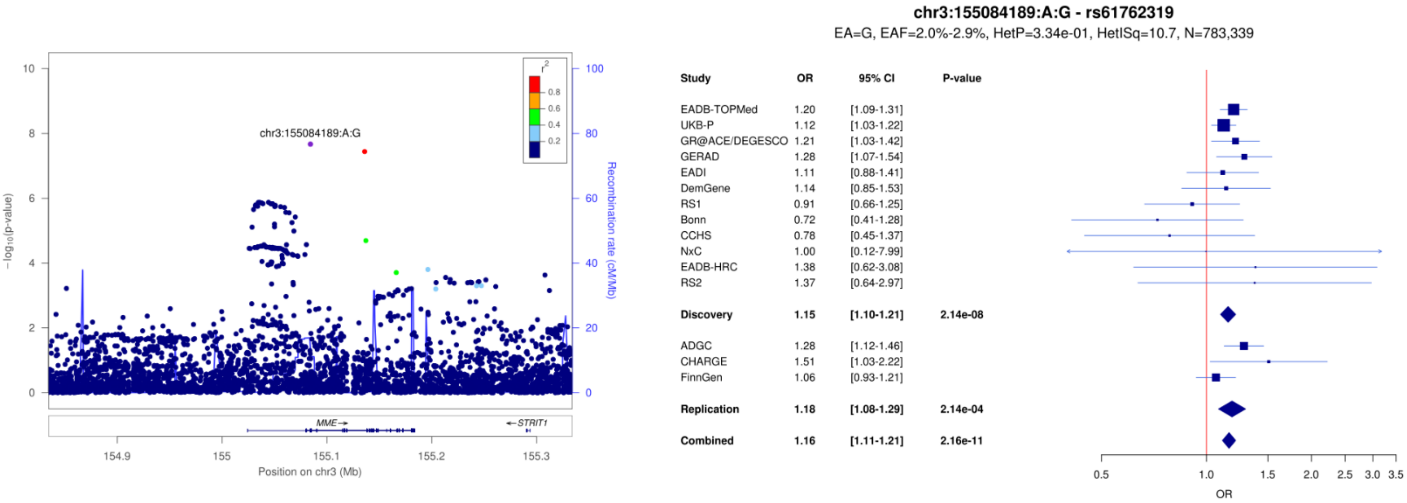

b

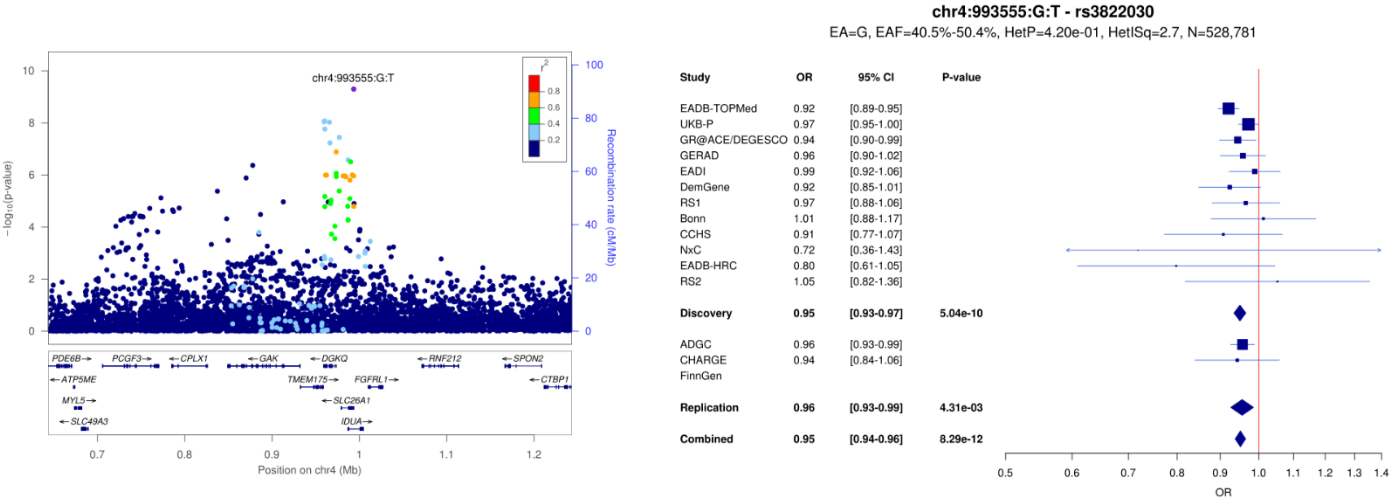

c

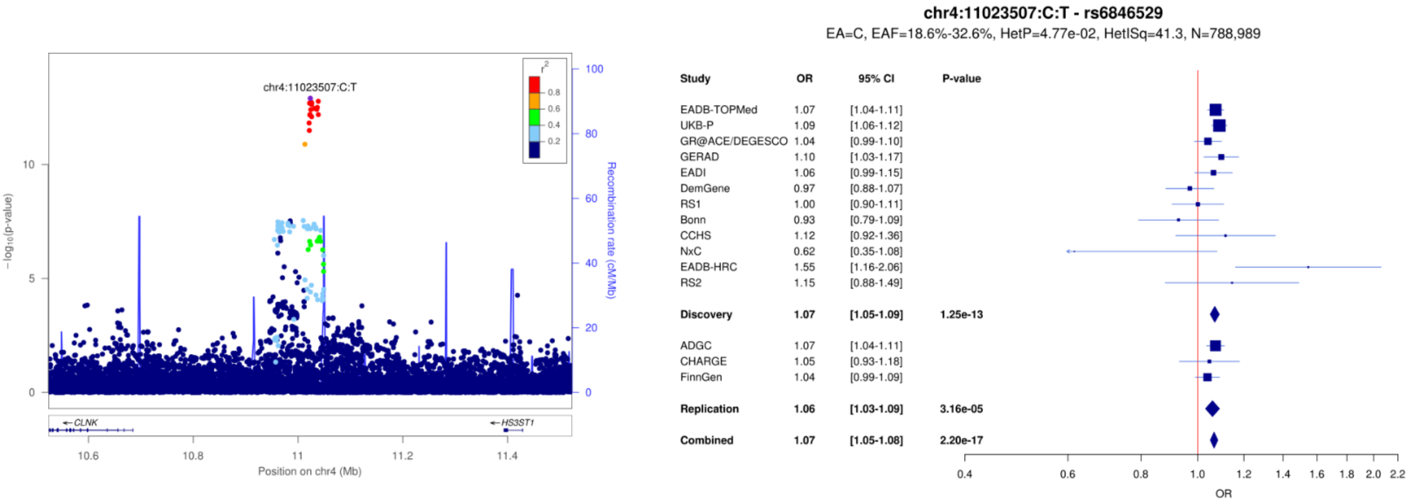

Supplementary Figure 6: LocusZoom and forest plots for (a) *RHOH*, (b) *ANKH* and (c) *COX7C* loci.

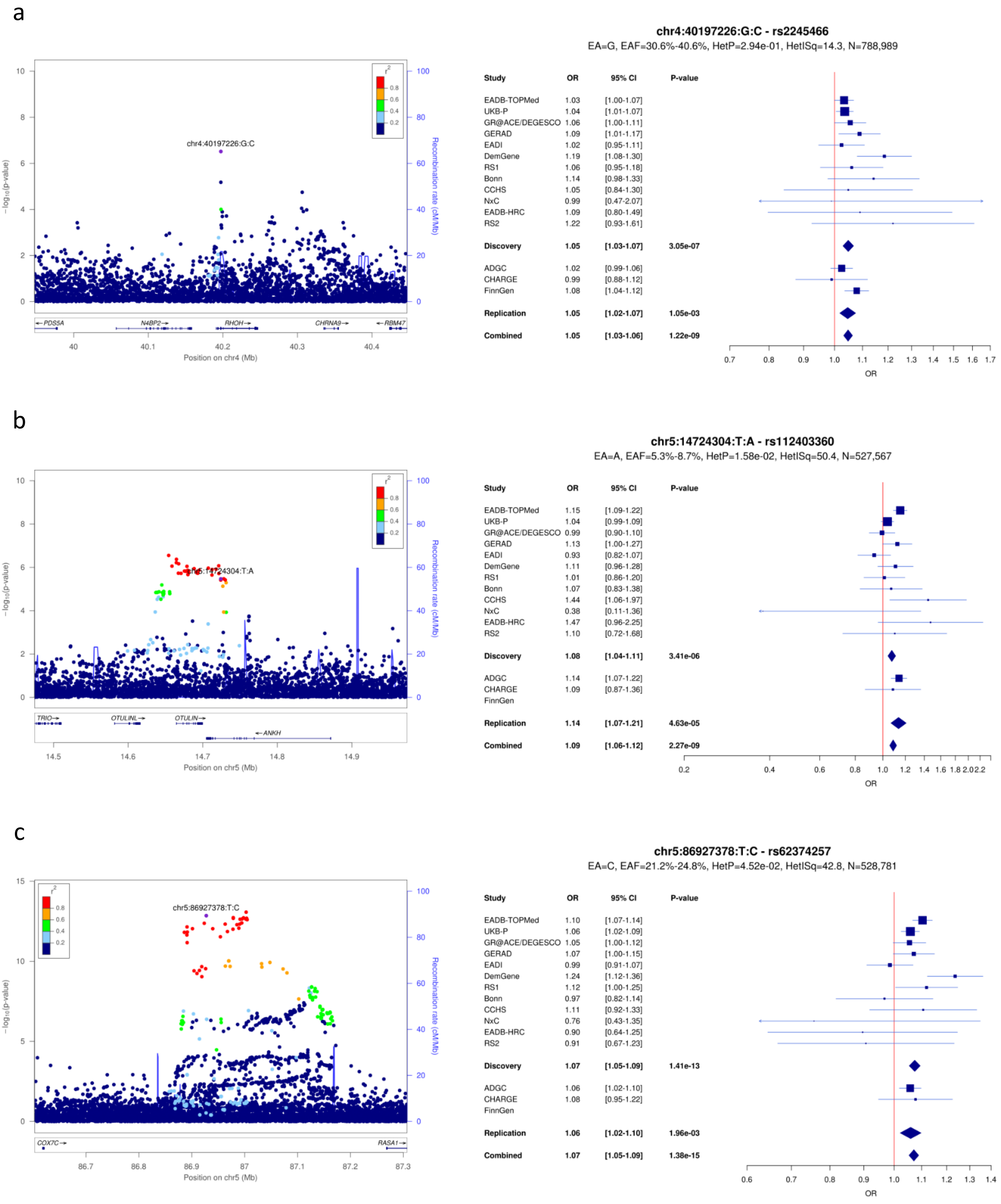

Supplementary Figure 7: LocusZoom and forest plots for (a) *TNIP1*, (b) *RASGEF1C* and (c) *HLA-DQA1* loci.

a

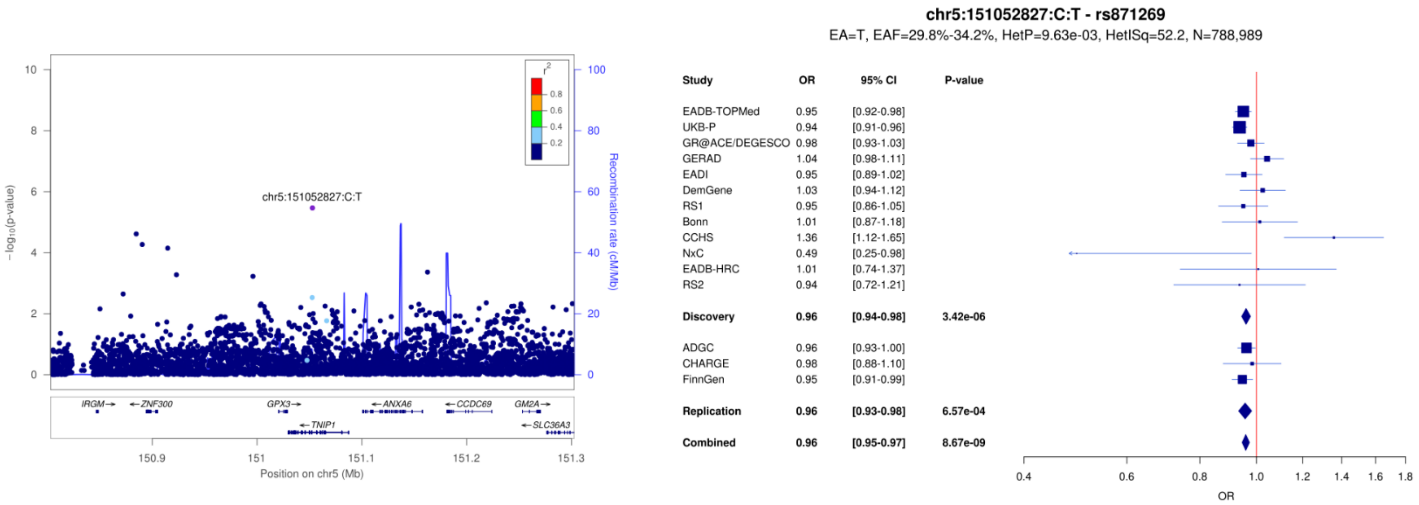

b

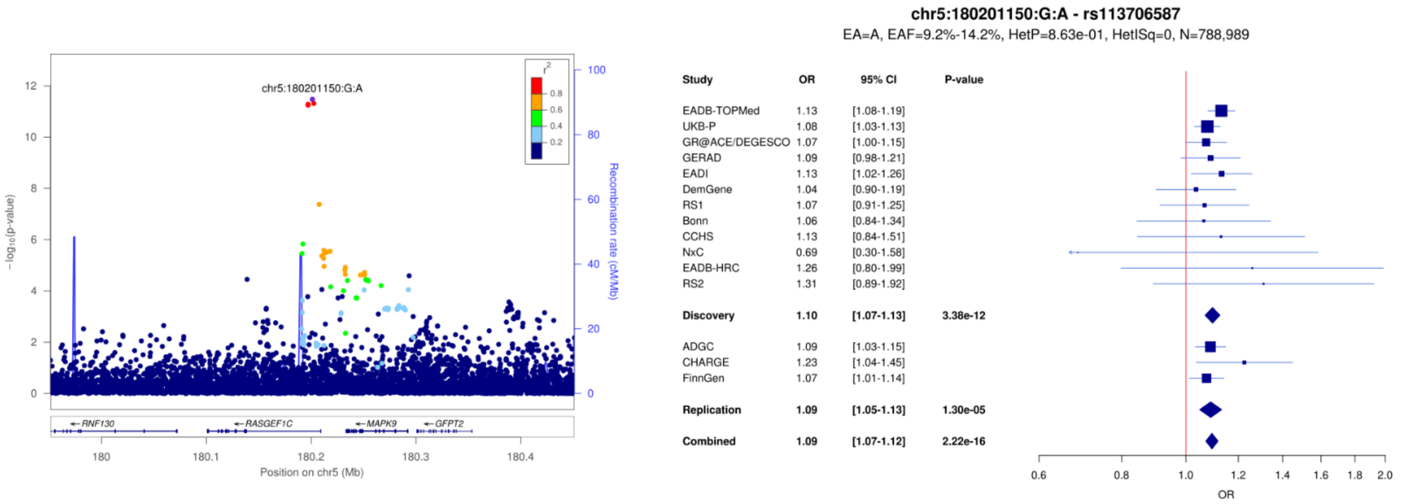

c

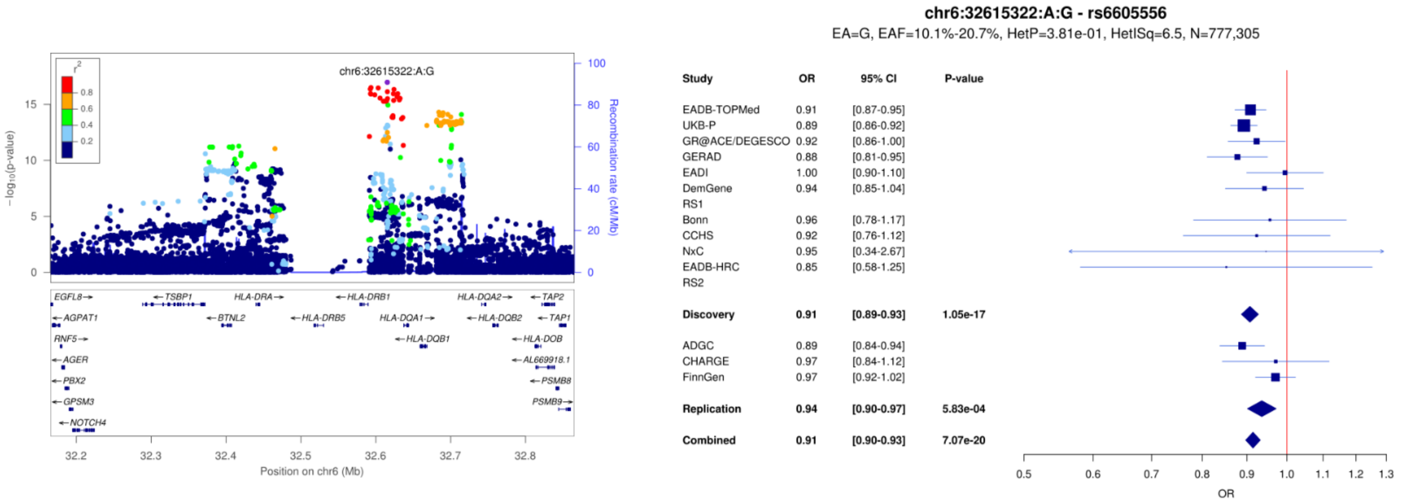

Supplementary Figure 8: LocusZoom and forest plots for (a) *UNC5CL*, (b) *TREM2* (R62H) and (c) *TREM2* (R47H) loci.

a

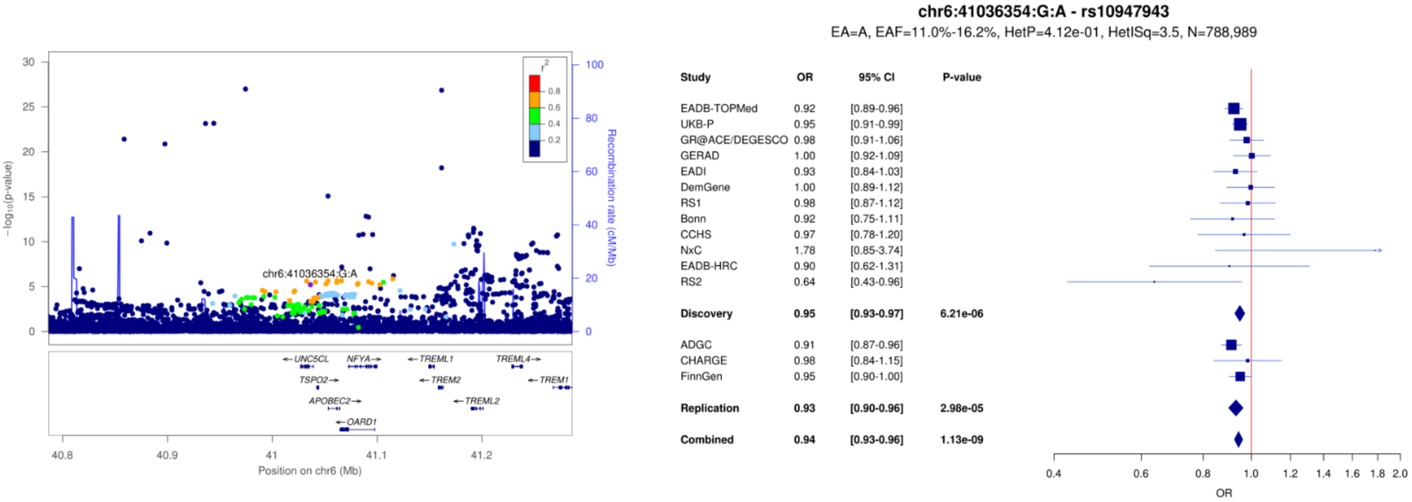

b

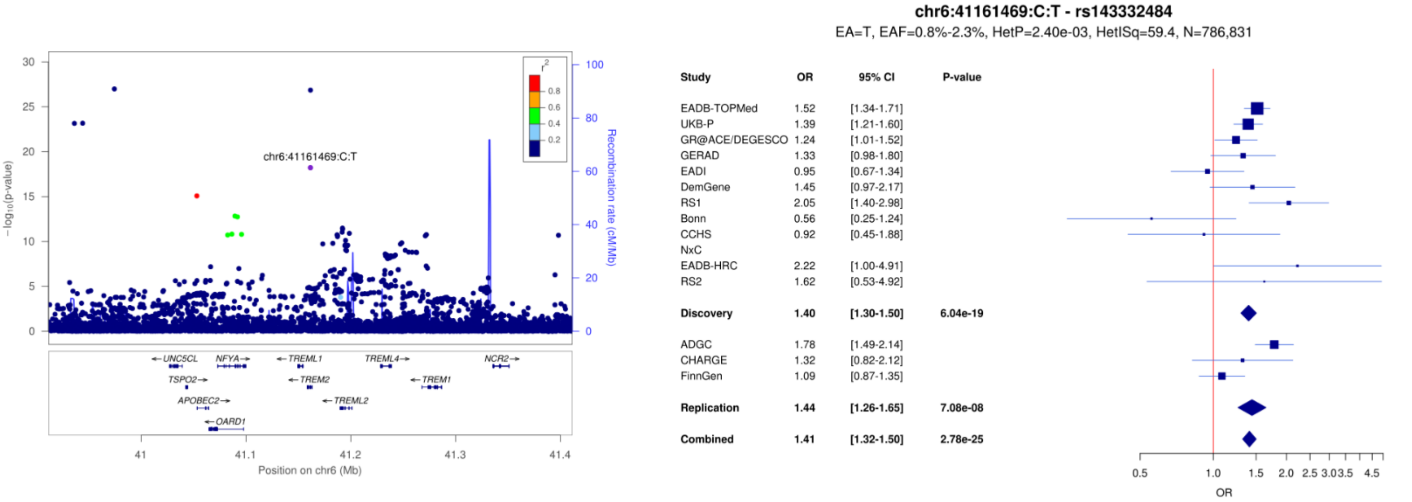

c

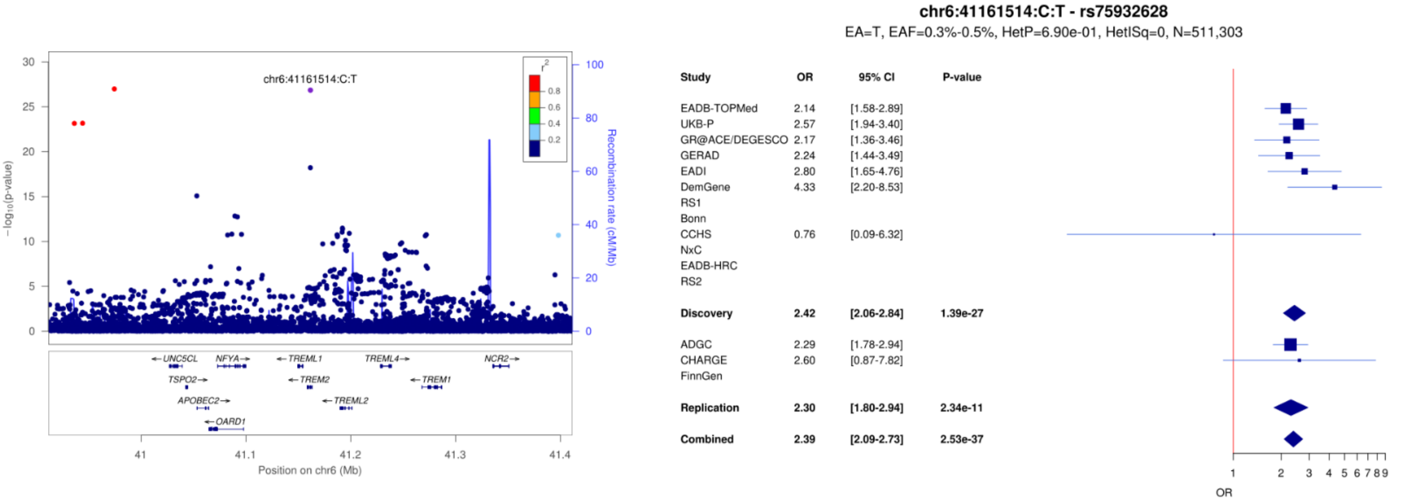

Supplementary Figure 9: LocusZoom and forest plots for (a) *TREML2*, (b) *CD2AP* and (c) *HS3ST5* loci.

a

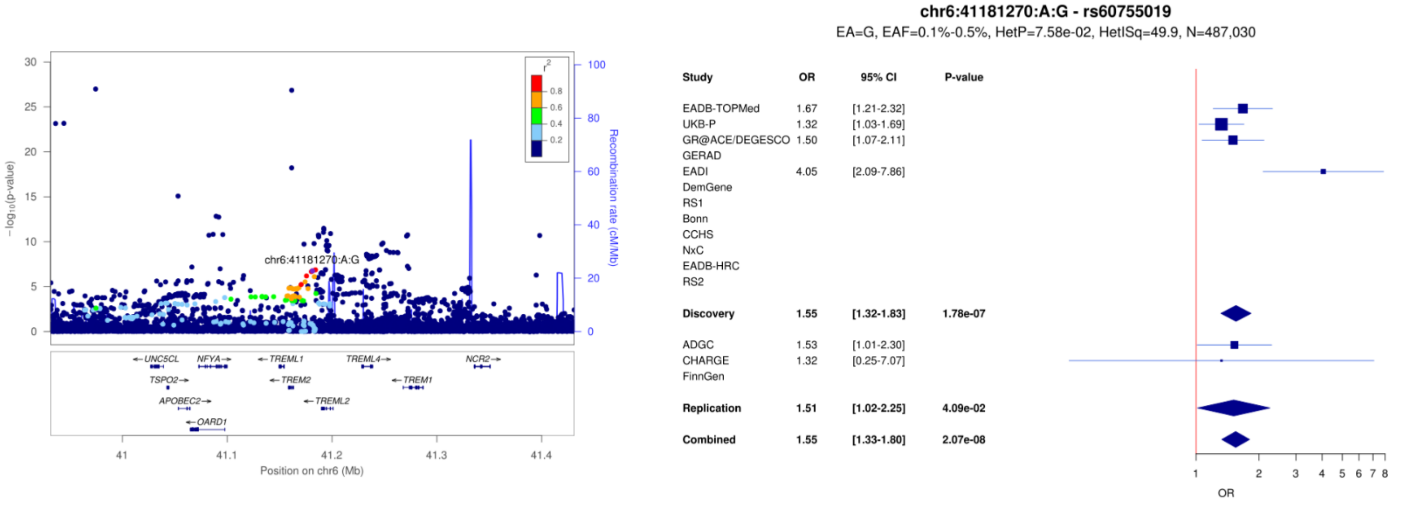

b

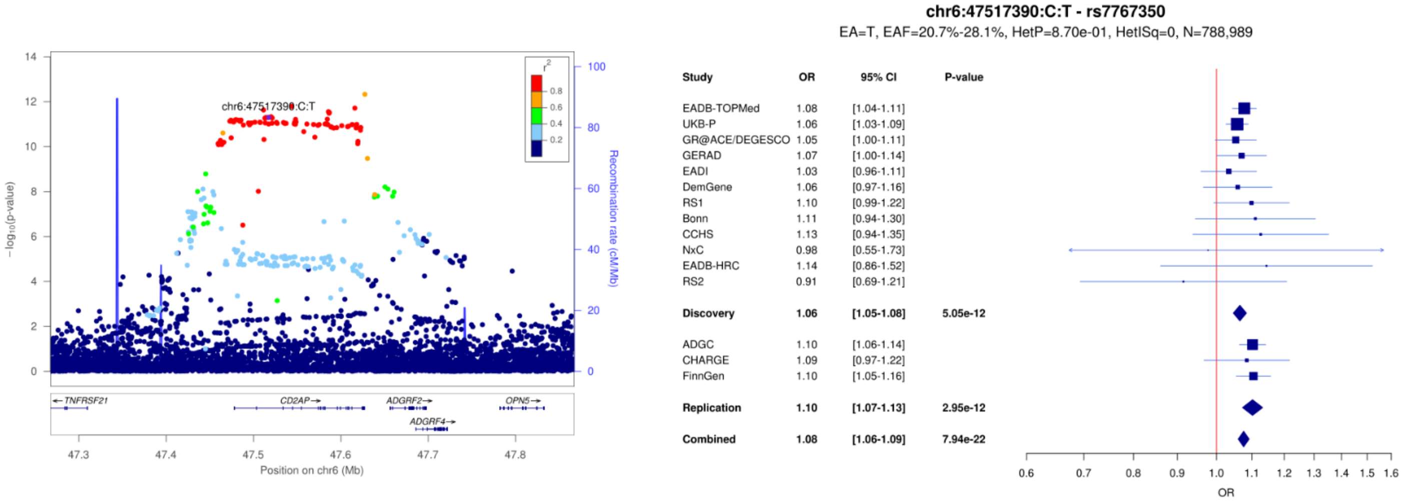

c

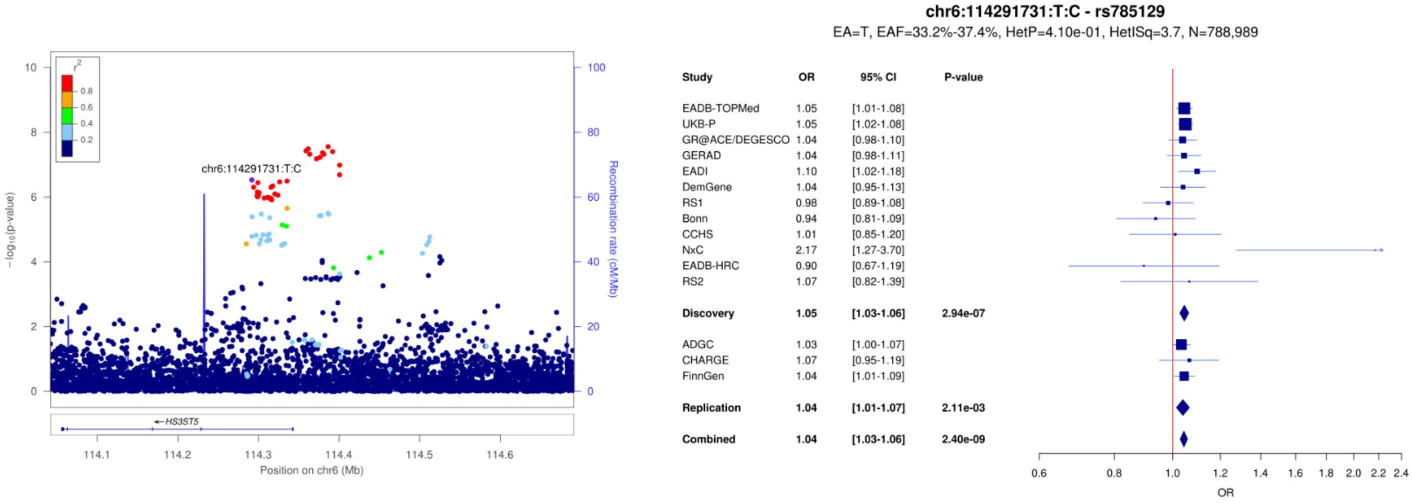

Supplementary Figure 10: LocusZoom and forest plots for (a) *UMAD1*, (b) *ICA1* and (c) *TMEM106B* loci.

a

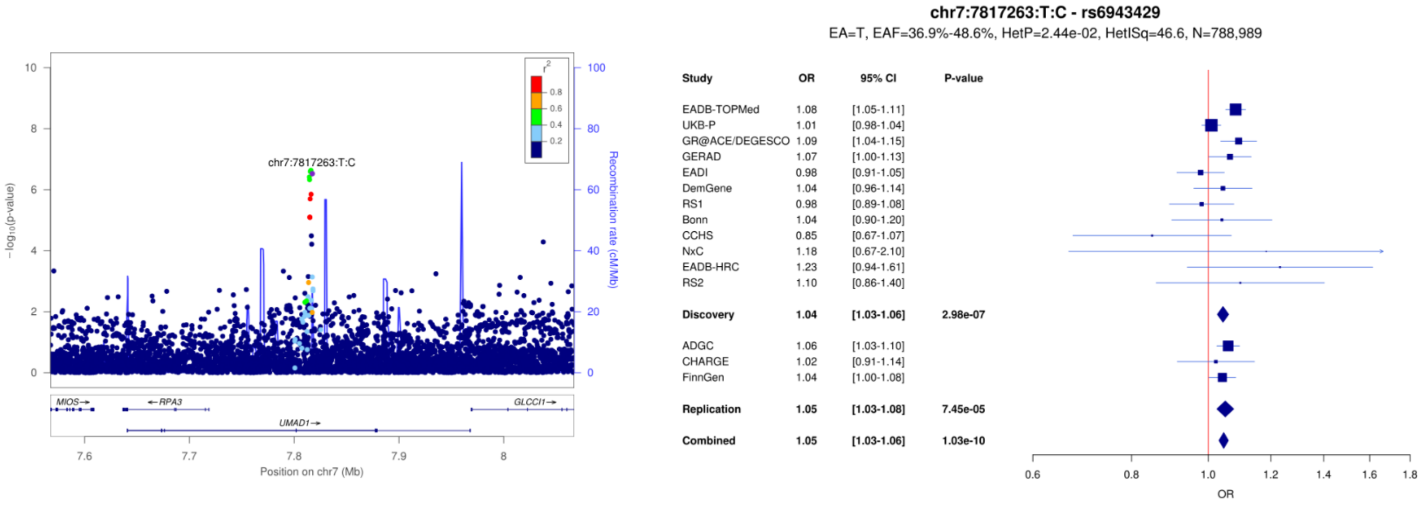

b

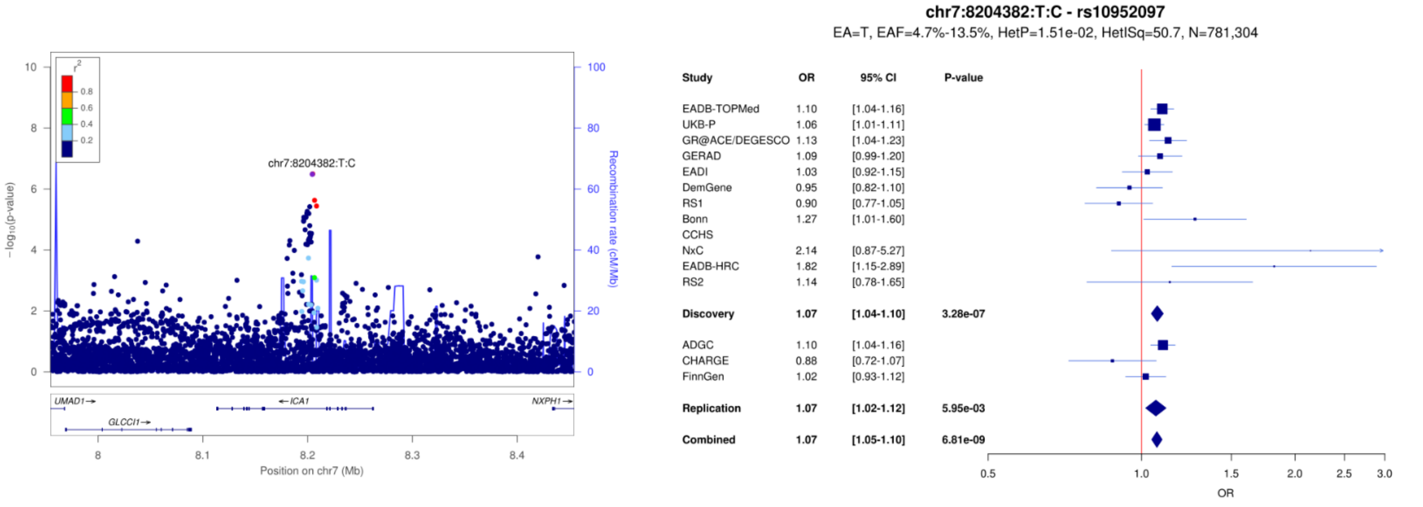

c

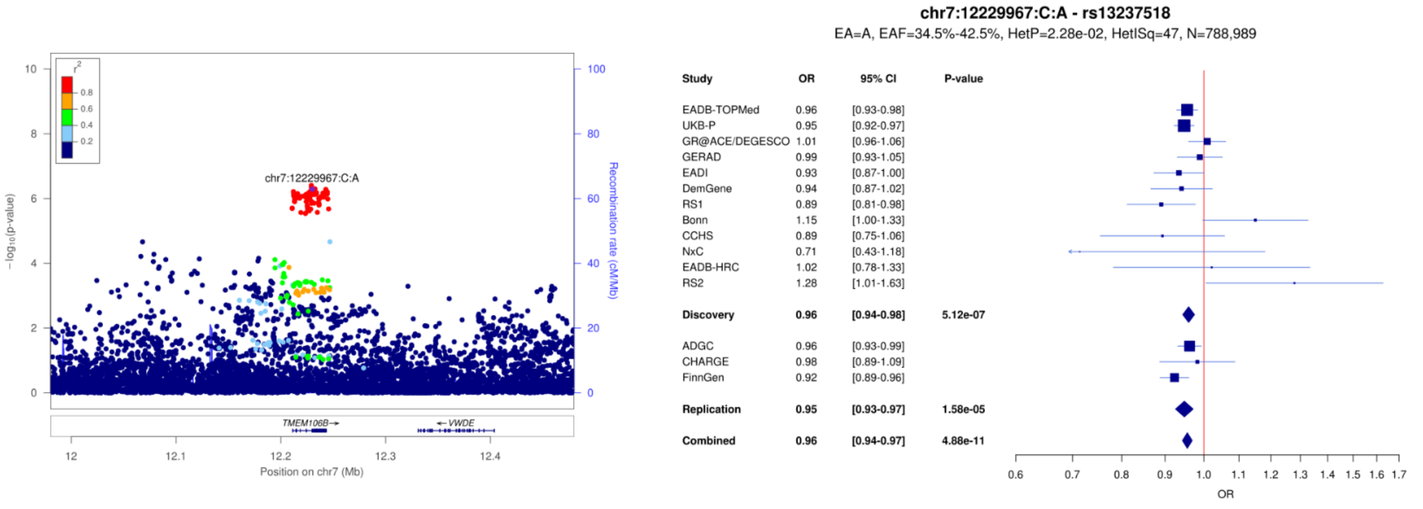

Supplementary Figure 11: LocusZoom and forest plots for (a) *JAZF1,* (b) *NME8* and (c) *SEC61G* loci.

a

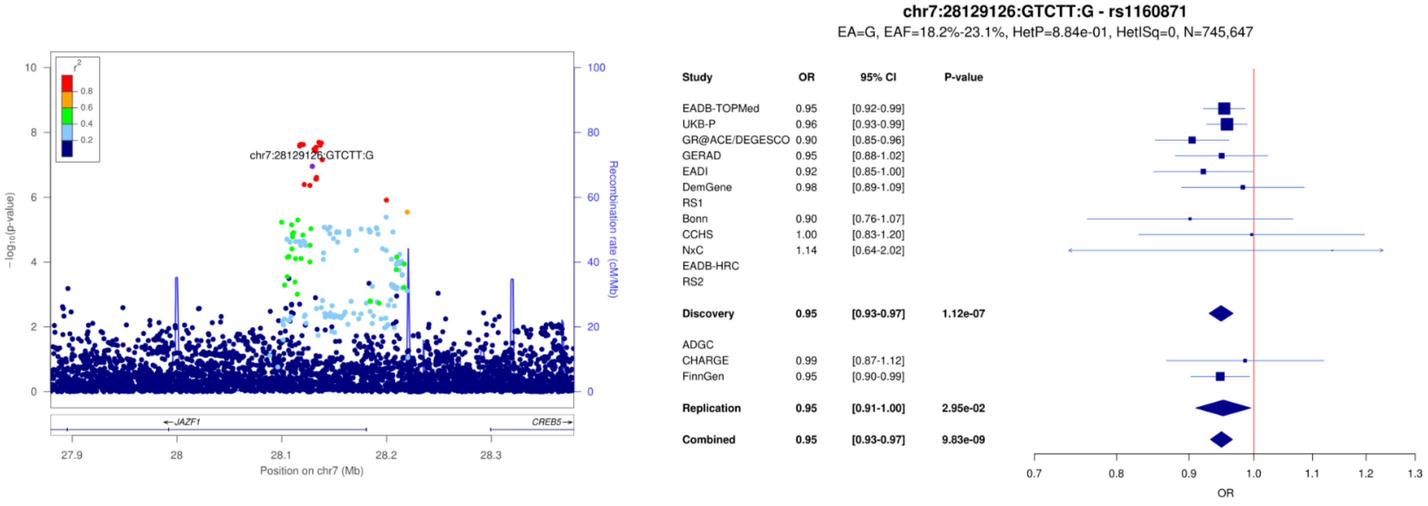

b

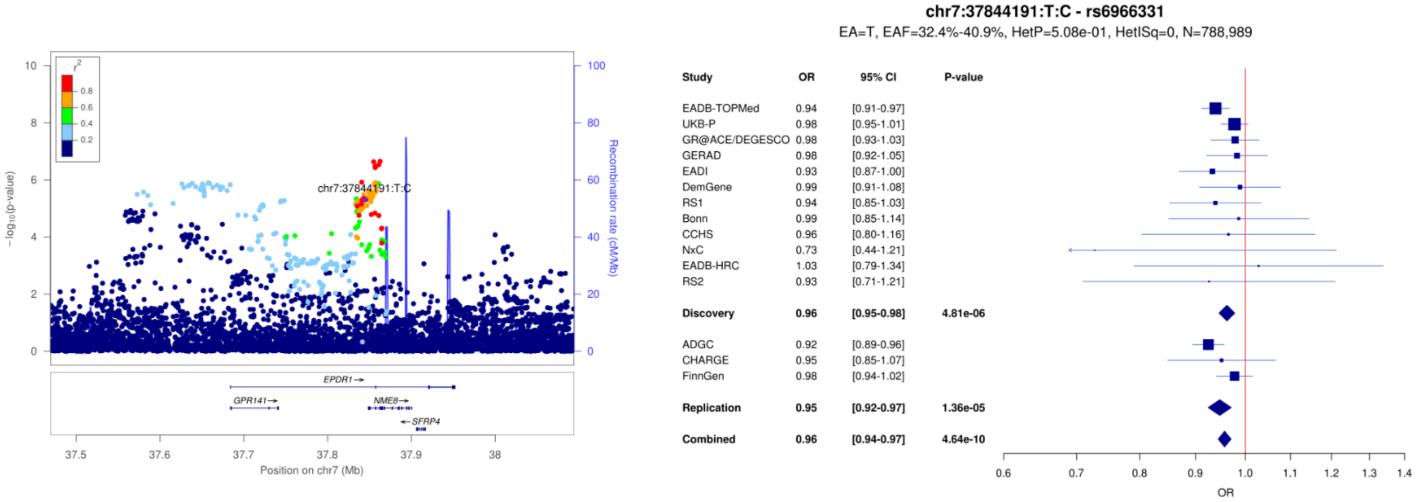

c

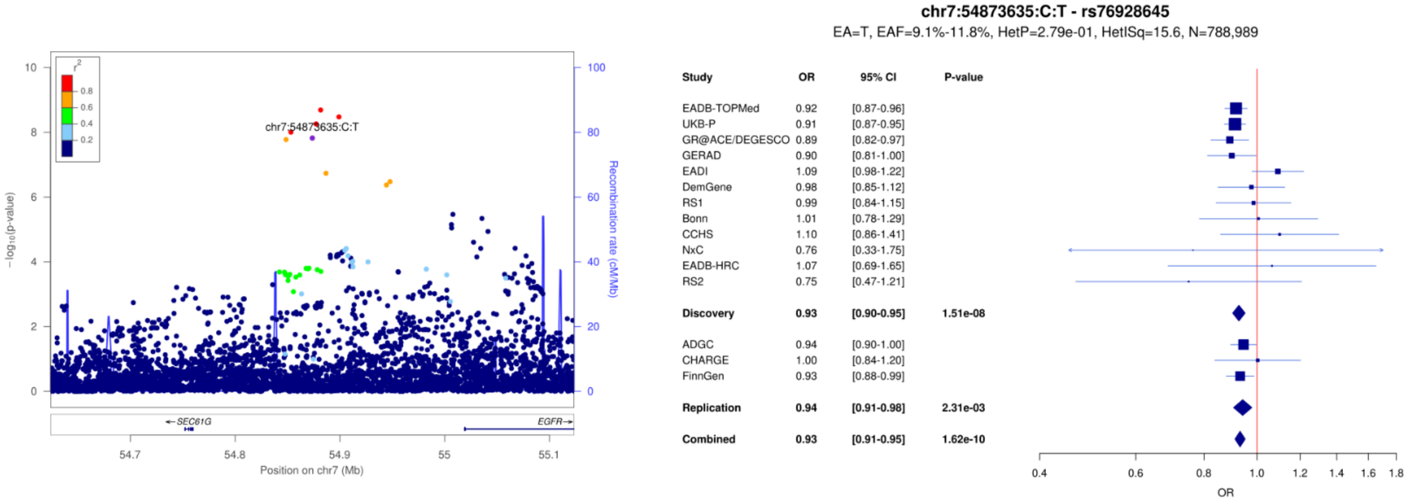

Supplementary Figure 12: LocusZoom and forest plots for (a) *ZCWPW1/NYAP1*, (b) *EPHA1* and (c) *CTSB* loci.

a

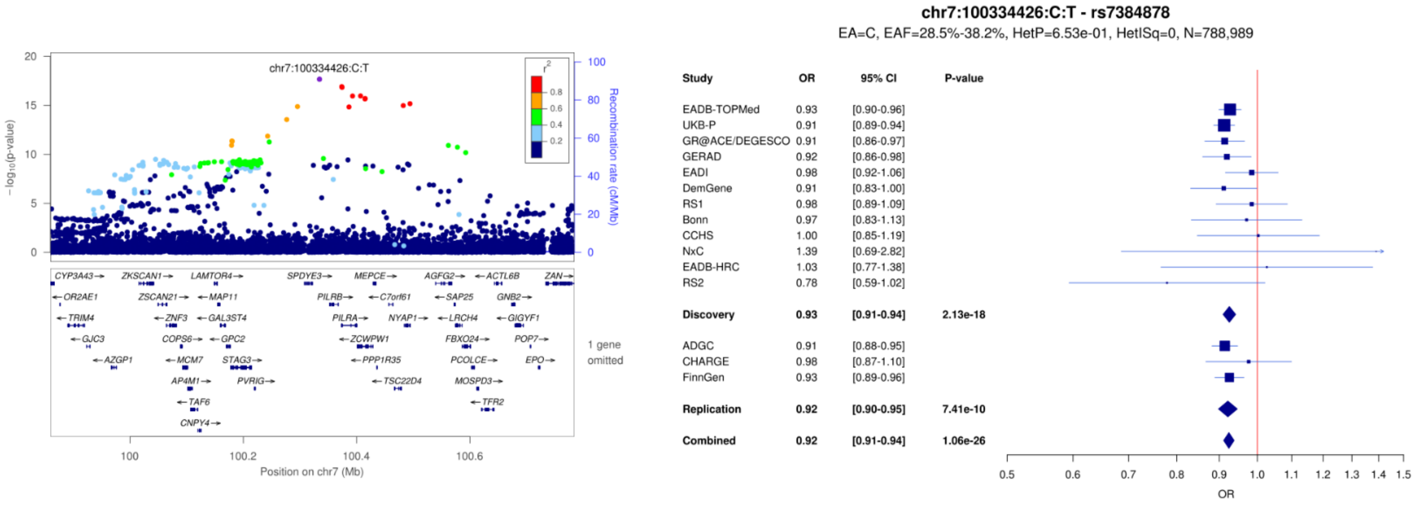

b

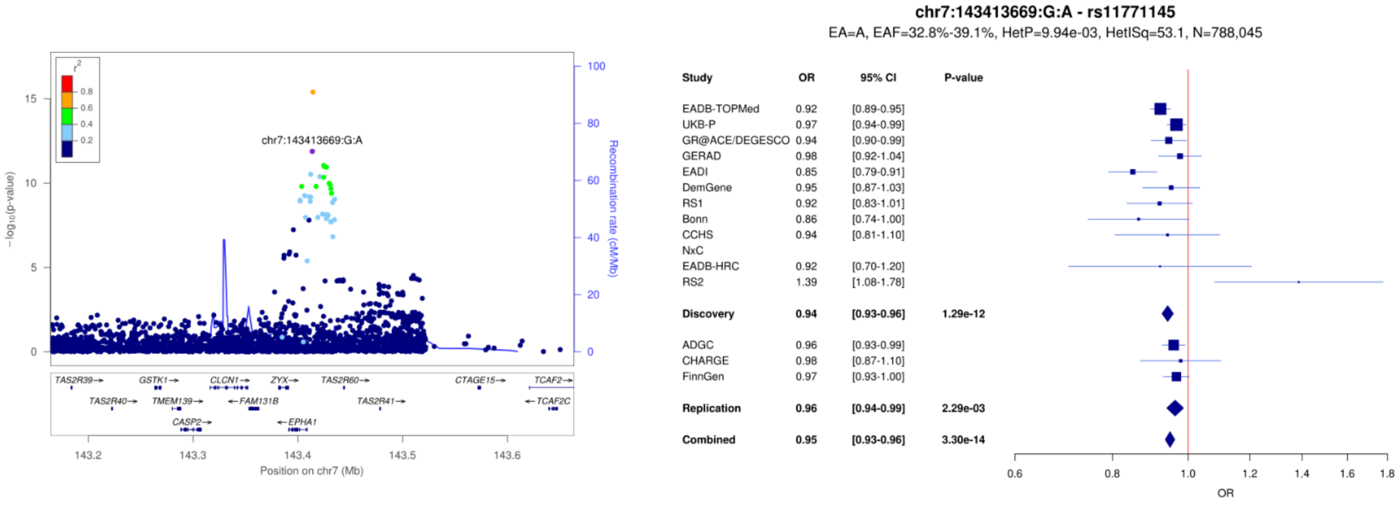

c

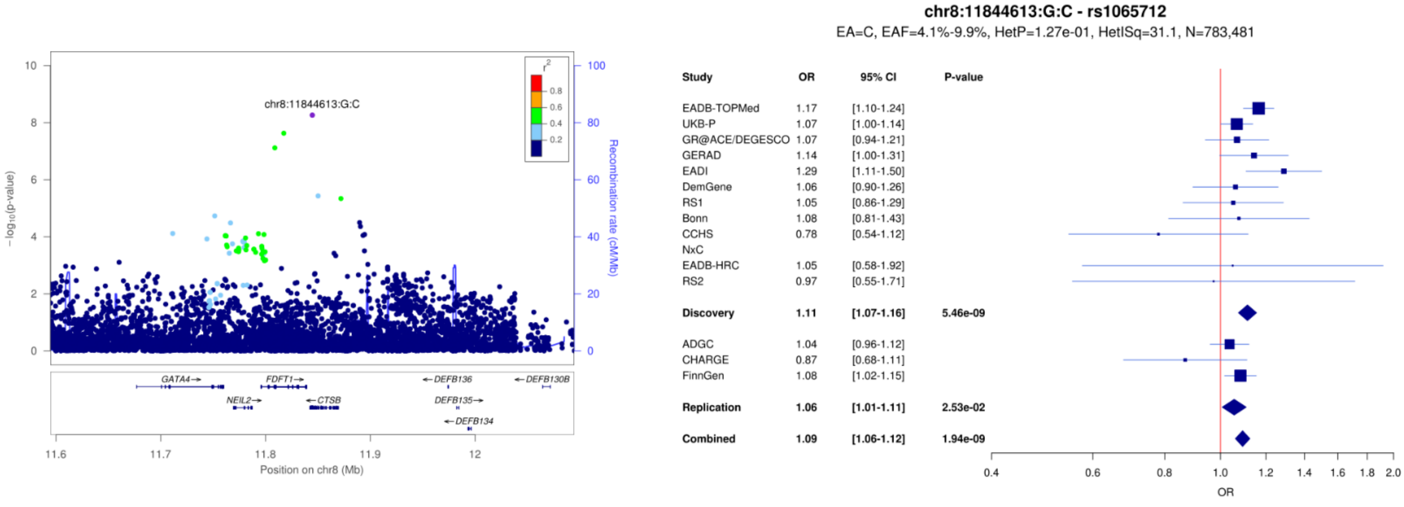

Supplementary Figure 13: LocusZoom and forest plots for (a) *PTK2B*, (b) *CLU* and (c) *SHARPIN* loci.

a

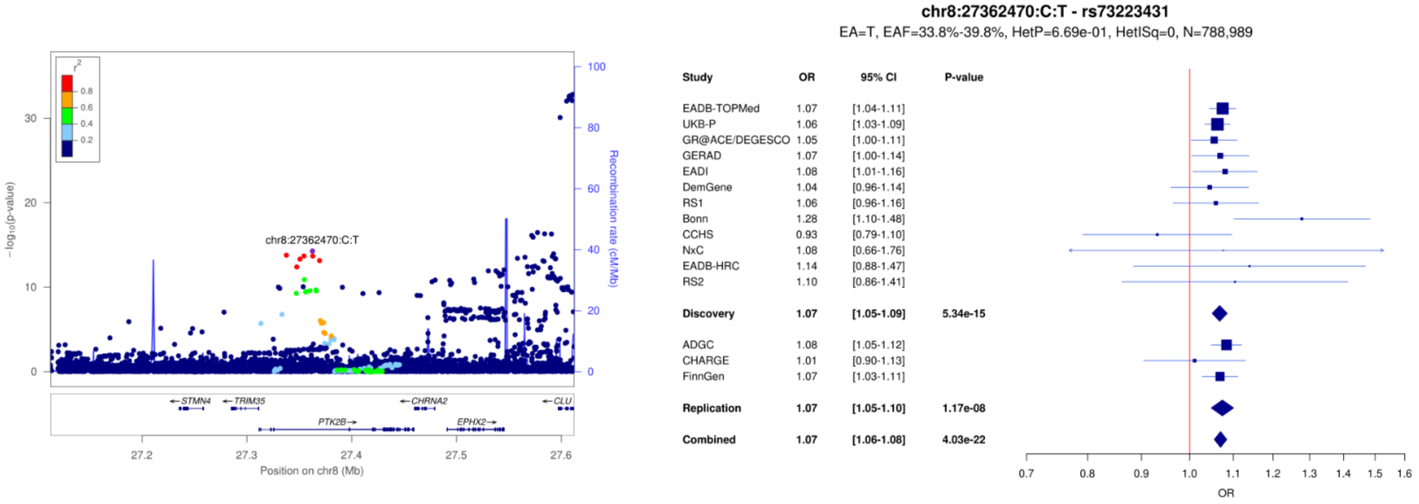

b

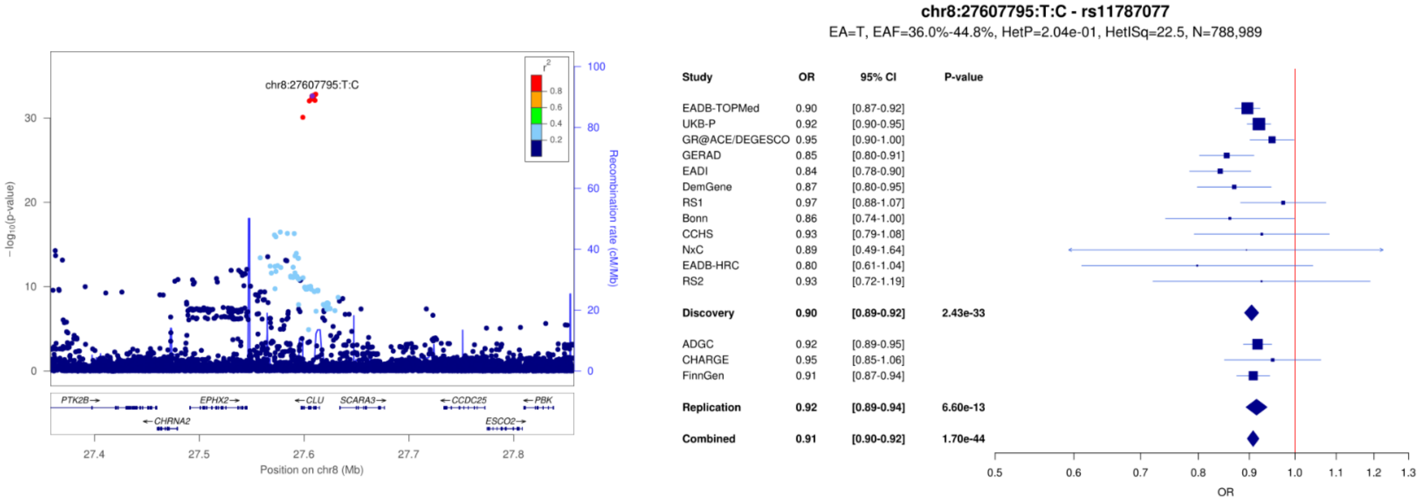

c

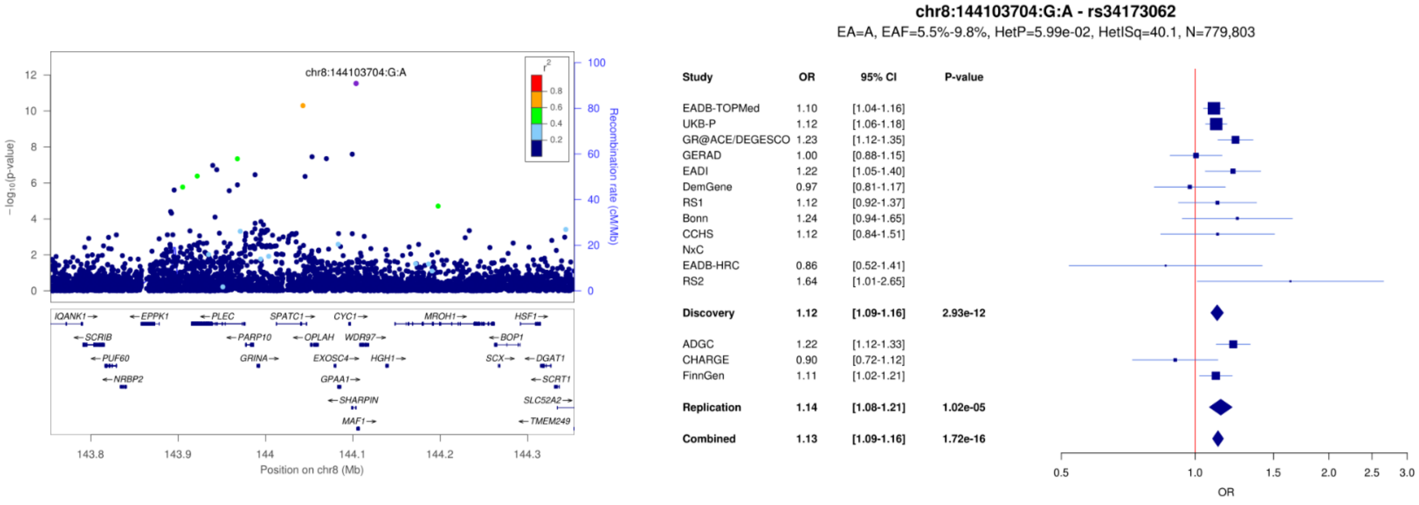

Supplementary Figure 14: LocusZoom and forest plots for (a) *ABCA1*, (b) *USP6NL* and (c) *ANK3* loci.

a

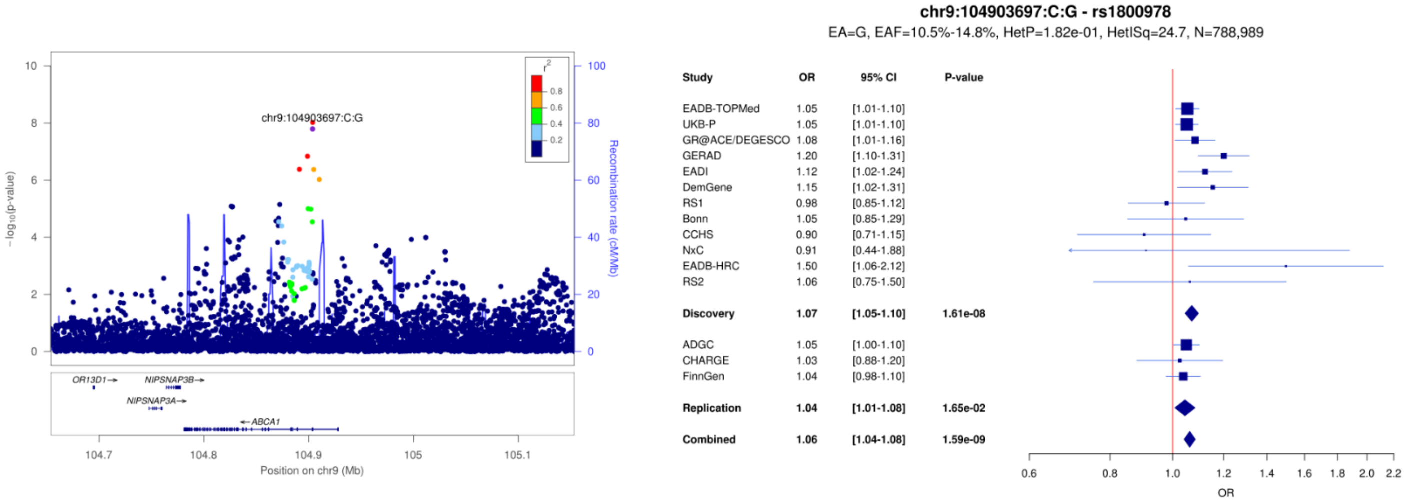

b

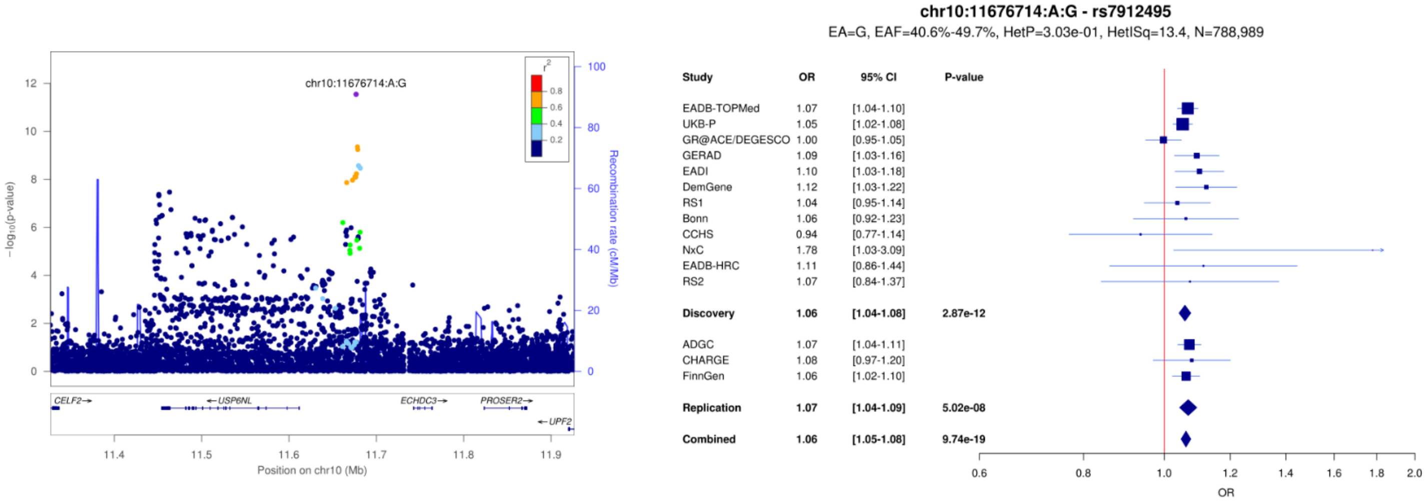

c

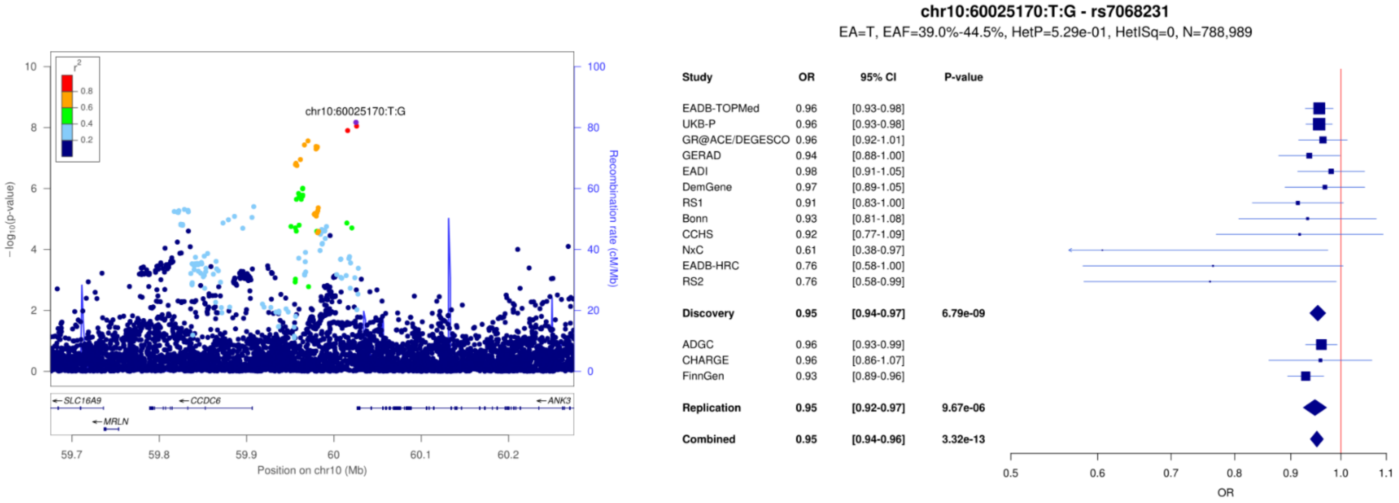

Supplementary Figure 15: LocusZoom and forest plots for (a) *TSPAN14*, (b) *BLNK* and (c) *PLEKHA1* loci.

a

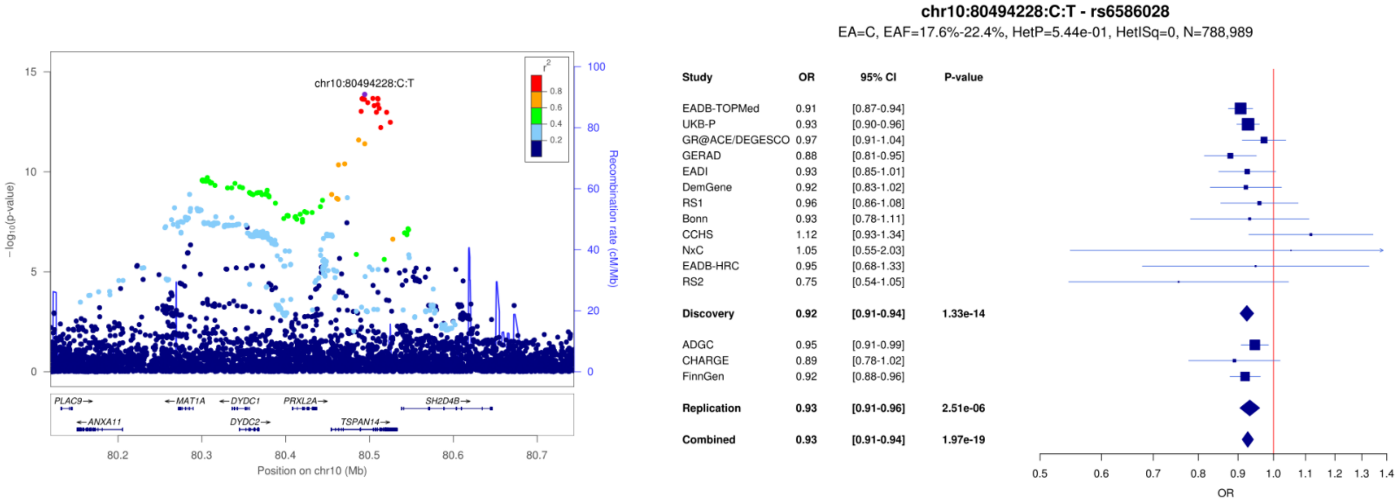

b

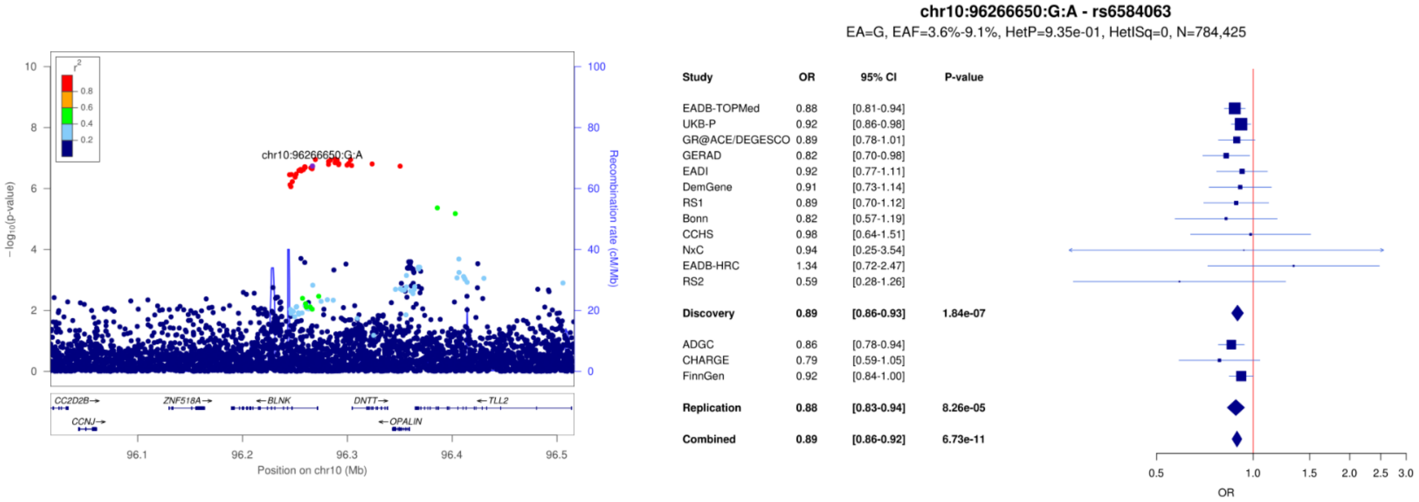

c

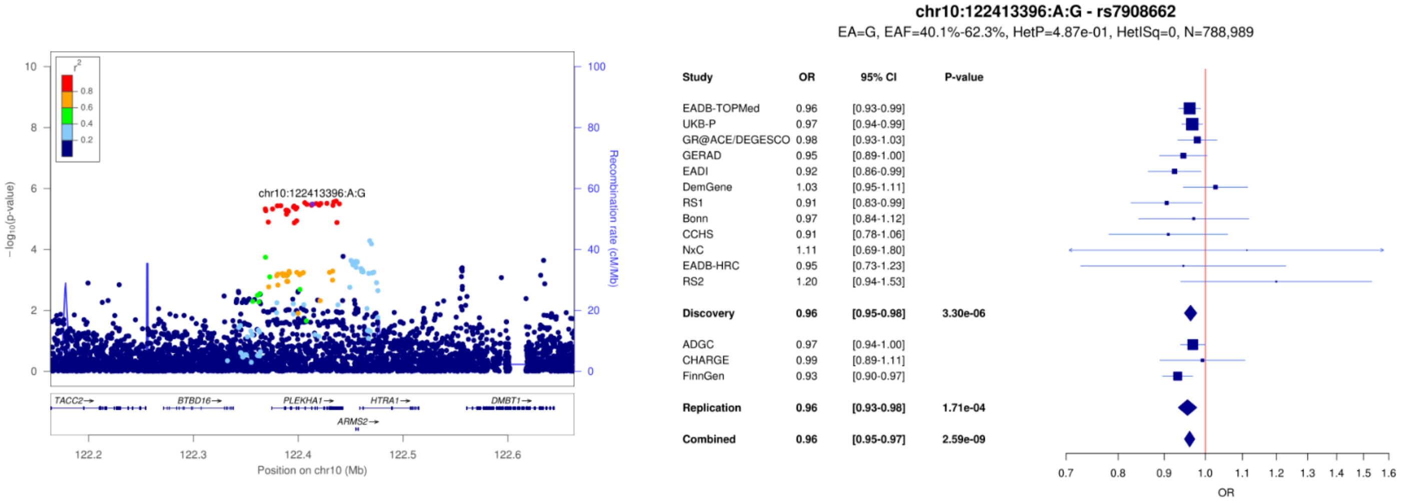

Supplementary Figure 16: LocusZoom and forest plots for (a) *CELF1/SPI1*, (b) *MS4A* and (c) *PICALM* loci.

a

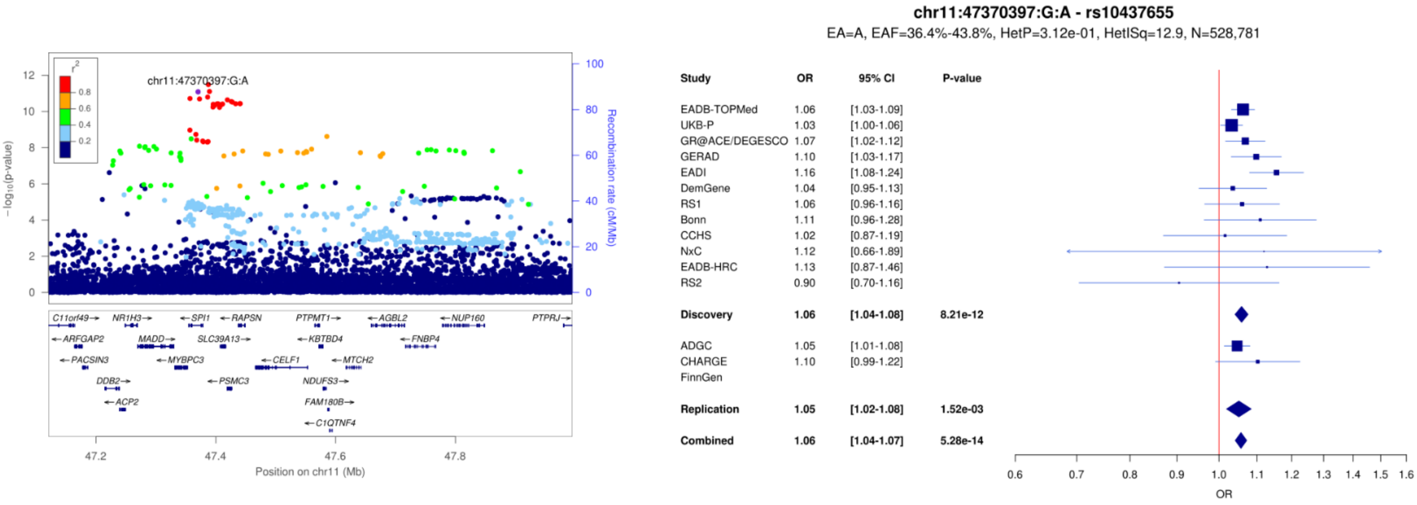

b

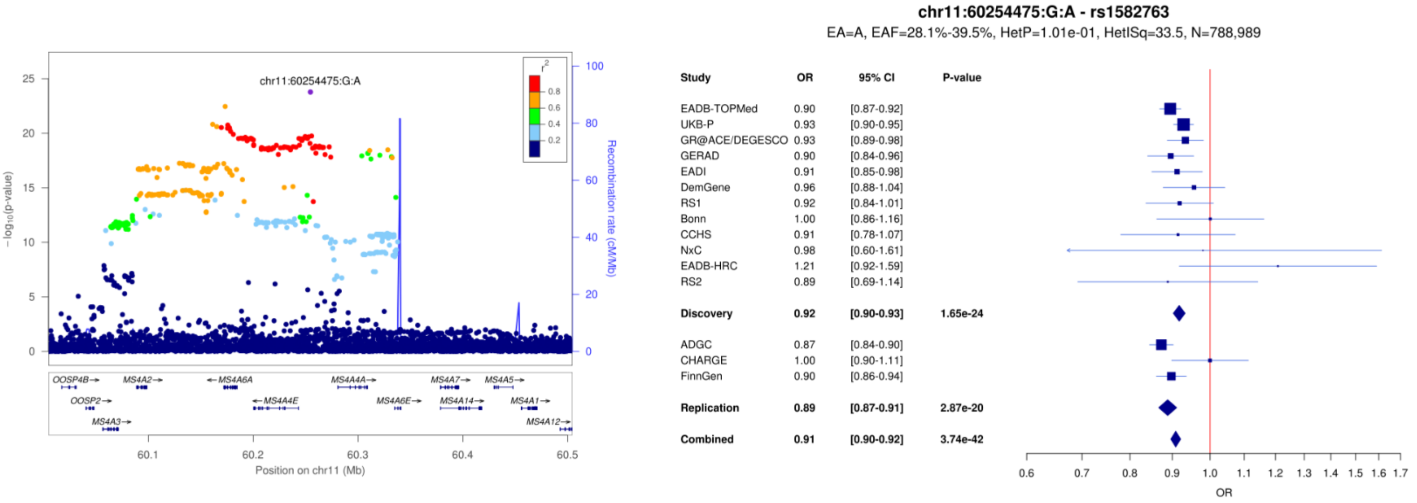

c

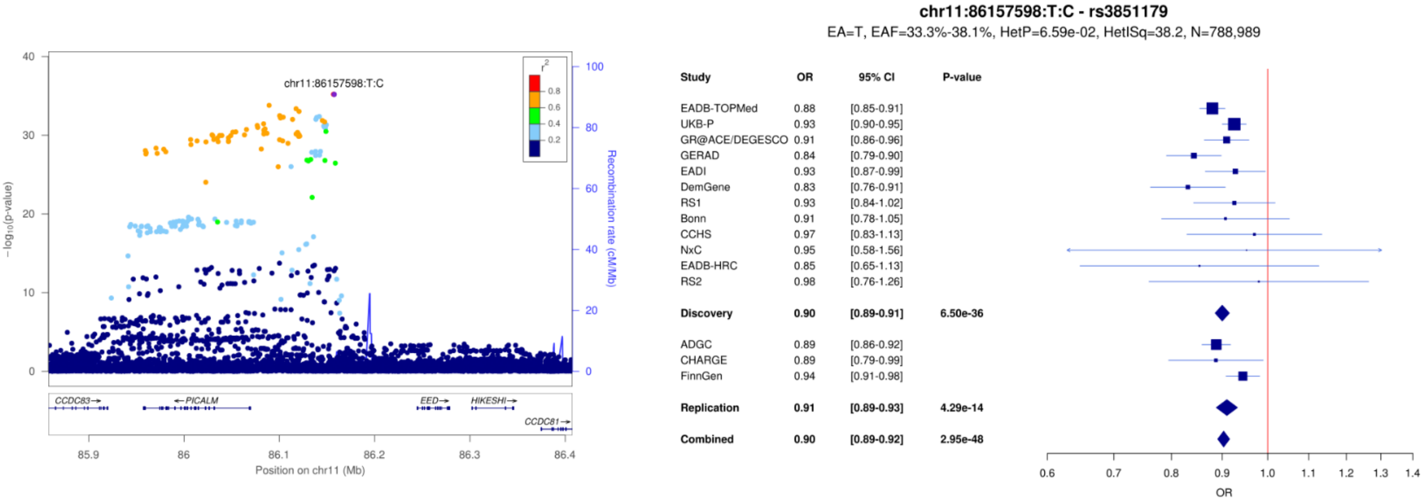

Supplementary Figure 17: LocusZoom and forest plots for (a) *SORL1* (1), (b) *SORL1* (2) and (c) *TPCN1* loci.

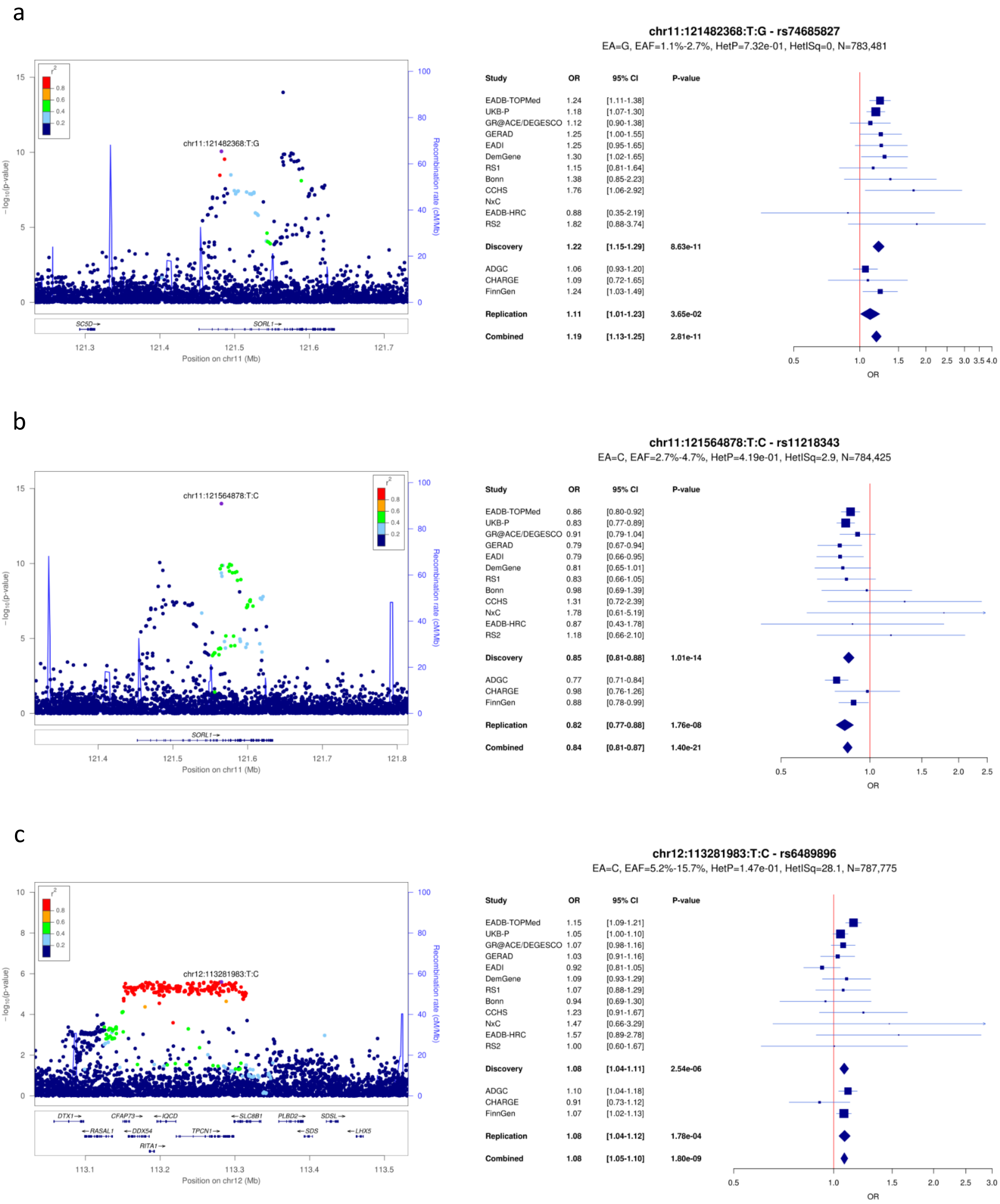

Supplementary Figure 18: LocusZoom and forest plots for (a) *FERMT2*, (b) *SLC24A4/RIN3* (1) and (c) *SLC24A4/RIN3* (2) loci.

a

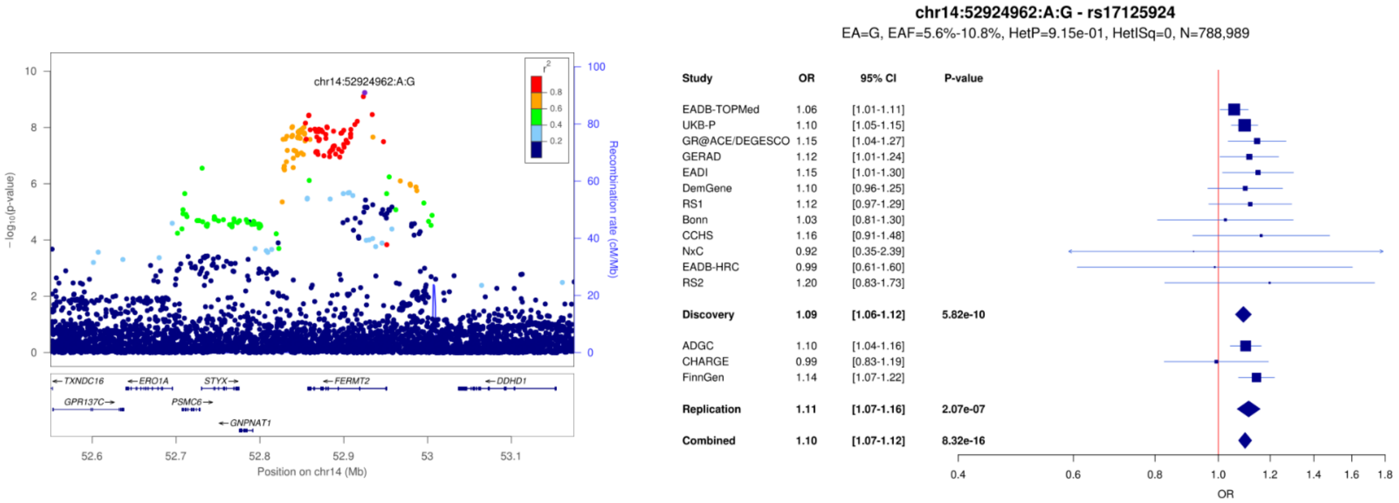

b

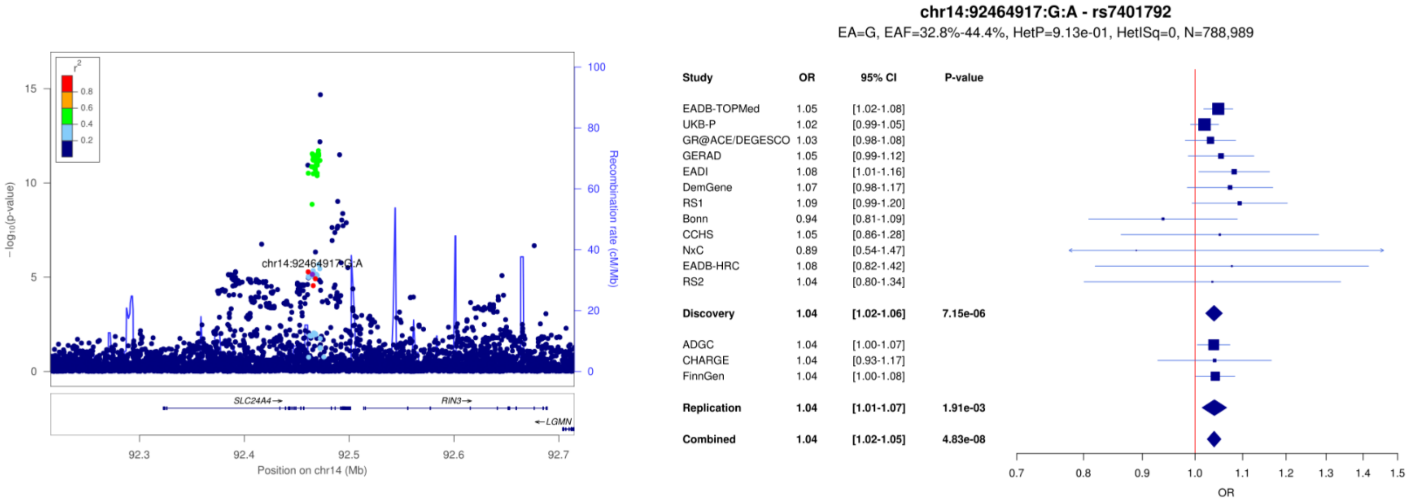

c

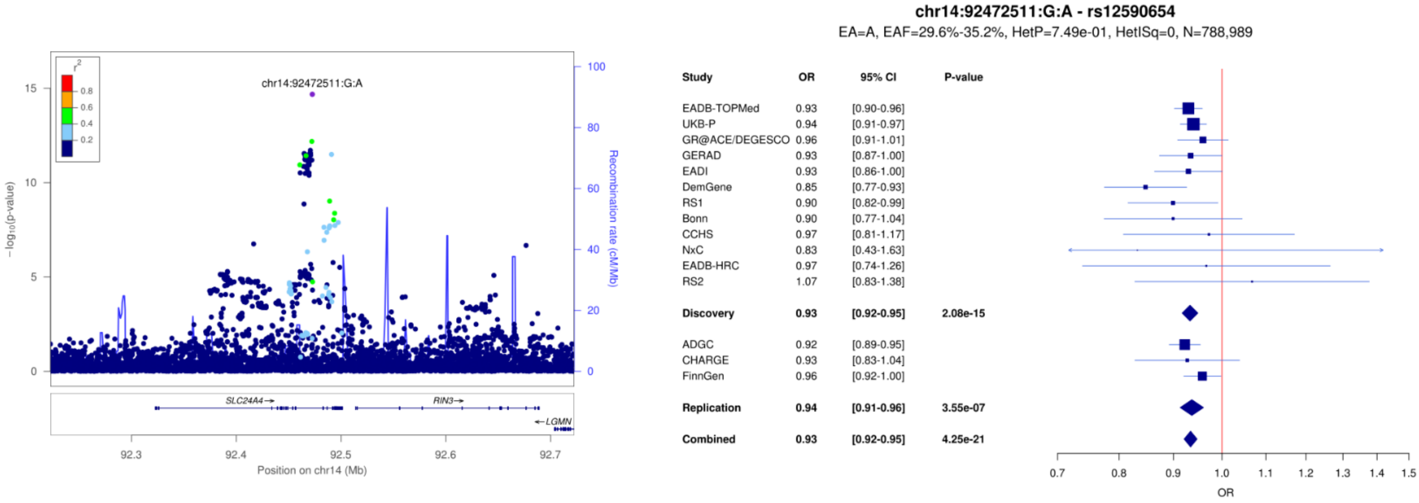

Supplementary Figure 19: LocusZoom and forest plots for (a) *IGH* gene cluster (1), (b) *IGH* gene cluster (2) and (c) *SPPL2A* loci.

a

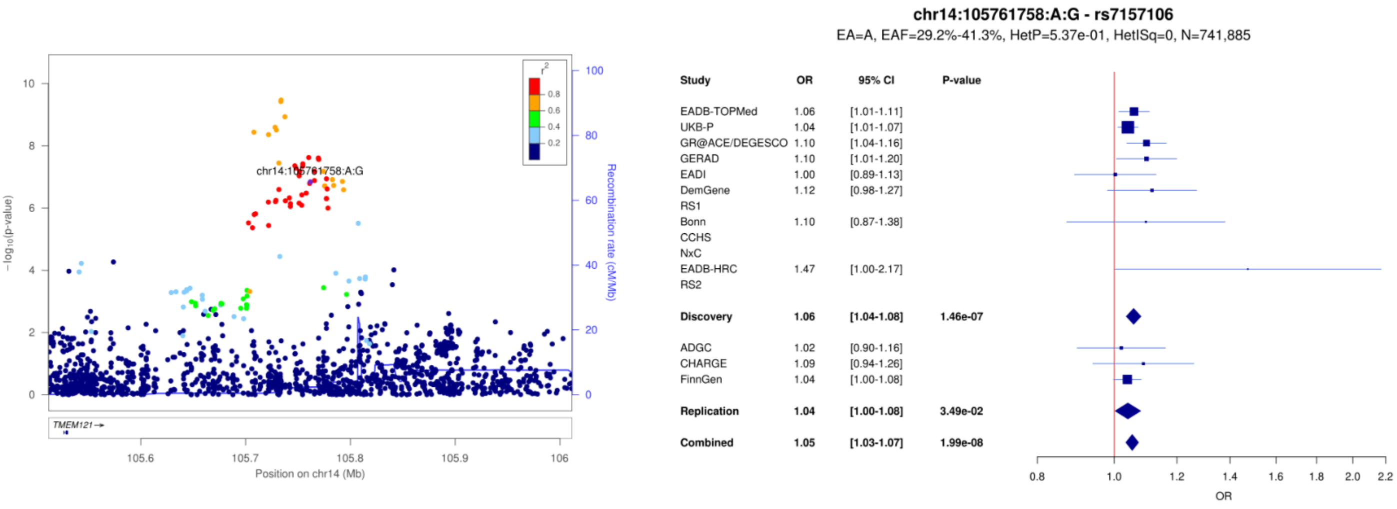

b

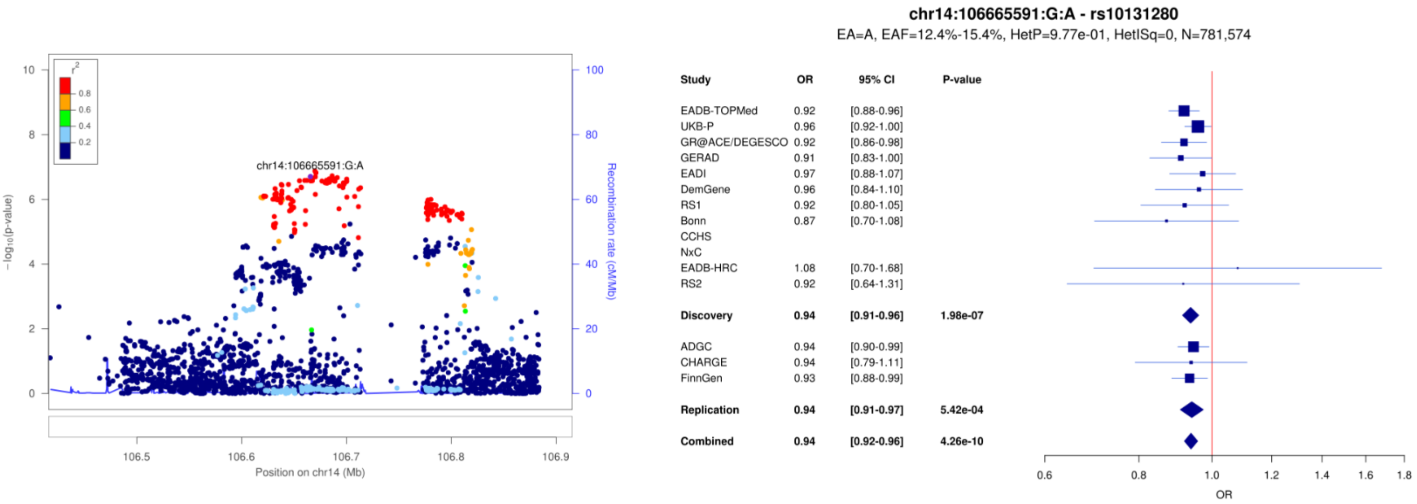

c

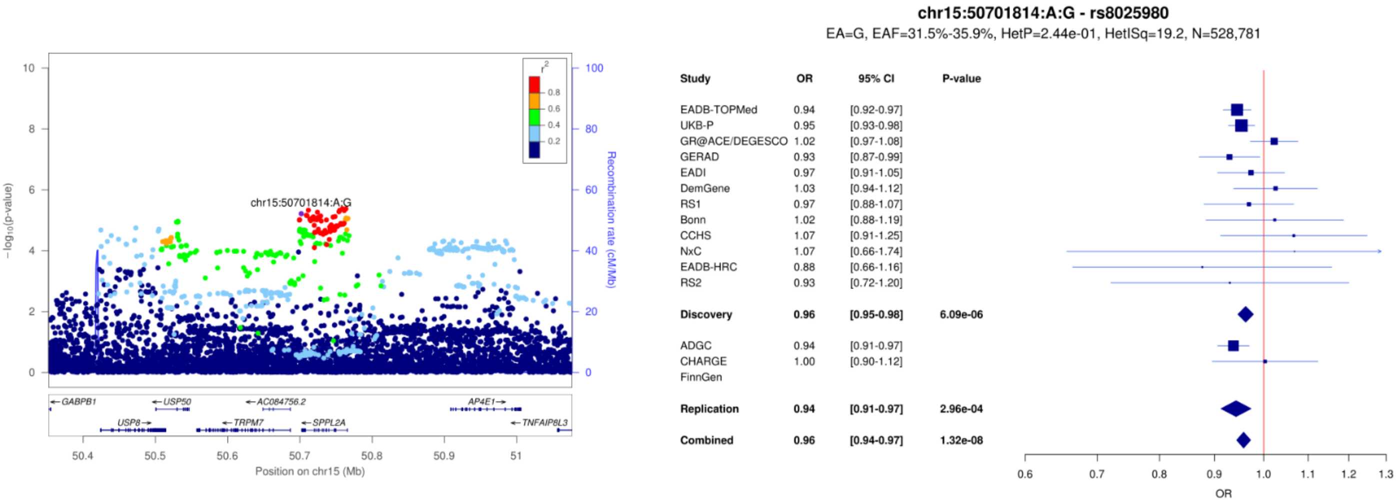

Supplementary Figure 20: LocusZoom and forest plots for (a) *ADAM10*, (b) *APH1B* and (c) *SNX1* loci.

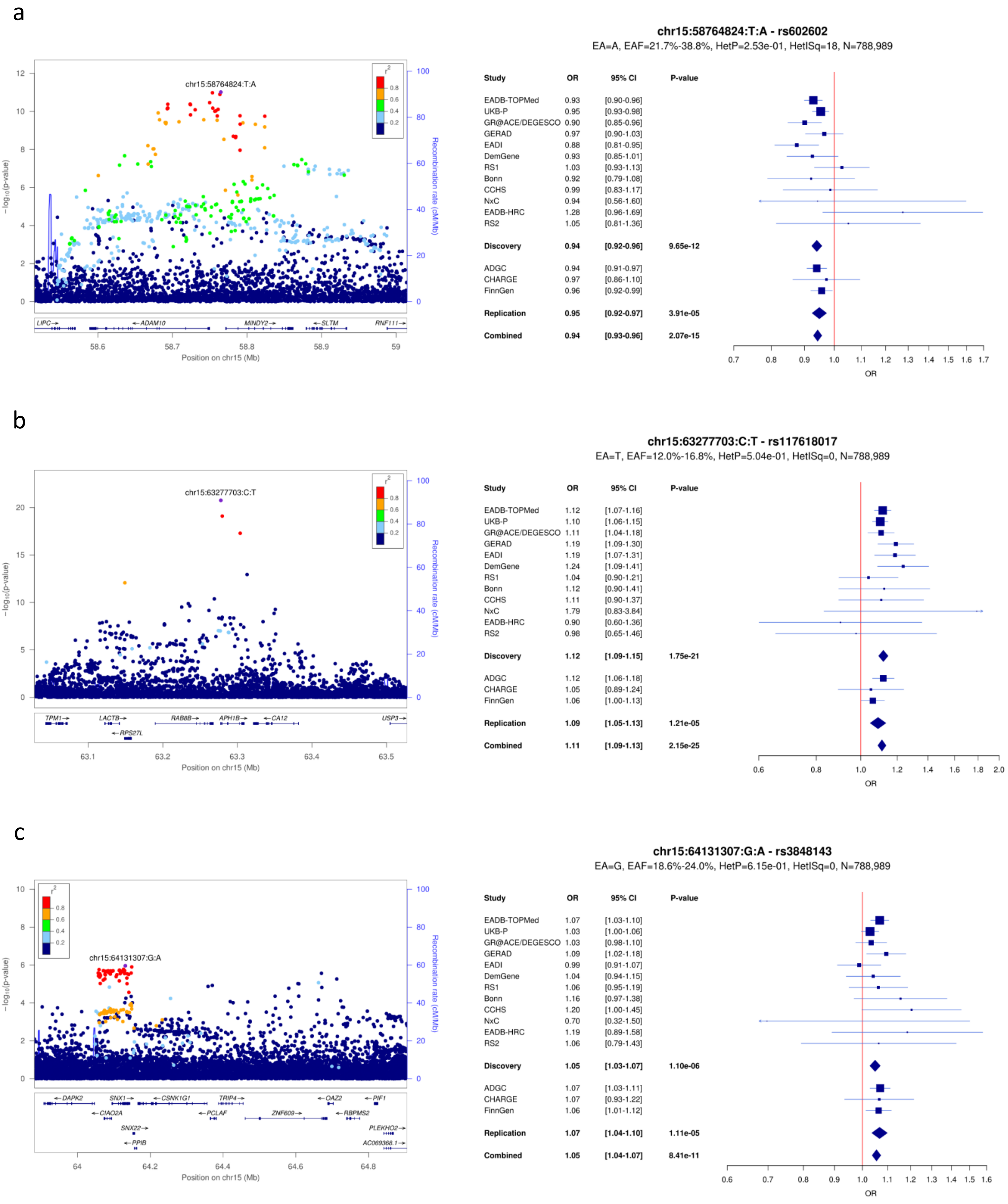

Supplementary Figure 21: LocusZoom and forest plots for (a) *CTSH*, (b) *DOC2A* and (c) *KAT8* loci.

a

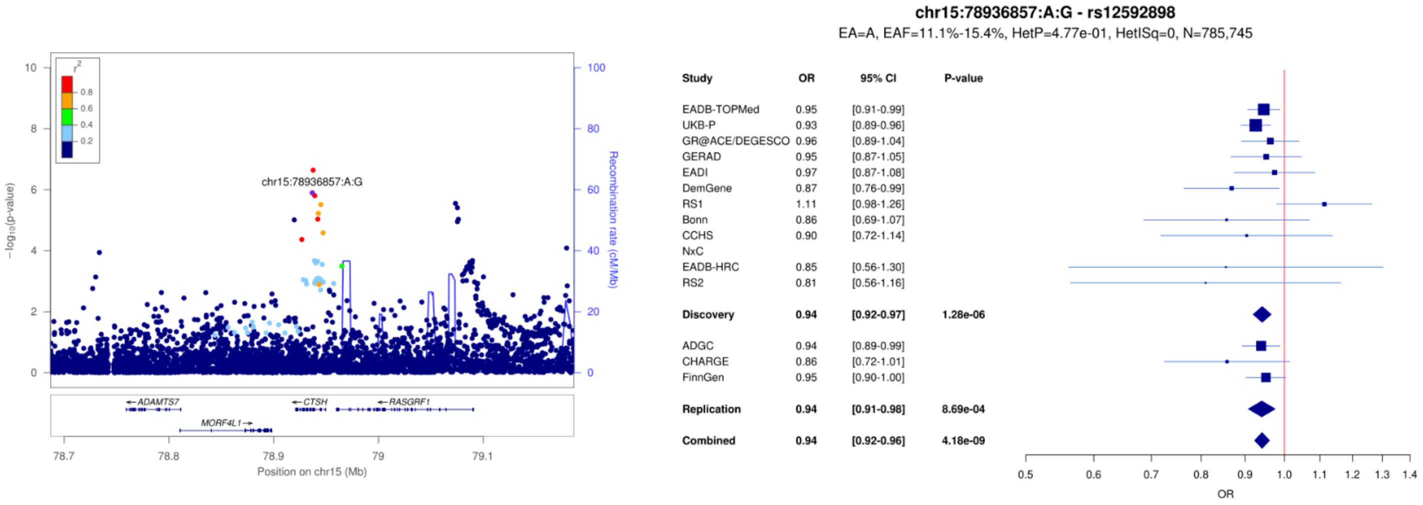

b

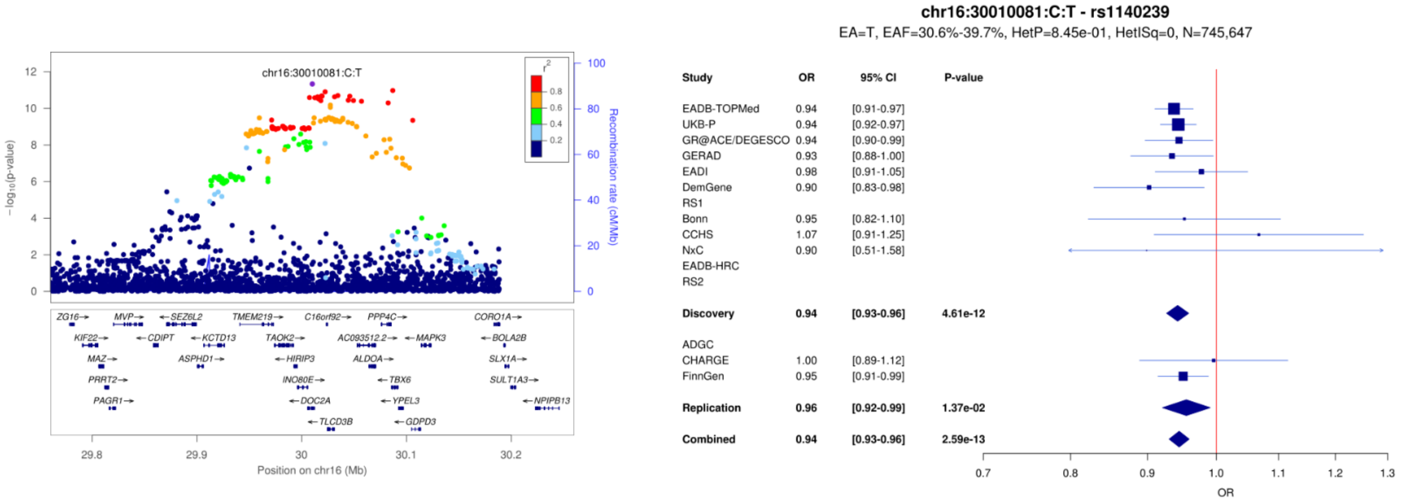

c

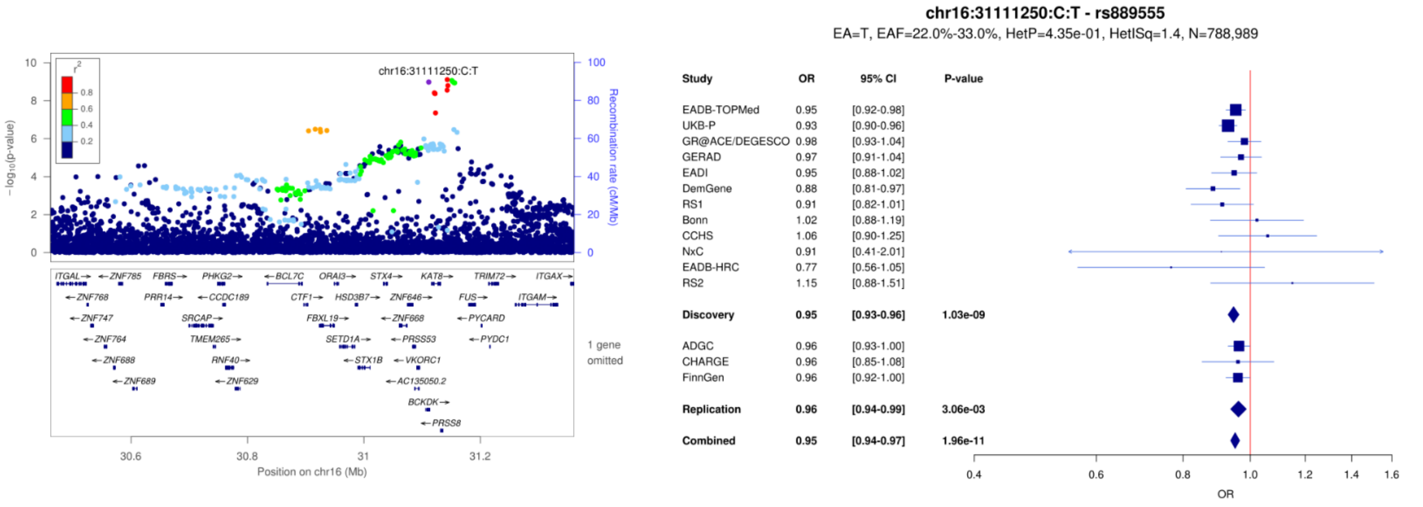

Supplementary Figure 22: LocusZoom and forest plots for (a) *IL34*, (b) *MAF* and (c) *PLCγ2* (1) loci.

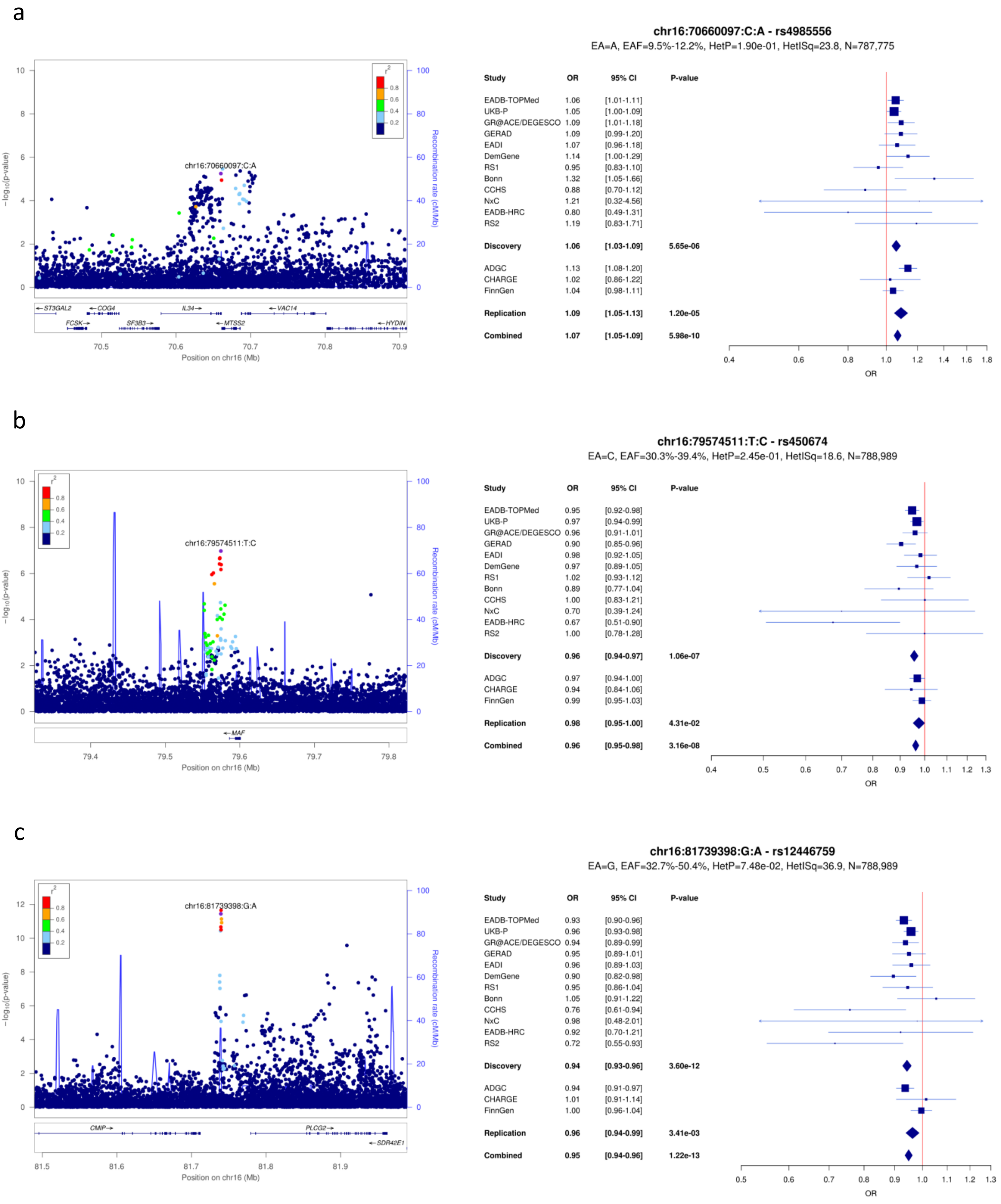

Supplementary Figure 23: LocusZoom and forest plots for (a) *PLCγ2* (2), (b) *FOXF1* and (c) *PRDM7* loci.

a

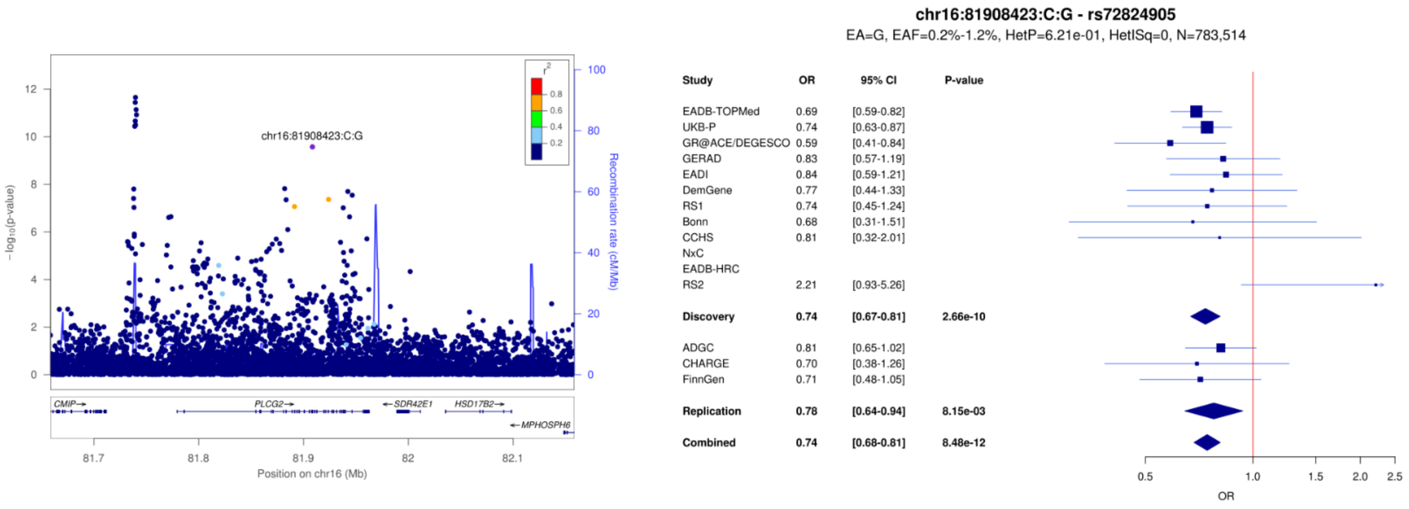

b

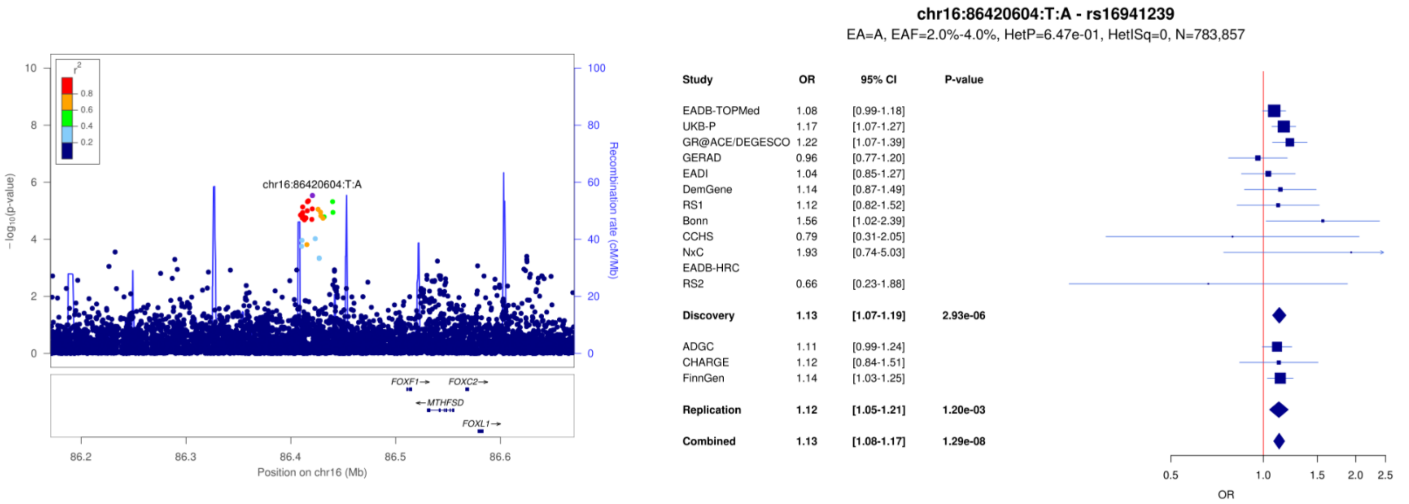

c

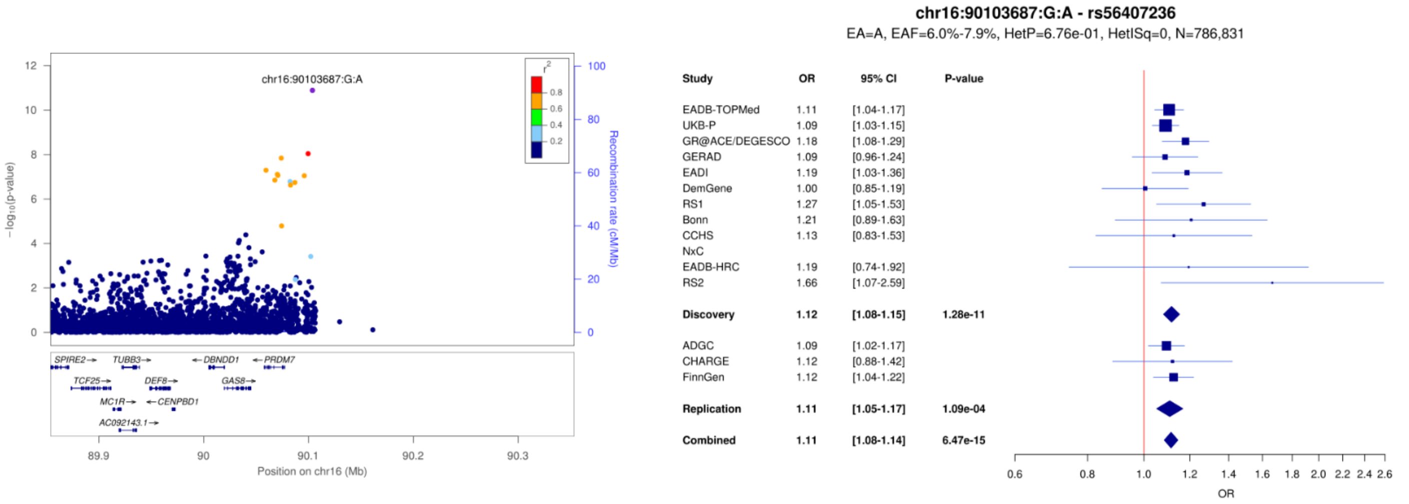

Supplementary Figure 24: LocusZoom and forest plots for (a) *WDR81*, (b) *SCIMP/RABEP1* and (c) *MYO15A* loci.

a

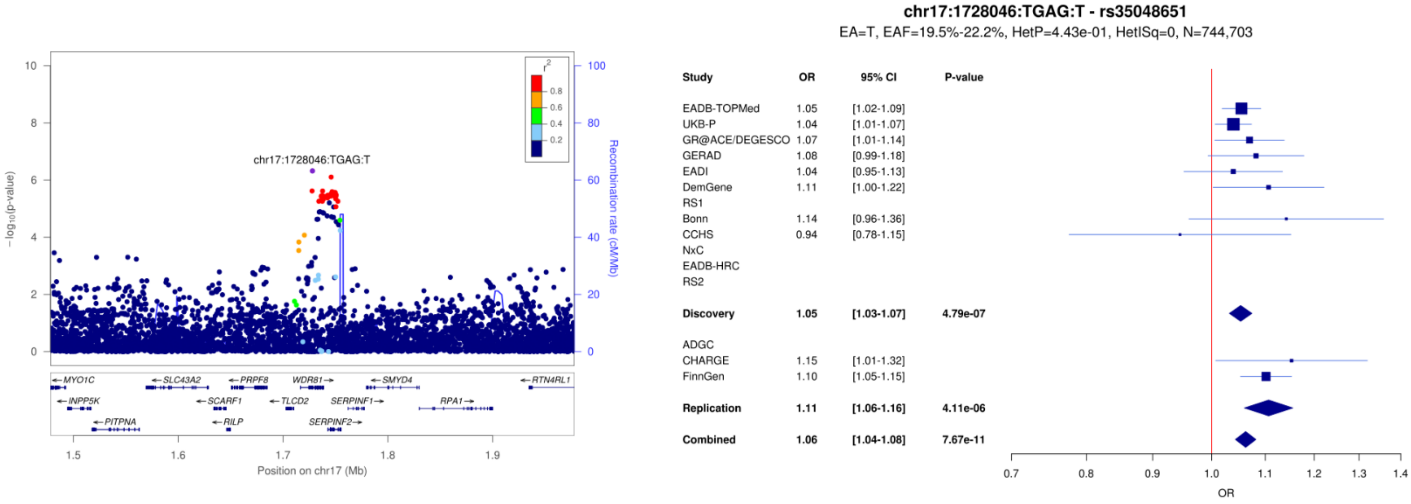

b

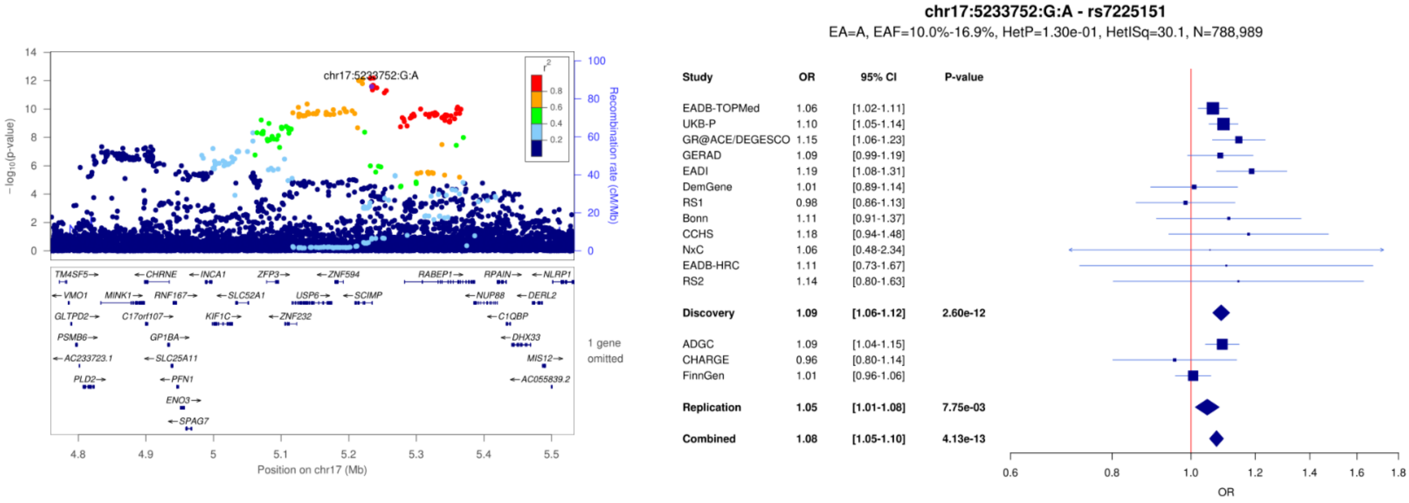

c

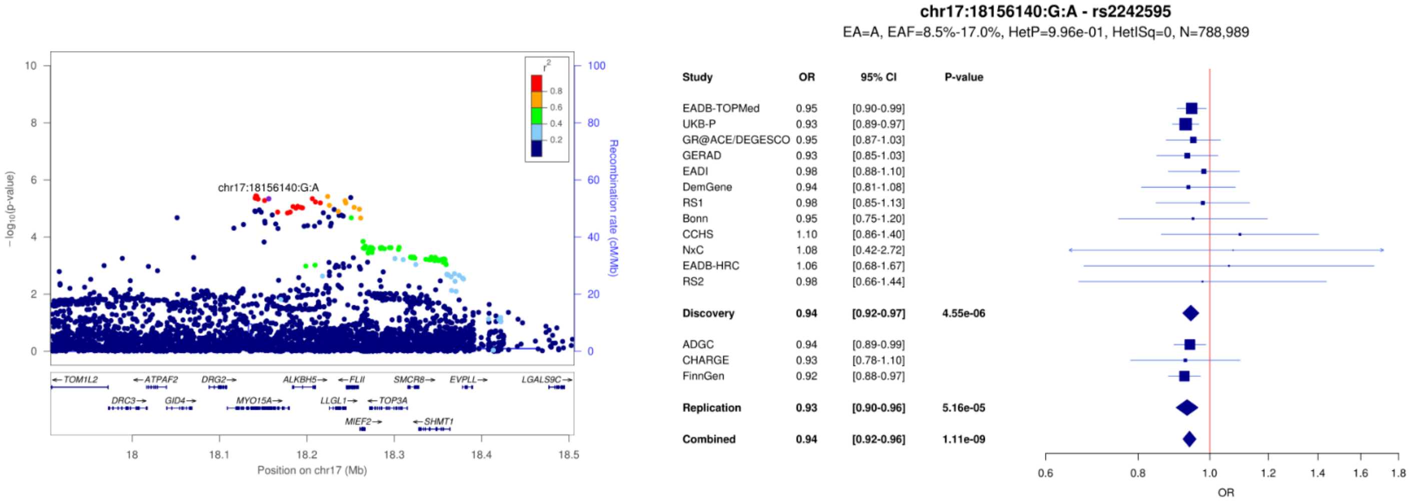

Supplementary Figure 25: LocusZoom and forest plots for (a) *GRN*, (b) *MAPT* and (c) *ABI3* loci.

a

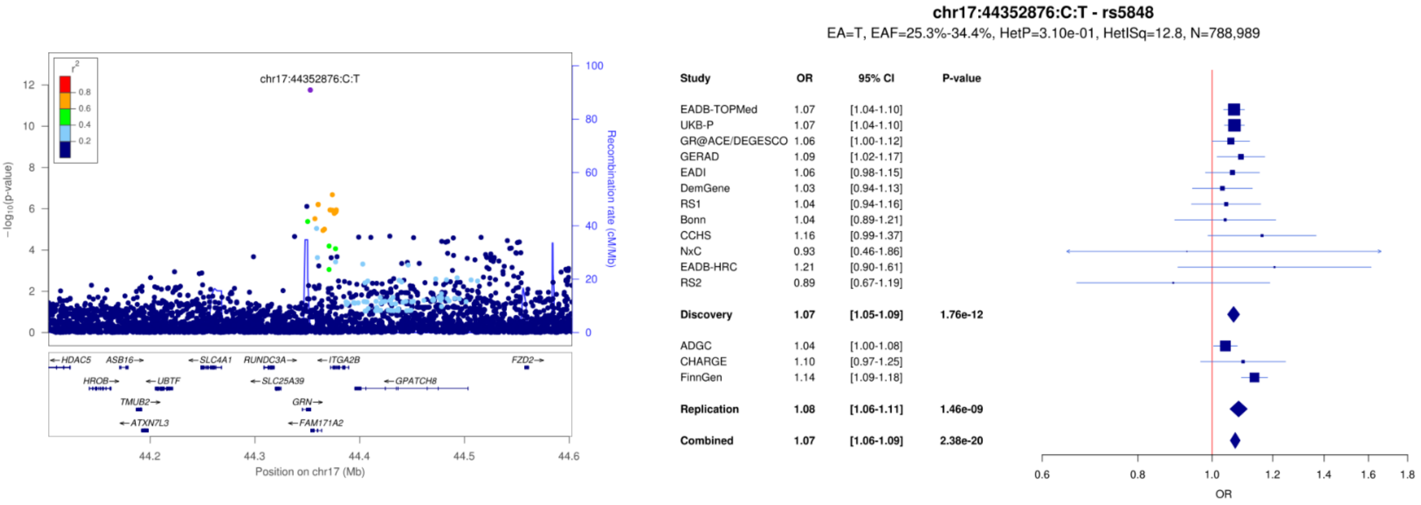

b

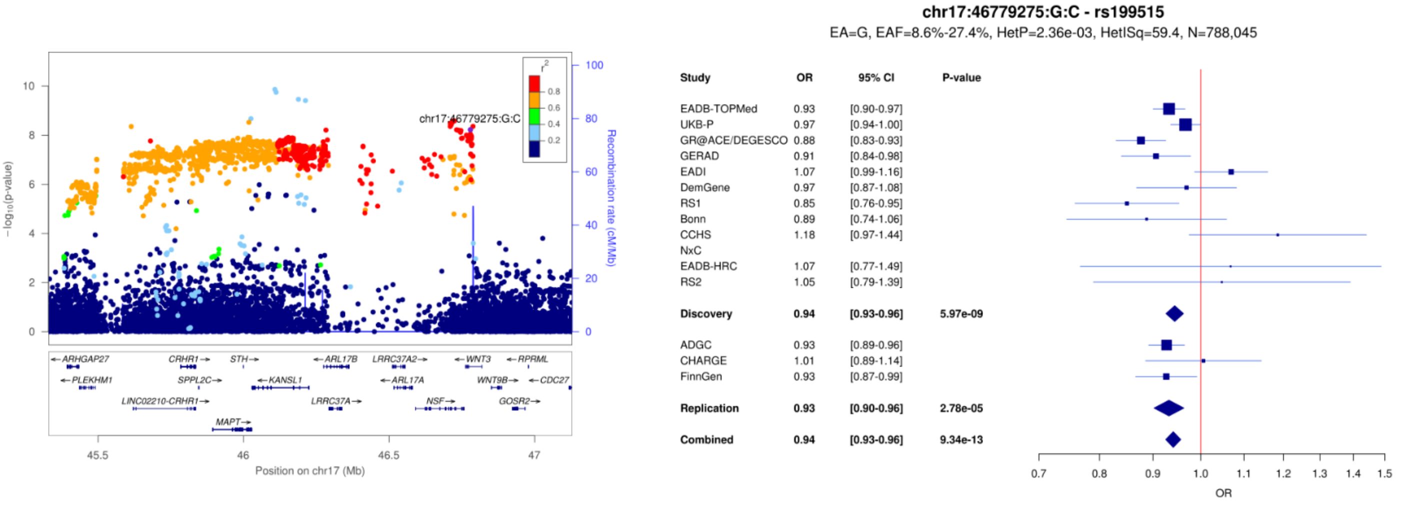

c

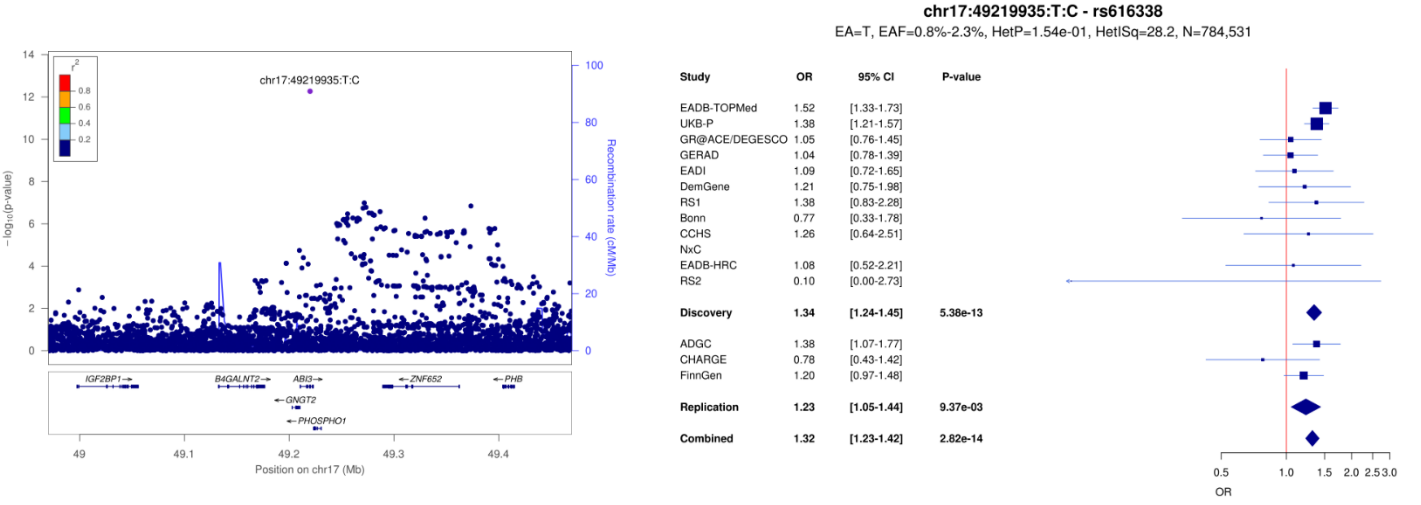

Supplementary Figure 26: LocusZoom and forest plots for (a) *TSPOAP1*, (b) *ACE* and (c) *ABCA7* loci.

a

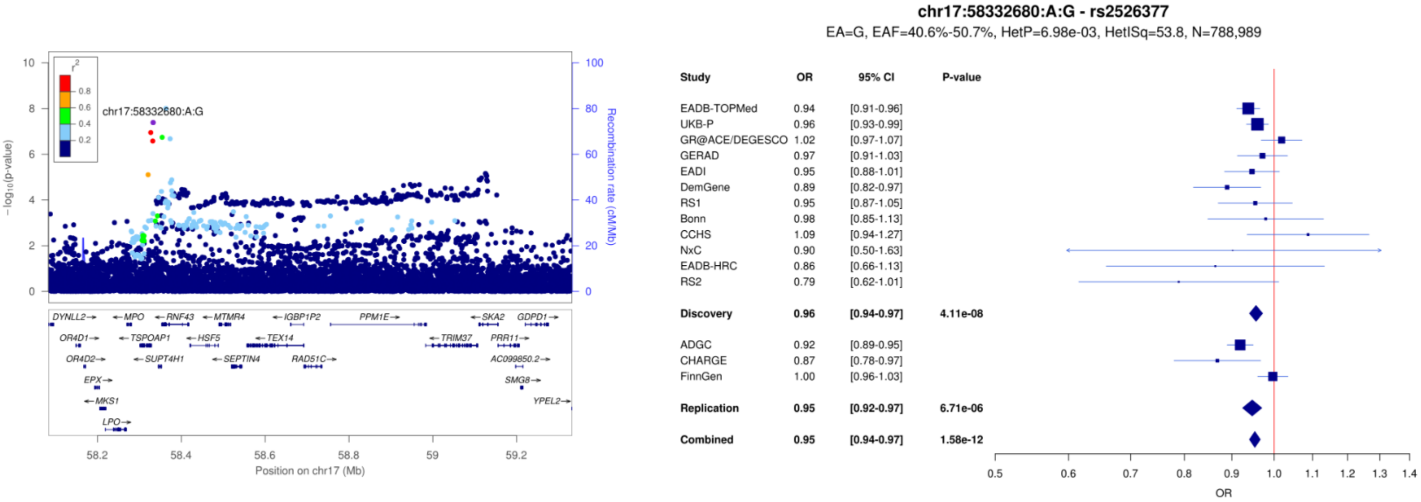

b

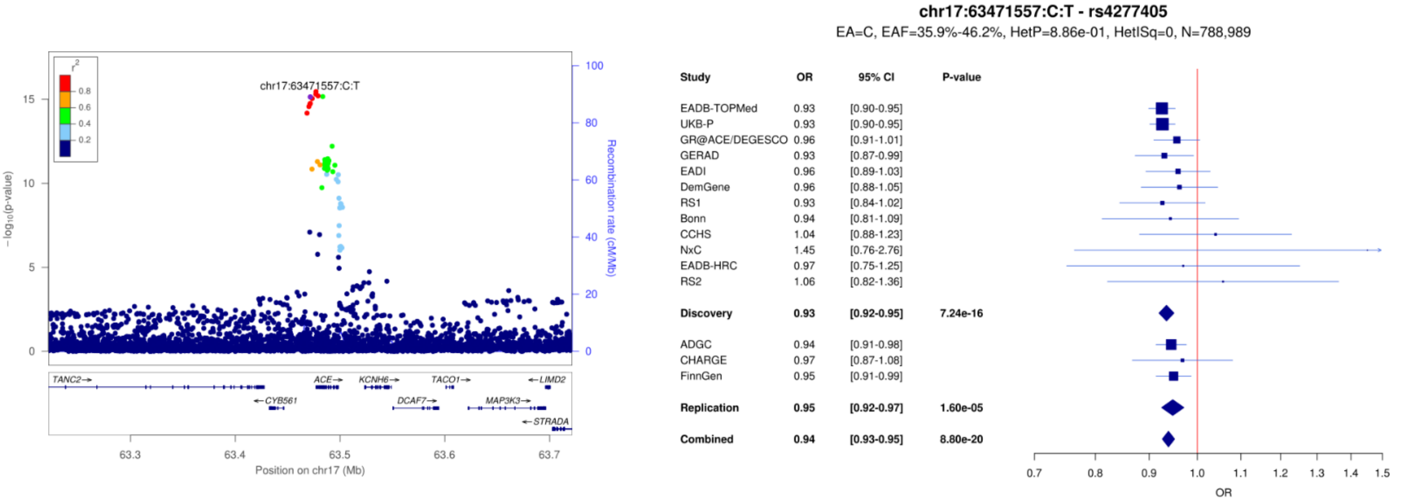

c

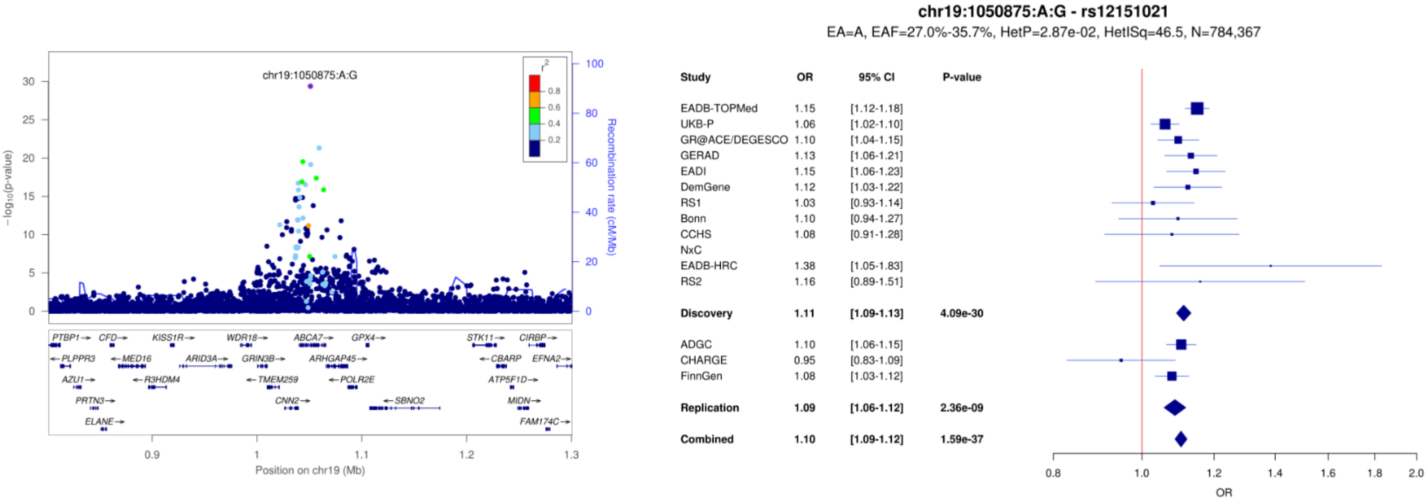

Supplementary Figure 27: LocusZoom and forest plots for (a) *KLF16*, (b) *SIGLEC11* and (c) *LILRB2* loci.

a

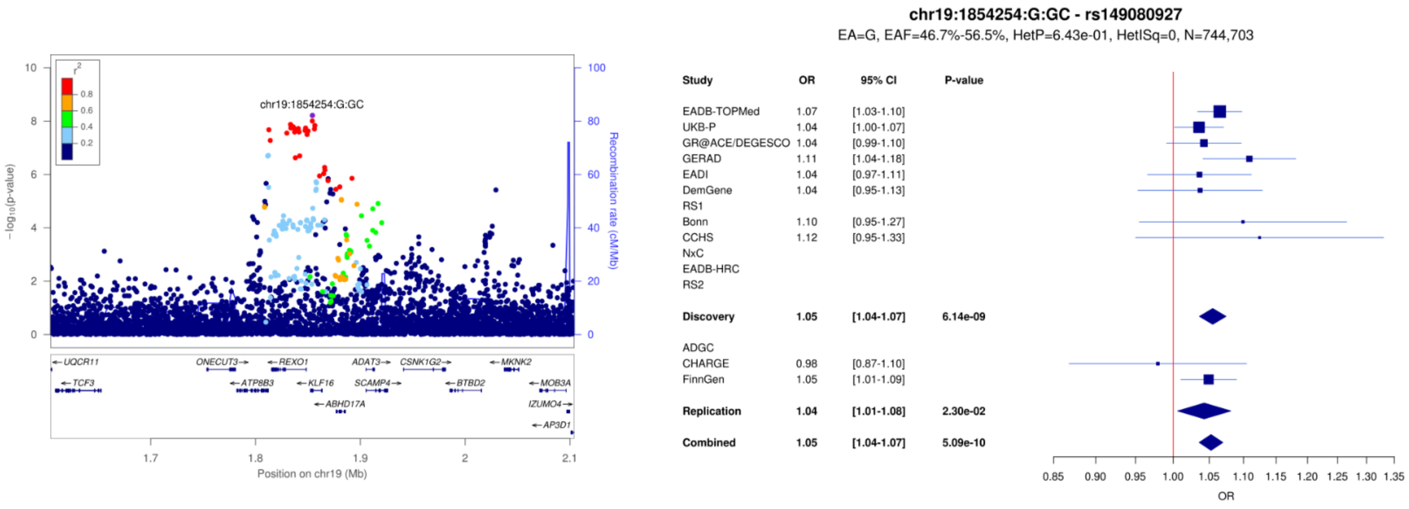

b

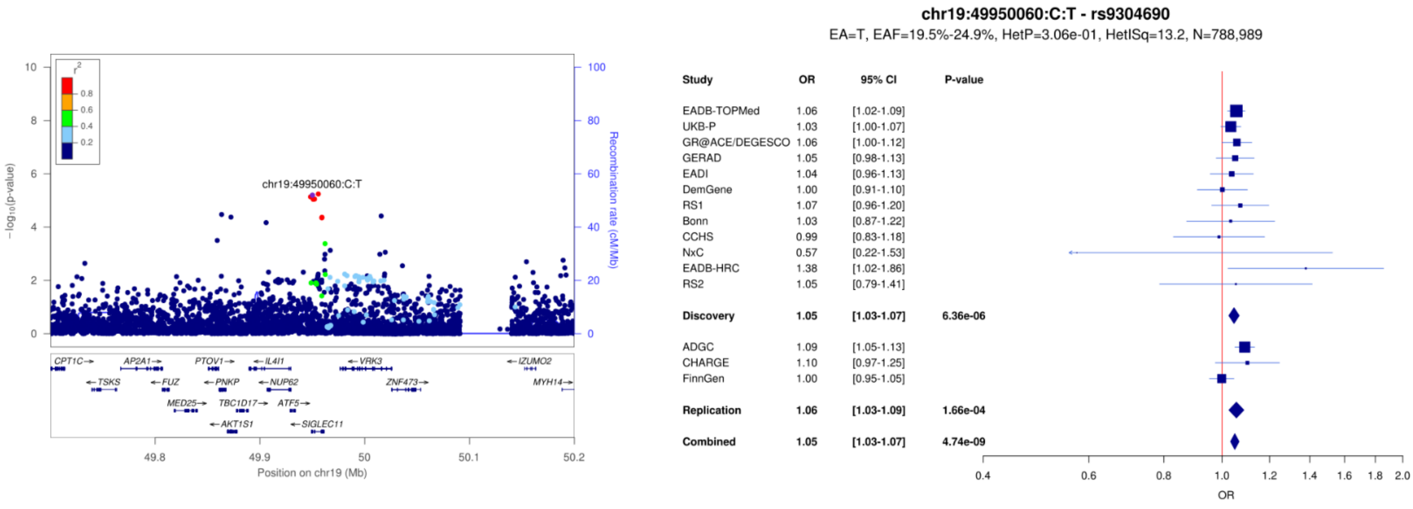

c

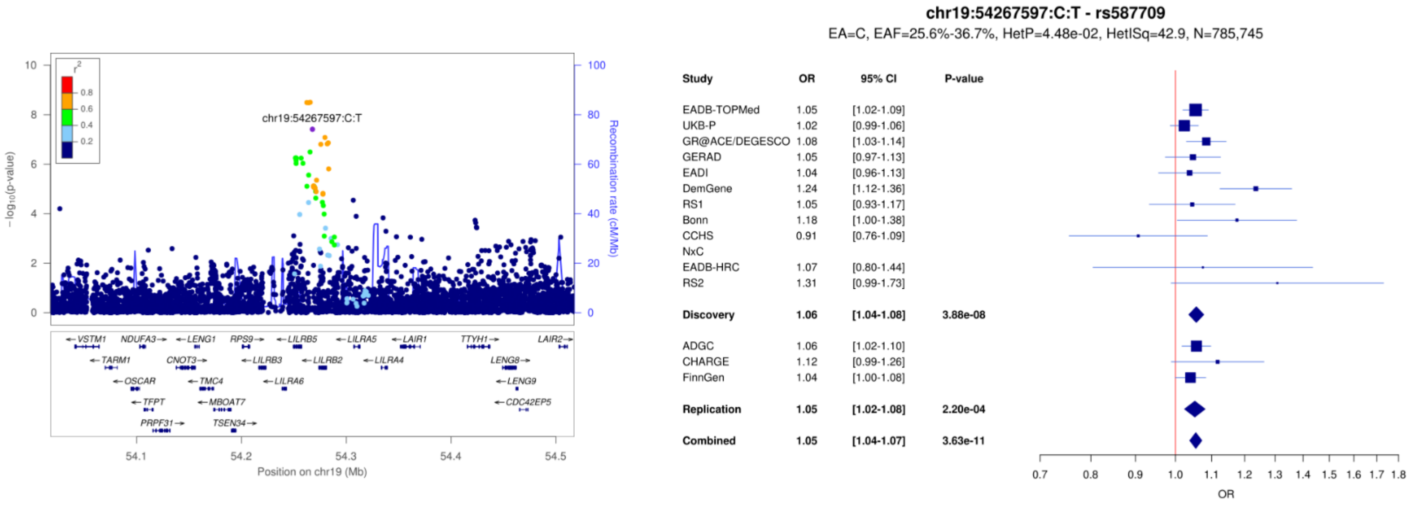

Supplementary Figure 28: LocusZoom and forest plots for (a) *RBCK1*, (b) *CASS4* and (c) *SLC2A4RG* loci.

a

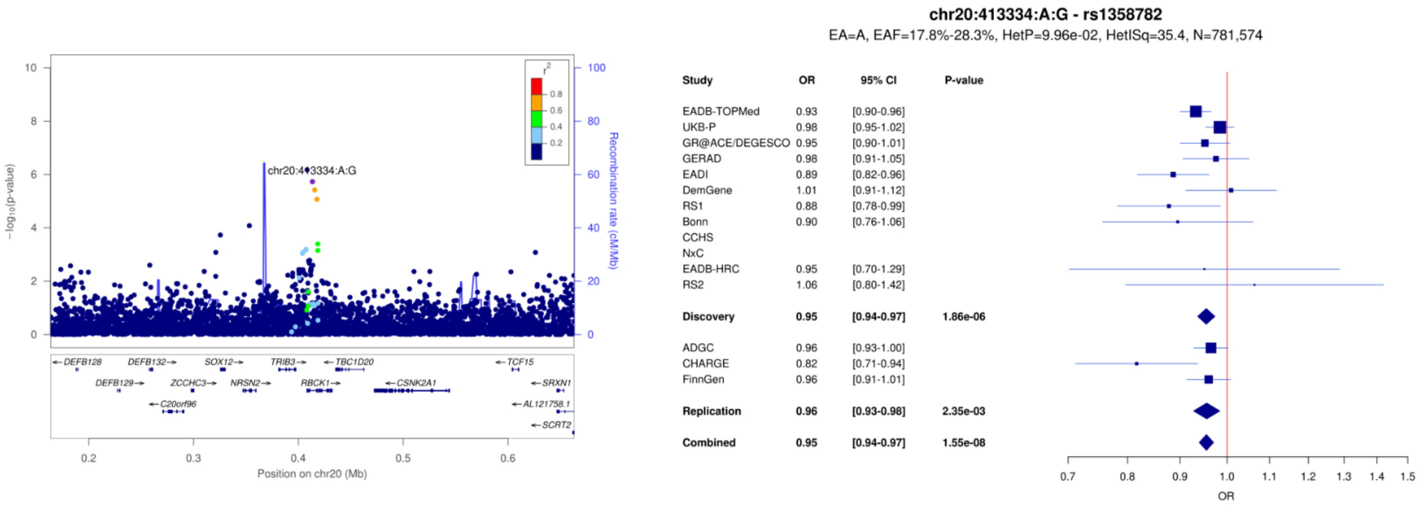

b

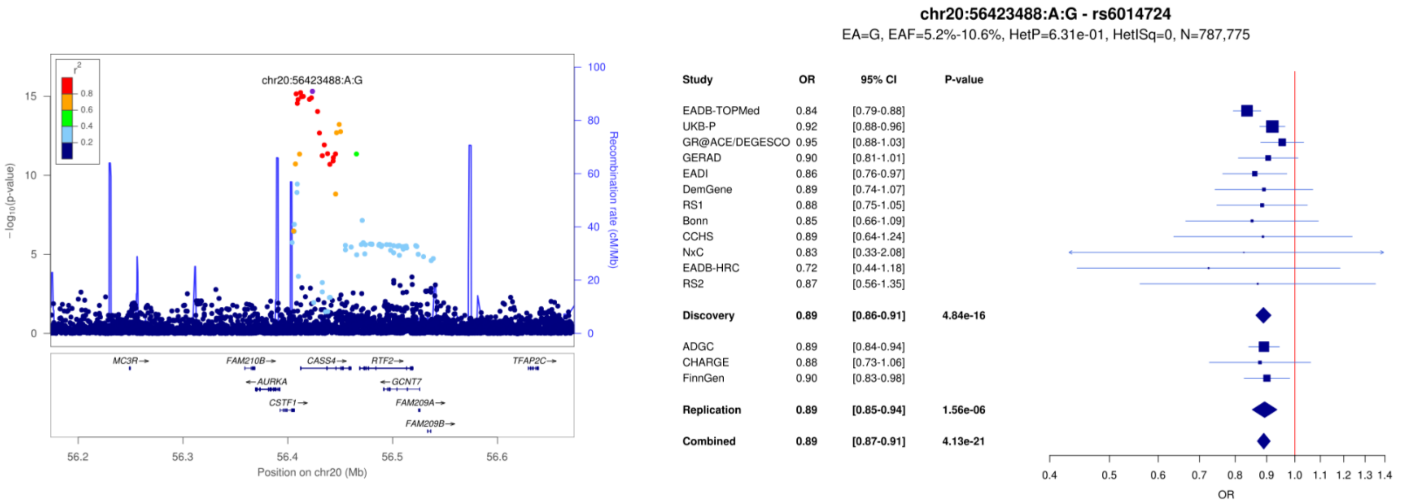

c

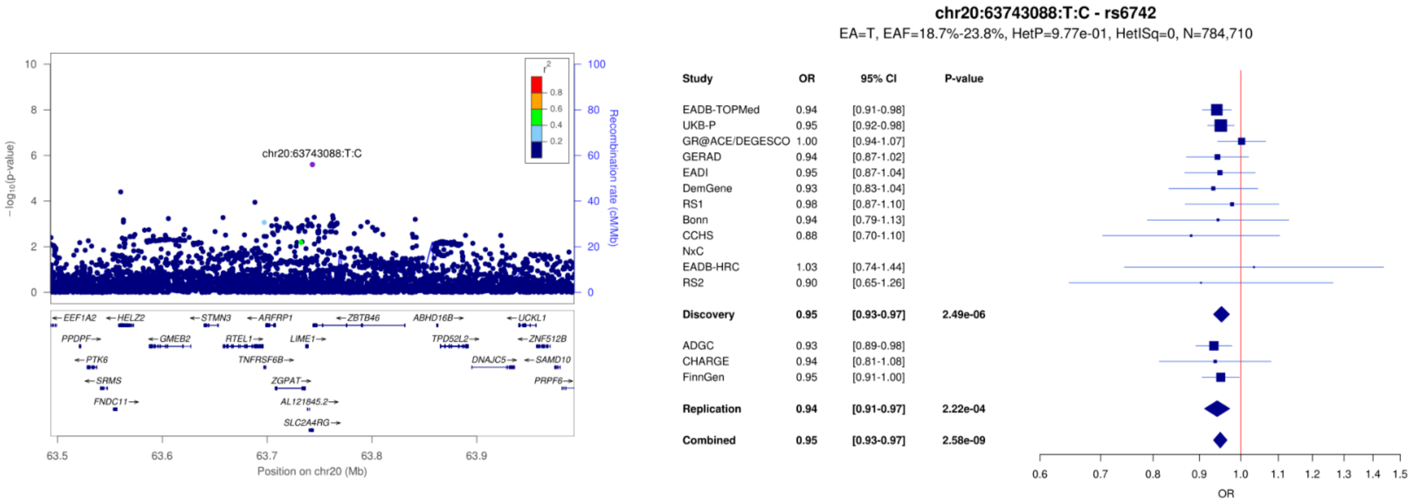

Supplementary Figure 29: LocusZoom and forest plots for (a) *APP* and (b) *ADAMTS1* loci.

a

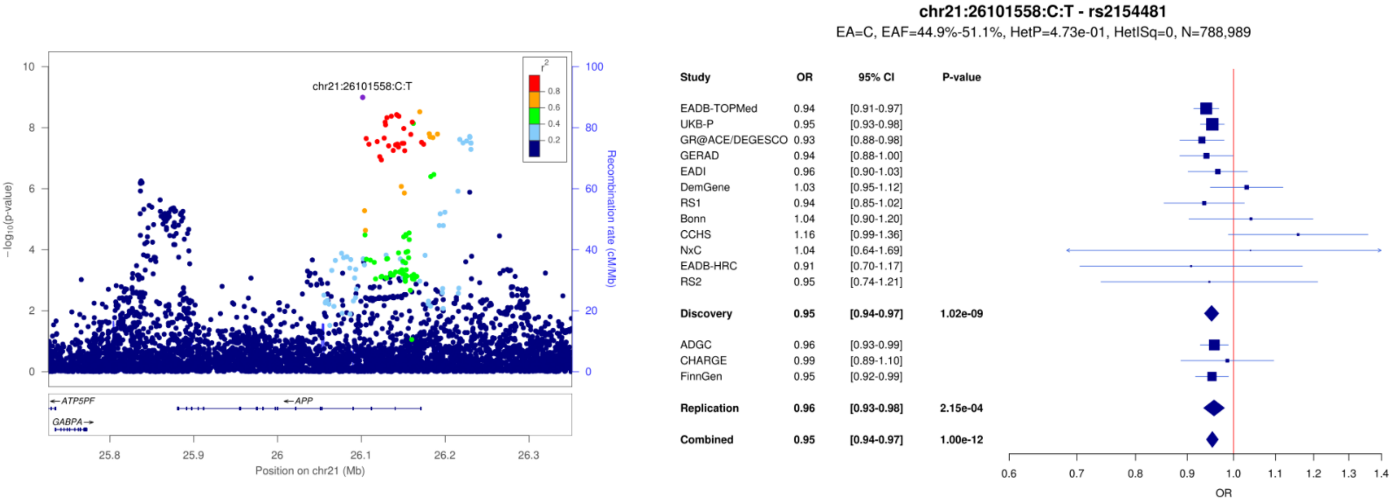

b

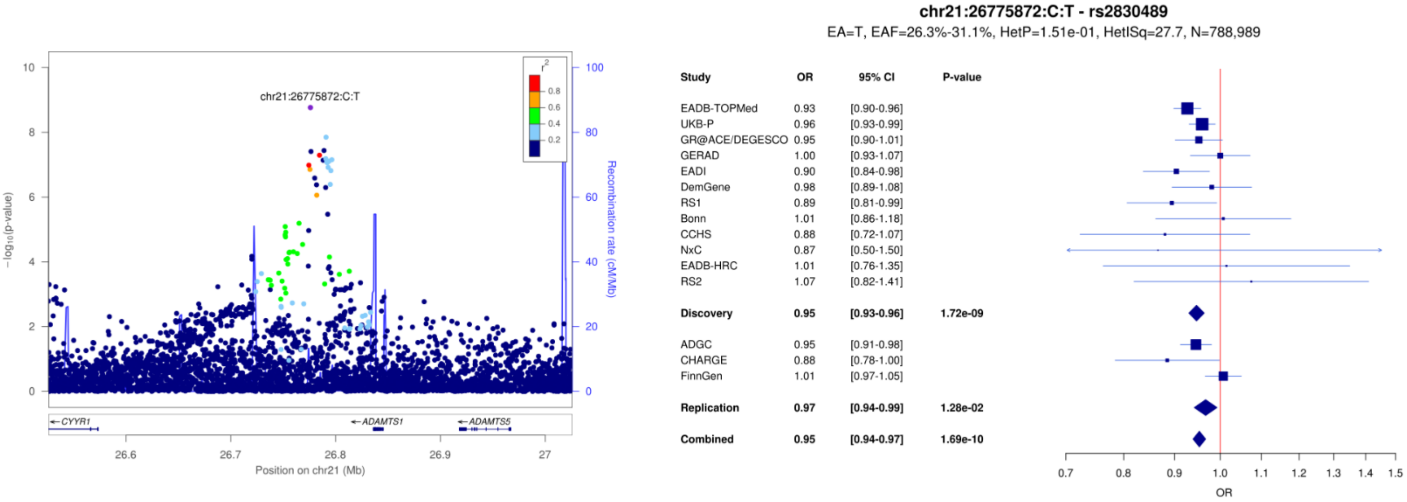

Supplementary Figure 30: Comparison of ORs of the genome-wide significant loci a) observed in the diagnosed cases only analysis and estimated in the UK-Biobank only including ADD-proxy cases (UKBB-P). b) observed in the diagnosed cases only analysis and estimated in Stage I including ADD-proxy cases. OR: odds-ratio

a

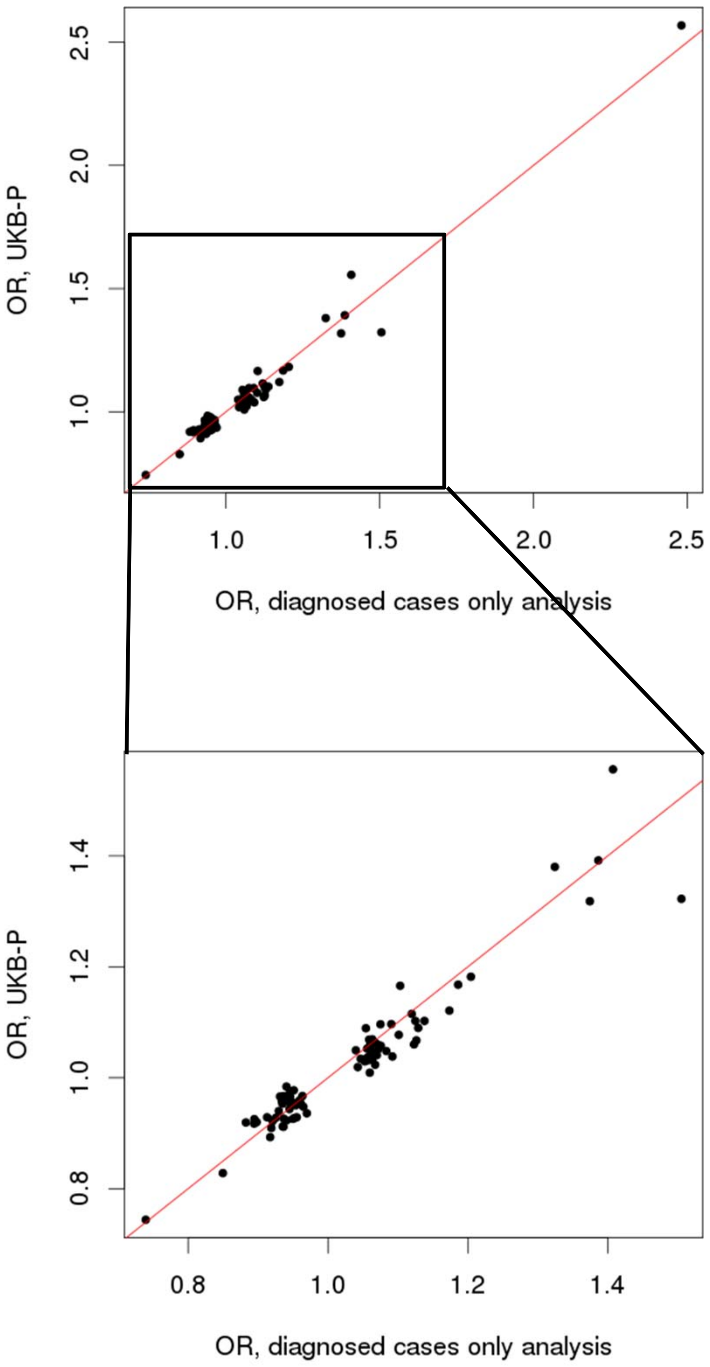

Supplementary Figure 30 continued

b

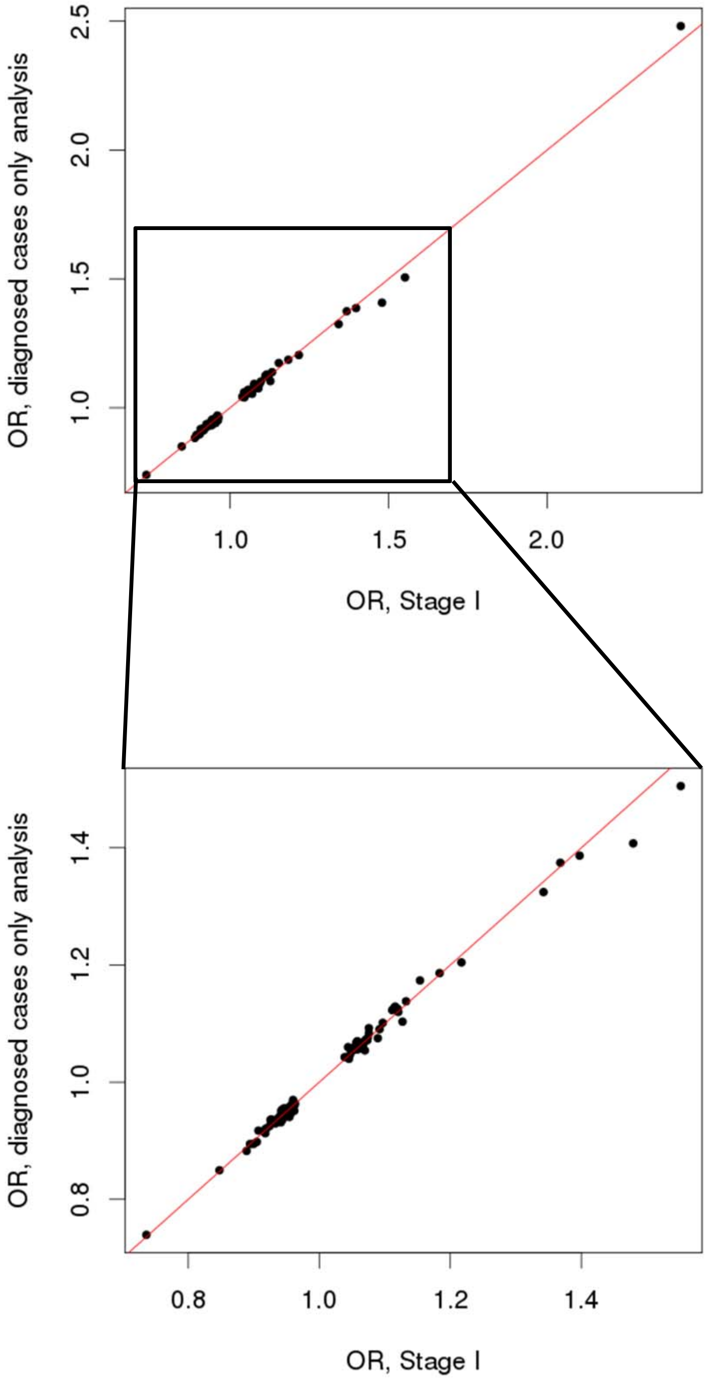

Supplementary Figure 31: Forest plots of the 8 HLA alleles associated with AD (FDR *P* value below 0.05).

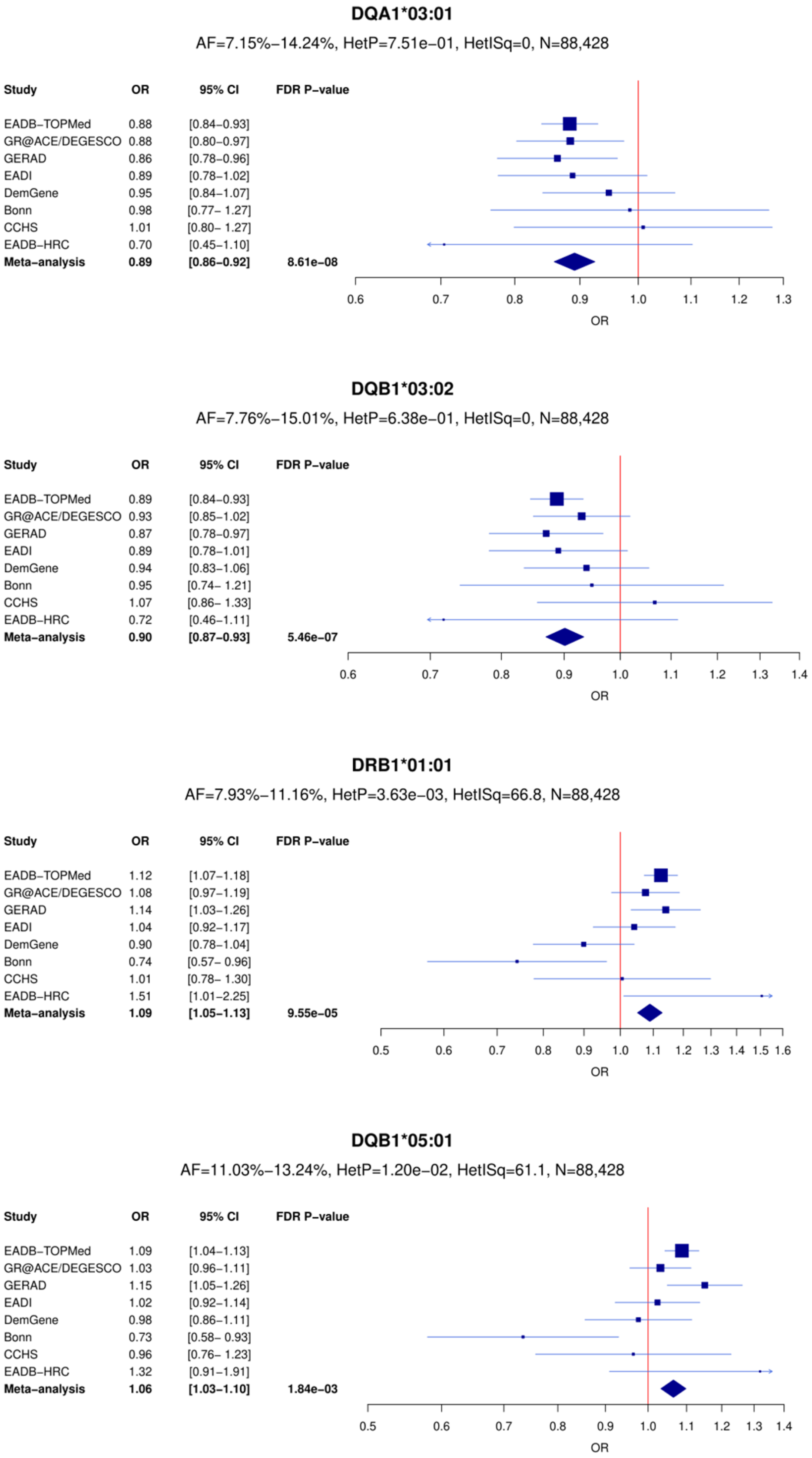

Supplementary Figure 31 continued

DRB1*04:04

AF=1.55%–5.86%, HetP=1.07e–01, HetISq=40.7, N=88,428

Study	OR	95% CI	FDR P–value
EADB–TOPMed	0.85	[0.77–0.94]	
GR@ACE/DEGESCO	0.94	[0.80–1.11]	
GERAD	0.76	[0.64–0.90]	
EADI	0.94	[0.75–1.18]	
DemGene	0.94	[0.79–1.13]	
Bonn	0.70	[0.41– 1.21]	
CCHS	1.30	[0.91– 1.86]	
EADB–HRC	0.34	[0.08–1.49]	
**Meta–analysis**	**0.88**	**[0.82–0.94]**	**2.77e–03**

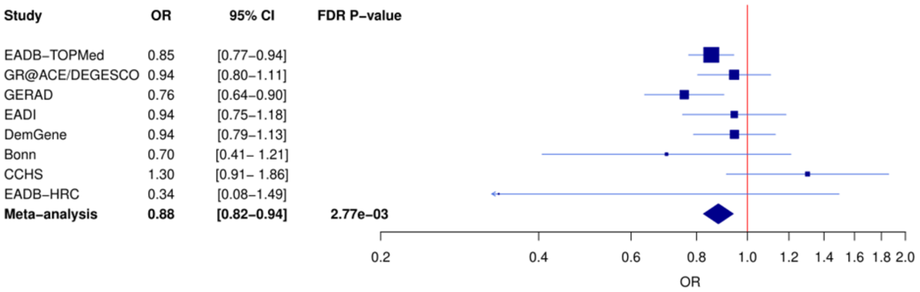

DQA1*01:01

AF=10.29%–11.97%, HetP=2.20e–03, HetISq=68.7, N=88,428

Study	OR	95% CI	FDR P–value
EADB–TOPMed	1.10	[1.05–1.15]	
GR@ACE/DEGESCO	1.01	[0.94–1.10]	
GERAD	1.13	[1.03–1.25]	
EADI	1.01	[0.90–1.12]	
DemGene	0.94	[0.82–1.08]	
Bonn	0.71	[0.55– 0.90]	
CCHS	0.96	[0.74– 1.23]	
EADB–HRC	1.42	[0.97–2.08]	
**Meta–analysis**	**1.06**	**[1.03–1.10]**	**4.96e–03**

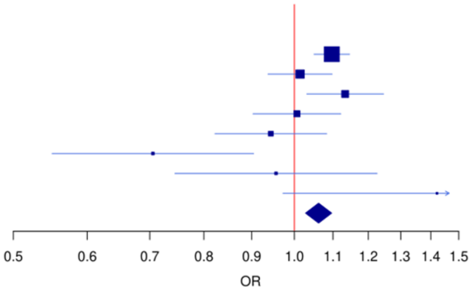

B*57:01

AF=2.5%–4.1%, HetP=2.00e–01, HetISq=28.5, N=88,428

Study	OR	95% CI	FDR P–value
EADB–TOPMed	1.10	[1.01–1.20]	
GR@ACE/DEGESCO	1.11	[0.93–1.31]	
GERAD	0.95	[0.81–1.12]	
EADI	1.15	[0.93–1.41]	
DemGene	1.26	[1.01–1.59]	
Bonn	1.44	[0.97– 2.15]	
CCHS	1.41	[0.98– 2.02]	
EADB–HRC	1.71	[0.85–3.46]	
**Meta–analysis**	**1.11**	**[1.04–1.18]**	**1.21e–02**

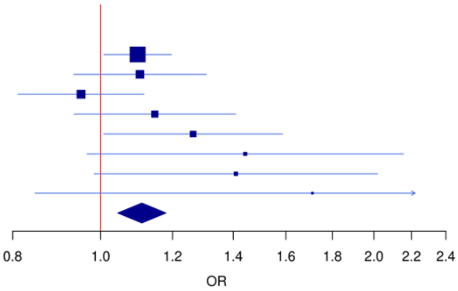

A*02:01

AF=24.22%–33.45%, HetP=2.68e–01, HetISq=20.3, N=88,428

Study	OR	95% CI	FDR P–value
EADB–TOPMed	1.04	[1.00–1.07]	
GR@ACE/DEGESCO	1.04	[0.98–1.10]	
GERAD	1.09	[1.02–1.17]	
EADI	1.02	[0.95–1.10]	
DemGene	0.99	[0.91–1.08]	
Bonn	1.00	[0.86– 1.18]	
CCHS	1.01	[0.86– 1.19]	
EADB–HRC	0.72	[0.53–0.98]	
**Meta–analysis**	**1.03**	**[1.01–1.06]**	**4.92e–02**

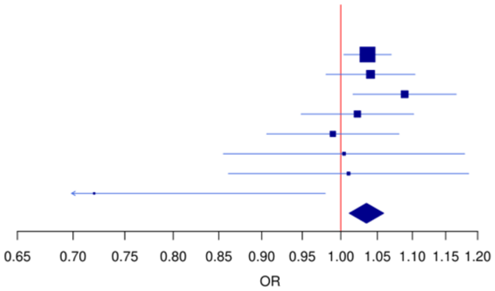

Supplementary Figure 32: Forest plots of the 3 three-locus haplotypes associated with AD (FDR *P* value below 0.05).

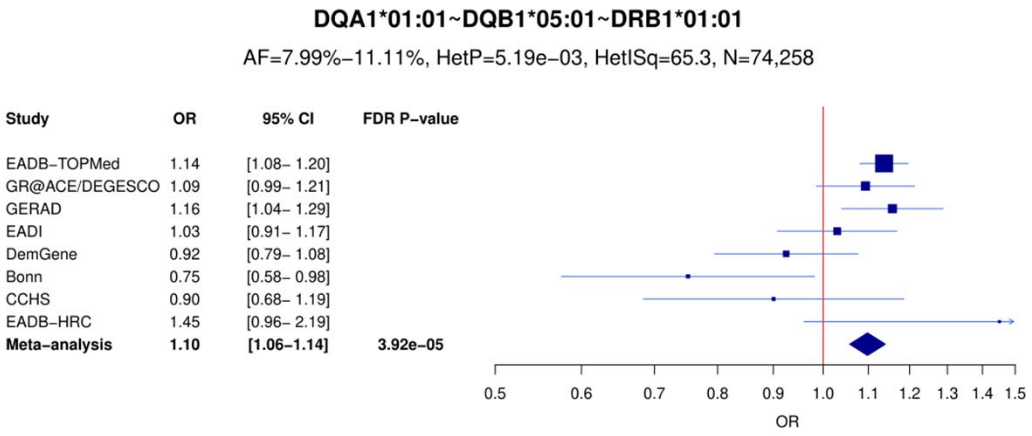

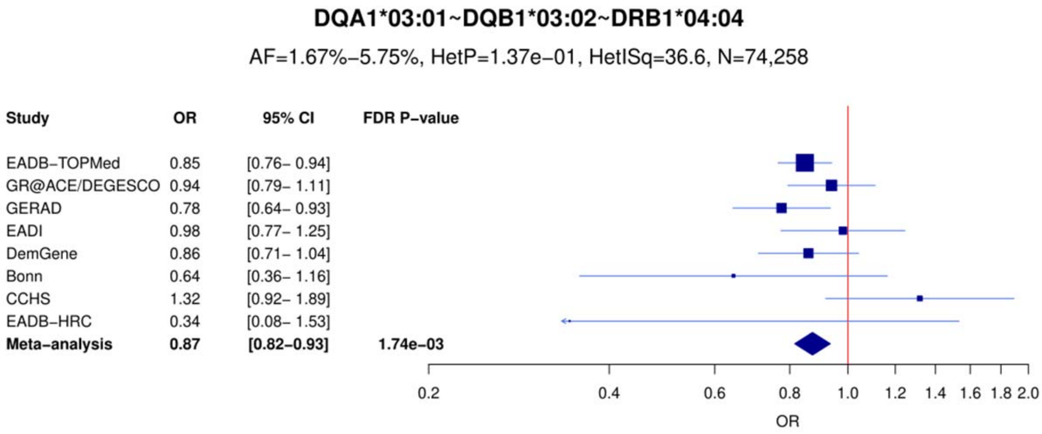

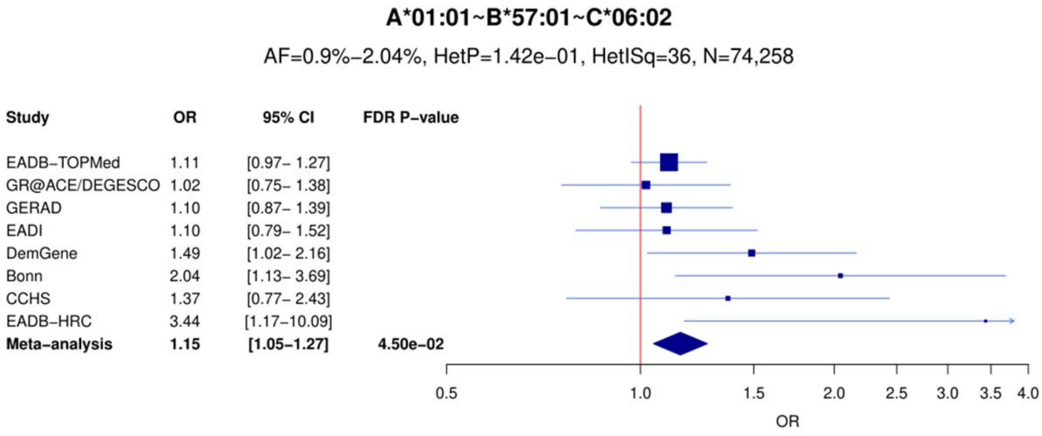

Supplementary Figure 33: Z-scores for neurodegenerative and AD related diseases for the risk allele of the ADD associated variants. *P* values (uncorrected) are denoted with \$ ($P < 5x10^{-8}$), X ($P < 1x10^{-5}$), # ($P < 1x10^{-3}$), * ($P < 0.05$).

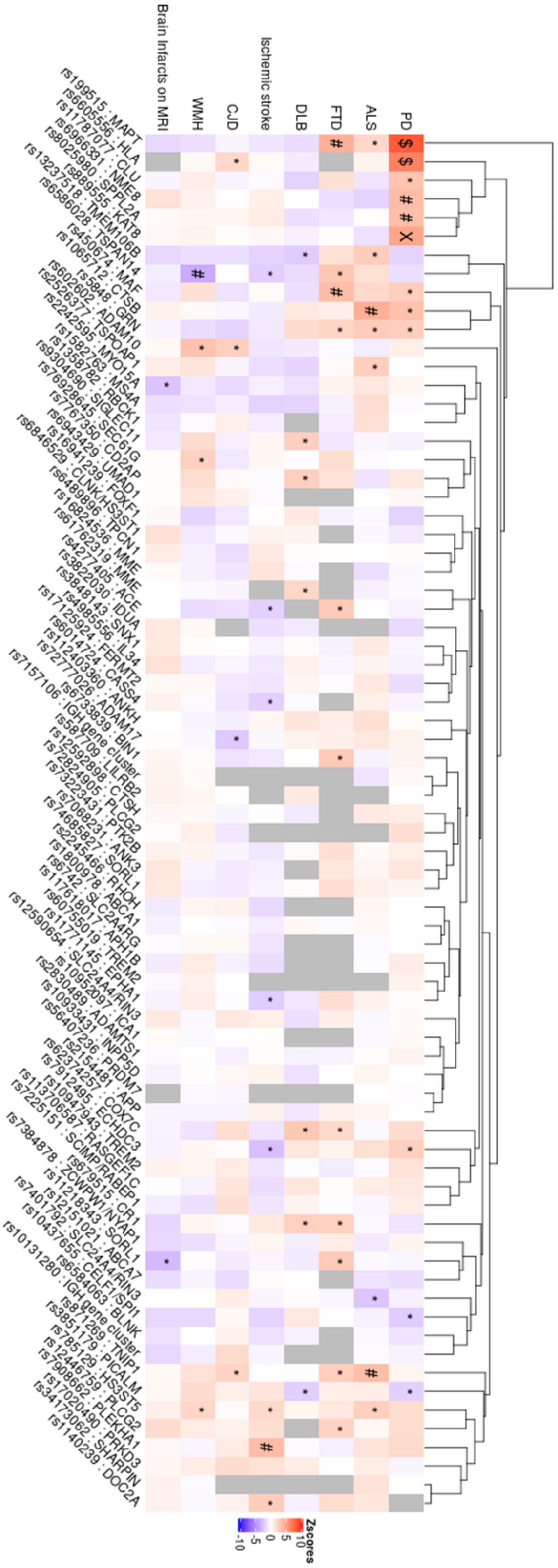

Supplementary Figure 34: Overview of the gene prioritization strategy used in the study. $Score_{gene}$ is the total weighted gene prioritization score for a gene, $Score_{top}$ is the top score observed in the investigated locus, $RelDiff_{gene}$ is the relative score difference for each gene in the investigated locus compared to the top-scoring gene, $MinRelDiff_{locus}$ is the minimum $RelDiff_{gene}$ observed in the investigated locus.

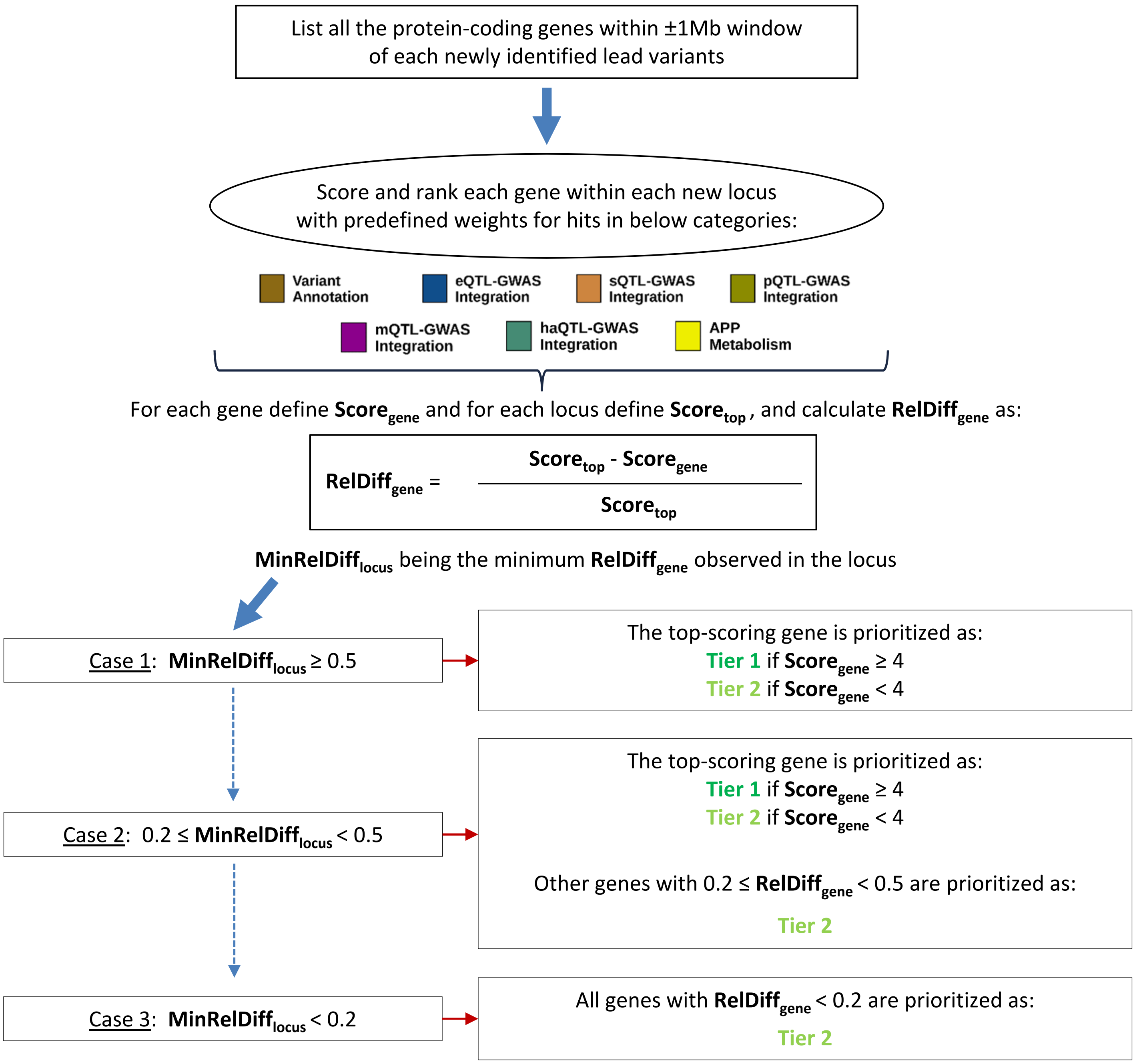

Supplementary Figure 35: Gene prioritization results (Loci 1-24).

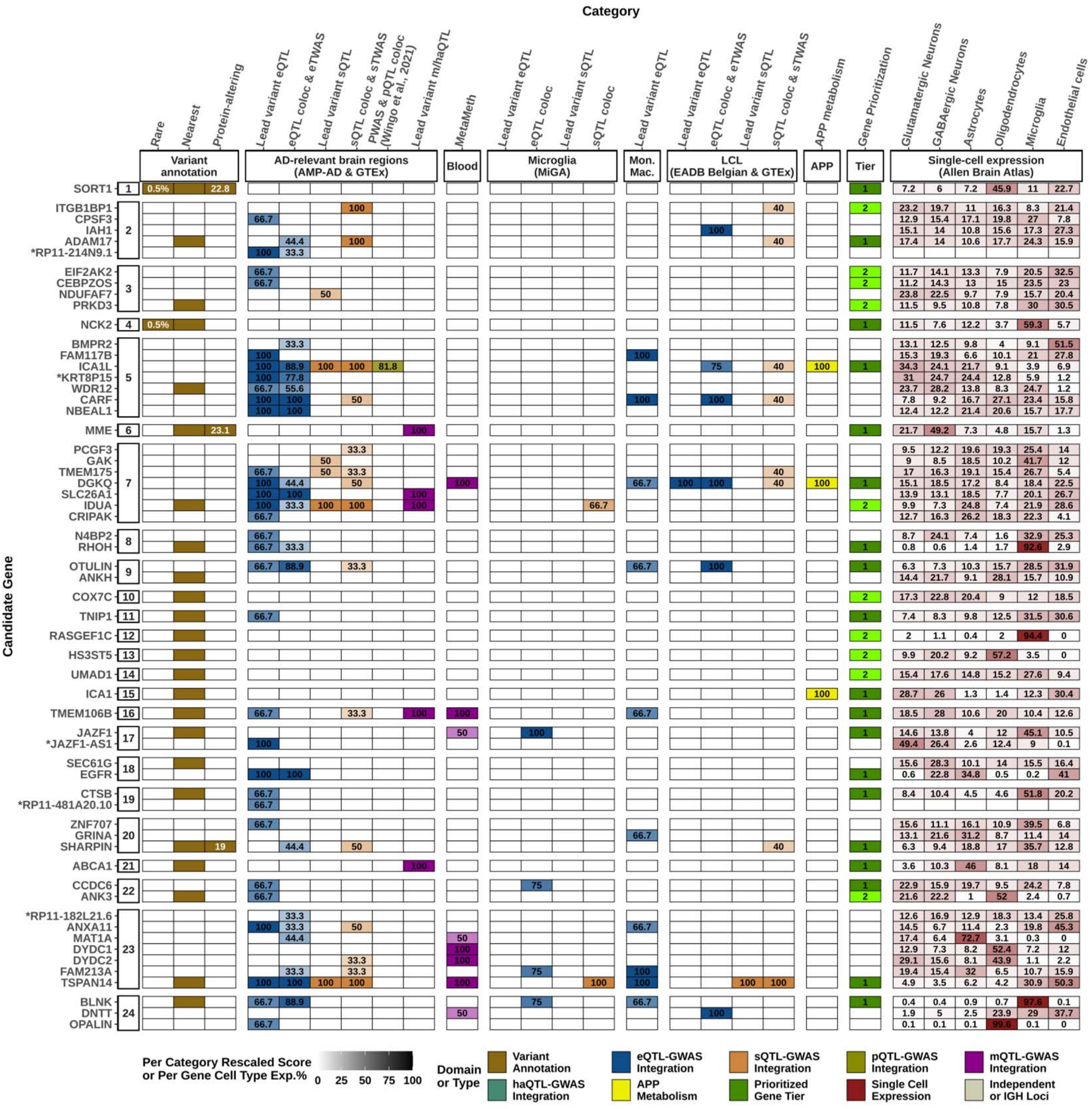

Supplementary Figure 35 continued (Loci 25-30)

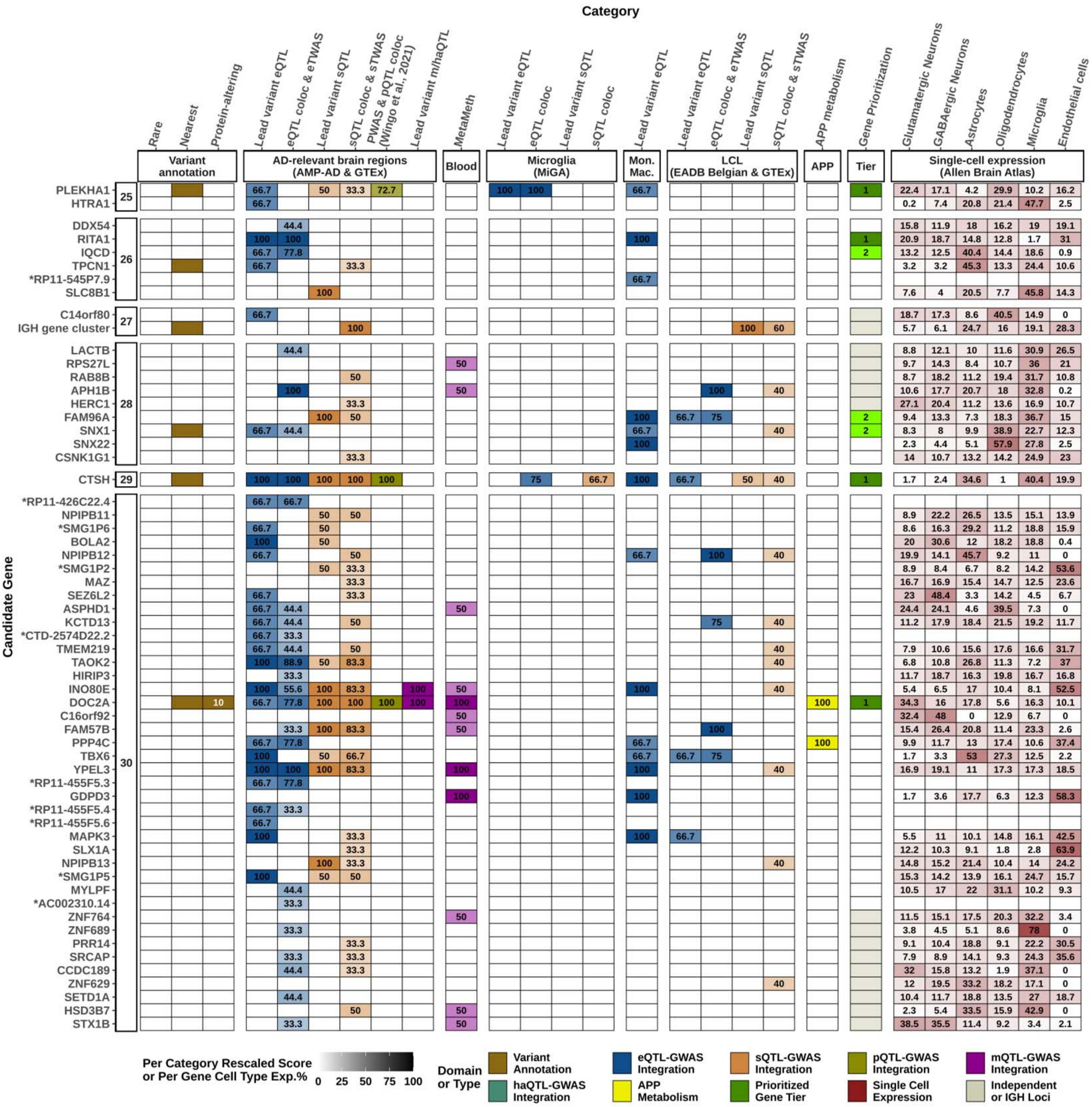

Supplementary Figure 35 continued (Loci 31-42)

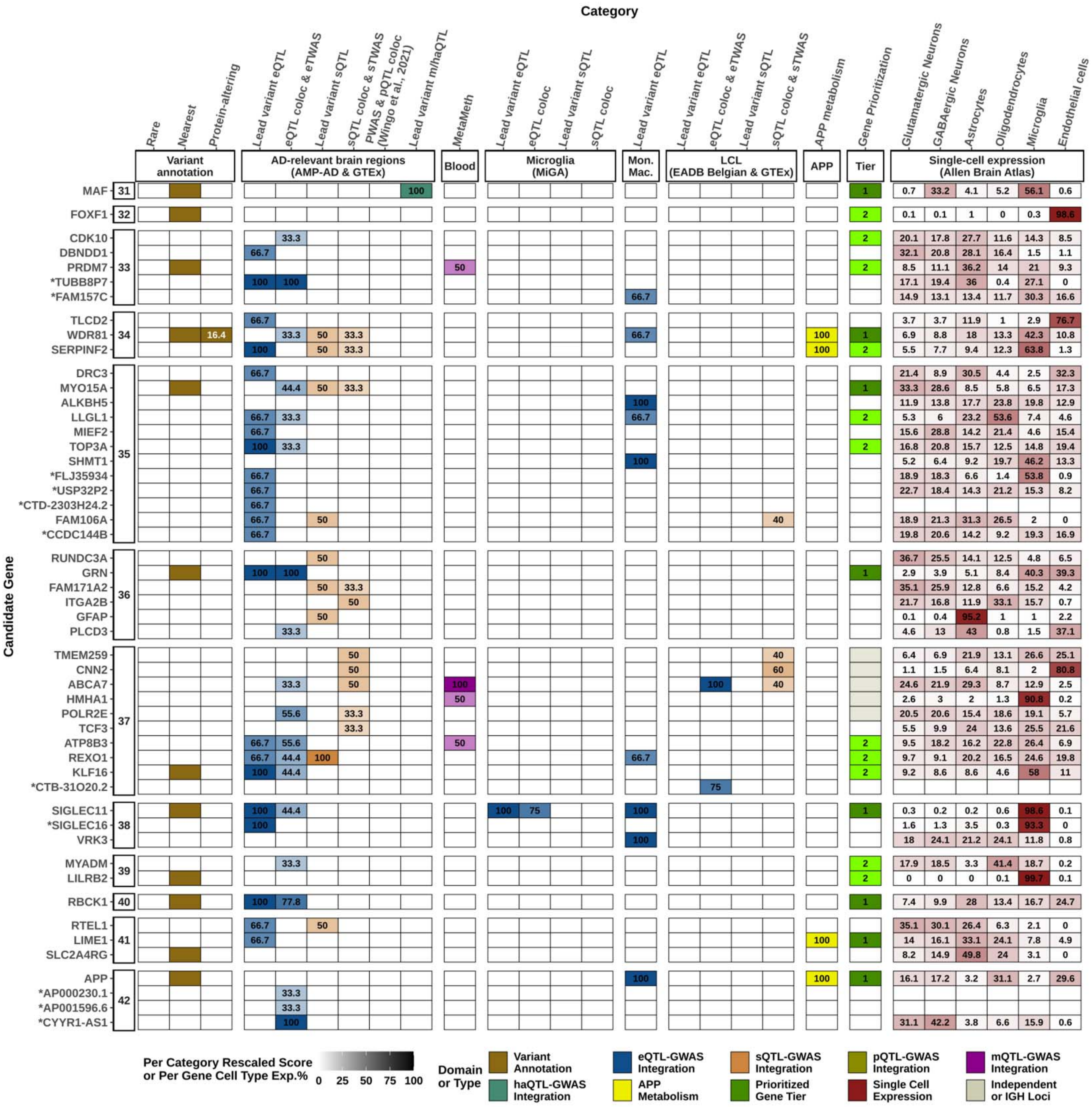

Supplementary Figure 36: eQTL effects of lead variants within novel ADD risk loci in (a) AD-relevant brain regions, LCL, microglia, blood, and (b) in naïve state and stimulated macrophages and monocytes.

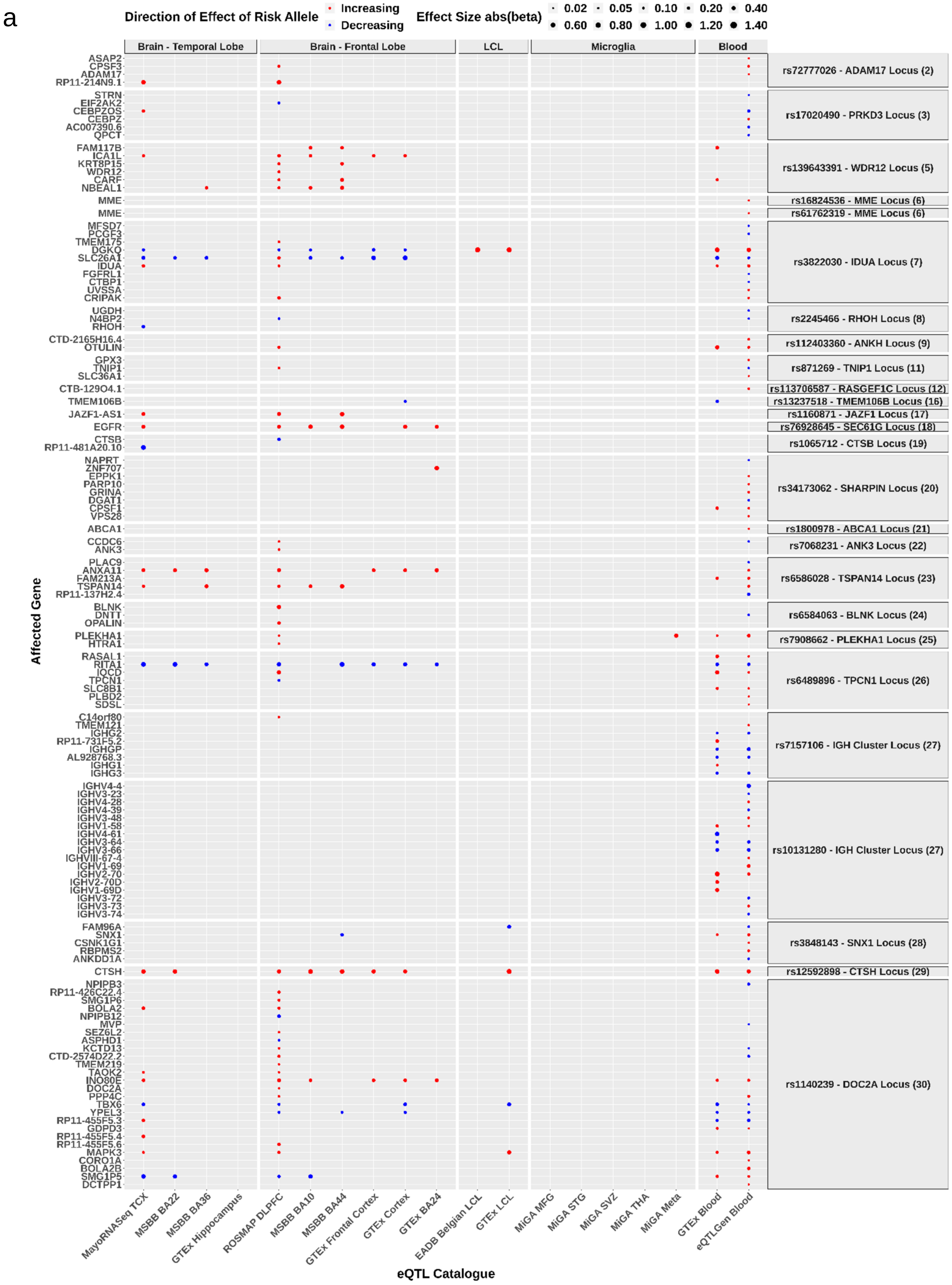

Supplementary Figure 36 continued

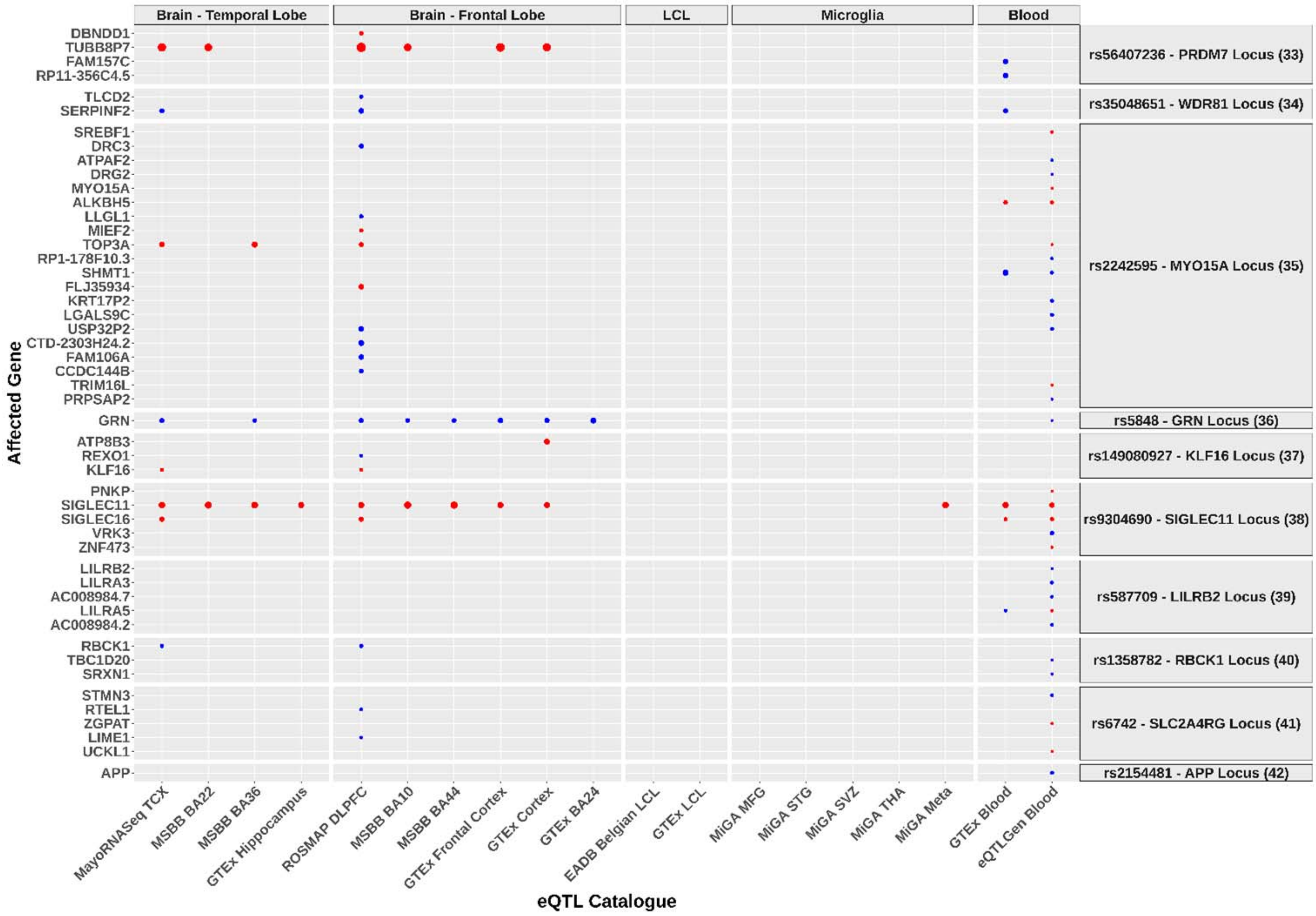

b

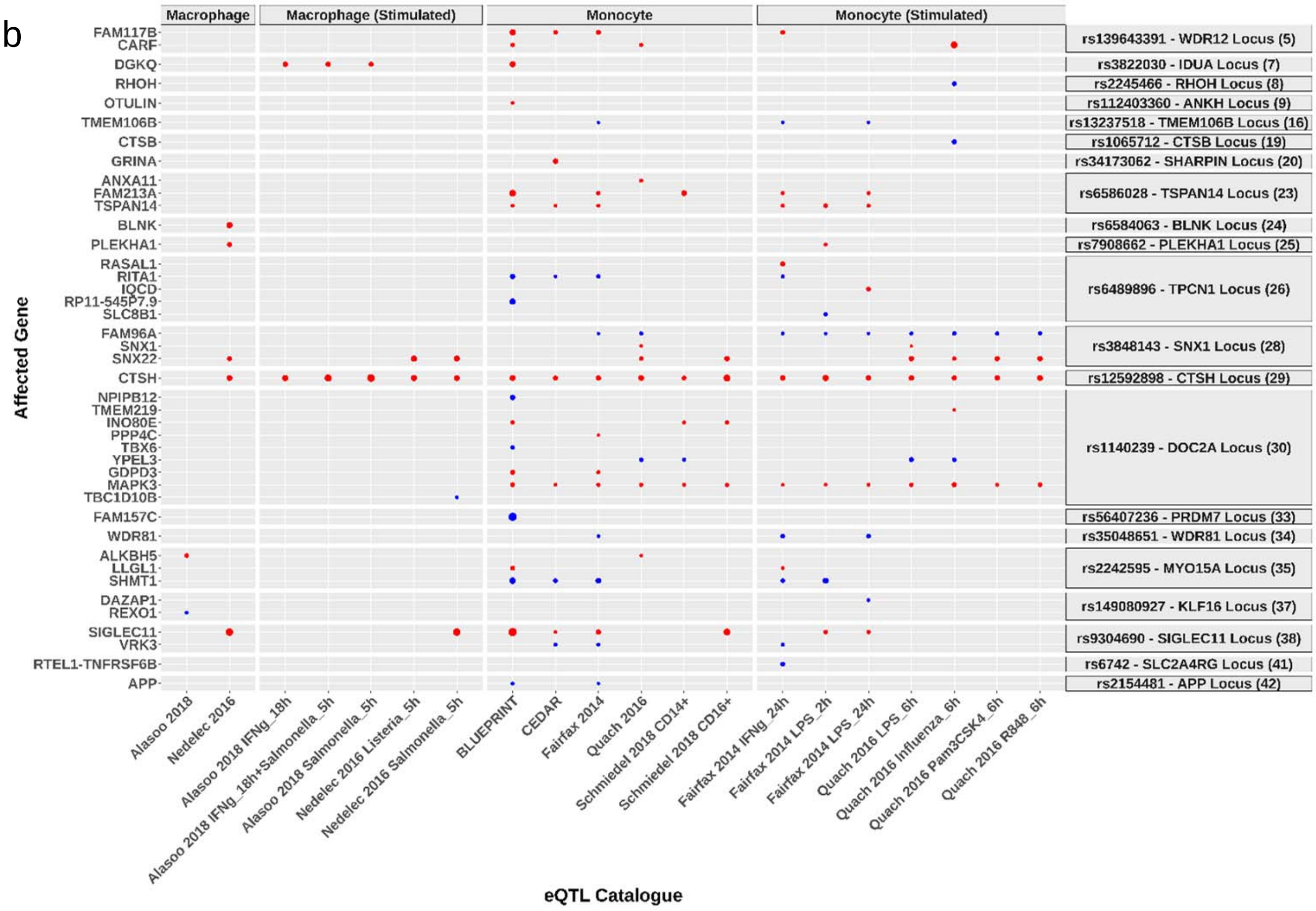

Supplementary Figure 37: sQTL effects of lead variants within novel ADD risk loci in AD-relevant brain regions, LCL, microglia, and blood.

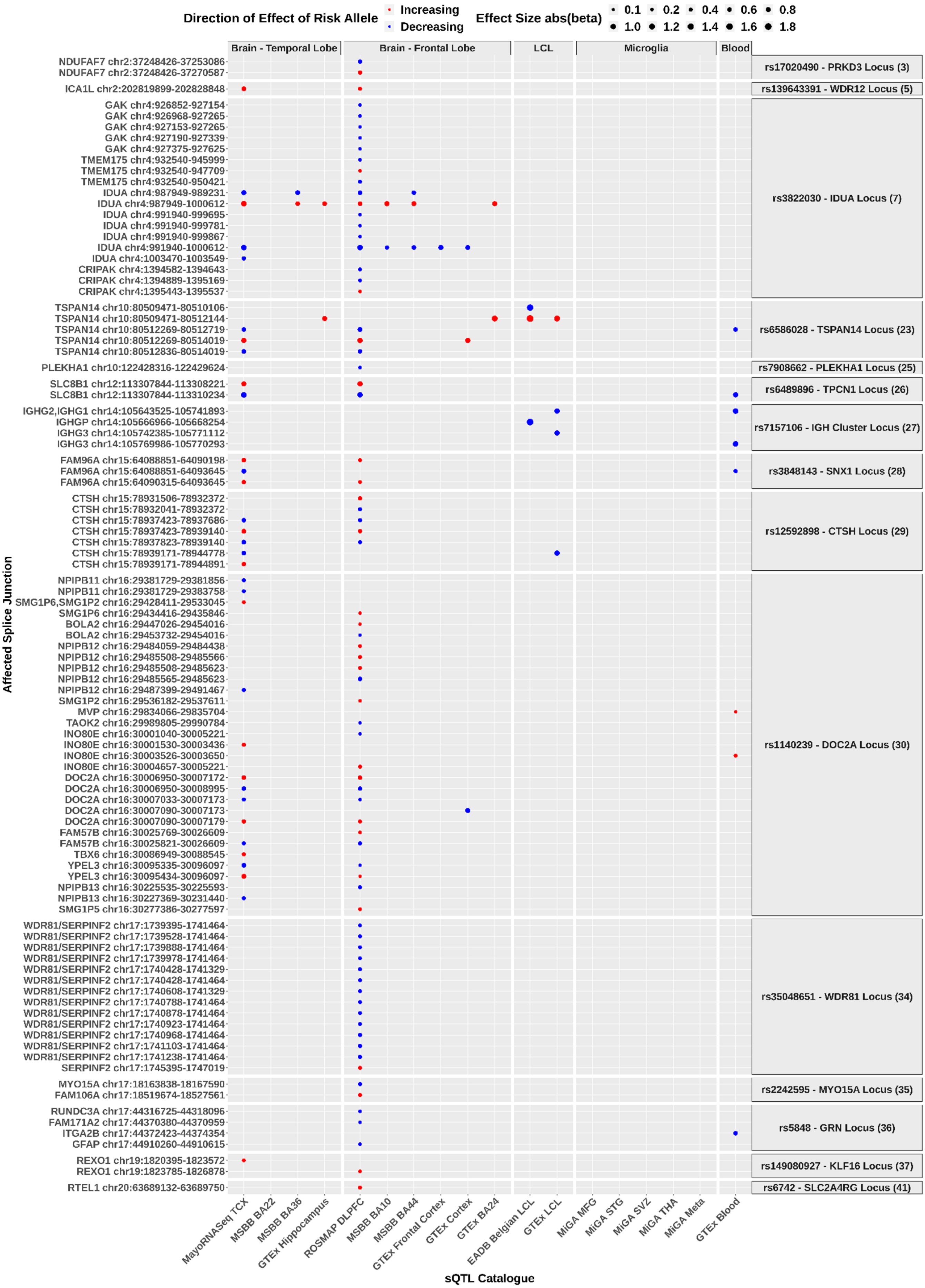

Supplementary Figure 38: (a) mQTL and haQTL effects of lead variants within novel ADD risk loci. (b) ADD-associated predicted methylation results using MetaMeth.

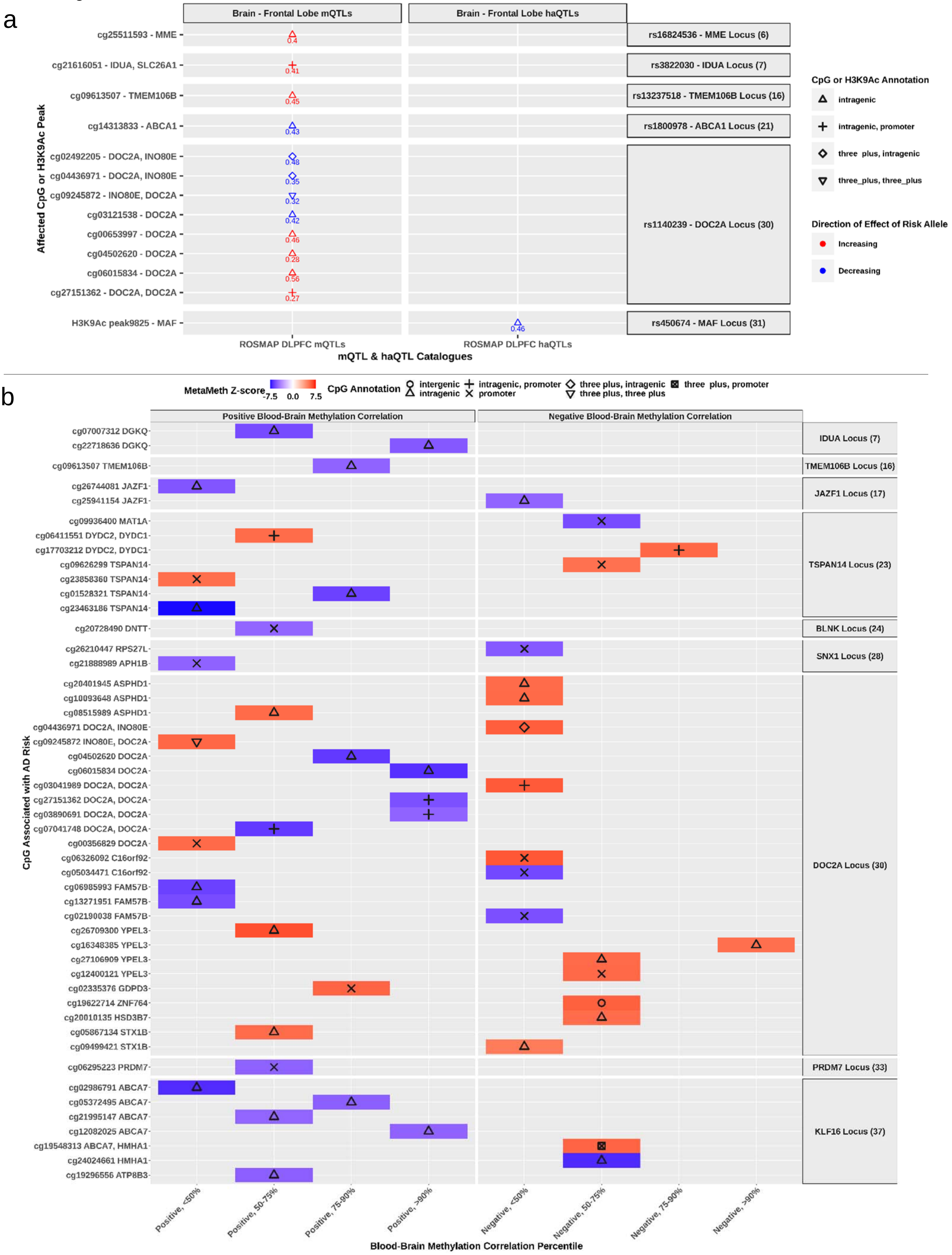

Supplementary Figure 39: Colocalization between eQTL signals for genes and ADD association signals.

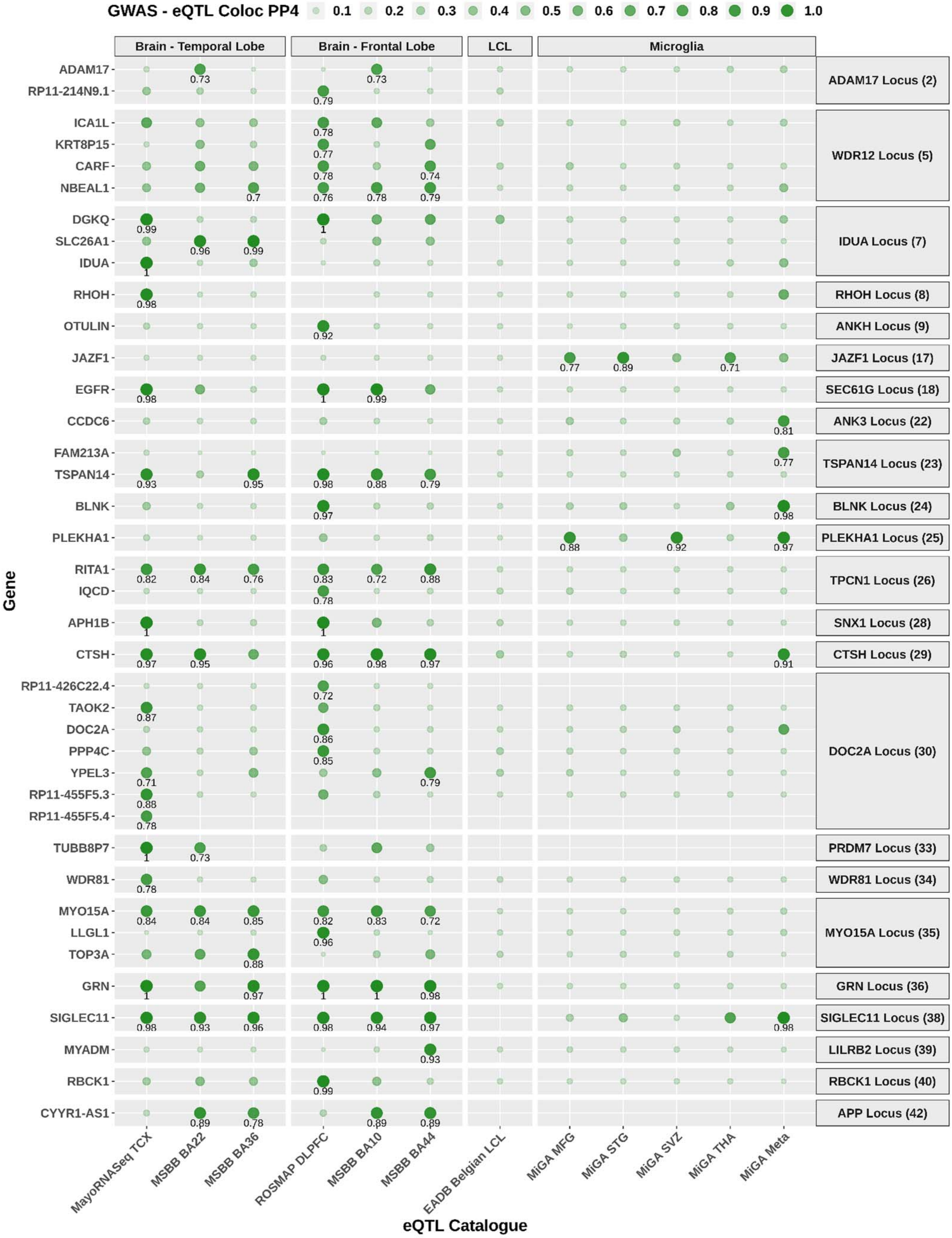

Supplementary Figure 40: Colocalization between sQTL signals for splice junctions and ADD association signals.

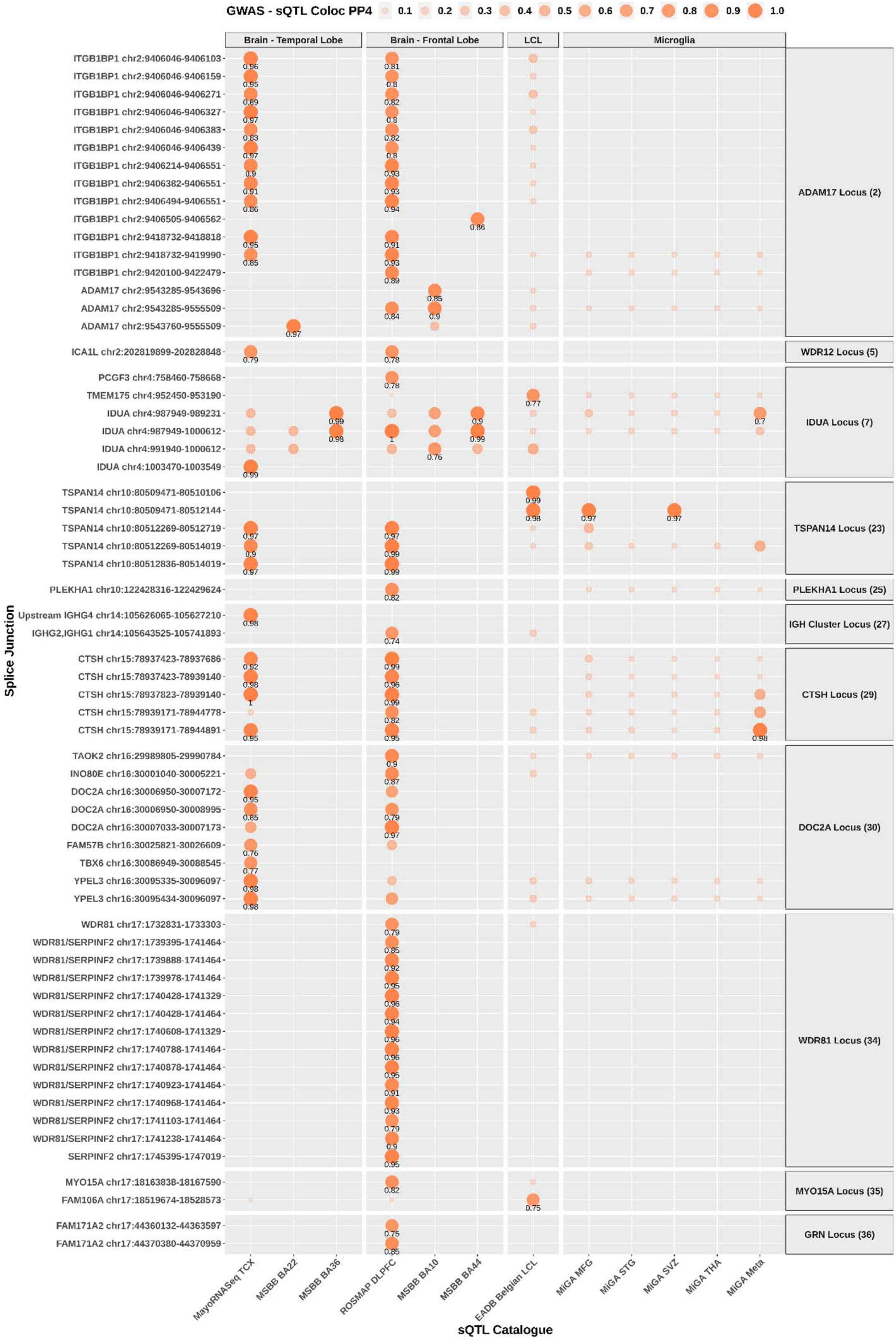

Supplementary Figure 41: TWAS of ADD using Expression Reference Panels.

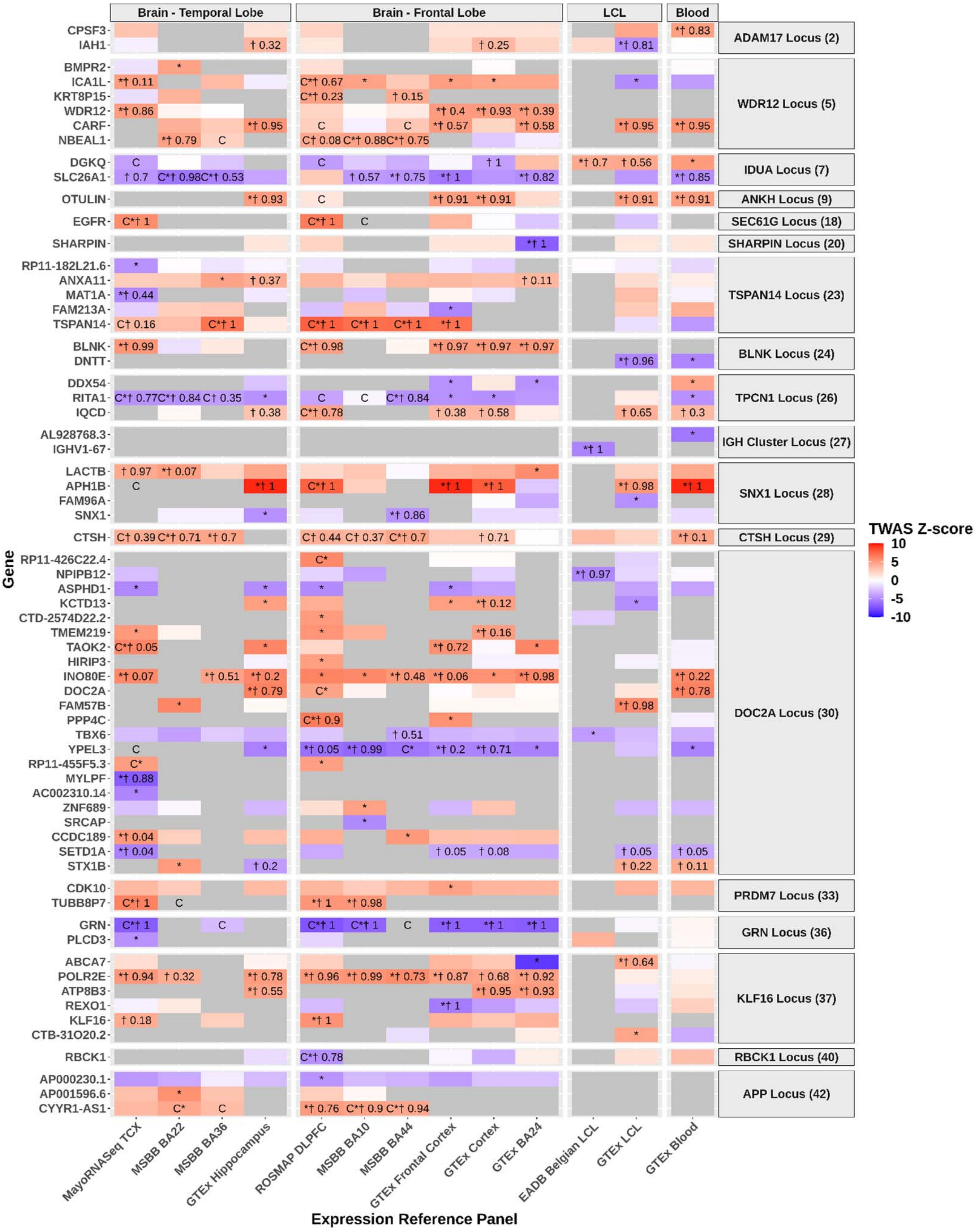

Supplementary Figure 42: TWAS of ADD using Splicing Reference Panels.

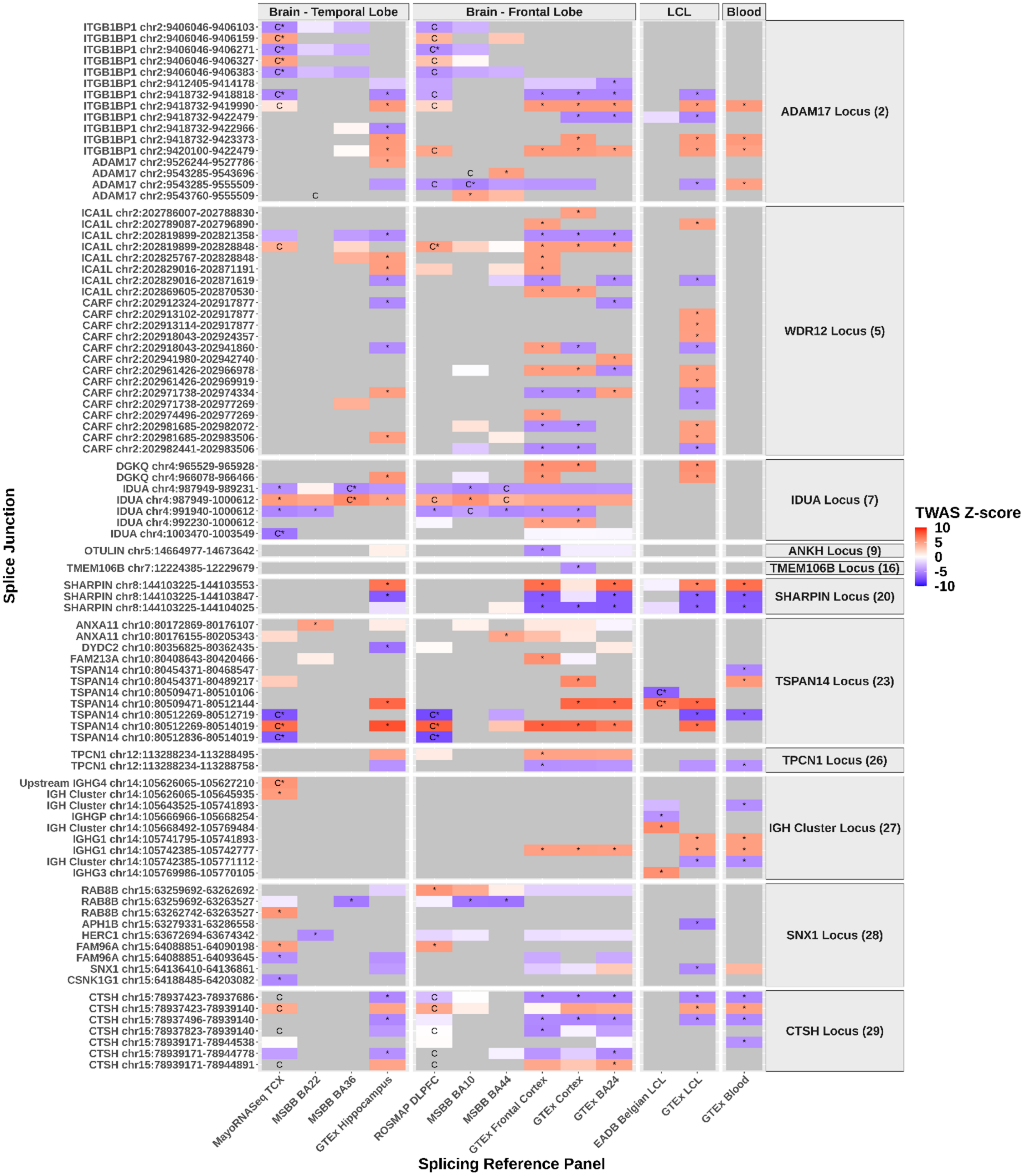

Supplementary Figure 42 continued

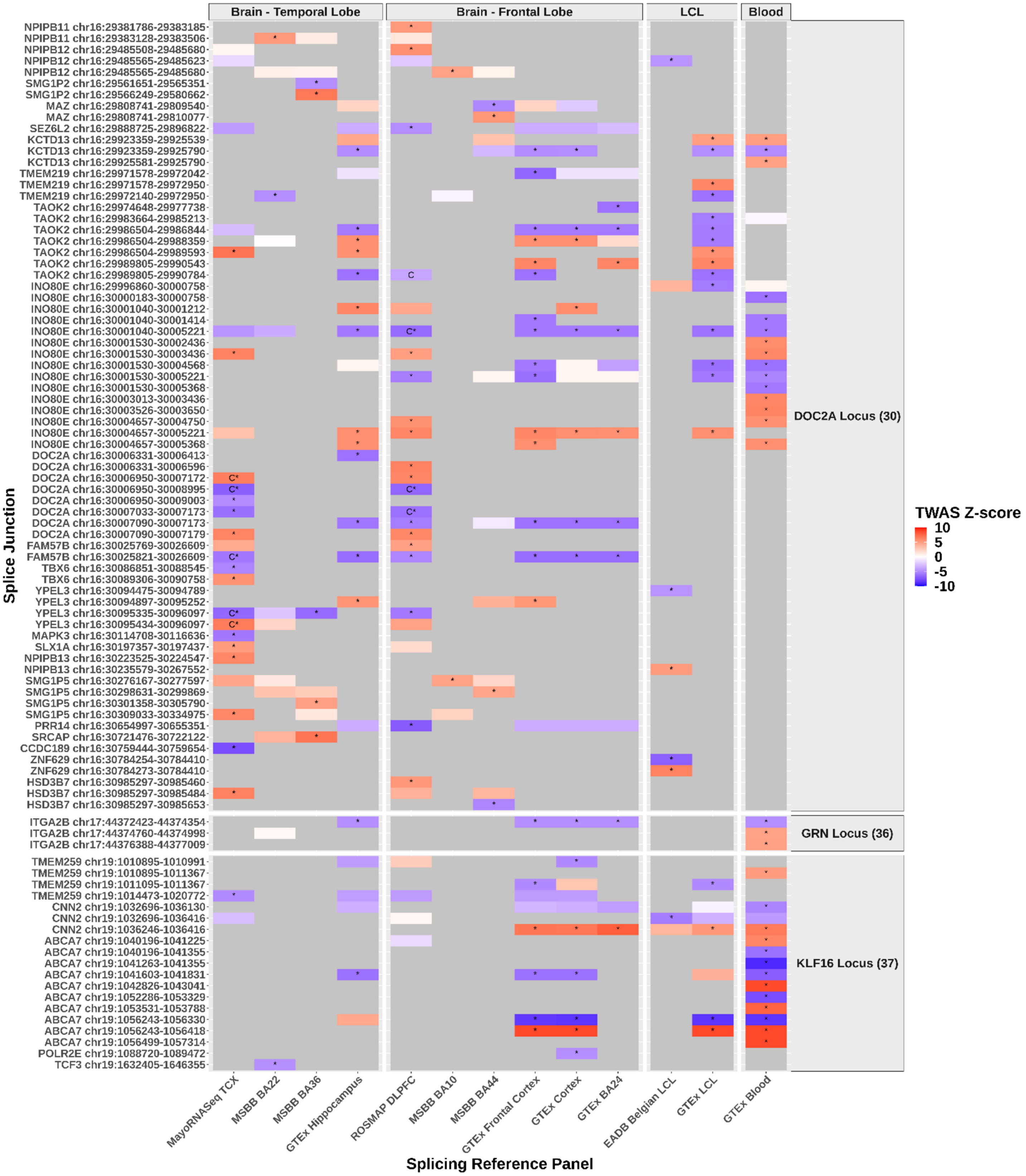

Supplementary Figure 43: PWAS of ADD using Protein Expression Reference Panels, as reported by Wingo et al., 2021.

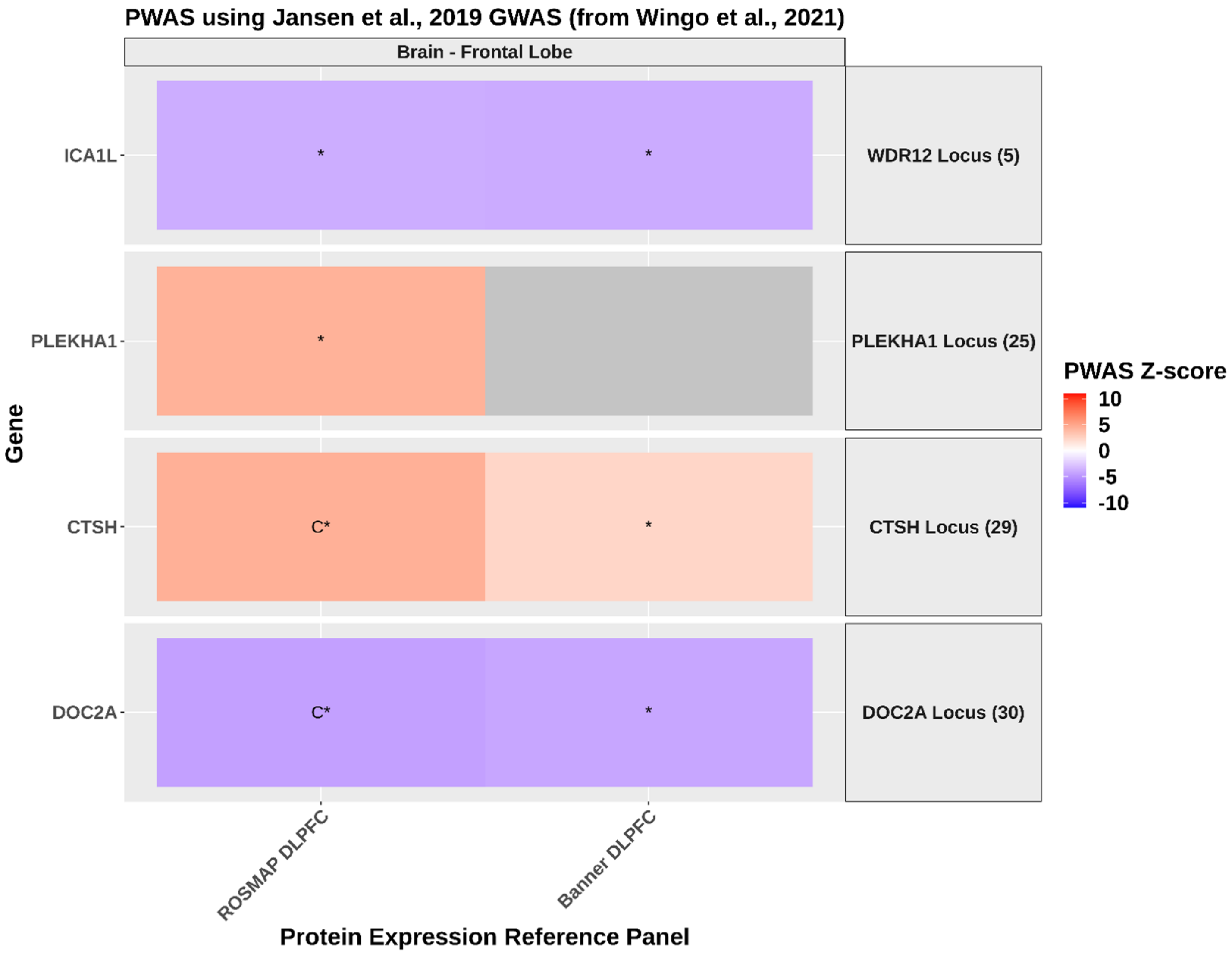

Supplementary Figure 44: Fine-mapping of expression TWAS results. (a) *GRN* locus in MayoRNASeq TCX, (b) *KLF16* locus in ROSMAP DLPFC, (c) *TSPAN14* locus in MSBB BA36, and (d) *DOC2A* locus in GTEx hippocampus.

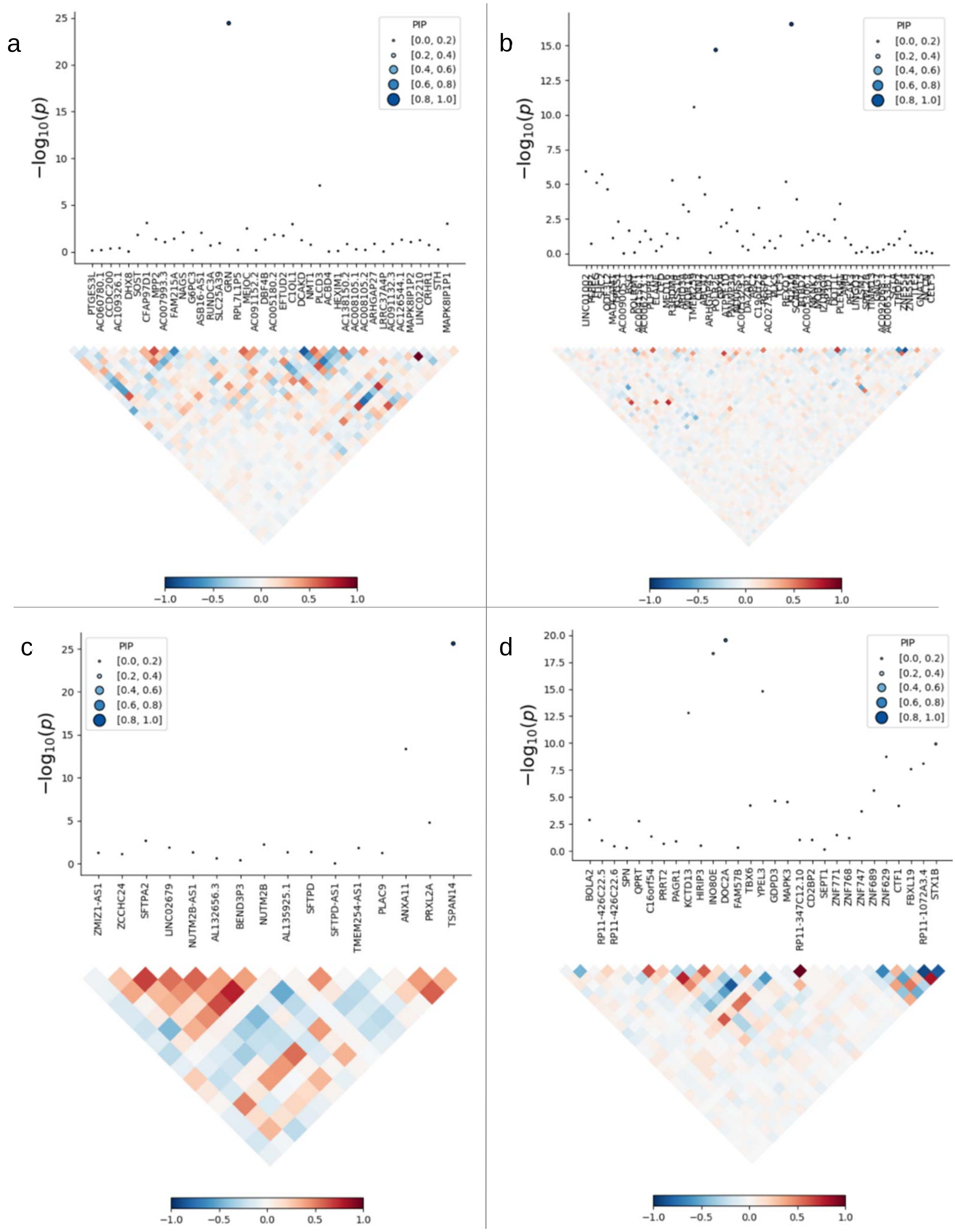

Supplementary Figure 45: Mean fluorescence intensity variations (log2 fold-change + SEM, triplicate experiments) of the mCherry signal obtained after the silencing of genes associated with the ADD risk in HEK293 cells stably over-expressing a mCherry-APP695WT-YFP in the 42 new loci (Chapuis et al., 2017).

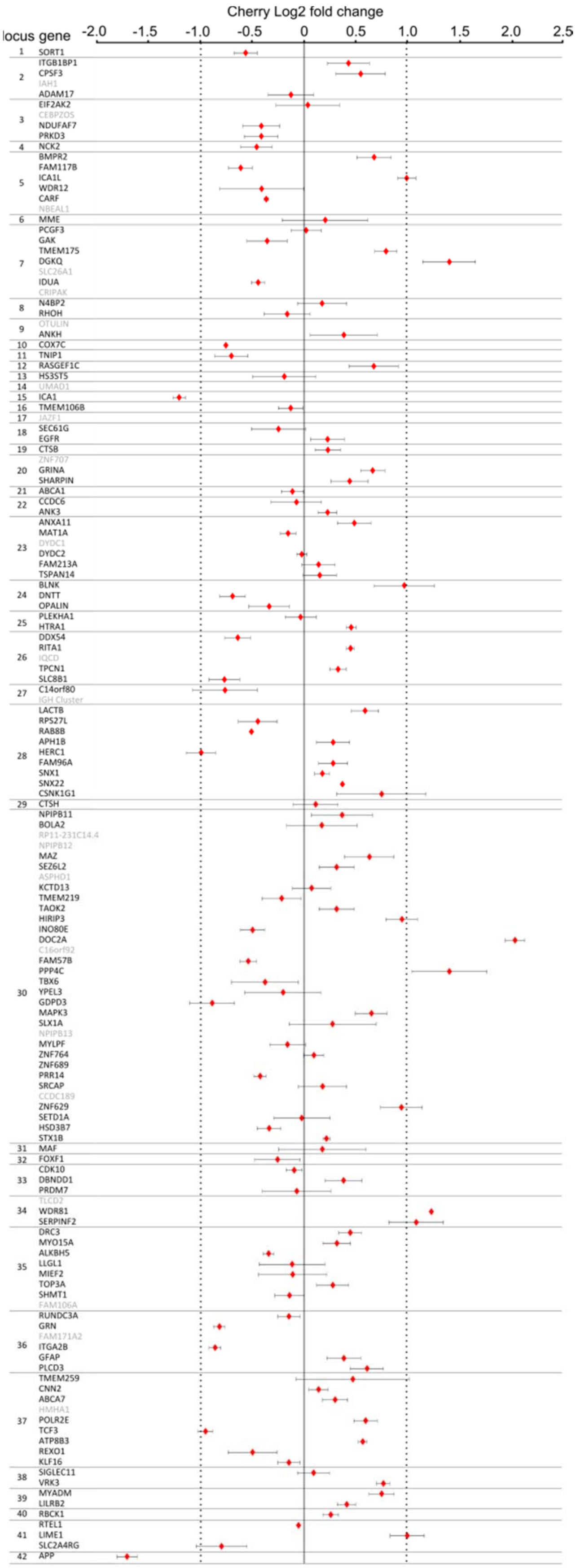

Supplementary Figure 46: STRING protein interaction analysis. The main networks are shown in a) previous genes, b) prioritized new genes and c) combined datasets.

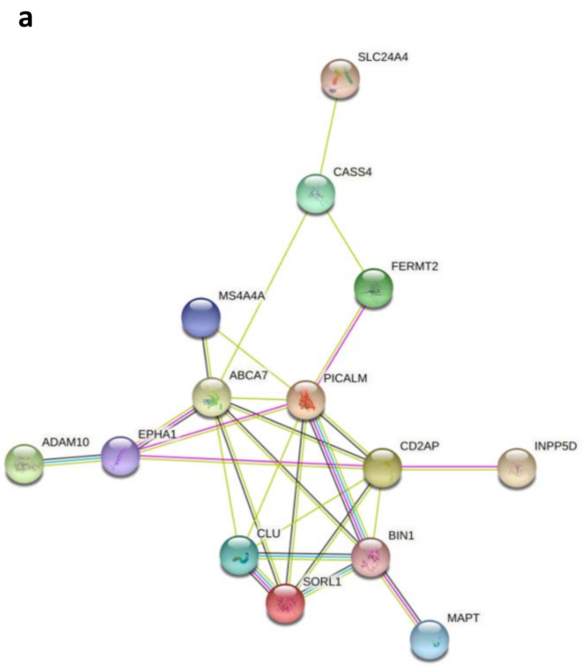

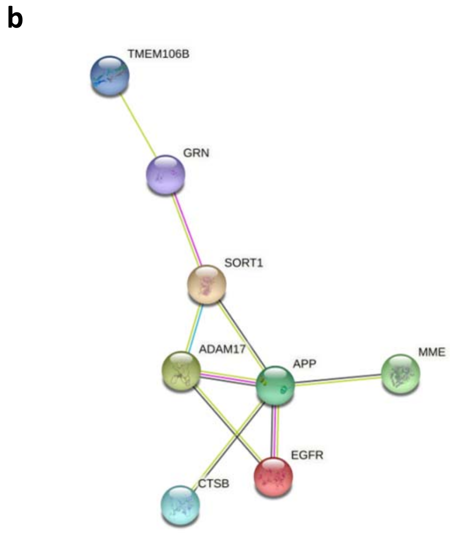

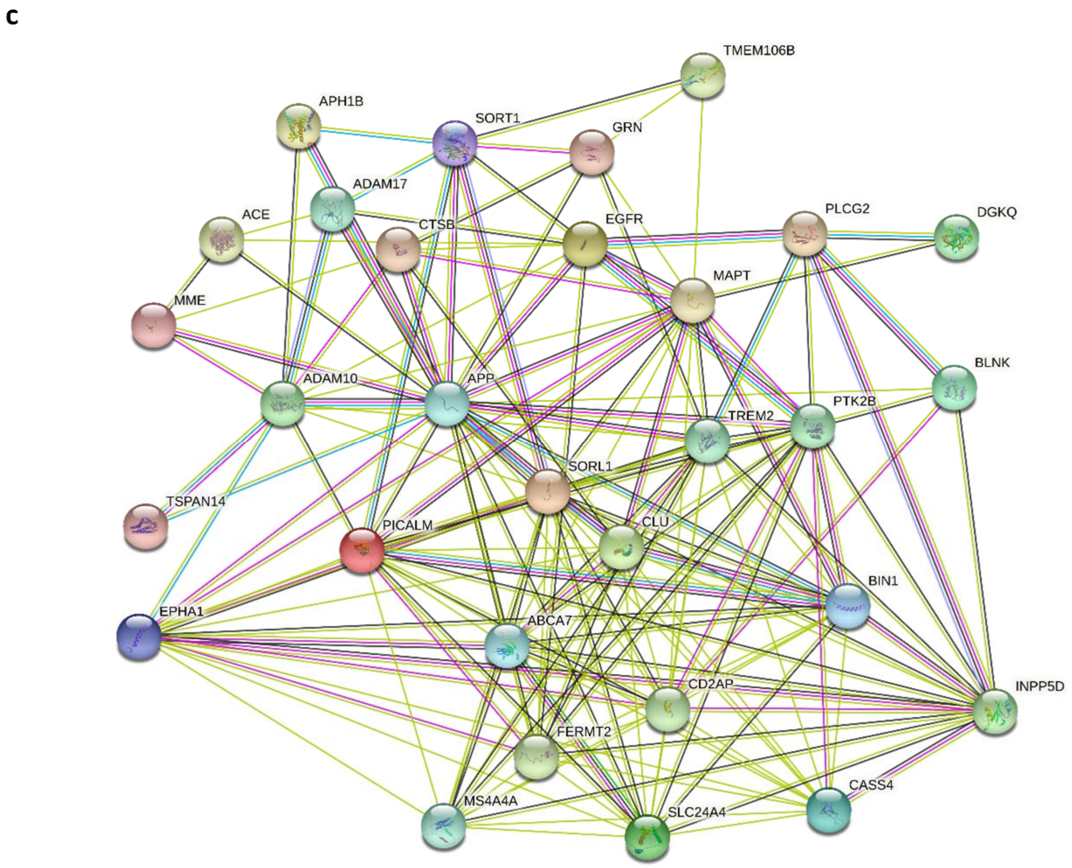

Supplementary Figure 47: Association of GRS with the risk of progression to all-causes-dementia in population-based (a) and MCI (b) cohorts.

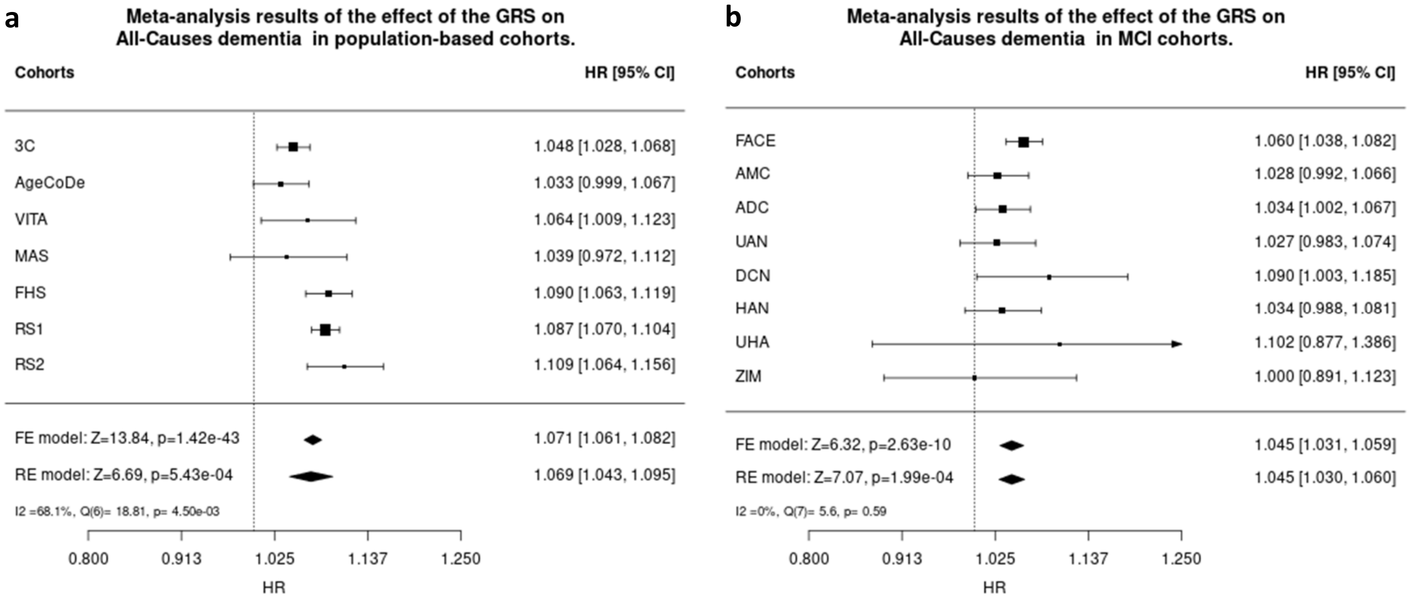

Supplementary Figure 48: TNF complex and signaling

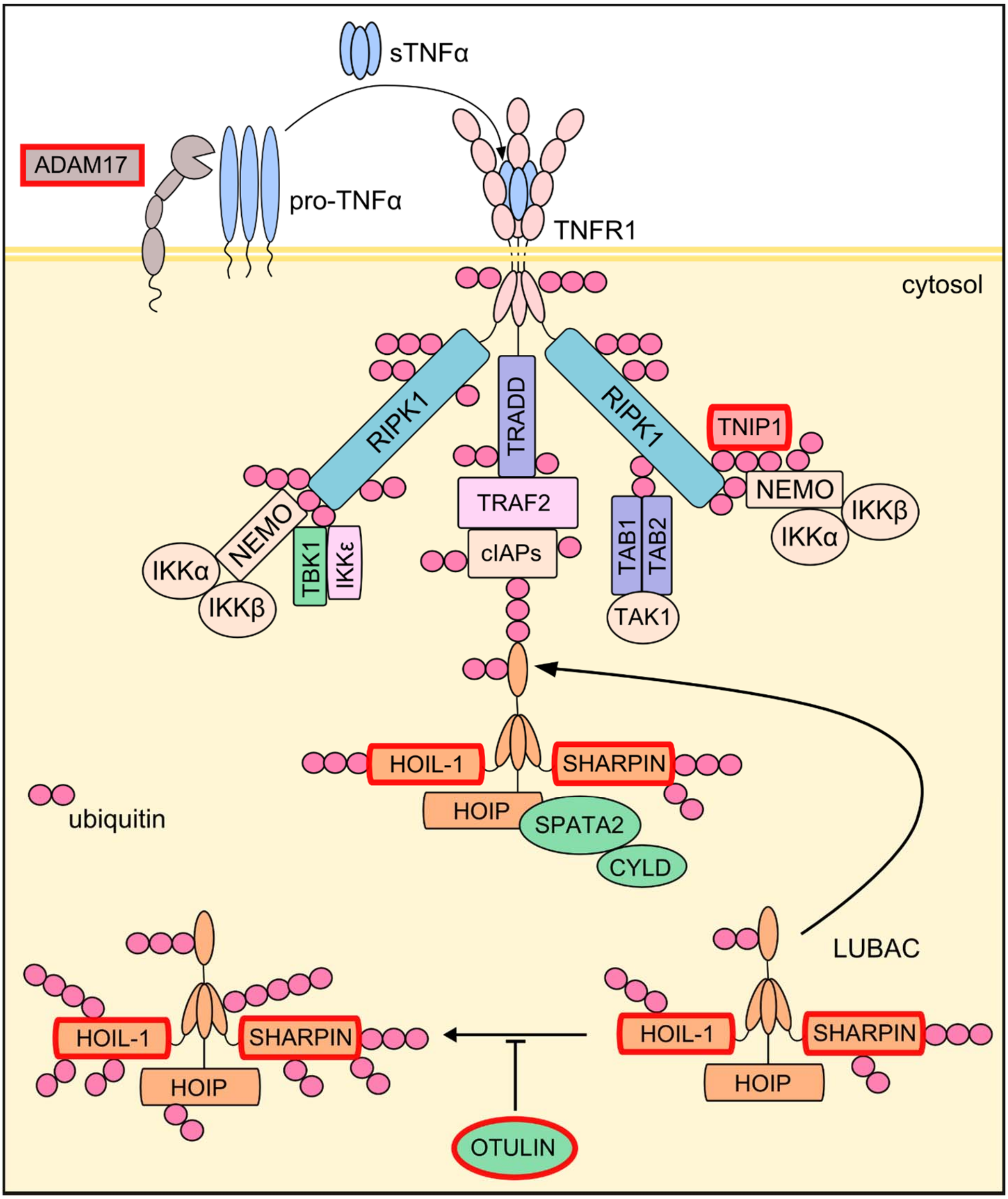

Supplementary Figure 49: EADB sample quality control

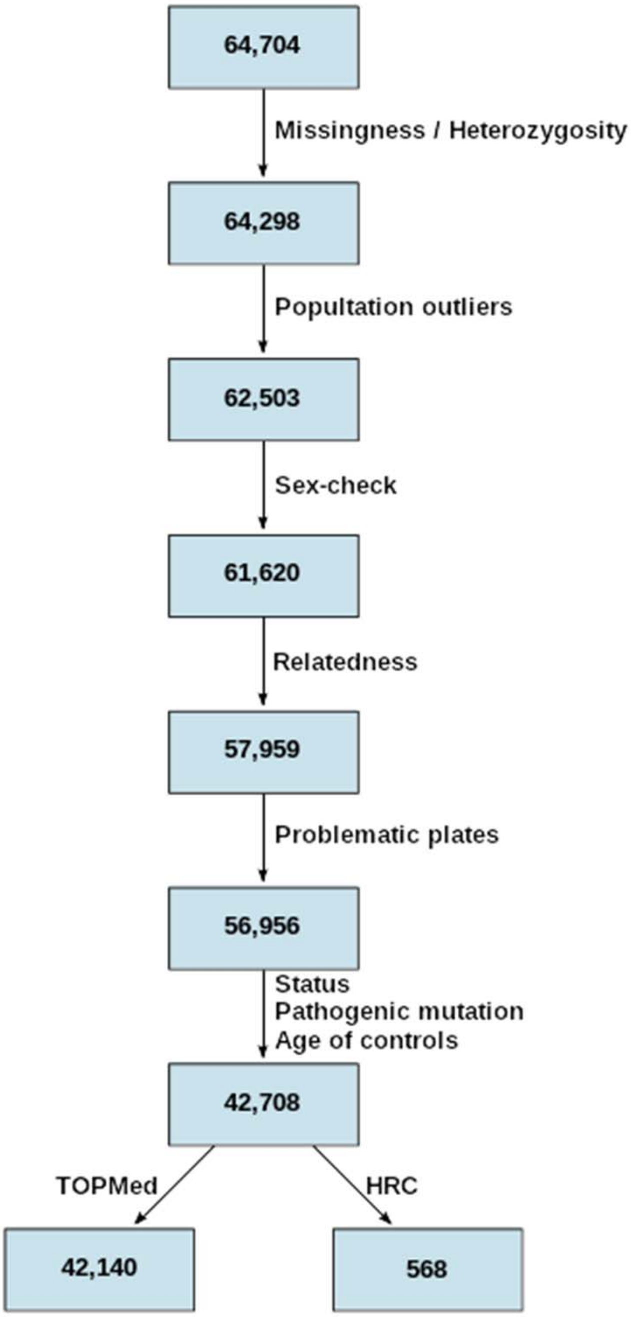

Supplementary Figure 50: EADB variant quality control

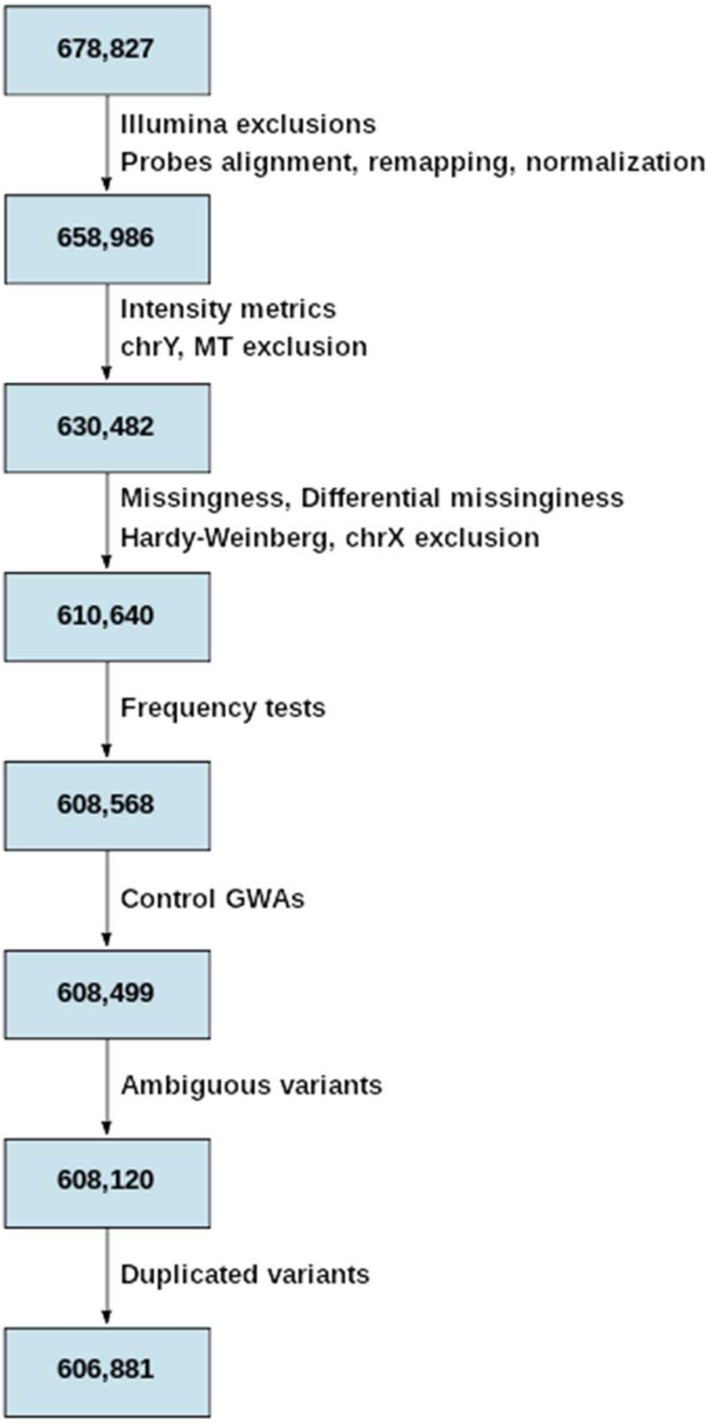

Supplement: Supplementary file 1 — Supplementary Note, Methods, Results and Figures 1–50. [file 41588_2022_1024_MOESM1_ESM.pdf]
