# Supplementary material for: New insights into the genetic etiology of Alzheimer’s disease and related dementias
Source: Nat Genet. 2022 Apr 4;54(4):412–36. doi: 10.1038/s41588-022-01024-z (PMC9005347; doi:10.1038/s41588-022-01024-z)
Supplement: Supplementary file 2 — Reporting Summary. [file 41588_2022_1024_MOESM2_ESM.pdf]

nature research

Corresponding author(s): Céline Bellenguez
Jean-Charles Lmabert

Last updated by author(s): Dec 10, 2021

Reporting Summary

Nature Research wishes to improve the reproducibility of the work that we publish. This form provides structure for consistency and transparency in reporting. For further information on Nature Research policies, see our Editorial Policies and the Editorial Policy Checklist.

Statistics

For all statistical analyses, confirm that the following items are present in the figure legend, table legend, main text, or Methods section.

n/a	Confirmed	
☐	☒	The exact sample size (*n*) for each experimental group/condition, given as a discrete number and unit of measurement
☐	☒	A statement on whether measurements were taken from distinct samples or whether the same sample was measured repeatedly
☐	☒	The statistical test(s) used AND whether they are one- or two-sided<br>*Only common tests should be described solely by name; describe more complex techniques in the Methods section.*
☐	☒	A description of all covariates tested
☐	☒	A description of any assumptions or corrections, such as tests of normality and adjustment for multiple comparisons
☐	☒	A full description of the statistical parameters including central tendency (e.g. means) or other basic estimates (e.g. regression coefficient) AND variation (e.g. standard deviation) or associated estimates of uncertainty (e.g. confidence intervals)
☐	☒	For null hypothesis testing, the test statistic (e.g. *F*, *t*, *r*) with confidence intervals, effect sizes, degrees of freedom and *P* value noted<br>*Give P values as exact values whenever suitable.*
☒	☐	For Bayesian analysis, information on the choice of priors and Markov chain Monte Carlo settings
☒	☐	For hierarchical and complex designs, identification of the appropriate level for tests and full reporting of outcomes
☒	☐	Estimates of effect sizes (e.g. Cohen's *d*, Pearson's *r*), indicating how they were calculated

Our web collection on statistics for biologists contains articles on many of the points above.

Software and code

Policy information about availability of computer code

Data collection	No software was used.
Data analysis	Bedtools 2.27.0<br>bcftools 1.9<br>bwa 0.7.17<br>coloc 4.0.4<br>Eagle 2.4; 2.0.5<br>EIGENSOFT 7.2.1<br>EIGENSTRAT<br>Enhanced FastQTL 2.184_gtex<br>EstiMeth 1.1<br>FlashPCA 2.0<br>FOCUS 0.7<br>GCTA-COJO from gcta 1.93.2beta<br>GENESIS 2.14.4<br>GenomeStudio 2.0.3<br>Gentrain 3.0<br>ggplot2 3.3.3<br>Guppy 3.2.4<br>GWAF 2.2<br>GWASTools 1.30.1<br>HIBAG 1.4<br>IGV 2.4.17

LDSC 1.0.1
Ldstore 2.0
Leafcutter 0.2.9
Liftover
MAGMA 1.08
METAL v2011-03-25
MetaXcan 0.6.12
Michigan Imputation Server 1.2.4
Minimac 3 and 4-1.0.2
minimap 2.17
mosdepth 0.2.9
NanoStat 1.1.2
NCBI remap
PBWT 3.1
Picard 2.22.6
PLINK 1.9 and 2.0
pygenometracks 3.5
qcat 1.0.1
R 3.6.0, 3.6.1 and 3.6.3
RegTools 0.5.1
RNASeQC 2.3.5
R package GenABEL 1.8-0
R package haplo.stats 1.8.6
R package ieugwasr 0.1.5
R package metafor 3.0.2
R package pec 2020.11.17
R package riskRegression 2020.12.8
R package stats 3.6.2
R package survIDINRI 1.1.1
R package survival 3.2.11
SAIGE 0.36.3.2 and 0.36.4
SamJdk 9750c96
Samtools 1.9
SeqMeta 1.6.7
SNPRelate 1.18.1
SNPTEST 2.5.3, 2.5.4-beta3 and 2.5.6
STAR 2.7.3a
STRING v11
vcffilterjdk v9750c96

For manuscripts utilizing custom algorithms or software that are central to the research but not yet described in published literature, software must be made available to editors and reviewers. We strongly encourage code deposition in a community repository (e.g. GitHub). See the Nature Research guidelines for submitting code & software for further information.

Data

Policy information about availability of data

All manuscripts must include a data availability statement. This statement should provide the following information, where applicable:

- Accession codes, unique identifiers, or web links for publicly available datasets
- A list of figures that have associated raw data
- A description of any restrictions on data availability

Genome-wide summary statistics have been deposited to the European Bioinformatics Institute GWAS Catalog (https://www.ebi.ac.uk/gwas/) under accession no. GCST90027158.
The significant e/sQTLs mapped and e/sTWAS functional reference panel weights generated for this study (in AD-relevant bulk brain regions from AMP-AD cohorts and in LCLs from the EADB Belgian cohort) are publicly available at https://doi.org/10.5281/zenodo.5745927 and https://doi.org/10.5281/zenodo.5745929.
Anonymized aligned reads of the amplicon-based long-read nanopore cDNA sequencing experiment conducted for the TSPAN14 splicing analysis are available through ENA under accession PRJEB49234.
Moreover, the following data used in the gene prioritization are publicly available:
AMP-AD rnaSeqReprocessing Study: https://www.synapse.org/#!Synapse:syn9702085
MayoRNAseq WGS VCFs: https://www.synapse.org/#!Synapse:syn11724002
ROSMAP WGS VCFs: https://www.synapse.org/#!Synapse:syn11724057
MSBB WGS VCFs: https://www.synapse.org/#!Synapse:syn11723899
eQTLGen: https://www.eqtlgen.org/
eQTL Catalogue database: https://www.ebi.ac.uk/eqtl/
Brain xQTL serve: http://mostafavilab.stat.ubc.ca/xqtl/
GTEx v8 eQTL and sQTL catalogues: https://www.gtexportal.org/
GTEx v8 expression and splicing prediction models: http://predictdb.org/
MiGA eQTLs: https://doi.org/10.5281/zenodo.4118605
MiGA sQTLs: https://doi.org/10.5281/zenodo.4118403
MiGA Meta-analysis: https://doi.org/10.5281/zenodo.4118676
Wingo et al. pQTL data: https://www.synapse.org/#!Synapse:syn23627957

Field-specific reporting

Please select the one below that is the best fit for your research. If you are not sure, read the appropriate sections before making your selection.

☒ Life sciences ☐ Behavioural & social sciences ☐ Ecological, evolutionary & environmental sciences

For a reference copy of the document with all sections, see nature.com/documents/nr-reporting-summary-flat.pdf

Life sciences study design

All studies must disclose on these points even when the disclosure is negative.

Sample size	Raw data used in this study was collected by the EADB consortia and summary statistics were recruited by external sources used for meta-analysis. Sample size was not pre-determined and was chosen based on all known available cohorts with relevant data collected to date, after quality control steps were performed in each cohort (described in detail in Supplementary Information) in particular to avoid any sample duplications. The sample size was calculated as the number of individuals summed across all studies in the meta-analysis, N=487,511.
Data exclusions	We exluded samples and variants based on standard quality control procedures for GWAS (Samples: Heterozygosity and missingness, Population outliers, Sex-check, Relatedness, Possibly problematic chips batch; Variants: Missingness and Hardy-Weinberg equilibrium, Frequency checks, Ambiguous variants, Duplicated variants). Complete details of our quality control procedures are provided in the methods and supplementary information section of the manuscript.
Replication	The meta-analysis strategy includes replication by default, as it weights the reported test statistics by the evidence of association across multiple samples. Further, SNP-based replication was carried out for the top GWAS association signals in an independent sample (N= 25,392 Alzheimer's disease cases and 276,086 controls; see Methods and supplementary information).
Randomization	Samples were randomized by case and control status on plates during genotyping at their independent study sites.
Blinding	genotyping was done blind without knowing the status of the individuals. The analysts were not blinded to the status of the individuals because QC procedures require knowing case and control status.

Reporting for specific materials, systems and methods

We require information from authors about some types of materials, experimental systems and methods used in many studies. Here, indicate whether each material, system or method listed is relevant to your study. If you are not sure if a list item applies to your research, read the appropriate section before selecting a response.

Materials & experimental systems

n/a	Involved in the study	
☒	☐	Antibodies
☒	☐	Eukaryotic cell lines
☒	☐	Palaeontology and archaeology
☒	☐	Animals and other organisms
☐	☒	Human research participants
☒	☐	Clinical data
☒	☐	Dual use research of concern

Methods

n/a	Involved in the study	
☒	☐	ChIP-seq
☒	☐	Flow cytometry
☒	☐	MRI-based neuroimaging

Human research participants

Policy information about studies involving human research participants

Population characteristics	We used multiple independent sets of participants in this study. We adjusted the analysis for principal components. Sample sizes, age and gender characteristics for our sample can be found per cohort and overall in Supplementary Tables 1 and Supplementary Information.
Recruitment	Participants from case-control studies were primarily recruited from clinics, nursing homes, disease registries, and hospitals, with controls being drawn from various ongoing studies and screened to exclude dementia/cognitive decline (see description of the samples in the supplmentary information). Cases were recruited according to clincal diagnsosis and defined as probable AD cases with a potential risk of misdiagnosis (estimated between 10 and 20% in the litterature). Controls included in the study were free of cognitive decline but a large part of them did not have any follow-up wit the possiblity that they developed dementia years later.<br>The UK Biobank recruited adult volunteers from national health registration records. UK Biobank participants are healthier than the general population, but since the data used in this study referred to parental diagnoses, the impact of selection bias should be minor.

Ethics oversight	Written informed consent was obtained from study participants or, for those with substantial cognitive impairment, from a caregiver, legal guardian, or other proxy, and the study protocols for all populations were reviewed and approved by the appropriate local Institutional review boards (see description of the samples in the supplementary information).

Note that full information on the approval of the study protocol must also be provided in the manuscript.
